# Patterns of Gene Expression in Peripheral Blood Mononuclear Cells and Outcomes from Patients with Sepsis Secondary to Community Acquired Pneumonia

**DOI:** 10.1371/journal.pone.0091886

**Published:** 2014-03-25

**Authors:** Patricia Severino, Eliézer Silva, Giovana Lotici Baggio-Zappia, Milena Karina Coló Brunialti, Laura Alejandra Nucci, Otelo Rigato Jr., Ismael Dale Cotrim Guerreiro da Silva, Flávia Ribeiro Machado, Reinaldo Salomao

**Affiliations:** 1 Center for Experimental Research, Instituto Israelita de Ensino e Pesquisa, Hospital Israelita Albert Einstein, São Paulo, Brazil; 2 Intensive Care Unit, Hospital Israelita Albert Einstein, São Paulo, Brazil; 3 Division of Infectious Diseases, Hospital São Paulo, Escola Paulista de Medicina (EPM), Universidade Federal de São Paulo (Unifesp), São Paulo, Brazil; 4 Intensive Care Unit, Hospital Sírio Libanês, São Paulo, Brazil; 5 Department of Gynecology, Escola Paulista de Medicina (EPM), Universidade Federal de São Paulo (Unifesp), São Paulo, Brazil; 6 Intensive Care Unit, Hospital São Paulo, Universidade Federal de São Paulo (Unifesp), São Paulo, Brazil; Charité, Campus Benjamin Franklin, Germany

## Abstract

Mechanisms governing the inflammatory response during sepsis have been shown to be complex, involving cross-talk between diverse signaling pathways. Current knowledge regarding the mechanisms underlying sepsis provides an incomplete picture of the syndrome, justifying additional efforts to understand this condition. Microarray-based expression profiling is a powerful approach for the investigation of complex clinical conditions such as sepsis. In this study, we investigate whole-genome expression profiles in mononuclear cells from survivors (n = 5) and non-survivors (n = 5) of sepsis. To circumvent the heterogeneity of septic patients, only patients admitted with sepsis caused by community-acquired pneumonia were included. Blood samples were collected at the time of sepsis diagnosis and seven days later to evaluate the role of biological processes or genes possibly involved in patient recovery. Principal Components Analysis (PCA) profiling discriminated between patients with early sepsis and healthy individuals. Genes with differential expression were grouped according to Gene Ontology, and most genes related to immune defense were up-regulated in septic patients. Additionally, PCA in the early stage was able to distinguish survivors from non-survivors. Differences in oxidative phosphorylation seem to be associated with clinical outcome because significant differences in the expression profile of genes related to mitochondrial electron transport chain (ETC) I–V were observed between survivors and non-survivors at the time of patient enrollment. Global gene expression profiles after seven days of sepsis progression seem to reproduce, to a certain extent, patterns collected at the time of diagnosis. Gene expression profiles comparing admission and follow-up samples differed between survivors and non-survivors, with decreased expression of genes related to immune functions in non-survivors. In conclusion, genes related to host defense and inflammatory response ontology were up-regulated during sepsis, consistent with the need for a host response to infection, and the sustainability of their expression in follow-up samples was associated with outcomes.

## Introduction

Sepsis has been defined as a systemic inflammatory response secondary to a proven or suspected infection [1]. Mechanisms governing this inflammatory response have been shown to be complex and dynamic [2]. A compensatory anti-inflammatory response (CARS) also takes place during sepsis, and the balance between both responses may underlie the pathophysiology of the syndrome [3]. Cell functional studies have underscored that the state of inflammatory response in sepsis is followed by a state of hypo-responsiveness or immunosuppression, which makes patients susceptible to late-stage infections with increased lethality [4], [5].

Microarray-based expression profiling is a powerful approach for the investigation of complex clinical conditions: the analysis of gene transcription at the genome level in sepsis potentially avoids results derived from biased assumptions. The application of microarray technology for biomarker discovery as well as for the comprehension of underlying mechanisms in sepsis and septic shock has been recently reviewed in the literature [6]. Two main approaches are readily distinguishable: experimental studies including endotoxemia studies in human volunteers [7], [8] and sepsis in experimental animals [9], and microarray-based studies targeting patients with sepsis or septic shock [10]–[12]. Despite the clear advantages of the controlled and reproducible first approach, which allows the investigator to overcome sample complexity, models are limited and cannot fully represent the inherent heterogeneity of clinical sepsis.

Patient-focused studies have produced findings on the hyperactivity of pathogen recognition receptors and signaling cascade pathways in sepsis, corroborating classical paradigms in sepsis research, but have not reached consensus regarding the two-phase model of an initial hyper-inflammatory phase followed by a compensatory anti-inflammatory phase [13], [14]. An alternate paradigm suggests that adaptive immune dysfunction is an early feature in sepsis, as has been reported in studies addressing the gene expression profiles of peripheral blood leukocytes after endotoxin challenge in humans [8] and mononuclear cell-specific gene expression profiles [12], [15].

Studies evaluating gene expression in LPS-induced tolerance models have supported a distinct scenario in which LPS-tolerant cells presenting tolerant (T) and non-tolerant (NT) genes are driven to control inflammation, yet preserving important functions, such as antimicrobial activity [16], [17].

Thus, the current state of knowledge on mechanisms underlying sepsis is far from providing a conclusive picture of the syndrome, justifying additional efforts to characterize the condition. In this study, we investigate whole-genome gene expression profiles of mononuclear cells from survivors and non-survivors of sepsis. Blood samples were collected at the time of sepsis diagnosis and seven days later, allowing us to evaluate the role of biological processes or genes that may be involved in patient recovery. Aiming to at least partially circumvent the heterogeneity of septic patient populations, we included only patients admitted with sepsis caused by community-acquired pneumonia.

## Materials and Methods

### Patients and Healthy Volunteers

A cohort of septic patients was enrolled from the Intensive Care Units of three general hospitals located in São Paulo, Brazil. This study was approved by the ethics committees of the participating hospitals, São Paulo Hospital (Study number 1477/06), Albert Einstein Hospital (Study number 07/549) and Sírio Libanês Hospital (Study number 2006/27). Written informed consent was obtained from all participants or, when necessary, from relatives before enrollment in the study protocol. Patients older than 18 years were enrolled within 48 hours of the first occurrence of organ dysfunction indicative of severe sepsis or septic shock. Exclusion criteria included patients under 18 years old, patients with immunosuppressive therapy, AIDS or end stage chronic illness, or who had been submitted to experimental therapy. Ten septic patients with community-acquired pneumonia as the primary source of infection were selected for this study, five of whom survived and five of whom died during hospitalization. Three healthy volunteers were enrolled as controls.

### Blood samples

Fifty milliliters of blood were collected in sodium heparin-treated tubes (BD Biosciences, Franklin Lakes, NJ, USA) from healthy volunteers and septic patients. Samples from septic patients were collected at two time points: D0 (within 48 hours of the first occurrence of organ dysfunction indicative of severe sepsis or septic shock) and D7 (seven days after the first sample was collected). Peripheral blood mononuclear cells were obtained using the Ficoll gradient method (Ficoll-Paque PLUS; GE Healthcare Bio-Sciences AB, Uppsala, Sweden). Cells were frozen in fetal bovine serum (Invitrogen-Gibco, Gaithersburg, MD, USA) with 10% dimethyl sulfoxide (Calbiochem, La Jolla, CA, USA) and stored in liquid nitrogen until use. The standard cell concentration was 1×10^7^ cells/mL.

### RNA extraction

Total RNA was isolated from peripheral mononuclear cells using an illustra RNAspin Mini Kit (GE Healthcare Bio-Sciences AB). The quality and concentration of the RNA was determined using an RNA Nano Chip Kit and a 2100 Bioanalyzer (Agilent Technologies, Santa Clara, CA, USA).

### Microarray analysis

Microarray analysis was performed using Agilent Whole Human Genome Microarray 4×44K arrays and the One Color Quick Amp Labeling Kit (Agilent Technologies). Hybridization and washing were performed according to the manufacturer's protocols (Agilent Technologies). The arrays were scanned using a GenePix 4000B Scanner (Axon) and analyzed using the Agilent Feature extraction software (version 9.5). The quality of the microarray data was assessed using the standard Agilent controls to verify expected quality control criteria. The gProcessedSignal from each array was loaded into the Partek Genomics Suite (v6.6), normalized between arrays using quantile normalization, and log transformed. We used Principal Components Analysis (PCA) as an exploratory tool to identify major effects influencing data. For subsequent statistical analysis we used the ANOVA implementation of Partek. The ANOVA model was defined by the experimental design and included variations due to volunteer group (sepsis, control), day of sepsis sample collection (D0, D7) and survival status (survivor, non-survivor). Patterns related to biological function were then assessed using Gene Ontology (GO) term enrichment analysis and KEGG pathway mapping through DAVID Bioinformatics Resources 6.7 (http://david.abcc.ncifcrf.gov). The raw microarray data can be assessed at Gene Expression Omnibus (GEO accession GSE48080). The cut-off for differential expression took into consideration the characteristics of each experiment. These data are reported in the results section.

## Results

### Patients and healthy volunteers

All patients included in the study were males with community acquired pneumonia (CAP) who either succumbed to or survived their sepsis episode, with age ranging from 25 to 92 years. Five patients were admitted with severe sepsis and five with septic shock. APACHEII scores ranged from 7 to 23, and SOFA scores ranged from 2 to 11 at enrollment (Table 1). Healthy controls, two females and one male, were 36, 58 and 84 years old.

**Table 1:** Demographic data and outcomes from septic patients included in this study.

Patients	Age (years)	Status at admission	Vasopressors	Apache II	SOFA	Outcome
P107	82	Septic shock	Yes	23	6	Alive
P140	47	Severe sepsis	Yes	16	11	Alive
P143	71	Severe sepsis	No	10	3	Alive
P146	57	Septic shock	Yes	14	10	Dead
P217	83	Severe sepsis	No	14	4	Dead
P227	92	Septic shock	Yes	12	10	Dead
P229	32	Severe sepsis	No	7	7	Alive
P239	84	Septic shock	Yes	19	9	Dead
P254	58	Septic shock	Yes	23	9	Dead
P260	25	Severe sepsis	No	15	2	Alive

### Global gene expression analysis characterizes septic patients

**Figure 1:**
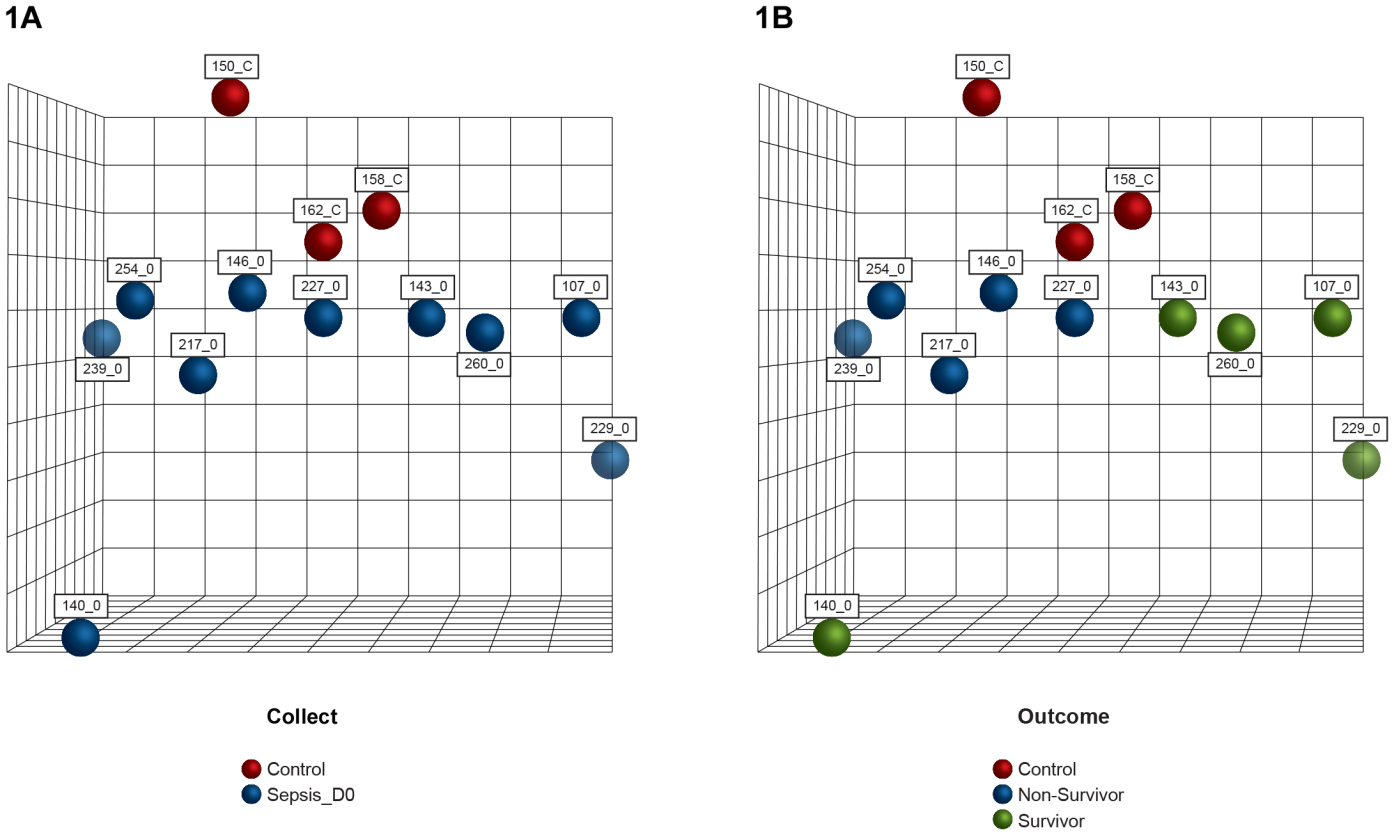
Global gene expression of healthy controls and septic patients. Unsupervised classification by principal components analysis of septic patients and controls. Principal components analysis (PCA) was used to classify 10 patients and 3 controls based on global gene expression. **A**: Septic patients cluster separately from healthy individuals at D0 (time of sepsis diagnosis). **B**: Global gene expression distinguishes survivors from non-survivors at D0. Numbers refers to the identification of patients and healthy volunteers.

Gene expression profiling by means of DNA microarrays was used to assess the behavior of biological processes characterizing septic patients. We performed principal components analysis and GO and KEGG term enrichment analyses to evaluate global patterns of expression between groups of individuals. The first analysis considered all septic patients at the moment of sepsis diagnosis (D0) compared to healthy individuals. Figure 1 depicts global differences in gene expression, as identified by PCA. Septic patients appear to cluster separately from healthy individuals (Figure 1A), and two distinct groups based on outcome are also apparent (Figure 1B).

One hundred and fifty one genes presented at least a 1.7-fold-change in gene expression between septic patients (D0) and healthy individuals: 104 were up-regulated and 49 were down-regulated in septic patients (Table S1, p≤0.05). To identify biological processes potentially connected with the septic phenotype, these genes were grouped by Gene Ontology (Table 2). Notably, most genes related to immune defense were up-regulated in septic patients.

**Table 2:** Gene Ontology (GO) terms significantly represented among genes differentially expressed between healthy individuals and patients at the time of sepsis diagnosis.

Term	Genes	p-value (FDR)
Response to wounding GO:0009611	**TNF** *, HPS6, **CXCL3, CCR1, CXCL2, CLU, TLR2, TLR4, CCL4**, IGSF10, **DYSF, CCL3L3, IL1B, NFKBIZ, CR1, IL8, PLEK, SOD2, S100A12, CD55, F5, ADM, STAB1, RIPK2, PTAFR**	9.48E-09
Inflammatory response GO:0006954	**NFKBIZ, CR1, TNF, IL8, CCR1, CXCL3, CLU, CXCL2, TLR2, TLR4, CCL4, S100A12, CD55, STAB1, CCL3L3, RIPK2, IL1B, PTAFR**	1.91E-06
Defense response GO:0006952	**NFKBIZ, CR1, TNF**, KLRC3, **ADORA2B, IL8, RNASE3, CCR1, CXCL3, CLU, CXCL2**, CD160, **TLR2, TLR4, CCL4, S100A12, GCH1, CD55, STAB1, CCL3L3, RIPK2, IL1B, PTAFR**	7.09E-06
Response to bacterium GO:0009617	**TNF, RNASE3, TLR2**, FASLG, **TLR4, GCH1, S100A12, ADM, STAB1, RIPK2, IL1B, PTAFR, MGST1**	9.62E-04
Positive regulation of interleukin-6 production GO:0032755	**TNF, ADORA2B, TLR2, IL1B, RIPK2, TLR4**	5.97E-04
Response to molecule of bacterial origin GO:0002237	**ADM, TLR2, IL1B, RIPK2**, FASLG, **TLR4, MGST1, PTAFR, GCH1**	8.23E-04
Immune response GO:0006955	**ICAM1, CR1, TNF, AQP9, IL8, CCR1, CXCL3, CLU, CXCL2, TLR2**, **FASLG, TLR4, CCL4, GCH1, CD55, CCL3L3, IL1B, CLEC4D, TREM1, PTAFR**	0.005040035
Response to lipopolysaccharides GO:0032496	**ADM, IL1B, RIPK2**, FASLG, **TLR4, MGST1, PTAFR, GCH1**	0.00513737
Chemotaxis GO:0006935	**RNASE2, IL8, CXCL3, CCR1, CXCL2, CCL3L3, IL1B**, AMOT, **CCL4, PTAFR**	0.010807363
Regulation of interleukin-6 production GO:0032675	**TNF, ADORA2B, TLR2, IL1B, RIPK2, TLR4**	0.017318007
Positive regulation of NF-κB transcription factor activity GO:0051092	**ICAM1, TNF, TLR2, IL1B, RIPK2, TLR4**	0.033317766

*Genes in bold: increased expression in septic patients than in healthy volunteers.

### Oxidative phosphorylation in early sepsis might be associated with patient outcome

When comparing data from survivors and non-survivors at the time of diagnosis (D0), focusing on genes with at least a 1.7-fold change and p-value <0.05, we observed no consistent dysregulation of biological processes (Table S2). However, we considered all genes that presented a significant variation based on the FDR corrected p-value, including some genes with less than a 1.7-fold-change, and identified 28 genes implicated in energy metabolism. The genes - UQCRC2, NDUFB5, NDUFB6, COX10, ATP5B, COX7C, COX5A, COX5B, NDUFB1, ATP5J, NDUFA4, NDUFA5, COX7A2, NDUFA8, NDUFA9, NDUFA7, COX4I2, NDUFC2, ATP5F1, NDUFA10, PPA2, NDUFA11, PPA1, ATP6V1C1, ATP6V1E1, ATP6V1E2, COX6A2, and ATP5C1 - exhibited mild but consistent variation in expression between the two groups.

### Sepsis progression and patient outcome

**Figure 2:**
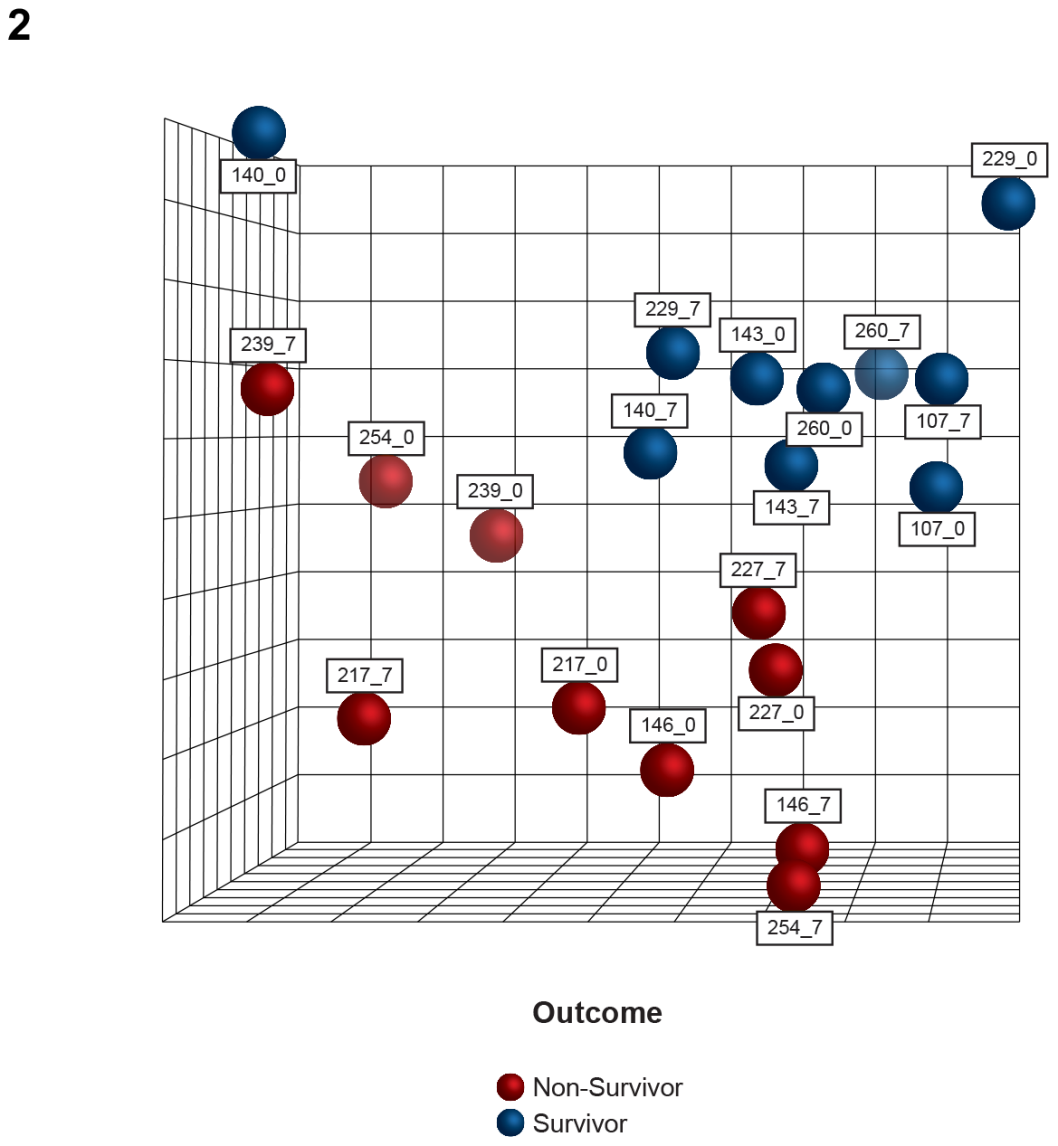
Global gene expression and patient outcome. Unsupervised classification by principal components analysis (PCA) of septic patients considering the outcome. Two separate clusters depict survivors and non-survivors, and this result is independent of the date of sample collection. Numbers refers to patients' identification and the day of sample collection (0 or 7).

With the aim of analyzing biological processes associated with sepsis progression, blood samples were collected 7 days after diagnosis (D7). A comparison of gene expression profiles on D0 and D7 reveals that patterns observed in PCA on D0 persist on D7, as depicted in Figure 2.

When comparing samples from survivors on D0 and D7 time points, although we observed differences in gene expression between the two groups, such differences were not robust enough to generate an acceptable p value (Table S3) and we were not able to identify a clear deregulation in cellular processes. For non-survivors, despite the fact that gene expression differences were not robust either (Table S4) through the analysis of these differences a broader induction of genes involved in immune response was seen on D0 when compared with D7 (Table 3).

**Table 3:** Gene Ontology (GO) terms significantly represented among genes differentially expressed between D0 and D7 time points for non-survivors.

Term	Genes*	p-value (FDR)
Inflammatory response GO:0006954	IL6, TNF, CCL2, OLR1, ADORA2A, KL, CFB, CCR1, CXCL3, CXCL2, IL1RN, NFKB1, CCL7, CXCL10, TNFAIP6, SIGLEC1, IL23A, CCL23, SAA1, PTX3, IL1A	1.07E-08
Defense response GO:0006952	TNF, CCL2, ADORA2A, CXCL3, CCR1, CXCL2, NFKB1, CD74, CCL7, CXCL10, IL23A, CCL23, SAA1, PTX3, IL1A, PLD1, IL6, OLR1, KL, CFB, IL1RN, TNFAIP6, SIGLEC1, IFNB1, CLEC5A	9.56E-07
Response to wounding GO:0009611	IL6, TNF, CCL2, OLR1, ADORA2A, KL, CFB, CCR1, CXCL3, CXCL2, IL1RN, NFKB1, CCL7, CXCL10, SIGLEC1, TNFAIP6, IL23A, CCL23, FGA, SAA1, PDGFRA, PTX3, IL1A	1.77E-06
Immune response GO:0006955	CSF3, TNF, CCL2, CXCL3, CCR1, CXCL2, OAS3, IFI44L, CCL7, CD74, CXCL10, IL23A, CCL23, PTX3, IL1A, IL6, OLR1, PTGER4, CFB, IL1RN, STXBP2, HLA-DQA2, OASL, TREM1, CLEC5A, GBP1	1.84E-06
Chemotaxis GO:0006935	CCRL2, PLD1, IL6, CCL23, CCL2, SAA1, CXCL3, CCR1, CXCL2, ITGA1, CCL7, CXCL10	2.59E-04
Cytokine-mediated signaling pathway GO:0019221	CSF3, IL6, TNF, CCL2, CCR1, DUOX1, IL1A	5.56E-02

*All genes presented increased expression on D0 than on D7.

Additionally we compared the D7 time point between survivor and non-survivors. Functional clustering of genes up-regulated on D7 in survivors compared with non-survivors included immune response (GO:0006955), defense response (GO:0006952), cell activation (GO:0001775) and antigen processing and presentation of peptide or polysaccharide antigen via MHC class II (GO:0002504) (Table 4). Global results comparing survivors and non-survivors on D7 are reported in Table S5.

**Table 4:** Gene Ontology (GO) terms significantly represented among genes differentially expressed between D7 samples for survivors and non-survivors.

Term	Genes*	p-value (FDR)
Immune response GO:0006955	KYNU, NBN, HLA-DRB1, HLA-DRB3, TLR1, OAS3, CCL8, STXBP2, TLR4, CTSS, HLA-DMA, HLA-DQA1, CD74, CD86, BPI, LILRA3, CLEC4A, LTF, CLEC7A, TREM1, CLEC5A, HLA-DRA	4.55E-06
Antigen processing and presentation of peptide or polysaccharide antigen via MHC class II GO:0002504	HLA-DRB1, HLA-DRB3, HLA-DMA, CD74, HLA-DQA1, HLA-DRA	1.74E-05
Defense response GO:0006952	PLD1, KYNU, CAMP, TLR1, SOCS6, CCL8, TLR4, CD74, SIGLEC1, BPI, LILRA3, DEFA4, LTF, CLEC7A, CLEC5A, HLA-DRA	1.21E-03
Cell activation GO:0001775	EGR1, NBN, CD86, PIK3CB, TLR1, STXBP2, TLR4, CLEC7A, HLA-DMA, CD74	2.34E-03

*All genes presented increased expression in survivors than in non-survivors.

## Discussion

The cohort of septic patients included in this study is representative of patients admitted in Intensive Care Units with CAP as a source of primary infection. The majority of patients were elderly, half of them in septic shock. Most of the patients with septic shock did not survive, while patients diagnosed with severe sepsis survived and were discharged. Two exceptions were patient P107, a septic shock patient who survived, and P217, a severe septic patient who died.

Gene expression profile in septic patients revealed differential regulation compared to healthy volunteers. PCA clearly segregated patients and healthy volunteers. Interestingly, global gene expression clustered P107 together with severe sepsis and P217 with septic shock, an indication that patient responses at early stages of sepsis could be indicative of outcome.

The expression of genes involved in response to bacteria in septic patients was noteworthy in our data (GO:0006952, GO:0009617, GO:0002237 and GO:0032496). Among these genes, we highlight the overexpression of TLR2 and TLR4 in septic patients. These genes are implicated in the recognition of bacterial cell wall components, such as lipopolysaccharide (LPS), and play a key role in the host response to infection [18]–[20]. TRL2 and TLR4, together with ICAM1, TNF, IL1B and RIPK2, are implicated in the positive regulation of nuclear factor-κB (NF-κB) (GO:0051092). This transcription factor has been reported to be critical for the expression of cytokines involved in inflammatory diseases such as sepsis syndrome, albeit through complex activation pathways [21]. Although NF-κB is not down-regulated at the transcript level in the samples studied here, the gene encoding NF-κB inhibitor zeta (NFKBIZ) is highly expressed in samples from septic patients. [22]. The expression of IL8, TNF, CXCL and IL1B in septic patients is implicated in the regulation of several interconnected pathways. These mediators interact within the NOD-Like receptor signaling pathway (http://www.genome.jp/kegg/pathway/hsa/hsa04621.html) and also act together with TRL2 and TRL4, elements of the Toll-like receptor signaling pathway (hsa04620:Toll-like receptor signaling pathway), in response to bacterial infection. Additionally, they may interact with CCR1, CCL3L3, and CCL4, elements of the Cytokine-cytokine receptor interaction pathway (http://www.genome.jp/kegg/pathway/hsa/hsa04060.html) that are similarly highly expressed in patient samples.

In addition, chemotaxis-related genes were identified as significantly dysregulated between the two groups (GO:0006935). The expression of CXCL2 is noteworthy because it is consistently up-regulated in early sepsis when compared to healthy individuals, and polymorphisms in this gene have been associated with outcomes in severe sepsis [23]. Chemotaxis is a complex process that leads to cell migration to the site of infection. This process involves endothelial activation by cytokines and the production of chemokines. Additionally, chemotaxis depends on the expression of chemokines receptors, L-selectins and integrins, which are involved in the activation, rolling and adhesion of leukocytes to endothelial cells, and in transmigration to the infected tissue. The increased expression of chemotaxis-related genes in mononuclear cells in samples collected at the time of admission suggests that these cells are recruited to infectious/inflammatory sites. This finding contrasts with functional studies evaluating neutrophil chemotaxis during lethal cecal ligation and puncture CLP sepsis. Reduced neutrophil migration to the site of infection is associated with a worse prognosis during sepsis [24]. Moreover, CXCR2, a chemokine receptor involved in neutrophil migration to sites of injury, was observed to be reduced on the surface of neutrophils from septic patients compared to healthy volunteers in our cohort (unpublished data) and in previous works [25], [26]. It has been shown that mice subjected to CLP show deficient neutrophil migration to the site of infection during severe sepsis, which is associated with decreased expression of CXCR2 on the cell surface [27].

Genes involved in different aspects of oxidative phosphorylation (http://www.genome.jp/kegg-bin/show_pathway?map00190) were found to be modulated in septic patients. Their products are components of mitochondrial electron transport chain (ETC) I–V. Interestingly, the majority of these differentially expressed genes, except for COX4I2 and COX6A2, were up-regulated in survivors compared to non-survivors patients, suggesting an increased level of mitochondrial dysfunction in the latter group.

In mitochondria, cellular energy in the form of ATP is produced via oxidative phosphorylation. Mitochondria are the source and targets of reactive oxygen species (ROS). In healthy cells, the generation of ROS is tightly controlled, but in disease states (including sepsis), ROS production is increased, causing tissue damage [28]. Recent studies suggest that mitochondrial dysfunction induced by oxidative stress might be involved in sepsis-mediated organ damage [29]. Additionally, an association between mitochondrial dysfunction and sepsis outcomes has been proposed [30].

There is increased ROS generation during experimental and clinical sepsis. Plasma samples from patients with septic shock that were co-cultured with human umbilical vein endothelial cells induced ROS generation, an effect related to the severity of septic shock [31]. We and others have reported increased ROS generation by neutrophils from septic patients [32]–[34]. Increased inducible nitric oxide synthase (iNOS) expression and NO metabolites have also been observed during sepsis. We found that monocytes and neutrophils from septic patients present increased NO production [35]. In addition to their direct effects, NO and superoxide (O2^−^) spontaneously react to form the toxic peroxynitrite anion (ONOO^−^), which leads to cytotoxic and pro-inflammatory responses [36]. Elevated levels of circulating nitrotyrosine have been observed in patients with primary septic shock, and concentrations are higher in non-surviving patients compared to survivors [37], [38].

ROS, NO and ONOO^−^ have a toxic impact on mitochondria by inducing ETC dysfunction and apoptosis. The observed decreased expression of genes belonging to ETC I–V in non-survivors, which could reflect compromised mitochondrial respiratory function, fits well with the known deleterious effects of the oxygen and nitrogen reactive species in sepsis.

Dysfunctions in zinc homeostasis have also been implicated in oxidative stress, and zinc reduces ROS via several mechanisms [39]. Two zinc transporter families have been characterized: the zinc transporter (ZnT)/solute carrier 30a (Slc30a) family and the Zrt/Irt-like protein (ZIP)/solute carrier 39a (Slc39a) family [40]. Zinc concentration in plasma has been correlated with the expression of zinc transporter genes as well as with patient outcome following sepsis [41], [42]. Although we do not present data on zinc concentration, we did identify three members of the SLC39A family that are down-regulated in non-survivors compared to survivors at D0 (SLC39A6, SLC39A9 and SLC39A11). This is coupled with broad dysregulation of several genes involved in oxidative phosphorylation.

Neutrophils and monocytes are the primary cells of innate immunity in host defense against infecting microorganisms. They share a number of cell functions including phagocytosis and intracellular killing of pathogens, production of cytokines and generation of reactive oxygen species. Considering, however, the specificities of each cell type, it would be interesting to evaluate if the gene expression modulation observed in mononuclear cells in our study has the same profile in neutrophils. In a previous work evaluating the TLR signaling pathway in patients in different stages of sepsis we found differences between PBMC and neutrophils gene expression, with a trend of neutrophils to present a broader up-regulation [20].

Patient's samples were collected 7 days after admission allowing us to analyze gene expression profile following sepsis progression and therapeutic interventions.

PCA revealed that the patterns observed at admission persisted on follow-up samples in survivors and non-survivors, indicating that the host response to sepsis at the onset of the syndrome is critical for patient outcome.

We found, however, that the gene ontology profiles in follow-up samples (D0–D7) differed between survivors and non-survivors. In non-survivors genes involved in immune response were down-regulated on D7 compared to D0. Consistent with these results, gene expression profiles in D7 samples from survivors differed from non-survivors, with the immune system response more intense in survivors. Notably, differences on D7 support a model in which restoring the ability to induce adaptive immunity during therapy is relevant for patient recovery.

As noted by Tang *et al.*, sepsis elicits an inducible activation of pathogen recognition receptors accompanied by an increase in the activities of signal transduction cascades. Changes in inflammatory responses are highly variable between studies, and there are inconsistent changes in the expression of pivotal inflammatory/anti-inflammatory cytokines, e.g. TNF-α, IL-1 and IL-10 [13]. In part, this may reflect the timing of patient's enrollment, since in a previous work we found a dynamic modulation of ex-vivo induction of TNF-α and IL-6 in whole blood of septic patients related to the stages of sepsis [43].

In general we may consider that changes in gene expression in our patients reflect a comprehensive host-response to infection and the sustainability of their expression in follow-up samples is associated with outcomes. In addition to the above described induction of PRRs, the majority of up-regulated genes clustered ontologically in host-defense pathways. While this profile might be predicted for a patient fighting a potentially fatal infection, similar trends have not been reported in other studies. Rather, genomic studies have suggested that immune suppression predominates during sepsis. One study evaluating whole genome gene expression in mononuclear cells from patients with sepsis reported sepsis-related immunosuppression and reduced inflammatory responses [12]. However, this conclusion may overstate the role of the four functional clusters that differ between septic and SIRS patients. For example, a lymphocyte activation cluster was increased in septic patients, while immune function and inflammatory response clusters were increased in SIRS patients. In line with these results, the same group has recently published an interesting paper using whole blood from septic patients and healthy volunteers, in which an “Immune Suppression Integer” is proposed in an attempt to correlate gene dysregulation with clinical outcomes [44].

It is likely that the discrepancies between genomic studies performed in clinical settings reflects differences of objectives, inclusion criteria and approaches to evaluate gene expressions, e.g., mononuclear cells vs. whole blood, as reviewed by Tang *et al.* [13]. In fact, different studies may provide insight into different aspects of the multi-complex host response during sepsis. Multicenter studies with large numbers of patients might contribute to more uniform results.

There are limitations to the present work. One of the most important limitations is the sample size, which may have contributed to the exclusion of many sepsis-related changes in gene expression from the final analysis. Nevertheless, our focus on a single, community acquired infection identified significant patterns that were sufficient to cluster patients and healthy volunteers, as well as survivors and non-survivors. Genes that were differentially expressed could be clustered accordingly to gene ontology and pathway. Further, the time elapsed between admission and follow-up samples did not allow observing changes occurring in earlier days of intervention. Seven days interval is, however, in consonance with earlier functional studies, which revealed that blood cells from septic patients take several days to restore their ex-vivo responses [45], [46], and with a genomic study in trauma patients showing that in patients with uncomplicated recovery, gene expression tends to return to baseline within 7–14 d for both up- and down-regulated genes, while in complicated patients changes persisted longer period [47]. Finally, in this pilot study, we did not confirm the results reported here with quantitative PCR because we were interested in global patterns and pathways of immune response rather than specific biomarkers.

## Conclusions

Patients admitted with sepsis secondary to CAP exhibit a gene induction profile when compared to healthy controls. Specifically, genes clustered in host defense and inflammatory response ontology were up-regulated during sepsis, consistent with the needs for a host response to infection. Additionally, the patterns of gene expression were able to cluster patients who survived from those who succumbed to the infection. Comparisons of gene expression from samples collected at the time of admission and in follow-up samples identified differences between survivors and non-survivors, with decreased expression of genes related to immune functions in non-survivors. Further studies evaluating other primary sources of sepsis are needed to evaluate if this is a general gene expression profile or reflects a specific set of septic patients.

## Supporting Information

Table S1 **Differential gene expression between septic patients at the time of diagnosis (D0) and healthy controls.** Only genes exhibiting a fold change of at least 1.7 and a p-value <0.05 are reported.
(DOCX)

Table S2 **Differential gene expression between survivor and non-survivors at the time of diagnosis (D0).** Only genes presenting a FDR corrected p-value <0.05 are reported.
(DOCX)

Table S3 **Differential gene expression between survivors at D0 and survivors at D7.** Only genes presenting at least a 1.7 fold change are reported.
(DOCX)

Table S4 **Differential gene expression between non-survivors at D0 and non-survivors at D7.** Only genes presenting at least a 1.7 fold change are reported.
(DOCX)

Table S5 **Differential gene expression between survivors at D7 and non-survivors at D7.** Only genes presenting at least a 1.7 fold change and p value <0.05 are reported.
(DOCX)
