# Supplementary material for: Patterns of Gene Expression in Peripheral Blood Mononuclear Cells and Outcomes from Patients with Sepsis Secondary to Community Acquired Pneumonia
Source: PLoS One. 2014 Mar 25;9(3):e91886. doi: 10.1371/journal.pone.0091886 (PMC3965402; doi:10.1371/journal.pone.0091886)
Supplement: Table S1 — Differential gene expression between septic patients at the time of diagnosis (D0) and healthy controls. Only genes exhibiting a fold change of at least 1.7 and a p-value <0.05 are reported. (DOCX) [file pone.0091886.s001.docx]

**Table S1: Differential gene expression between septic patients at the time of diagnosis (D0) and healthy controls. Only genes exhibiting a fold change of at least 1.7 and a p-value < 0.05 are reported.**

| Gene symbol | p-value  (Sepsis_D0 vs. Control) | FoldChange  (Sepsis_D0 vs. Control) | FoldChange  Description |
| --- | --- | --- | --- |
| CD160 | 3.22E-03 | -2.98 | Sepsis_D0 down vs Control |
| SH2D1B | 1.92E-03 | -2.88 | Sepsis_D0 down vs Control |
| RPAP1 | 2.32E-03 | -2.81 | Sepsis_D0 down vs Control |
| BAIAP3 | 1.75E-03 | -2.55 | Sepsis_D0 down vs Control |
| KLRC3 | 2.19E-03 | -2.55 | Sepsis_D0 down vs Control |
| CDKN1C | 2.92E-03 | -2.51 | Sepsis_D0 down vs Control |
| FCRL3 | 1.45E-04 | -2.41 | Sepsis_D0 down vs Control |
| CGI-38 | 7.50E-06 | -2.29 | Sepsis_D0 down vs Control |
| GPR92 | 6.33E-04 | -2.23 | Sepsis_D0 down vs Control |
| ZNF367 | 5.82E-05 | -2.11 | Sepsis_D0 down vs Control |
| KLRC1 | 2.50E-03 | -2.11 | Sepsis_D0 down vs Control |
| P2RY5 | 2.47E-03 | -2.06 | Sepsis_D0 down vs Control |
| DDX28 | 2.30E-04 | -2.04 | Sepsis_D0 down vs Control |
| HPS6 | 6.66E-04 | -2.02 | Sepsis_D0 down vs Control |
| ZDHHC11 | 3.84E-03 | -2.01 | Sepsis_D0 down vs Control |
| EID3 | 3.16E-03 | -1.99 | Sepsis_D0 down vs Control |
| ZNF57 | 5.84E-04 | -1.99 | Sepsis_D0 down vs Control |
| CCDC65 | 4.73E-03 | -1.95 | Sepsis_D0 down vs Control |
| ZNF555 | 1.39E-04 | -1.93 | Sepsis_D0 down vs Control |
| FBXL16 | 3.37E-03 | -1.91 | Sepsis_D0 down vs Control |
| ZNF567 | 6.59E-04 | -1.88 | Sepsis_D0 down vs Control |
| IER5L | 2.36E-04 | -1.88 | Sepsis_D0 down vs Control |
| ZNF627 | 8.03E-04 | -1.88 | Sepsis_D0 down vs Control |
| ZNF329 | 3.77E-04 | -1.87 | Sepsis_D0 down vs Control |
| FAM102A | 4.37E-03 | -1.87 | Sepsis_D0 down vs Control |
| TRAF5 | 2.75E-03 | -1.86 | Sepsis_D0 down vs Control |
| PLEKHG3 | 1.99E-04 | -1.85 | Sepsis_D0 down vs Control |
| CCDC102A | 3.12E-03 | -1.84 | Sepsis_D0 down vs Control |
| FASLG | 1.14E-03 | -1.84 | Sepsis_D0 down vs Control |
| U2AF1L2 | 1.14E-03 | -1.80 | Sepsis_D0 down vs Control |
| PLEKHG1 | 4.08E-03 | -1.80 | Sepsis_D0 down vs Control |
| DYRK2 | 4.35E-04 | -1.80 | Sepsis_D0 down vs Control |
| TXNIP | 1.40E-04 | -1.79 | Sepsis_D0 down vs Control |
| ADAMTS1 | 3.65E-05 | -1.79 | Sepsis_D0 down vs Control |
| EPHA2 | 3.74E-05 | -1.79 | Sepsis_D0 down vs Control |
| NCAM1 | 4.80E-03 | -1.79 | Sepsis_D0 down vs Control |
| ATP9A | 4.26E-03 | -1.76 | Sepsis_D0 down vs Control |
| IGSF10 | 1.91E-03 | -1.76 | Sepsis_D0 down vs Control |
| SERTAD3 | 3.04E-05 | -1.75 | Sepsis_D0 down vs Control |
| FBXL21 | 4.91E-04 | -1.75 | Sepsis_D0 down vs Control |
| TBCC | 2.58E-06 | -1.74 | Sepsis_D0 down vs Control |
| AMOT | 9.34E-05 | -1.74 | Sepsis_D0 down vs Control |
| HERPUD1 | 3.08E-03 | -1.72 | Sepsis_D0 down vs Control |
| ING1 | 6.43E-04 | -1.72 | Sepsis_D0 down vs Control |
| IFIT5 | 2.38E-03 | -1.72 | Sepsis_D0 down vs Control |
| CAPN12 | 1.52E-03 | -1.71 | Sepsis_D0 down vs Control |
| RNF113A | 2.26E-03 | -1.71 | Sepsis_D0 down vs Control |
| PTAFR | 8.13E-04 | 1.70 | Sepsis_D0 up vs Control |
| TLR4 | 2.51E-03 | 1.71 | Sepsis_D0 up vs Control |
| TUBB2A | 1.08E-03 | 1.71 | Sepsis_D0 up vs Control |
| KLHL2 | 7.19E-04 | 1.71 | Sepsis_D0 up vs Control |
| MUC1 | 4.25E-03 | 1.71 | Sepsis_D0 up vs Control |
| UBAP1 | 2.48E-03 | 1.72 | Sepsis_D0 up vs Control |
| IGFBP7 | 1.71E-03 | 1.72 | Sepsis_D0 up vs Control |
| MYL6B | 1.64E-03 | 1.73 | Sepsis_D0 up vs Control |
| BLOC1S1 | 2.41E-06 | 1.73 | Sepsis_D0 up vs Control |
| NRAS | 8.53E-04 | 1.74 | Sepsis_D0 up vs Control |
| CLU | 4.42E-03 | 1.75 | Sepsis_D0 up vs Control |
| KRTAP8-1 | 2.13E-03 | 1.75 | Sepsis_D0 up vs Control |
| UBE2D1 | 1.25E-03 | 1.76 | Sepsis_D0 up vs Control |
| MRVI1 | 2.56E-03 | 1.78 | Sepsis_D0 up vs Control |
| HTRA4 | 4.97E-03 | 1.78 | Sepsis_D0 up vs Control |
| S100A11 | 2.81E-03 | 1.80 | Sepsis_D0 up vs Control |
| FUT4 | 3.69E-03 | 1.80 | Sepsis_D0 up vs Control |
| TMEM2 | 4.72E-03 | 1.80 | Sepsis_D0 up vs Control |
| NT5DC2 | 2.13E-03 | 1.81 | Sepsis_D0 up vs Control |
| WDFY3 | 6.35E-04 | 1.82 | Sepsis_D0 up vs Control |
| OPLAH | 4.60E-03 | 1.82 | Sepsis_D0 up vs Control |
| IPPK | 2.73E-03 | 1.82 | Sepsis_D0 up vs Control |
| KREMEN1 | 6.52E-04 | 1.83 | Sepsis_D0 up vs Control |
| IDH1 | 4.15E-03 | 1.84 | Sepsis_D0 up vs Control |
| PNPLA8 | 2.03E-03 | 1.84 | Sepsis_D0 up vs Control |
| PTPRE | 3.55E-03 | 1.84 | Sepsis_D0 up vs Control |
| RAB1A | 3.01E-03 | 1.86 | Sepsis_D0 up vs Control |
| GNA15 | 4.36E-03 | 1.87 | Sepsis_D0 up vs Control |
| CD93 | 4.16E-04 | 1.88 | Sepsis_D0 up vs Control |
| TMPO | 9.25E-04 | 1.89 | Sepsis_D0 up vs Control |
| TLR2 | 3.06E-03 | 1.91 | Sepsis_D0 up vs Control |
| GCH1 | 4.51E-03 | 1.96 | Sepsis_D0 up vs Control |
| SART2 | 7.89E-04 | 2.01 | Sepsis_D0 up vs Control |
| F5 | 1.85E-05 | 2.04 | Sepsis_D0 up vs Control |
| CTSD | 1.57E-03 | 2.05 | Sepsis_D0 up vs Control |
| SLC22A4 | 5.87E-04 | 2.06 | Sepsis_D0 up vs Control |
| TAF13 | 3.86E-03 | 2.06 | Sepsis_D0 up vs Control |
| CR1 | 4.06E-04 | 2.07 | Sepsis_D0 up vs Control |
| DGAT2 | 3.32E-03 | 2.08 | Sepsis_D0 up vs Control |
| PIGA | 4.61E-03 | 2.09 | Sepsis_D0 up vs Control |
| GAS7 | 7.07E-05 | 2.12 | Sepsis_D0 up vs Control |
| NP | 9.57E-05 | 2.14 | Sepsis_D0 up vs Control |
| DDEF1IT1 | 2.00E-03 | 2.14 | Sepsis_D0 up vs Control |
| MXD1 | 9.65E-04 | 2.21 | Sepsis_D0 up vs Control |
| PTDSR | 3.36E-03 | 2.21 | Sepsis_D0 up vs Control |
| S100A12 | 2.18E-04 | 2.22 | Sepsis_D0 up vs Control |
| CLEC4D | 5.43E-04 | 2.22 | Sepsis_D0 up vs Control |
| LTB4DH | 2.12E-03 | 2.23 | Sepsis_D0 up vs Control |
| SLC16A7 | 3.58E-03 | 2.23 | Sepsis_D0 up vs Control |
| MGST1 | 3.20E-05 | 2.24 | Sepsis_D0 up vs Control |
| ASGR2 | 3.68E-03 | 2.25 | Sepsis_D0 up vs Control |
| CCR1 | 2.43E-04 | 2.26 | Sepsis_D0 up vs Control |
| CDKN2B | 4.35E-03 | 2.26 | Sepsis_D0 up vs Control |
| TREM1 | 3.05E-03 | 2.27 | Sepsis_D0 up vs Control |
| UBE2J1 | 3.92E-03 | 2.32 | Sepsis_D0 up vs Control |
| ACPP | 3.01E-03 | 2.38 | Sepsis_D0 up vs Control |
| EFCAB2 | 2.94E-03 | 2.40 | Sepsis_D0 up vs Control |
| ADORA2B | 2.63E-03 | 2.42 | Sepsis_D0 up vs Control |
| ETS2 | 2.56E-03 | 2.44 | Sepsis_D0 up vs Control |
| TNFAIP3 | 2.71E-03 | 2.47 | Sepsis_D0 up vs Control |
| CIAS1 | 1.78E-03 | 2.49 | Sepsis_D0 up vs Control |
| DYSF | 1.62E-04 | 2.52 | Sepsis_D0 up vs Control |
| PLAC8 | 3.71E-03 | 2.52 | Sepsis_D0 up vs Control |
| EAF1 | 6.34E-04 | 2.53 | Sepsis_D0 up vs Control |
| CD55 | 3.64E-04 | 2.56 | Sepsis_D0 up vs Control |
| RIPK2 | 3.09E-03 | 2.57 | Sepsis_D0 up vs Control |
| B4GALT5 | 4.48E-03 | 2.58 | Sepsis_D0 up vs Control |
| GAS6 | 1.92E-03 | 2.64 | Sepsis_D0 up vs Control |
| PLEK | 3.17E-03 | 2.66 | Sepsis_D0 up vs Control |
| LIN7A | 7.50E-04 | 2.68 | Sepsis_D0 up vs Control |
| HRB | 2.18E-04 | 2.70 | Sepsis_D0 up vs Control |
| SULT1B1 | 2.52E-03 | 2.71 | Sepsis_D0 up vs Control |
| STAB1 | 9.48E-04 | 2.71 | Sepsis_D0 up vs Control |
| TNF | 2.68E-04 | 2.74 | Sepsis_D0 up vs Control |
| PDE4B | 1.72E-03 | 2.79 | Sepsis_D0 up vs Control |
| MCEMP1 | 2.72E-03 | 2.81 | Sepsis_D0 up vs Control |
| PPP1R15A | 1.03E-04 | 2.81 | Sepsis_D0 up vs Control |
| NFKBIZ | 7.61E-04 | 2.84 | Sepsis_D0 up vs Control |
| FPRL1 | 9.41E-04 | 2.91 | Sepsis_D0 up vs Control |
| AHR | 3.61E-04 | 2.93 | Sepsis_D0 up vs Control |
| PHLDA1 | 2.89E-03 | 3.06 | Sepsis_D0 up vs Control |
| METTL7B | 2.30E-03 | 3.08 | Sepsis_D0 up vs Control |
| IL1B | 4.05E-05 | 3.24 | Sepsis_D0 up vs Control |
| BCL2A1 | 3.82E-04 | 3.25 | Sepsis_D0 up vs Control |
| MAFB | 1.01E-04 | 3.31 | Sepsis_D0 up vs Control |
| SPRY1 | 3.09E-04 | 3.35 | Sepsis_D0 up vs Control |
| KLF5 | 2.44E-03 | 3.41 | Sepsis_D0 up vs Control |
| ADM | 7.64E-04 | 3.59 | Sepsis_D0 up vs Control |
| SOD2 | 3.15E-03 | 3.71 | Sepsis_D0 up vs Control |
| RNASE2 | 3.76E-03 | 3.72 | Sepsis_D0 up vs Control |
| PBEF1 | 3.12E-03 | 3.78 | Sepsis_D0 up vs Control |
| CCL4 | 2.44E-03 | 3.81 | Sepsis_D0 up vs Control |
| LDLR | 1.24E-03 | 3.82 | Sepsis_D0 up vs Control |
| SNAI1 | 4.48E-03 | 3.83 | Sepsis_D0 up vs Control |
| TNNT1 | 1.19E-03 | 4.06 | Sepsis_D0 up vs Control |
| RNASE3 | 2.96E-03 | 4.07 | Sepsis_D0 up vs Control |
| IL8 | 5.94E-04 | 4.11 | Sepsis_D0 up vs Control |
| AQP9 | 2.08E-04 | 4.39 | Sepsis_D0 up vs Control |
| CCL3L3 | 3.87E-05 | 4.63 | Sepsis_D0 up vs Control |
| MS4A4A | 3.98E-03 | 5.03 | Sepsis_D0 up vs Control |
| ICAM1 | 4.86E-03 | 5.14 | Sepsis_D0 up vs Control |
| CXCL3 | 2.29E-03 | 7.70 | Sepsis_D0 up vs Control |
| FAM20A | 1.16E-04 | 8.14 | Sepsis_D0 up vs Control |
| CXCL2 | 9.09E-06 | 9.44 | Sepsis_D0 up vs Control |
