# Supplementary material for: Patterns of Gene Expression in Peripheral Blood Mononuclear Cells and Outcomes from Patients with Sepsis Secondary to Community Acquired Pneumonia
Source: PLoS One. 2014 Mar 25;9(3):e91886. doi: 10.1371/journal.pone.0091886 (PMC3965402; doi:10.1371/journal.pone.0091886)
Supplement: Table S2 — Differential gene expression between survivor and non-survivors at the time of diagnosis (D0). Only genes presenting a FDR corrected p-value <0.05 are reported. (DOCX) [file pone.0091886.s002.docx]

**Table S2: Differential gene expression between survivor and non-survivors at the time of diagnosis (D0). Only genes presenting a FDR corrected p-value < 0.05 are reported.**

| Gene Symbol | p-value | FoldChange Sepsis_D0 non-survivor vs. Sepsis_D0 survivor | FoldChange Description |
| --- | --- | --- | --- |
| ALAS2 | 1.77E-03 | -6.03 | Sepsis_D0_non-survivor down vs Sepsis_D0_survivor |
| BPGM | 7.61E-03 | -3.85 | Sepsis_D0_non-survivor down vs Sepsis_D0_survivor |
| HP | 3.18E-03 | -3.55 | Sepsis_D0_non-survivor down vs Sepsis_D0_survivor |
| HPR | 1.45E-03 | -3.10 | Sepsis_D0_non-survivor down vs Sepsis_D0_survivor |
| HBD | 4.41E-04 | -3.08 | Sepsis_D0_non-survivor down vs Sepsis_D0_survivor |
| CMTM2 | 8.78E-04 | -3.05 | Sepsis_D0_non-survivor down vs Sepsis_D0_survivor |
| CR1L | 1.01E-03 | -2.96 | Sepsis_D0_non-survivor down vs Sepsis_D0_survivor |
| LCN2 | 2.26E-03 | -2.84 | Sepsis_D0_non-survivor down vs Sepsis_D0_survivor |
| CLEC12A | 5.42E-05 | -2.81 | Sepsis_D0_non-survivor down vs Sepsis_D0_survivor |
| MBOAT2 | 6.15E-04 | -2.67 | Sepsis_D0_non-survivor down vs Sepsis_D0_survivor |
| RPGRIP1 | 5.67E-03 | -2.63 | Sepsis_D0_non-survivor down vs Sepsis_D0_survivor |
| FECH | 2.66E-03 | -2.63 | Sepsis_D0_non-survivor down vs Sepsis_D0_survivor |
| YIPF4 | 9.59E-04 | -2.61 | Sepsis_D0_non-survivor down vs Sepsis_D0_survivor |
| AK3 | 2.52E-03 | -2.56 | Sepsis_D0_non-survivor down vs Sepsis_D0_survivor |
| MICA | 4.73E-03 | -2.49 | Sepsis_D0_non-survivor down vs Sepsis_D0_survivor |
| GUF1 | 3.28E-03 | -2.48 | Sepsis_D0_non-survivor down vs Sepsis_D0_survivor |
| CETN3 | 2.07E-03 | -2.44 | Sepsis_D0_non-survivor down vs Sepsis_D0_survivor |
| BRUNOL6 | 4.74E-03 | -2.41 | Sepsis_D0_non-survivor down vs Sepsis_D0_survivor |
| UBXD4 | 2.87E-03 | -2.41 | Sepsis_D0_non-survivor down vs Sepsis_D0_survivor |
| RBM12B | 1.85E-03 | -2.37 | Sepsis_D0_non-survivor down vs Sepsis_D0_survivor |
| CG018 | 1.06E-03 | -2.35 | Sepsis_D0_non-survivor down vs Sepsis_D0_survivor |
| HUS1 | 3.80E-04 | -2.35 | Sepsis_D0_non-survivor down vs Sepsis_D0_survivor |
| TMEM157 | 1.07E-05 | -2.34 | Sepsis_D0_non-survivor down vs Sepsis_D0_survivor |
| RRAGB | 4.03E-03 | -2.27 | Sepsis_D0_non-survivor down vs Sepsis_D0_survivor |
| ZCRB1 | 8.21E-04 | -2.25 | Sepsis_D0_non-survivor down vs Sepsis_D0_survivor |
| ACTA2 | 3.04E-04 | -2.25 | Sepsis_D0_non-survivor down vs Sepsis_D0_survivor |
| MKRN2 | 1.91E-03 | -2.24 | Sepsis_D0_non-survivor down vs Sepsis_D0_survivor |
| SPRY1 | 1.68E-04 | -2.24 | Sepsis_D0_non-survivor down vs Sepsis_D0_survivor |
| EFCAB2 | 5.20E-04 | -2.23 | Sepsis_D0_non-survivor down vs Sepsis_D0_survivor |
| FRAT1 | 8.74E-04 | -2.23 | Sepsis_D0_non-survivor down vs Sepsis_D0_survivor |
| ARMCX3 | 5.41E-03 | -2.21 | Sepsis_D0_non-survivor down vs Sepsis_D0_survivor |
| TARSL2 | 1.93E-04 | -2.19 | Sepsis_D0_non-survivor down vs Sepsis_D0_survivor |
| NSDHL | 4.54E-03 | -2.18 | Sepsis_D0_non-survivor down vs Sepsis_D0_survivor |
| GALNT4 | 1.06E-04 | -2.18 | Sepsis_D0_non-survivor down vs Sepsis_D0_survivor |
| TIGD2 | 2.11E-03 | -2.18 | Sepsis_D0_non-survivor down vs Sepsis_D0_survivor |
| GAS6 | 6.61E-04 | -2.16 | Sepsis_D0_non-survivor down vs Sepsis_D0_survivor |
| MFAP4 | 4.02E-03 | -2.15 | Sepsis_D0_non-survivor down vs Sepsis_D0_survivor |
| ZNF184 | 7.69E-04 | -2.14 | Sepsis_D0_non-survivor down vs Sepsis_D0_survivor |
| HACE1 | 8.26E-04 | -2.14 | Sepsis_D0_non-survivor down vs Sepsis_D0_survivor |
| TK2 | 6.11E-04 | -2.13 | Sepsis_D0_non-survivor down vs Sepsis_D0_survivor |
| LRRC35 | 1.13E-03 | -2.13 | Sepsis_D0_non-survivor down vs Sepsis_D0_survivor |
| RPP30 | 1.08E-03 | -2.12 | Sepsis_D0_non-survivor down vs Sepsis_D0_survivor |
| STCH | 1.00E-04 | -2.11 | Sepsis_D0_non-survivor down vs Sepsis_D0_survivor |
| ING2 | 3.80E-03 | -2.11 | Sepsis_D0_non-survivor down vs Sepsis_D0_survivor |
| MOSPD1 | 4.54E-03 | -2.11 | Sepsis_D0_non-survivor down vs Sepsis_D0_survivor |
| PARP16 | 2.79E-03 | -2.10 | Sepsis_D0_non-survivor down vs Sepsis_D0_survivor |
| ECSM2 | 8.97E-04 | -2.09 | Sepsis_D0_non-survivor down vs Sepsis_D0_survivor |
| CHEK2 | 4.50E-04 | -2.09 | Sepsis_D0_non-survivor down vs Sepsis_D0_survivor |
| MAP2K5 | 6.29E-04 | -2.09 | Sepsis_D0_non-survivor down vs Sepsis_D0_survivor |
| VAMP4 | 5.52E-03 | -2.08 | Sepsis_D0_non-survivor down vs Sepsis_D0_survivor |
| CHST9 | 8.43E-04 | -2.07 | Sepsis_D0_non-survivor down vs Sepsis_D0_survivor |
| HIST1H2AL | 3.18E-03 | -2.07 | Sepsis_D0_non-survivor down vs Sepsis_D0_survivor |
| CBR4 | 2.06E-03 | -2.07 | Sepsis_D0_non-survivor down vs Sepsis_D0_survivor |
| SULT1B1 | 1.38E-03 | -2.07 | Sepsis_D0_non-survivor down vs Sepsis_D0_survivor |
| ZAK | 5.80E-04 | -2.07 | Sepsis_D0_non-survivor down vs Sepsis_D0_survivor |
| APIP | 2.85E-03 | -2.07 | Sepsis_D0_non-survivor down vs Sepsis_D0_survivor |
| DCLRE1A | 3.09E-03 | -2.06 | Sepsis_D0_non-survivor down vs Sepsis_D0_survivor |
| AP4S1 | 4.47E-04 | -2.04 | Sepsis_D0_non-survivor down vs Sepsis_D0_survivor |
| ACVR1 | 5.02E-03 | -2.03 | Sepsis_D0_non-survivor down vs Sepsis_D0_survivor |
| EXOSC2 | 5.98E-03 | -2.03 | Sepsis_D0_non-survivor down vs Sepsis_D0_survivor |
| CSNK2A2 | 1.17E-03 | -2.02 | Sepsis_D0_non-survivor down vs Sepsis_D0_survivor |
| GOLPH4 | 6.70E-04 | -2.01 | Sepsis_D0_non-survivor down vs Sepsis_D0_survivor |
| UGCG | 3.04E-03 | -2.00 | Sepsis_D0_non-survivor down vs Sepsis_D0_survivor |
| HEL308 | 2.79E-03 | -2.00 | Sepsis_D0_non-survivor down vs Sepsis_D0_survivor |
| TMEM163 | 4.06E-04 | -2.00 | Sepsis_D0_non-survivor down vs Sepsis_D0_survivor |
| TRFP | 5.98E-03 | -2.00 | Sepsis_D0_non-survivor down vs Sepsis_D0_survivor |
| MPHOSPH6 | 2.20E-03 | -2.00 | Sepsis_D0_non-survivor down vs Sepsis_D0_survivor |
| ERMAP | 4.35E-03 | -1.99 | Sepsis_D0_non-survivor down vs Sepsis_D0_survivor |
| MTMR10 | 4.49E-03 | -1.99 | Sepsis_D0_non-survivor down vs Sepsis_D0_survivor |
| SMAD5 | 5.86E-03 | -1.99 | Sepsis_D0_non-survivor down vs Sepsis_D0_survivor |
| ARHGAP18 | 4.17E-03 | -1.99 | Sepsis_D0_non-survivor down vs Sepsis_D0_survivor |
| YOD1 | 3.42E-03 | -1.98 | Sepsis_D0_non-survivor down vs Sepsis_D0_survivor |
| MRS2L | 2.51E-04 | -1.97 | Sepsis_D0_non-survivor down vs Sepsis_D0_survivor |
| NDUFB5 | 3.16E-03 | -1.96 | Sepsis_D0_non-survivor down vs Sepsis_D0_survivor |
| CDCA7 | 5.02E-03 | -1.96 | Sepsis_D0_non-survivor down vs Sepsis_D0_survivor |
| PIN4 | 5.36E-03 | -1.95 | Sepsis_D0_non-survivor down vs Sepsis_D0_survivor |
| ITSN1 | 5.38E-04 | -1.95 | Sepsis_D0_non-survivor down vs Sepsis_D0_survivor |
| SRD5A1 | 3.20E-03 | -1.95 | Sepsis_D0_non-survivor down vs Sepsis_D0_survivor |
| MPP7 | 4.69E-03 | -1.95 | Sepsis_D0_non-survivor down vs Sepsis_D0_survivor |
| FAM121A | 4.30E-03 | -1.94 | Sepsis_D0_non-survivor down vs Sepsis_D0_survivor |
| NAPE-PLD | 4.63E-03 | -1.94 | Sepsis_D0_non-survivor down vs Sepsis_D0_survivor |
| LIMA1 | 2.51E-04 | -1.93 | Sepsis_D0_non-survivor down vs Sepsis_D0_survivor |
| SPFH2 | 1.43E-04 | -1.93 | Sepsis_D0_non-survivor down vs Sepsis_D0_survivor |
| ZDHHC2 | 5.78E-04 | -1.93 | Sepsis_D0_non-survivor down vs Sepsis_D0_survivor |
| ZNF354A | 2.66E-03 | -1.93 | Sepsis_D0_non-survivor down vs Sepsis_D0_survivor |
| LOH11CR2A | 8.98E-04 | -1.92 | Sepsis_D0_non-survivor down vs Sepsis_D0_survivor |
| B3GNT1 | 1.46E-03 | -1.92 | Sepsis_D0_non-survivor down vs Sepsis_D0_survivor |
| GCAT | 7.20E-05 | -1.92 | Sepsis_D0_non-survivor down vs Sepsis_D0_survivor |
| SNAPC5 | 1.16E-03 | -1.92 | Sepsis_D0_non-survivor down vs Sepsis_D0_survivor |
| IRAK4 | 4.48E-03 | -1.92 | Sepsis_D0_non-survivor down vs Sepsis_D0_survivor |
| FAM98A | 1.11E-03 | -1.91 | Sepsis_D0_non-survivor down vs Sepsis_D0_survivor |
| PHF6 | 5.02E-03 | -1.91 | Sepsis_D0_non-survivor down vs Sepsis_D0_survivor |
| CALD1 | 4.96E-03 | -1.91 | Sepsis_D0_non-survivor down vs Sepsis_D0_survivor |
| NUCB2 | 9.12E-04 | -1.91 | Sepsis_D0_non-survivor down vs Sepsis_D0_survivor |
| RWDD4A | 2.73E-04 | -1.91 | Sepsis_D0_non-survivor down vs Sepsis_D0_survivor |
| SNAPC1 | 1.76E-03 | -1.90 | Sepsis_D0_non-survivor down vs Sepsis_D0_survivor |
| LY75 | 5.50E-04 | -1.90 | Sepsis_D0_non-survivor down vs Sepsis_D0_survivor |
| MYSM1 | 2.02E-04 | -1.90 | Sepsis_D0_non-survivor down vs Sepsis_D0_survivor |
| MGST1 | 1.37E-05 | -1.89 | Sepsis_D0_non-survivor down vs Sepsis_D0_survivor |
| DDR1 | 9.62E-04 | -1.89 | Sepsis_D0_non-survivor down vs Sepsis_D0_survivor |
| TXNDC10 | 1.48E-03 | -1.89 | Sepsis_D0_non-survivor down vs Sepsis_D0_survivor |
| SPFH1 | 5.43E-03 | -1.89 | Sepsis_D0_non-survivor down vs Sepsis_D0_survivor |
| PPCS | 5.33E-04 | -1.88 | Sepsis_D0_non-survivor down vs Sepsis_D0_survivor |
| RFXAP | 9.33E-04 | -1.88 | Sepsis_D0_non-survivor down vs Sepsis_D0_survivor |
| TMLHE | 4.46E-03 | -1.88 | Sepsis_D0_non-survivor down vs Sepsis_D0_survivor |
| PDIA5 | 8.30E-07 | -1.88 | Sepsis_D0_non-survivor down vs Sepsis_D0_survivor |
| PDE6H | 4.79E-03 | -1.88 | Sepsis_D0_non-survivor down vs Sepsis_D0_survivor |
| NEK4 | 1.54E-03 | -1.87 | Sepsis_D0_non-survivor down vs Sepsis_D0_survivor |
| PPA1 | 5.09E-04 | -1.87 | Sepsis_D0_non-survivor down vs Sepsis_D0_survivor |
| NUDT3 | 1.60E-03 | -1.87 | Sepsis_D0_non-survivor down vs Sepsis_D0_survivor |
| GCNT3 | 3.61E-03 | -1.87 | Sepsis_D0_non-survivor down vs Sepsis_D0_survivor |
| PAPD5 | 5.42E-04 | -1.87 | Sepsis_D0_non-survivor down vs Sepsis_D0_survivor |
| PCYOX1 | 1.17E-03 | -1.87 | Sepsis_D0_non-survivor down vs Sepsis_D0_survivor |
| CCDC109A | 1.45E-03 | -1.87 | Sepsis_D0_non-survivor down vs Sepsis_D0_survivor |
| RPL39L | 4.52E-03 | -1.86 | Sepsis_D0_non-survivor down vs Sepsis_D0_survivor |
| HMG4L | 4.00E-03 | -1.86 | Sepsis_D0_non-survivor down vs Sepsis_D0_survivor |
| HLX1 | 3.94E-03 | -1.86 | Sepsis_D0_non-survivor down vs Sepsis_D0_survivor |
| RDX | 2.75E-03 | -1.86 | Sepsis_D0_non-survivor down vs Sepsis_D0_survivor |
| NNT | 4.47E-03 | -1.86 | Sepsis_D0_non-survivor down vs Sepsis_D0_survivor |
| SLC37A3 | 1.05E-04 | -1.86 | Sepsis_D0_non-survivor down vs Sepsis_D0_survivor |
| BRF2 | 3.90E-03 | -1.86 | Sepsis_D0_non-survivor down vs Sepsis_D0_survivor |
| ABHD12 | 4.85E-03 | -1.86 | Sepsis_D0_non-survivor down vs Sepsis_D0_survivor |
| NMNAT1 | 5.26E-03 | -1.86 | Sepsis_D0_non-survivor down vs Sepsis_D0_survivor |
| SH3BP4 | 1.94E-03 | -1.86 | Sepsis_D0_non-survivor down vs Sepsis_D0_survivor |
| MOBKL1A | 2.98E-03 | -1.86 | Sepsis_D0_non-survivor down vs Sepsis_D0_survivor |
| HKR3 | 2.42E-04 | -1.85 | Sepsis_D0_non-survivor down vs Sepsis_D0_survivor |
| PECR | 1.83E-04 | -1.85 | Sepsis_D0_non-survivor down vs Sepsis_D0_survivor |
| ABHD3 | 4.49E-03 | -1.85 | Sepsis_D0_non-survivor down vs Sepsis_D0_survivor |
| MRPL42P5 | 4.91E-03 | -1.85 | Sepsis_D0_non-survivor down vs Sepsis_D0_survivor |
| TOPORS | 2.38E-03 | -1.85 | Sepsis_D0_non-survivor down vs Sepsis_D0_survivor |
| SPOCK1 | 2.48E-04 | -1.84 | Sepsis_D0_non-survivor down vs Sepsis_D0_survivor |
| METTL7B | 5.26E-03 | -1.84 | Sepsis_D0_non-survivor down vs Sepsis_D0_survivor |
| ZNFN1A5 | 3.23E-03 | -1.83 | Sepsis_D0_non-survivor down vs Sepsis_D0_survivor |
| SAMHD1 | 7.88E-04 | -1.83 | Sepsis_D0_non-survivor down vs Sepsis_D0_survivor |
| CENPN | 1.61E-03 | -1.83 | Sepsis_D0_non-survivor down vs Sepsis_D0_survivor |
| A4GNT | 2.66E-03 | -1.83 | Sepsis_D0_non-survivor down vs Sepsis_D0_survivor |
| ALDH9A1 | 3.46E-04 | -1.82 | Sepsis_D0_non-survivor down vs Sepsis_D0_survivor |
| CENPH | 1.36E-03 | -1.82 | Sepsis_D0_non-survivor down vs Sepsis_D0_survivor |
| STK32C | 2.45E-03 | -1.82 | Sepsis_D0_non-survivor down vs Sepsis_D0_survivor |
| LYSMD3 | 4.45E-04 | -1.82 | Sepsis_D0_non-survivor down vs Sepsis_D0_survivor |
| ZNF33A | 9.75E-05 | -1.82 | Sepsis_D0_non-survivor down vs Sepsis_D0_survivor |
| ARL6IP6 | 1.62E-03 | -1.82 | Sepsis_D0_non-survivor down vs Sepsis_D0_survivor |
| CNIH | 3.02E-04 | -1.81 | Sepsis_D0_non-survivor down vs Sepsis_D0_survivor |
| PTTG2 | 5.35E-03 | -1.81 | Sepsis_D0_non-survivor down vs Sepsis_D0_survivor |
| TRIM38 | 2.40E-04 | -1.81 | Sepsis_D0_non-survivor down vs Sepsis_D0_survivor |
| IRF4 | 4.36E-03 | -1.81 | Sepsis_D0_non-survivor down vs Sepsis_D0_survivor |
| RFK | 7.20E-03 | -1.80 | Sepsis_D0_non-survivor down vs Sepsis_D0_survivor |
| DMRT1 | 7.47E-03 | -1.80 | Sepsis_D0_non-survivor down vs Sepsis_D0_survivor |
| ALG11 | 2.91E-03 | -1.80 | Sepsis_D0_non-survivor down vs Sepsis_D0_survivor |
| CRKL | 1.22E-03 | -1.80 | Sepsis_D0_non-survivor down vs Sepsis_D0_survivor |
| KENAE | 7.97E-04 | -1.80 | Sepsis_D0_non-survivor down vs Sepsis_D0_survivor |
| TMEM107 | 6.63E-03 | -1.80 | Sepsis_D0_non-survivor down vs Sepsis_D0_survivor |
| ARL1 | 1.29E-03 | -1.80 | Sepsis_D0_non-survivor down vs Sepsis_D0_survivor |
| SLC1A5 | 5.10E-03 | -1.79 | Sepsis_D0_non-survivor down vs Sepsis_D0_survivor |
| FBXL4 | 7.27E-03 | -1.79 | Sepsis_D0_non-survivor down vs Sepsis_D0_survivor |
| TMTC4 | 3.76E-04 | -1.79 | Sepsis_D0_non-survivor down vs Sepsis_D0_survivor |
| PCNA | 2.56E-03 | -1.79 | Sepsis_D0_non-survivor down vs Sepsis_D0_survivor |
| TGDS | 3.22E-03 | -1.79 | Sepsis_D0_non-survivor down vs Sepsis_D0_survivor |
| PRKAG1 | 2.02E-03 | -1.79 | Sepsis_D0_non-survivor down vs Sepsis_D0_survivor |
| TOR1B | 5.92E-03 | -1.79 | Sepsis_D0_non-survivor down vs Sepsis_D0_survivor |
| RAB11FIP2 | 2.50E-03 | -1.79 | Sepsis_D0_non-survivor down vs Sepsis_D0_survivor |
| LIN7A | 1.59E-03 | -1.78 | Sepsis_D0_non-survivor down vs Sepsis_D0_survivor |
| HPCA | 2.45E-03 | -1.78 | Sepsis_D0_non-survivor down vs Sepsis_D0_survivor |
| MORN2 | 5.56E-03 | -1.78 | Sepsis_D0_non-survivor down vs Sepsis_D0_survivor |
| AGPAT2 | 2.09E-03 | -1.78 | Sepsis_D0_non-survivor down vs Sepsis_D0_survivor |
| GOLGA5 | 1.58E-03 | -1.78 | Sepsis_D0_non-survivor down vs Sepsis_D0_survivor |
| DC2 | 5.80E-04 | -1.78 | Sepsis_D0_non-survivor down vs Sepsis_D0_survivor |
| UPF3B | 2.64E-03 | -1.78 | Sepsis_D0_non-survivor down vs Sepsis_D0_survivor |
| RIT1 | 2.50E-04 | -1.78 | Sepsis_D0_non-survivor down vs Sepsis_D0_survivor |
| EIF1AY | 5.35E-03 | -1.78 | Sepsis_D0_non-survivor down vs Sepsis_D0_survivor |
| RRM2B | 6.67E-03 | -1.78 | Sepsis_D0_non-survivor down vs Sepsis_D0_survivor |
| DUS2L | 7.30E-03 | -1.77 | Sepsis_D0_non-survivor down vs Sepsis_D0_survivor |
| MBIP | 7.72E-04 | -1.77 | Sepsis_D0_non-survivor down vs Sepsis_D0_survivor |
| DHRSX | 1.40E-03 | -1.77 | Sepsis_D0_non-survivor down vs Sepsis_D0_survivor |
| CPD | 3.80E-04 | -1.77 | Sepsis_D0_non-survivor down vs Sepsis_D0_survivor |
| CMAS | 1.92E-04 | -1.77 | Sepsis_D0_non-survivor down vs Sepsis_D0_survivor |
| SGTB | 6.67E-03 | -1.77 | Sepsis_D0_non-survivor down vs Sepsis_D0_survivor |
| TRIM23 | 2.78E-03 | -1.77 | Sepsis_D0_non-survivor down vs Sepsis_D0_survivor |
| POLR3C | 3.71E-04 | -1.77 | Sepsis_D0_non-survivor down vs Sepsis_D0_survivor |
| SLC44A1 | 3.55E-03 | -1.77 | Sepsis_D0_non-survivor down vs Sepsis_D0_survivor |
| GORASP1 | 5.20E-03 | -1.77 | Sepsis_D0_non-survivor down vs Sepsis_D0_survivor |
| ATP6V1E2 | 1.40E-03 | -1.76 | Sepsis_D0_non-survivor down vs Sepsis_D0_survivor |
| GCLM | 5.21E-03 | -1.76 | Sepsis_D0_non-survivor down vs Sepsis_D0_survivor |
| CCPG1 | 7.56E-04 | -1.76 | Sepsis_D0_non-survivor down vs Sepsis_D0_survivor |
| TMEM5 | 5.74E-04 | -1.76 | Sepsis_D0_non-survivor down vs Sepsis_D0_survivor |
| FAM44A | 1.86E-04 | -1.76 | Sepsis_D0_non-survivor down vs Sepsis_D0_survivor |
| PLSCR4 | 6.34E-03 | -1.75 | Sepsis_D0_non-survivor down vs Sepsis_D0_survivor |
| SDCCAG8 | 3.20E-03 | -1.75 | Sepsis_D0_non-survivor down vs Sepsis_D0_survivor |
| CMPK | 1.39E-03 | -1.75 | Sepsis_D0_non-survivor down vs Sepsis_D0_survivor |
| PPM1D | 3.71E-03 | -1.75 | Sepsis_D0_non-survivor down vs Sepsis_D0_survivor |
| DUSP11 | 7.63E-03 | -1.75 | Sepsis_D0_non-survivor down vs Sepsis_D0_survivor |
| ATP5C1 | 9.58E-04 | -1.75 | Sepsis_D0_non-survivor down vs Sepsis_D0_survivor |
| ME3 | 1.55E-03 | -1.75 | Sepsis_D0_non-survivor down vs Sepsis_D0_survivor |
| LRP8 | 1.80E-03 | -1.75 | Sepsis_D0_non-survivor down vs Sepsis_D0_survivor |
| POLR3B | 5.83E-03 | -1.75 | Sepsis_D0_non-survivor down vs Sepsis_D0_survivor |
| TSPYL5 | 4.82E-03 | -1.75 | Sepsis_D0_non-survivor down vs Sepsis_D0_survivor |
| SFT2D2 | 1.26E-03 | -1.74 | Sepsis_D0_non-survivor down vs Sepsis_D0_survivor |
| TSPAN2 | 8.94E-04 | -1.74 | Sepsis_D0_non-survivor down vs Sepsis_D0_survivor |
| ESD | 1.43E-04 | -1.74 | Sepsis_D0_non-survivor down vs Sepsis_D0_survivor |
| PTPLB | 2.94E-03 | -1.74 | Sepsis_D0_non-survivor down vs Sepsis_D0_survivor |
| SPTY2D1 | 4.17E-03 | -1.74 | Sepsis_D0_non-survivor down vs Sepsis_D0_survivor |
| WDSUB1 | 1.69E-03 | -1.74 | Sepsis_D0_non-survivor down vs Sepsis_D0_survivor |
| SEMA3C | 5.08E-03 | -1.74 | Sepsis_D0_non-survivor down vs Sepsis_D0_survivor |
| NFS1 | 1.40E-03 | -1.74 | Sepsis_D0_non-survivor down vs Sepsis_D0_survivor |
| RAD50 | 4.72E-04 | -1.73 | Sepsis_D0_non-survivor down vs Sepsis_D0_survivor |
| ZNF23 | 5.63E-03 | -1.73 | Sepsis_D0_non-survivor down vs Sepsis_D0_survivor |
| CCDC53 | 4.67E-04 | -1.73 | Sepsis_D0_non-survivor down vs Sepsis_D0_survivor |
| FAM122C | 5.32E-03 | -1.73 | Sepsis_D0_non-survivor down vs Sepsis_D0_survivor |
| CRIPT | 9.28E-04 | -1.73 | Sepsis_D0_non-survivor down vs Sepsis_D0_survivor |
| PPA2 | 4.91E-03 | -1.73 | Sepsis_D0_non-survivor down vs Sepsis_D0_survivor |
| TTRAP | 8.18E-04 | -1.73 | Sepsis_D0_non-survivor down vs Sepsis_D0_survivor |
| ACSL3 | 5.17E-03 | -1.73 | Sepsis_D0_non-survivor down vs Sepsis_D0_survivor |
| DNAPTP6 | 4.04E-03 | -1.72 | Sepsis_D0_non-survivor down vs Sepsis_D0_survivor |
| TMEM126B | 1.69E-04 | -1.72 | Sepsis_D0_non-survivor down vs Sepsis_D0_survivor |
| ADPRH | 1.34E-03 | -1.72 | Sepsis_D0_non-survivor down vs Sepsis_D0_survivor |
| ZNF673 | 4.78E-03 | -1.72 | Sepsis_D0_non-survivor down vs Sepsis_D0_survivor |
| ICK | 2.13E-03 | -1.72 | Sepsis_D0_non-survivor down vs Sepsis_D0_survivor |
| S100Z | 1.81E-03 | -1.72 | Sepsis_D0_non-survivor down vs Sepsis_D0_survivor |
| IER3IP1 | 2.79E-03 | -1.72 | Sepsis_D0_non-survivor down vs Sepsis_D0_survivor |
| DGCR2 | 5.75E-03 | -1.72 | Sepsis_D0_non-survivor down vs Sepsis_D0_survivor |
| PHF5A | 3.60E-03 | -1.72 | Sepsis_D0_non-survivor down vs Sepsis_D0_survivor |
| DCUN1D5 | 3.11E-04 | -1.72 | Sepsis_D0_non-survivor down vs Sepsis_D0_survivor |
| PODN | 3.45E-03 | -1.72 | Sepsis_D0_non-survivor down vs Sepsis_D0_survivor |
| ATXN3 | 2.66E-03 | -1.72 | Sepsis_D0_non-survivor down vs Sepsis_D0_survivor |
| DLEU8 | 1.31E-03 | -1.72 | Sepsis_D0_non-survivor down vs Sepsis_D0_survivor |
| GARS | 2.16E-03 | -1.71 | Sepsis_D0_non-survivor down vs Sepsis_D0_survivor |
| FAM82A | 2.73E-03 | -1.71 | Sepsis_D0_non-survivor down vs Sepsis_D0_survivor |
| RHBDD1 | 6.68E-03 | -1.71 | Sepsis_D0_non-survivor down vs Sepsis_D0_survivor |
| PIGF | 8.05E-04 | -1.71 | Sepsis_D0_non-survivor down vs Sepsis_D0_survivor |
| GDAP2 | 4.32E-03 | -1.71 | Sepsis_D0_non-survivor down vs Sepsis_D0_survivor |
| COX18 | 2.84E-03 | -1.71 | Sepsis_D0_non-survivor down vs Sepsis_D0_survivor |
| LMBR1 | 2.20E-03 | -1.71 | Sepsis_D0_non-survivor down vs Sepsis_D0_survivor |
| SLC27A4 | 6.35E-04 | -1.70 | Sepsis_D0_non-survivor down vs Sepsis_D0_survivor |
| LTB4DH | 3.12E-03 | -1.70 | Sepsis_D0_non-survivor down vs Sepsis_D0_survivor |
| CD109 | 6.26E-03 | -1.70 | Sepsis_D0_non-survivor down vs Sepsis_D0_survivor |
| TBC1D24 | 2.37E-03 | -1.70 | Sepsis_D0_non-survivor down vs Sepsis_D0_survivor |
| TMEM65 | 2.12E-03 | -1.70 | Sepsis_D0_non-survivor down vs Sepsis_D0_survivor |
| CEP63 | 1.15E-03 | -1.70 | Sepsis_D0_non-survivor down vs Sepsis_D0_survivor |
| FGFR1OP2 | 2.40E-03 | -1.70 | Sepsis_D0_non-survivor down vs Sepsis_D0_survivor |
| NUDT4 | 3.96E-03 | -1.70 | Sepsis_D0_non-survivor down vs Sepsis_D0_survivor |
| ZNF415 | 9.36E-04 | -1.70 | Sepsis_D0_non-survivor down vs Sepsis_D0_survivor |
| GCA | 6.10E-03 | -1.69 | Sepsis_D0_non-survivor down vs Sepsis_D0_survivor |
| TTLL1 | 9.29E-04 | -1.69 | Sepsis_D0_non-survivor down vs Sepsis_D0_survivor |
| CGRRF1 | 3.96E-03 | -1.69 | Sepsis_D0_non-survivor down vs Sepsis_D0_survivor |
| HTATIP2 | 1.69E-03 | -1.69 | Sepsis_D0_non-survivor down vs Sepsis_D0_survivor |
| ITGAE | 2.47E-03 | -1.69 | Sepsis_D0_non-survivor down vs Sepsis_D0_survivor |
| DONSON | 3.36E-03 | -1.69 | Sepsis_D0_non-survivor down vs Sepsis_D0_survivor |
| PSMC6 | 7.34E-03 | -1.69 | Sepsis_D0_non-survivor down vs Sepsis_D0_survivor |
| SLC39A6 | 1.19E-03 | -1.68 | Sepsis_D0_non-survivor down vs Sepsis_D0_survivor |
| TMEM2 | 2.18E-03 | -1.68 | Sepsis_D0_non-survivor down vs Sepsis_D0_survivor |
| MBNL2 | 5.44E-03 | -1.68 | Sepsis_D0_non-survivor down vs Sepsis_D0_survivor |
| MTMR12 | 9.80E-06 | -1.68 | Sepsis_D0_non-survivor down vs Sepsis_D0_survivor |
| CYTL1 | 4.09E-04 | -1.68 | Sepsis_D0_non-survivor down vs Sepsis_D0_survivor |
| GABPA | 4.13E-03 | -1.68 | Sepsis_D0_non-survivor down vs Sepsis_D0_survivor |
| PIGV | 7.59E-03 | -1.68 | Sepsis_D0_non-survivor down vs Sepsis_D0_survivor |
| PGBD4 | 1.81E-05 | -1.68 | Sepsis_D0_non-survivor down vs Sepsis_D0_survivor |
| ZMYM5 | 3.67E-04 | -1.68 | Sepsis_D0_non-survivor down vs Sepsis_D0_survivor |
| GYG1 | 3.61E-03 | -1.68 | Sepsis_D0_non-survivor down vs Sepsis_D0_survivor |
| EAF2 | 5.22E-03 | -1.68 | Sepsis_D0_non-survivor down vs Sepsis_D0_survivor |
| GPR155 | 1.86E-03 | -1.67 | Sepsis_D0_non-survivor down vs Sepsis_D0_survivor |
| LPAAT-THETA | 4.64E-03 | -1.67 | Sepsis_D0_non-survivor down vs Sepsis_D0_survivor |
| ZNF32 | 5.14E-03 | -1.67 | Sepsis_D0_non-survivor down vs Sepsis_D0_survivor |
| CCDC28A | 3.45E-03 | -1.67 | Sepsis_D0_non-survivor down vs Sepsis_D0_survivor |
| SENP6 | 4.32E-03 | -1.67 | Sepsis_D0_non-survivor down vs Sepsis_D0_survivor |
| CAB39L | 1.33E-03 | -1.67 | Sepsis_D0_non-survivor down vs Sepsis_D0_survivor |
| ZNRF2 | 4.07E-03 | -1.67 | Sepsis_D0_non-survivor down vs Sepsis_D0_survivor |
| LRP5L | 6.15E-03 | -1.67 | Sepsis_D0_non-survivor down vs Sepsis_D0_survivor |
| TMOD3 | 4.96E-03 | -1.67 | Sepsis_D0_non-survivor down vs Sepsis_D0_survivor |
| SORT1 | 4.27E-03 | -1.67 | Sepsis_D0_non-survivor down vs Sepsis_D0_survivor |
| AMOT | 1.54E-03 | -1.67 | Sepsis_D0_non-survivor down vs Sepsis_D0_survivor |
| WDR51B | 5.49E-03 | -1.67 | Sepsis_D0_non-survivor down vs Sepsis_D0_survivor |
| SMARCAL1 | 2.10E-03 | -1.67 | Sepsis_D0_non-survivor down vs Sepsis_D0_survivor |
| TMEM106B | 3.67E-03 | -1.67 | Sepsis_D0_non-survivor down vs Sepsis_D0_survivor |
| COMMD10 | 1.73E-03 | -1.67 | Sepsis_D0_non-survivor down vs Sepsis_D0_survivor |
| PAK2 | 3.71E-03 | -1.67 | Sepsis_D0_non-survivor down vs Sepsis_D0_survivor |
| MNAB | 2.82E-03 | -1.67 | Sepsis_D0_non-survivor down vs Sepsis_D0_survivor |
| PLCL2 | 3.02E-03 | -1.67 | Sepsis_D0_non-survivor down vs Sepsis_D0_survivor |
| HNRPLL | 8.55E-04 | -1.66 | Sepsis_D0_non-survivor down vs Sepsis_D0_survivor |
| SLMAP | 5.13E-04 | -1.66 | Sepsis_D0_non-survivor down vs Sepsis_D0_survivor |
| UBE2J2 | 1.70E-03 | -1.66 | Sepsis_D0_non-survivor down vs Sepsis_D0_survivor |
| ATP6V1C1 | 6.33E-03 | -1.66 | Sepsis_D0_non-survivor down vs Sepsis_D0_survivor |
| YIPF6 | 3.12E-03 | -1.66 | Sepsis_D0_non-survivor down vs Sepsis_D0_survivor |
| YAF2 | 6.75E-04 | -1.66 | Sepsis_D0_non-survivor down vs Sepsis_D0_survivor |
| TCEB3 | 2.06E-03 | -1.66 | Sepsis_D0_non-survivor down vs Sepsis_D0_survivor |
| GPR65 | 5.31E-03 | -1.65 | Sepsis_D0_non-survivor down vs Sepsis_D0_survivor |
| FTS | 2.71E-03 | -1.65 | Sepsis_D0_non-survivor down vs Sepsis_D0_survivor |
| UBE2B | 4.19E-05 | -1.65 | Sepsis_D0_non-survivor down vs Sepsis_D0_survivor |
| CUL2 | 4.14E-03 | -1.65 | Sepsis_D0_non-survivor down vs Sepsis_D0_survivor |
| ABHD5 | 3.73E-03 | -1.65 | Sepsis_D0_non-survivor down vs Sepsis_D0_survivor |
| RB1 | 5.38E-03 | -1.65 | Sepsis_D0_non-survivor down vs Sepsis_D0_survivor |
| SMEK2 | 1.87E-03 | -1.65 | Sepsis_D0_non-survivor down vs Sepsis_D0_survivor |
| PDCD10 | 3.02E-03 | -1.65 | Sepsis_D0_non-survivor down vs Sepsis_D0_survivor |
| RABGAP1L | 6.91E-03 | -1.65 | Sepsis_D0_non-survivor down vs Sepsis_D0_survivor |
| SPATA5L1 | 7.40E-04 | -1.65 | Sepsis_D0_non-survivor down vs Sepsis_D0_survivor |
| INTS3 | 9.87E-04 | -1.65 | Sepsis_D0_non-survivor down vs Sepsis_D0_survivor |
| TBC1D23 | 2.74E-03 | -1.65 | Sepsis_D0_non-survivor down vs Sepsis_D0_survivor |
| STAU2 | 1.64E-03 | -1.65 | Sepsis_D0_non-survivor down vs Sepsis_D0_survivor |
| TNPO3 | 1.78E-03 | -1.65 | Sepsis_D0_non-survivor down vs Sepsis_D0_survivor |
| SAMD8 | 5.50E-03 | -1.65 | Sepsis_D0_non-survivor down vs Sepsis_D0_survivor |
| GABARAPL2 | 2.06E-03 | -1.65 | Sepsis_D0_non-survivor down vs Sepsis_D0_survivor |
| COX10 | 2.16E-03 | -1.65 | Sepsis_D0_non-survivor down vs Sepsis_D0_survivor |
| RNF6 | 3.02E-03 | -1.65 | Sepsis_D0_non-survivor down vs Sepsis_D0_survivor |
| CS | 1.14E-03 | -1.65 | Sepsis_D0_non-survivor down vs Sepsis_D0_survivor |
| INTS8 | 3.28E-03 | -1.65 | Sepsis_D0_non-survivor down vs Sepsis_D0_survivor |
| FAM82B | 4.27E-03 | -1.64 | Sepsis_D0_non-survivor down vs Sepsis_D0_survivor |
| ERGIC2 | 8.61E-04 | -1.64 | Sepsis_D0_non-survivor down vs Sepsis_D0_survivor |
| KLHL7 | 6.89E-04 | -1.64 | Sepsis_D0_non-survivor down vs Sepsis_D0_survivor |
| PREI3 | 8.74E-05 | -1.64 | Sepsis_D0_non-survivor down vs Sepsis_D0_survivor |
| SSR3 | 5.60E-03 | -1.64 | Sepsis_D0_non-survivor down vs Sepsis_D0_survivor |
| PYGL | 5.51E-04 | -1.64 | Sepsis_D0_non-survivor down vs Sepsis_D0_survivor |
| FAM105A | 3.24E-03 | -1.64 | Sepsis_D0_non-survivor down vs Sepsis_D0_survivor |
| PSMD14 | 1.05E-03 | -1.64 | Sepsis_D0_non-survivor down vs Sepsis_D0_survivor |
| ATPBD4 | 3.74E-04 | -1.64 | Sepsis_D0_non-survivor down vs Sepsis_D0_survivor |
| ZBTB39 | 6.88E-03 | -1.64 | Sepsis_D0_non-survivor down vs Sepsis_D0_survivor |
| IQGAP2 | 4.00E-03 | -1.64 | Sepsis_D0_non-survivor down vs Sepsis_D0_survivor |
| MCFP | 3.27E-04 | -1.64 | Sepsis_D0_non-survivor down vs Sepsis_D0_survivor |
| PRR8 | 8.27E-04 | -1.64 | Sepsis_D0_non-survivor down vs Sepsis_D0_survivor |
| SRP19 | 2.92E-03 | -1.64 | Sepsis_D0_non-survivor down vs Sepsis_D0_survivor |
| CSF2RA | 1.44E-03 | -1.64 | Sepsis_D0_non-survivor down vs Sepsis_D0_survivor |
| CETN2 | 1.24E-03 | -1.64 | Sepsis_D0_non-survivor down vs Sepsis_D0_survivor |
| MS4A3 | 7.72E-03 | -1.63 | Sepsis_D0_non-survivor down vs Sepsis_D0_survivor |
| MOCS2 | 3.02E-03 | -1.63 | Sepsis_D0_non-survivor down vs Sepsis_D0_survivor |
| HPRT1 | 1.24E-04 | -1.63 | Sepsis_D0_non-survivor down vs Sepsis_D0_survivor |
| PIGH | 2.84E-04 | -1.63 | Sepsis_D0_non-survivor down vs Sepsis_D0_survivor |
| SRP9 | 1.87E-03 | -1.63 | Sepsis_D0_non-survivor down vs Sepsis_D0_survivor |
| TM2D1 | 2.60E-03 | -1.63 | Sepsis_D0_non-survivor down vs Sepsis_D0_survivor |
| ZNF318 | 6.72E-03 | -1.63 | Sepsis_D0_non-survivor down vs Sepsis_D0_survivor |
| ABT1 | 1.25E-03 | -1.63 | Sepsis_D0_non-survivor down vs Sepsis_D0_survivor |
| AMMECR1 | 1.67E-03 | -1.63 | Sepsis_D0_non-survivor down vs Sepsis_D0_survivor |
| NIT2 | 2.78E-04 | -1.63 | Sepsis_D0_non-survivor down vs Sepsis_D0_survivor |
| EIF2S2 | 2.69E-03 | -1.62 | Sepsis_D0_non-survivor down vs Sepsis_D0_survivor |
| RAB27A | 3.01E-04 | -1.62 | Sepsis_D0_non-survivor down vs Sepsis_D0_survivor |
| AMZ2 | 2.21E-03 | -1.62 | Sepsis_D0_non-survivor down vs Sepsis_D0_survivor |
| DOPEY2 | 7.47E-03 | -1.62 | Sepsis_D0_non-survivor down vs Sepsis_D0_survivor |
| ATP6V1E1 | 5.20E-04 | -1.62 | Sepsis_D0_non-survivor down vs Sepsis_D0_survivor |
| ZNF34 | 6.96E-03 | -1.62 | Sepsis_D0_non-survivor down vs Sepsis_D0_survivor |
| MAP4K5 | 7.43E-03 | -1.62 | Sepsis_D0_non-survivor down vs Sepsis_D0_survivor |
| AASDH | 7.43E-03 | -1.62 | Sepsis_D0_non-survivor down vs Sepsis_D0_survivor |
| SOAT1 | 6.40E-03 | -1.62 | Sepsis_D0_non-survivor down vs Sepsis_D0_survivor |
| AGL | 2.69E-03 | -1.62 | Sepsis_D0_non-survivor down vs Sepsis_D0_survivor |
| ATP2B4 | 2.20E-03 | -1.62 | Sepsis_D0_non-survivor down vs Sepsis_D0_survivor |
| ZNF124 | 3.06E-03 | -1.62 | Sepsis_D0_non-survivor down vs Sepsis_D0_survivor |
| PLXNC1 | 5.89E-03 | -1.62 | Sepsis_D0_non-survivor down vs Sepsis_D0_survivor |
| CLIC6 | 1.91E-04 | -1.62 | Sepsis_D0_non-survivor down vs Sepsis_D0_survivor |
| HSPC268 | 2.43E-03 | -1.62 | Sepsis_D0_non-survivor down vs Sepsis_D0_survivor |
| XYLT2 | 4.27E-03 | -1.61 | Sepsis_D0_non-survivor down vs Sepsis_D0_survivor |
| CCNDBP1 | 1.02E-03 | -1.61 | Sepsis_D0_non-survivor down vs Sepsis_D0_survivor |
| IFNW1 | 7.13E-03 | -1.61 | Sepsis_D0_non-survivor down vs Sepsis_D0_survivor |
| RIPK3 | 2.17E-03 | -1.61 | Sepsis_D0_non-survivor down vs Sepsis_D0_survivor |
| NSUN6 | 5.49E-03 | -1.61 | Sepsis_D0_non-survivor down vs Sepsis_D0_survivor |
| ASB7 | 1.47E-03 | -1.61 | Sepsis_D0_non-survivor down vs Sepsis_D0_survivor |
| DNAJC19 | 1.78E-03 | -1.61 | Sepsis_D0_non-survivor down vs Sepsis_D0_survivor |
| SLC22A4 | 1.18E-03 | -1.61 | Sepsis_D0_non-survivor down vs Sepsis_D0_survivor |
| ACBD5 | 1.43E-03 | -1.61 | Sepsis_D0_non-survivor down vs Sepsis_D0_survivor |
| THRAP6 | 1.37E-04 | -1.61 | Sepsis_D0_non-survivor down vs Sepsis_D0_survivor |
| HBLD2 | 3.65E-03 | -1.60 | Sepsis_D0_non-survivor down vs Sepsis_D0_survivor |
| SLC26A2 | 5.67E-03 | -1.60 | Sepsis_D0_non-survivor down vs Sepsis_D0_survivor |
| ZNF277 | 5.68E-03 | -1.60 | Sepsis_D0_non-survivor down vs Sepsis_D0_survivor |
| PXK | 9.14E-04 | -1.60 | Sepsis_D0_non-survivor down vs Sepsis_D0_survivor |
| SLC30A5 | 6.08E-03 | -1.60 | Sepsis_D0_non-survivor down vs Sepsis_D0_survivor |
| TTC1 | 2.88E-03 | -1.60 | Sepsis_D0_non-survivor down vs Sepsis_D0_survivor |
| ACTR8 | 2.06E-03 | -1.60 | Sepsis_D0_non-survivor down vs Sepsis_D0_survivor |
| SPAG16 | 3.40E-03 | -1.60 | Sepsis_D0_non-survivor down vs Sepsis_D0_survivor |
| RWDD2 | 3.30E-03 | -1.60 | Sepsis_D0_non-survivor down vs Sepsis_D0_survivor |
| MSL3L1 | 1.00E-03 | -1.60 | Sepsis_D0_non-survivor down vs Sepsis_D0_survivor |
| BLOC1S2 | 1.16E-03 | -1.60 | Sepsis_D0_non-survivor down vs Sepsis_D0_survivor |
| SMARCA3 | 4.66E-03 | -1.60 | Sepsis_D0_non-survivor down vs Sepsis_D0_survivor |
| IMPACT | 7.07E-03 | -1.60 | Sepsis_D0_non-survivor down vs Sepsis_D0_survivor |
| RNF8 | 5.20E-03 | -1.60 | Sepsis_D0_non-survivor down vs Sepsis_D0_survivor |
| MRPL36 | 4.32E-03 | -1.60 | Sepsis_D0_non-survivor down vs Sepsis_D0_survivor |
| PIGO | 1.49E-05 | -1.60 | Sepsis_D0_non-survivor down vs Sepsis_D0_survivor |
| SS18L1 | 1.15E-03 | -1.60 | Sepsis_D0_non-survivor down vs Sepsis_D0_survivor |
| MSH3 | 2.69E-03 | -1.60 | Sepsis_D0_non-survivor down vs Sepsis_D0_survivor |
| NAPG | 3.37E-04 | -1.60 | Sepsis_D0_non-survivor down vs Sepsis_D0_survivor |
| AGTPBP1 | 4.96E-05 | -1.60 | Sepsis_D0_non-survivor down vs Sepsis_D0_survivor |
| MTDH | 1.43E-05 | -1.60 | Sepsis_D0_non-survivor down vs Sepsis_D0_survivor |
| GBA2 | 1.54E-03 | -1.59 | Sepsis_D0_non-survivor down vs Sepsis_D0_survivor |
| RNF138 | 6.20E-03 | -1.59 | Sepsis_D0_non-survivor down vs Sepsis_D0_survivor |
| GBAS | 2.43E-03 | -1.59 | Sepsis_D0_non-survivor down vs Sepsis_D0_survivor |
| RMND5A | 5.38E-03 | -1.59 | Sepsis_D0_non-survivor down vs Sepsis_D0_survivor |
| IL10RB | 2.22E-03 | -1.59 | Sepsis_D0_non-survivor down vs Sepsis_D0_survivor |
| SPIN | 2.40E-03 | -1.59 | Sepsis_D0_non-survivor down vs Sepsis_D0_survivor |
| ARPC4 | 5.50E-04 | -1.59 | Sepsis_D0_non-survivor down vs Sepsis_D0_survivor |
| TMEM71 | 2.74E-03 | -1.59 | Sepsis_D0_non-survivor down vs Sepsis_D0_survivor |
| XRN1 | 6.08E-03 | -1.59 | Sepsis_D0_non-survivor down vs Sepsis_D0_survivor |
| FXC1 | 7.12E-03 | -1.59 | Sepsis_D0_non-survivor down vs Sepsis_D0_survivor |
| CTDSPL2 | 4.54E-03 | -1.59 | Sepsis_D0_non-survivor down vs Sepsis_D0_survivor |
| FAM33A | 6.90E-03 | -1.59 | Sepsis_D0_non-survivor down vs Sepsis_D0_survivor |
| EIF2B3 | 1.49E-03 | -1.59 | Sepsis_D0_non-survivor down vs Sepsis_D0_survivor |
| RAB1A | 3.41E-03 | -1.59 | Sepsis_D0_non-survivor down vs Sepsis_D0_survivor |
| CRBN | 2.41E-04 | -1.59 | Sepsis_D0_non-survivor down vs Sepsis_D0_survivor |
| WDR61 | 6.18E-04 | -1.59 | Sepsis_D0_non-survivor down vs Sepsis_D0_survivor |
| PLGLB2 | 7.80E-04 | -1.59 | Sepsis_D0_non-survivor down vs Sepsis_D0_survivor |
| CHORDC1 | 5.32E-04 | -1.59 | Sepsis_D0_non-survivor down vs Sepsis_D0_survivor |
| PEX19 | 1.76E-03 | -1.59 | Sepsis_D0_non-survivor down vs Sepsis_D0_survivor |
| CTAGEP | 1.33E-03 | -1.59 | Sepsis_D0_non-survivor down vs Sepsis_D0_survivor |
| KCTD3 | 8.05E-04 | -1.59 | Sepsis_D0_non-survivor down vs Sepsis_D0_survivor |
| TRAM2 | 5.38E-03 | -1.59 | Sepsis_D0_non-survivor down vs Sepsis_D0_survivor |
| RABL4 | 6.39E-06 | -1.58 | Sepsis_D0_non-survivor down vs Sepsis_D0_survivor |
| TBC1D22B | 7.47E-03 | -1.58 | Sepsis_D0_non-survivor down vs Sepsis_D0_survivor |
| SWS1 | 5.32E-03 | -1.58 | Sepsis_D0_non-survivor down vs Sepsis_D0_survivor |
| PTPLAD1 | 6.38E-03 | -1.58 | Sepsis_D0_non-survivor down vs Sepsis_D0_survivor |
| HIRIP5 | 1.02E-03 | -1.58 | Sepsis_D0_non-survivor down vs Sepsis_D0_survivor |
| ZMYM6 | 3.16E-03 | -1.58 | Sepsis_D0_non-survivor down vs Sepsis_D0_survivor |
| BICD2 | 1.35E-03 | -1.58 | Sepsis_D0_non-survivor down vs Sepsis_D0_survivor |
| LGALS8 | 2.17E-03 | -1.58 | Sepsis_D0_non-survivor down vs Sepsis_D0_survivor |
| ZNF25 | 6.32E-03 | -1.58 | Sepsis_D0_non-survivor down vs Sepsis_D0_survivor |
| ANAPC4 | 7.36E-03 | -1.58 | Sepsis_D0_non-survivor down vs Sepsis_D0_survivor |
| UGP2 | 2.74E-04 | -1.58 | Sepsis_D0_non-survivor down vs Sepsis_D0_survivor |
| PTGES2 | 2.55E-04 | -1.58 | Sepsis_D0_non-survivor down vs Sepsis_D0_survivor |
| CAMK2G | 3.77E-05 | -1.58 | Sepsis_D0_non-survivor down vs Sepsis_D0_survivor |
| MBP | 6.72E-04 | -1.58 | Sepsis_D0_non-survivor down vs Sepsis_D0_survivor |
| UTP11L | 3.03E-03 | -1.58 | Sepsis_D0_non-survivor down vs Sepsis_D0_survivor |
| RPN2 | 1.11E-04 | -1.58 | Sepsis_D0_non-survivor down vs Sepsis_D0_survivor |
| LAPTM4A | 1.74E-04 | -1.58 | Sepsis_D0_non-survivor down vs Sepsis_D0_survivor |
| NDUFB6 | 6.09E-05 | -1.58 | Sepsis_D0_non-survivor down vs Sepsis_D0_survivor |
| MOBK1B | 4.55E-03 | -1.57 | Sepsis_D0_non-survivor down vs Sepsis_D0_survivor |
| LRRC57 | 2.67E-03 | -1.57 | Sepsis_D0_non-survivor down vs Sepsis_D0_survivor |
| SNX12 | 1.47E-03 | -1.57 | Sepsis_D0_non-survivor down vs Sepsis_D0_survivor |
| EXOC5 | 5.08E-03 | -1.57 | Sepsis_D0_non-survivor down vs Sepsis_D0_survivor |
| C1D | 2.62E-03 | -1.57 | Sepsis_D0_non-survivor down vs Sepsis_D0_survivor |
| TRIAP1 | 5.18E-03 | -1.57 | Sepsis_D0_non-survivor down vs Sepsis_D0_survivor |
| GMCL1 | 3.31E-04 | -1.57 | Sepsis_D0_non-survivor down vs Sepsis_D0_survivor |
| ZNF690 | 3.59E-04 | -1.57 | Sepsis_D0_non-survivor down vs Sepsis_D0_survivor |
| CLU | 3.66E-03 | -1.57 | Sepsis_D0_non-survivor down vs Sepsis_D0_survivor |
| BIK | 4.76E-03 | -1.57 | Sepsis_D0_non-survivor down vs Sepsis_D0_survivor |
| ADCY9 | 3.70E-03 | -1.57 | Sepsis_D0_non-survivor down vs Sepsis_D0_survivor |
| ACADS | 3.13E-03 | -1.57 | Sepsis_D0_non-survivor down vs Sepsis_D0_survivor |
| ZNF518 | 2.01E-03 | -1.57 | Sepsis_D0_non-survivor down vs Sepsis_D0_survivor |
| ALAD | 5.68E-04 | -1.57 | Sepsis_D0_non-survivor down vs Sepsis_D0_survivor |
| GOLT1B | 3.68E-03 | -1.57 | Sepsis_D0_non-survivor down vs Sepsis_D0_survivor |
| LRRC39 | 5.71E-03 | -1.57 | Sepsis_D0_non-survivor down vs Sepsis_D0_survivor |
| PDSS2 | 2.42E-03 | -1.57 | Sepsis_D0_non-survivor down vs Sepsis_D0_survivor |
| PTPN22 | 8.43E-04 | -1.57 | Sepsis_D0_non-survivor down vs Sepsis_D0_survivor |
| RSRC1 | 1.18E-03 | -1.57 | Sepsis_D0_non-survivor down vs Sepsis_D0_survivor |
| TXNDC13 | 1.19E-04 | -1.57 | Sepsis_D0_non-survivor down vs Sepsis_D0_survivor |
| PIK3R4 | 2.94E-03 | -1.57 | Sepsis_D0_non-survivor down vs Sepsis_D0_survivor |
| HSPBAP1 | 3.94E-03 | -1.57 | Sepsis_D0_non-survivor down vs Sepsis_D0_survivor |
| IKBKAP | 5.56E-04 | -1.57 | Sepsis_D0_non-survivor down vs Sepsis_D0_survivor |
| PKP4 | 4.60E-03 | -1.57 | Sepsis_D0_non-survivor down vs Sepsis_D0_survivor |
| DPY19L3 | 5.84E-03 | -1.57 | Sepsis_D0_non-survivor down vs Sepsis_D0_survivor |
| RALY | 1.14E-03 | -1.56 | Sepsis_D0_non-survivor down vs Sepsis_D0_survivor |
| PLDN | 1.58E-03 | -1.56 | Sepsis_D0_non-survivor down vs Sepsis_D0_survivor |
| CAB39 | 3.59E-03 | -1.56 | Sepsis_D0_non-survivor down vs Sepsis_D0_survivor |
| TXNL2 | 4.96E-04 | -1.56 | Sepsis_D0_non-survivor down vs Sepsis_D0_survivor |
| FAM44C | 5.94E-04 | -1.56 | Sepsis_D0_non-survivor down vs Sepsis_D0_survivor |
| TGFBI | 4.57E-03 | -1.56 | Sepsis_D0_non-survivor down vs Sepsis_D0_survivor |
| BET3L | 4.34E-03 | -1.56 | Sepsis_D0_non-survivor down vs Sepsis_D0_survivor |
| PPP4R1 | 1.39E-03 | -1.56 | Sepsis_D0_non-survivor down vs Sepsis_D0_survivor |
| ATP8B3 | 3.52E-03 | -1.56 | Sepsis_D0_non-survivor down vs Sepsis_D0_survivor |
| COQ3 | 4.50E-03 | -1.56 | Sepsis_D0_non-survivor down vs Sepsis_D0_survivor |
| SIGLEC7 | 1.03E-03 | -1.56 | Sepsis_D0_non-survivor down vs Sepsis_D0_survivor |
| FAM48A | 2.29E-03 | -1.56 | Sepsis_D0_non-survivor down vs Sepsis_D0_survivor |
| PIGB | 4.16E-03 | -1.56 | Sepsis_D0_non-survivor down vs Sepsis_D0_survivor |
| SLC23A2 | 1.92E-03 | -1.56 | Sepsis_D0_non-survivor down vs Sepsis_D0_survivor |
| ARRB1 | 6.73E-03 | -1.56 | Sepsis_D0_non-survivor down vs Sepsis_D0_survivor |
| ZNF222 | 1.55E-04 | -1.56 | Sepsis_D0_non-survivor down vs Sepsis_D0_survivor |
| CHMP5 | 1.59E-03 | -1.56 | Sepsis_D0_non-survivor down vs Sepsis_D0_survivor |
| CRYZL1 | 2.57E-04 | -1.56 | Sepsis_D0_non-survivor down vs Sepsis_D0_survivor |
| ABHD13 | 4.76E-03 | -1.56 | Sepsis_D0_non-survivor down vs Sepsis_D0_survivor |
| HPS5 | 3.01E-03 | -1.56 | Sepsis_D0_non-survivor down vs Sepsis_D0_survivor |
| ARL2BP | 4.58E-05 | -1.55 | Sepsis_D0_non-survivor down vs Sepsis_D0_survivor |
| PCBD2 | 5.17E-04 | -1.55 | Sepsis_D0_non-survivor down vs Sepsis_D0_survivor |
| PMS2L2 | 1.20E-03 | -1.55 | Sepsis_D0_non-survivor down vs Sepsis_D0_survivor |
| CAT | 6.05E-03 | -1.55 | Sepsis_D0_non-survivor down vs Sepsis_D0_survivor |
| SCCPDH | 6.54E-04 | -1.55 | Sepsis_D0_non-survivor down vs Sepsis_D0_survivor |
| PIP5K1B | 3.58E-03 | -1.55 | Sepsis_D0_non-survivor down vs Sepsis_D0_survivor |
| SLC39A9 | 2.12E-03 | -1.55 | Sepsis_D0_non-survivor down vs Sepsis_D0_survivor |
| LEMD3 | 2.39E-03 | -1.55 | Sepsis_D0_non-survivor down vs Sepsis_D0_survivor |
| ZNF322B | 1.72E-03 | -1.55 | Sepsis_D0_non-survivor down vs Sepsis_D0_survivor |
| DENND2D | 6.60E-03 | -1.55 | Sepsis_D0_non-survivor down vs Sepsis_D0_survivor |
| HIATL1 | 6.61E-03 | -1.55 | Sepsis_D0_non-survivor down vs Sepsis_D0_survivor |
| TATDN3 | 2.77E-03 | -1.55 | Sepsis_D0_non-survivor down vs Sepsis_D0_survivor |
| SEC24C | 8.75E-04 | -1.55 | Sepsis_D0_non-survivor down vs Sepsis_D0_survivor |
| PTPLAD2 | 4.21E-03 | -1.55 | Sepsis_D0_non-survivor down vs Sepsis_D0_survivor |
| NSF | 3.01E-03 | -1.55 | Sepsis_D0_non-survivor down vs Sepsis_D0_survivor |
| HTRA4 | 5.75E-03 | -1.55 | Sepsis_D0_non-survivor down vs Sepsis_D0_survivor |
| TRSPAP1 | 4.74E-03 | -1.55 | Sepsis_D0_non-survivor down vs Sepsis_D0_survivor |
| OAT | 4.54E-04 | -1.55 | Sepsis_D0_non-survivor down vs Sepsis_D0_survivor |
| MGMT | 2.23E-07 | -1.55 | Sepsis_D0_non-survivor down vs Sepsis_D0_survivor |
| MDS025 | 6.48E-03 | -1.55 | Sepsis_D0_non-survivor down vs Sepsis_D0_survivor |
| BP75 | 1.64E-03 | -1.54 | Sepsis_D0_non-survivor down vs Sepsis_D0_survivor |
| EFHC1 | 3.53E-03 | -1.54 | Sepsis_D0_non-survivor down vs Sepsis_D0_survivor |
| MAP3K7IP3 | 5.16E-03 | -1.54 | Sepsis_D0_non-survivor down vs Sepsis_D0_survivor |
| PECI | 5.27E-04 | -1.54 | Sepsis_D0_non-survivor down vs Sepsis_D0_survivor |
| CR1 | 1.58E-03 | -1.54 | Sepsis_D0_non-survivor down vs Sepsis_D0_survivor |
| MTRF1 | 2.77E-03 | -1.54 | Sepsis_D0_non-survivor down vs Sepsis_D0_survivor |
| CLK4 | 5.06E-04 | -1.54 | Sepsis_D0_non-survivor down vs Sepsis_D0_survivor |
| FNTA | 4.68E-03 | -1.54 | Sepsis_D0_non-survivor down vs Sepsis_D0_survivor |
| INPP5F | 5.17E-03 | -1.54 | Sepsis_D0_non-survivor down vs Sepsis_D0_survivor |
| WHSC1 | 2.06E-03 | -1.54 | Sepsis_D0_non-survivor down vs Sepsis_D0_survivor |
| RFC1 | 9.76E-04 | -1.54 | Sepsis_D0_non-survivor down vs Sepsis_D0_survivor |
| ZDHHC17 | 2.08E-03 | -1.54 | Sepsis_D0_non-survivor down vs Sepsis_D0_survivor |
| PRDX3 | 3.48E-03 | -1.54 | Sepsis_D0_non-survivor down vs Sepsis_D0_survivor |
| MTX2 | 2.22E-03 | -1.53 | Sepsis_D0_non-survivor down vs Sepsis_D0_survivor |
| PCDHAC2 | 1.51E-04 | -1.53 | Sepsis_D0_non-survivor down vs Sepsis_D0_survivor |
| SQRDL | 4.43E-03 | -1.53 | Sepsis_D0_non-survivor down vs Sepsis_D0_survivor |
| SRPK2 | 1.60E-03 | -1.53 | Sepsis_D0_non-survivor down vs Sepsis_D0_survivor |
| MPPE1 | 2.36E-03 | -1.53 | Sepsis_D0_non-survivor down vs Sepsis_D0_survivor |
| ZNF300 | 4.41E-03 | -1.53 | Sepsis_D0_non-survivor down vs Sepsis_D0_survivor |
| SRP54 | 1.49E-03 | -1.53 | Sepsis_D0_non-survivor down vs Sepsis_D0_survivor |
| SLC9A6 | 1.49E-03 | -1.53 | Sepsis_D0_non-survivor down vs Sepsis_D0_survivor |
| SEC22C | 1.79E-03 | -1.53 | Sepsis_D0_non-survivor down vs Sepsis_D0_survivor |
| SKIV2L2 | 5.71E-03 | -1.53 | Sepsis_D0_non-survivor down vs Sepsis_D0_survivor |
| FAM11A | 3.07E-03 | -1.53 | Sepsis_D0_non-survivor down vs Sepsis_D0_survivor |
| CCDC89 | 1.33E-04 | -1.53 | Sepsis_D0_non-survivor down vs Sepsis_D0_survivor |
| INVS | 9.78E-04 | -1.53 | Sepsis_D0_non-survivor down vs Sepsis_D0_survivor |
| MTFMT | 9.15E-04 | -1.53 | Sepsis_D0_non-survivor down vs Sepsis_D0_survivor |
| COPS5 | 2.86E-03 | -1.53 | Sepsis_D0_non-survivor down vs Sepsis_D0_survivor |
| PRKAB2 | 3.91E-04 | -1.53 | Sepsis_D0_non-survivor down vs Sepsis_D0_survivor |
| ACTG2 | 4.86E-03 | -1.53 | Sepsis_D0_non-survivor down vs Sepsis_D0_survivor |
| ACTR2 | 1.16E-03 | -1.53 | Sepsis_D0_non-survivor down vs Sepsis_D0_survivor |
| BAK1 | 6.54E-03 | -1.53 | Sepsis_D0_non-survivor down vs Sepsis_D0_survivor |
| ADCK2 | 3.64E-04 | -1.53 | Sepsis_D0_non-survivor down vs Sepsis_D0_survivor |
| MTHFS | 7.46E-03 | -1.52 | Sepsis_D0_non-survivor down vs Sepsis_D0_survivor |
| MMAA | 3.25E-03 | -1.52 | Sepsis_D0_non-survivor down vs Sepsis_D0_survivor |
| NSMCE1 | 1.12E-03 | -1.52 | Sepsis_D0_non-survivor down vs Sepsis_D0_survivor |
| RAB20 | 6.79E-04 | -1.52 | Sepsis_D0_non-survivor down vs Sepsis_D0_survivor |
| ZNF558 | 2.02E-03 | -1.52 | Sepsis_D0_non-survivor down vs Sepsis_D0_survivor |
| CTPS | 7.30E-03 | -1.52 | Sepsis_D0_non-survivor down vs Sepsis_D0_survivor |
| KPNA4 | 6.80E-03 | -1.52 | Sepsis_D0_non-survivor down vs Sepsis_D0_survivor |
| LPGAT1 | 3.69E-03 | -1.52 | Sepsis_D0_non-survivor down vs Sepsis_D0_survivor |
| IPPK | 4.76E-03 | -1.52 | Sepsis_D0_non-survivor down vs Sepsis_D0_survivor |
| GPD2 | 2.80E-06 | -1.52 | Sepsis_D0_non-survivor down vs Sepsis_D0_survivor |
| MYO5A | 7.20E-03 | -1.52 | Sepsis_D0_non-survivor down vs Sepsis_D0_survivor |
| SEMA7A | 1.59E-03 | -1.52 | Sepsis_D0_non-survivor down vs Sepsis_D0_survivor |
| HADHSC | 5.14E-03 | -1.52 | Sepsis_D0_non-survivor down vs Sepsis_D0_survivor |
| L09234 | 4.55E-03 | -1.52 | Sepsis_D0_non-survivor down vs Sepsis_D0_survivor |
| PHACTR3 | 5.32E-03 | -1.52 | Sepsis_D0_non-survivor down vs Sepsis_D0_survivor |
| ACAA2 | 1.88E-04 | -1.52 | Sepsis_D0_non-survivor down vs Sepsis_D0_survivor |
| PRPF18 | 3.63E-04 | -1.51 | Sepsis_D0_non-survivor down vs Sepsis_D0_survivor |
| KREMEN1 | 1.32E-03 | -1.51 | Sepsis_D0_non-survivor down vs Sepsis_D0_survivor |
| TOPBP1 | 1.69E-03 | -1.51 | Sepsis_D0_non-survivor down vs Sepsis_D0_survivor |
| MCFD2 | 2.02E-03 | -1.51 | Sepsis_D0_non-survivor down vs Sepsis_D0_survivor |
| DPP8 | 1.21E-03 | -1.51 | Sepsis_D0_non-survivor down vs Sepsis_D0_survivor |
| CEP27 | 6.36E-03 | -1.51 | Sepsis_D0_non-survivor down vs Sepsis_D0_survivor |
| UIP1 | 4.85E-03 | -1.51 | Sepsis_D0_non-survivor down vs Sepsis_D0_survivor |
| GLT1D1 | 8.05E-05 | -1.51 | Sepsis_D0_non-survivor down vs Sepsis_D0_survivor |
| CYP20A1 | 3.00E-03 | -1.51 | Sepsis_D0_non-survivor down vs Sepsis_D0_survivor |
| PCMT1 | 6.15E-03 | -1.51 | Sepsis_D0_non-survivor down vs Sepsis_D0_survivor |
| INHBE | 7.25E-03 | -1.51 | Sepsis_D0_non-survivor down vs Sepsis_D0_survivor |
| VPS54 | 1.40E-04 | -1.51 | Sepsis_D0_non-survivor down vs Sepsis_D0_survivor |
| UBE2Q1 | 8.02E-04 | -1.51 | Sepsis_D0_non-survivor down vs Sepsis_D0_survivor |
| SRXN1 | 9.20E-04 | -1.51 | Sepsis_D0_non-survivor down vs Sepsis_D0_survivor |
| LEPROTL1 | 1.72E-03 | -1.51 | Sepsis_D0_non-survivor down vs Sepsis_D0_survivor |
| CA5B | 1.20E-03 | -1.51 | Sepsis_D0_non-survivor down vs Sepsis_D0_survivor |
| USP47 | 4.16E-04 | -1.51 | Sepsis_D0_non-survivor down vs Sepsis_D0_survivor |
| FAM8A1 | 6.72E-03 | -1.51 | Sepsis_D0_non-survivor down vs Sepsis_D0_survivor |
| IBTK | 2.46E-03 | -1.51 | Sepsis_D0_non-survivor down vs Sepsis_D0_survivor |
| MRPL33 | 1.60E-03 | -1.51 | Sepsis_D0_non-survivor down vs Sepsis_D0_survivor |
| DTWD1 | 7.36E-03 | -1.51 | Sepsis_D0_non-survivor down vs Sepsis_D0_survivor |
| DNM1L | 3.21E-03 | -1.51 | Sepsis_D0_non-survivor down vs Sepsis_D0_survivor |
| GRSF1 | 1.29E-03 | -1.51 | Sepsis_D0_non-survivor down vs Sepsis_D0_survivor |
| KIDINS220 | 1.71E-03 | -1.51 | Sepsis_D0_non-survivor down vs Sepsis_D0_survivor |
| GTF2A2 | 5.65E-03 | -1.51 | Sepsis_D0_non-survivor down vs Sepsis_D0_survivor |
| LDOC1L | 1.05E-03 | -1.51 | Sepsis_D0_non-survivor down vs Sepsis_D0_survivor |
| UBP1 | 1.05E-03 | -1.51 | Sepsis_D0_non-survivor down vs Sepsis_D0_survivor |
| AP3S2 | 5.54E-04 | -1.51 | Sepsis_D0_non-survivor down vs Sepsis_D0_survivor |
| ATP9B | 6.90E-03 | -1.51 | Sepsis_D0_non-survivor down vs Sepsis_D0_survivor |
| GNRH1 | 4.61E-03 | -1.50 | Sepsis_D0_non-survivor down vs Sepsis_D0_survivor |
| HAGH | 2.72E-03 | -1.50 | Sepsis_D0_non-survivor down vs Sepsis_D0_survivor |
| TMEM70 | 5.21E-03 | -1.50 | Sepsis_D0_non-survivor down vs Sepsis_D0_survivor |
| ZFAND2A | 5.82E-03 | -1.50 | Sepsis_D0_non-survivor down vs Sepsis_D0_survivor |
| TMEM126A | 6.30E-04 | -1.50 | Sepsis_D0_non-survivor down vs Sepsis_D0_survivor |
| CANX | 5.16E-03 | -1.50 | Sepsis_D0_non-survivor down vs Sepsis_D0_survivor |
| NUP54 | 3.96E-03 | -1.50 | Sepsis_D0_non-survivor down vs Sepsis_D0_survivor |
| GCLC | 3.79E-03 | -1.50 | Sepsis_D0_non-survivor down vs Sepsis_D0_survivor |
| TM7SF3 | 8.54E-04 | -1.50 | Sepsis_D0_non-survivor down vs Sepsis_D0_survivor |
| UVRAG | 5.29E-03 | -1.50 | Sepsis_D0_non-survivor down vs Sepsis_D0_survivor |
| DCTN6 | 6.20E-04 | -1.50 | Sepsis_D0_non-survivor down vs Sepsis_D0_survivor |
| UBR1 | 3.71E-03 | -1.50 | Sepsis_D0_non-survivor down vs Sepsis_D0_survivor |
| RPS6KB1 | 5.41E-03 | -1.50 | Sepsis_D0_non-survivor down vs Sepsis_D0_survivor |
| KCTD18 | 1.91E-03 | -1.50 | Sepsis_D0_non-survivor down vs Sepsis_D0_survivor |
| SLC38A6 | 2.55E-03 | -1.50 | Sepsis_D0_non-survivor down vs Sepsis_D0_survivor |
| DEK | 2.79E-03 | -1.50 | Sepsis_D0_non-survivor down vs Sepsis_D0_survivor |
| NAT12 | 2.40E-03 | -1.50 | Sepsis_D0_non-survivor down vs Sepsis_D0_survivor |
| ADH5 | 5.14E-04 | -1.50 | Sepsis_D0_non-survivor down vs Sepsis_D0_survivor |
| TXNDC4 | 1.56E-04 | -1.50 | Sepsis_D0_non-survivor down vs Sepsis_D0_survivor |
| USP8 | 5.93E-04 | -1.50 | Sepsis_D0_non-survivor down vs Sepsis_D0_survivor |
| FKBP3 | 1.29E-04 | -1.50 | Sepsis_D0_non-survivor down vs Sepsis_D0_survivor |
| THOC7 | 5.16E-03 | -1.50 | Sepsis_D0_non-survivor down vs Sepsis_D0_survivor |
| COMMD8 | 4.21E-03 | -1.50 | Sepsis_D0_non-survivor down vs Sepsis_D0_survivor |
| UFM1 | 2.17E-04 | -1.50 | Sepsis_D0_non-survivor down vs Sepsis_D0_survivor |
| BMI1 | 1.61E-03 | -1.50 | Sepsis_D0_non-survivor down vs Sepsis_D0_survivor |
| HTRA3 | 8.40E-04 | -1.50 | Sepsis_D0_non-survivor down vs Sepsis_D0_survivor |
| NDUFA5 | 1.31E-03 | -1.50 | Sepsis_D0_non-survivor down vs Sepsis_D0_survivor |
| JTV1 | 2.19E-03 | -1.50 | Sepsis_D0_non-survivor down vs Sepsis_D0_survivor |
| MTERFD3 | 5.71E-03 | -1.50 | Sepsis_D0_non-survivor down vs Sepsis_D0_survivor |
| DLD | 7.46E-04 | -1.50 | Sepsis_D0_non-survivor down vs Sepsis_D0_survivor |
| GAS7 | 6.01E-04 | -1.49 | Sepsis_D0_non-survivor down vs Sepsis_D0_survivor |
| FAIM | 4.64E-04 | -1.49 | Sepsis_D0_non-survivor down vs Sepsis_D0_survivor |
| CD164 | 7.12E-03 | -1.49 | Sepsis_D0_non-survivor down vs Sepsis_D0_survivor |
| IFNAR1 | 7.59E-03 | -1.49 | Sepsis_D0_non-survivor down vs Sepsis_D0_survivor |
| PEX7 | 7.16E-03 | -1.49 | Sepsis_D0_non-survivor down vs Sepsis_D0_survivor |
| WDR36 | 4.65E-03 | -1.49 | Sepsis_D0_non-survivor down vs Sepsis_D0_survivor |
| AUH | 2.17E-03 | -1.49 | Sepsis_D0_non-survivor down vs Sepsis_D0_survivor |
| ACOX1 | 5.61E-03 | -1.49 | Sepsis_D0_non-survivor down vs Sepsis_D0_survivor |
| UBPH | 1.94E-03 | -1.49 | Sepsis_D0_non-survivor down vs Sepsis_D0_survivor |
| MAGEA9 | 5.18E-03 | -1.49 | Sepsis_D0_non-survivor down vs Sepsis_D0_survivor |
| ADCK1 | 6.21E-03 | -1.49 | Sepsis_D0_non-survivor down vs Sepsis_D0_survivor |
| MGST2 | 1.73E-03 | -1.49 | Sepsis_D0_non-survivor down vs Sepsis_D0_survivor |
| MALT1 | 5.71E-03 | -1.49 | Sepsis_D0_non-survivor down vs Sepsis_D0_survivor |
| MED28 | 1.44E-03 | -1.49 | Sepsis_D0_non-survivor down vs Sepsis_D0_survivor |
| PTPN2 | 3.81E-03 | -1.49 | Sepsis_D0_non-survivor down vs Sepsis_D0_survivor |
| RTCD1 | 2.02E-03 | -1.49 | Sepsis_D0_non-survivor down vs Sepsis_D0_survivor |
| ACADSB | 8.41E-04 | -1.49 | Sepsis_D0_non-survivor down vs Sepsis_D0_survivor |
| USP31 | 5.42E-03 | -1.49 | Sepsis_D0_non-survivor down vs Sepsis_D0_survivor |
| HOOK3 | 2.81E-04 | -1.49 | Sepsis_D0_non-survivor down vs Sepsis_D0_survivor |
| TBPL1 | 9.43E-04 | -1.49 | Sepsis_D0_non-survivor down vs Sepsis_D0_survivor |
| ETFA | 6.73E-03 | -1.49 | Sepsis_D0_non-survivor down vs Sepsis_D0_survivor |
| ABCC5 | 5.73E-03 | -1.49 | Sepsis_D0_non-survivor down vs Sepsis_D0_survivor |
| FUBP1 | 7.72E-03 | -1.49 | Sepsis_D0_non-survivor down vs Sepsis_D0_survivor |
| DPM1 | 3.45E-04 | -1.49 | Sepsis_D0_non-survivor down vs Sepsis_D0_survivor |
| MON2 | 4.96E-03 | -1.49 | Sepsis_D0_non-survivor down vs Sepsis_D0_survivor |
| RBM18 | 2.54E-03 | -1.49 | Sepsis_D0_non-survivor down vs Sepsis_D0_survivor |
| MYNN | 5.19E-03 | -1.49 | Sepsis_D0_non-survivor down vs Sepsis_D0_survivor |
| NARG2 | 2.24E-03 | -1.48 | Sepsis_D0_non-survivor down vs Sepsis_D0_survivor |
| TMEM109 | 8.63E-04 | -1.48 | Sepsis_D0_non-survivor down vs Sepsis_D0_survivor |
| PHC3 | 4.07E-03 | -1.48 | Sepsis_D0_non-survivor down vs Sepsis_D0_survivor |
| GGCX | 4.02E-03 | -1.48 | Sepsis_D0_non-survivor down vs Sepsis_D0_survivor |
| TPRKB | 4.89E-03 | -1.48 | Sepsis_D0_non-survivor down vs Sepsis_D0_survivor |
| CHRNB1 | 6.67E-03 | -1.48 | Sepsis_D0_non-survivor down vs Sepsis_D0_survivor |
| TRIP12 | 2.39E-03 | -1.48 | Sepsis_D0_non-survivor down vs Sepsis_D0_survivor |
| RIOK1 | 3.67E-03 | -1.48 | Sepsis_D0_non-survivor down vs Sepsis_D0_survivor |
| BRD7 | 4.54E-04 | -1.48 | Sepsis_D0_non-survivor down vs Sepsis_D0_survivor |
| VPS29 | 7.61E-04 | -1.48 | Sepsis_D0_non-survivor down vs Sepsis_D0_survivor |
| SIRT3 | 3.86E-04 | -1.48 | Sepsis_D0_non-survivor down vs Sepsis_D0_survivor |
| PUS7L | 2.21E-03 | -1.48 | Sepsis_D0_non-survivor down vs Sepsis_D0_survivor |
| PRPF39 | 1.93E-03 | -1.48 | Sepsis_D0_non-survivor down vs Sepsis_D0_survivor |
| SERTAD2 | 5.72E-03 | -1.48 | Sepsis_D0_non-survivor down vs Sepsis_D0_survivor |
| PCGF5 | 5.40E-03 | -1.48 | Sepsis_D0_non-survivor down vs Sepsis_D0_survivor |
| LZIC | 1.28E-03 | -1.48 | Sepsis_D0_non-survivor down vs Sepsis_D0_survivor |
| FER | 1.84E-03 | -1.48 | Sepsis_D0_non-survivor down vs Sepsis_D0_survivor |
| MRPL1 | 4.67E-03 | -1.48 | Sepsis_D0_non-survivor down vs Sepsis_D0_survivor |
| CCNC | 1.66E-03 | -1.48 | Sepsis_D0_non-survivor down vs Sepsis_D0_survivor |
| VPS36 | 3.56E-04 | -1.48 | Sepsis_D0_non-survivor down vs Sepsis_D0_survivor |
| BRE | 1.79E-03 | -1.48 | Sepsis_D0_non-survivor down vs Sepsis_D0_survivor |
| PCF11 | 3.20E-03 | -1.48 | Sepsis_D0_non-survivor down vs Sepsis_D0_survivor |
| UQCRC2 | 5.35E-03 | -1.47 | Sepsis_D0_non-survivor down vs Sepsis_D0_survivor |
| HISPPD1 | 3.66E-03 | -1.47 | Sepsis_D0_non-survivor down vs Sepsis_D0_survivor |
| SUPT7L | 4.18E-04 | -1.47 | Sepsis_D0_non-survivor down vs Sepsis_D0_survivor |
| SEC23A | 2.36E-03 | -1.47 | Sepsis_D0_non-survivor down vs Sepsis_D0_survivor |
| ACSS2 | 6.16E-03 | -1.47 | Sepsis_D0_non-survivor down vs Sepsis_D0_survivor |
| KCTD10 | 5.89E-03 | -1.47 | Sepsis_D0_non-survivor down vs Sepsis_D0_survivor |
| NUDCD2 | 4.12E-04 | -1.47 | Sepsis_D0_non-survivor down vs Sepsis_D0_survivor |
| PRKRA | 4.60E-03 | -1.47 | Sepsis_D0_non-survivor down vs Sepsis_D0_survivor |
| FBXO31 | 6.37E-04 | -1.47 | Sepsis_D0_non-survivor down vs Sepsis_D0_survivor |
| DNAJA5 | 3.93E-04 | -1.47 | Sepsis_D0_non-survivor down vs Sepsis_D0_survivor |
| SDF2 | 1.12E-03 | -1.47 | Sepsis_D0_non-survivor down vs Sepsis_D0_survivor |
| TRAPPC2 | 3.70E-03 | -1.47 | Sepsis_D0_non-survivor down vs Sepsis_D0_survivor |
| ZNF294 | 6.08E-03 | -1.47 | Sepsis_D0_non-survivor down vs Sepsis_D0_survivor |
| CDK2AP1 | 6.80E-03 | -1.47 | Sepsis_D0_non-survivor down vs Sepsis_D0_survivor |
| METTL5 | 1.51E-05 | -1.47 | Sepsis_D0_non-survivor down vs Sepsis_D0_survivor |
| RBM34 | 2.23E-03 | -1.47 | Sepsis_D0_non-survivor down vs Sepsis_D0_survivor |
| SURF6 | 7.09E-03 | -1.47 | Sepsis_D0_non-survivor down vs Sepsis_D0_survivor |
| ZNF84 | 3.51E-03 | -1.47 | Sepsis_D0_non-survivor down vs Sepsis_D0_survivor |
| FNBP1L | 4.70E-03 | -1.47 | Sepsis_D0_non-survivor down vs Sepsis_D0_survivor |
| ZNF283 | 8.31E-04 | -1.47 | Sepsis_D0_non-survivor down vs Sepsis_D0_survivor |
| F5 | 1.94E-04 | -1.47 | Sepsis_D0_non-survivor down vs Sepsis_D0_survivor |
| VPS26B | 3.60E-03 | -1.47 | Sepsis_D0_non-survivor down vs Sepsis_D0_survivor |
| EXT2 | 1.09E-03 | -1.47 | Sepsis_D0_non-survivor down vs Sepsis_D0_survivor |
| RBMS1 | 4.54E-03 | -1.47 | Sepsis_D0_non-survivor down vs Sepsis_D0_survivor |
| REXO2 | 2.88E-03 | -1.47 | Sepsis_D0_non-survivor down vs Sepsis_D0_survivor |
| RWDD3 | 1.44E-03 | -1.46 | Sepsis_D0_non-survivor down vs Sepsis_D0_survivor |
| C1GALT1C1 | 2.80E-03 | -1.46 | Sepsis_D0_non-survivor down vs Sepsis_D0_survivor |
| AKAP7 | 3.94E-03 | -1.46 | Sepsis_D0_non-survivor down vs Sepsis_D0_survivor |
| RAD17 | 2.70E-03 | -1.46 | Sepsis_D0_non-survivor down vs Sepsis_D0_survivor |
| PSMA2 | 1.15E-03 | -1.46 | Sepsis_D0_non-survivor down vs Sepsis_D0_survivor |
| ACTR10 | 7.36E-03 | -1.46 | Sepsis_D0_non-survivor down vs Sepsis_D0_survivor |
| ZDHHC6 | 5.78E-03 | -1.46 | Sepsis_D0_non-survivor down vs Sepsis_D0_survivor |
| TRIO | 2.66E-03 | -1.46 | Sepsis_D0_non-survivor down vs Sepsis_D0_survivor |
| CEP57 | 2.07E-04 | -1.46 | Sepsis_D0_non-survivor down vs Sepsis_D0_survivor |
| GMFB | 2.76E-04 | -1.46 | Sepsis_D0_non-survivor down vs Sepsis_D0_survivor |
| UCHL3 | 5.69E-03 | -1.46 | Sepsis_D0_non-survivor down vs Sepsis_D0_survivor |
| TMED5 | 1.34E-03 | -1.46 | Sepsis_D0_non-survivor down vs Sepsis_D0_survivor |
| CHST5 | 1.70E-03 | -1.46 | Sepsis_D0_non-survivor down vs Sepsis_D0_survivor |
| SERPINB6 | 1.40E-03 | -1.46 | Sepsis_D0_non-survivor down vs Sepsis_D0_survivor |
| DOCK11 | 1.92E-03 | -1.46 | Sepsis_D0_non-survivor down vs Sepsis_D0_survivor |
| NIPSNAP1 | 9.50E-04 | -1.46 | Sepsis_D0_non-survivor down vs Sepsis_D0_survivor |
| FBXW2 | 8.40E-04 | -1.46 | Sepsis_D0_non-survivor down vs Sepsis_D0_survivor |
| ST7 | 6.74E-03 | -1.46 | Sepsis_D0_non-survivor down vs Sepsis_D0_survivor |
| SURF4 | 3.03E-03 | -1.46 | Sepsis_D0_non-survivor down vs Sepsis_D0_survivor |
| CMTM4 | 1.75E-04 | -1.46 | Sepsis_D0_non-survivor down vs Sepsis_D0_survivor |
| PMS1 | 1.04E-03 | -1.46 | Sepsis_D0_non-survivor down vs Sepsis_D0_survivor |
| THEM2 | 3.55E-03 | -1.46 | Sepsis_D0_non-survivor down vs Sepsis_D0_survivor |
| GLRX5 | 5.10E-03 | -1.46 | Sepsis_D0_non-survivor down vs Sepsis_D0_survivor |
| CDK5RAP2 | 6.87E-03 | -1.45 | Sepsis_D0_non-survivor down vs Sepsis_D0_survivor |
| PCMTD2 | 7.37E-03 | -1.45 | Sepsis_D0_non-survivor down vs Sepsis_D0_survivor |
| TM9SF2 | 1.37E-03 | -1.45 | Sepsis_D0_non-survivor down vs Sepsis_D0_survivor |
| ACAD8 | 6.95E-04 | -1.45 | Sepsis_D0_non-survivor down vs Sepsis_D0_survivor |
| PAIP1 | 2.46E-04 | -1.45 | Sepsis_D0_non-survivor down vs Sepsis_D0_survivor |
| SEC61G | 6.55E-03 | -1.45 | Sepsis_D0_non-survivor down vs Sepsis_D0_survivor |
| TMCO3 | 3.09E-03 | -1.45 | Sepsis_D0_non-survivor down vs Sepsis_D0_survivor |
| KIF1B | 4.70E-06 | -1.45 | Sepsis_D0_non-survivor down vs Sepsis_D0_survivor |
| BAG5 | 3.15E-03 | -1.45 | Sepsis_D0_non-survivor down vs Sepsis_D0_survivor |
| CD93 | 2.10E-03 | -1.45 | Sepsis_D0_non-survivor down vs Sepsis_D0_survivor |
| MRPS14 | 3.59E-03 | -1.45 | Sepsis_D0_non-survivor down vs Sepsis_D0_survivor |
| DSCR3 | 6.05E-03 | -1.45 | Sepsis_D0_non-survivor down vs Sepsis_D0_survivor |
| MTHFSD | 5.67E-03 | -1.45 | Sepsis_D0_non-survivor down vs Sepsis_D0_survivor |
| COQ5 | 2.57E-03 | -1.45 | Sepsis_D0_non-survivor down vs Sepsis_D0_survivor |
| TATDN1 | 1.56E-03 | -1.45 | Sepsis_D0_non-survivor down vs Sepsis_D0_survivor |
| EXT1 | 1.98E-03 | -1.45 | Sepsis_D0_non-survivor down vs Sepsis_D0_survivor |
| GTF2H5 | 3.52E-03 | -1.45 | Sepsis_D0_non-survivor down vs Sepsis_D0_survivor |
| RAP1A | 4.68E-03 | -1.45 | Sepsis_D0_non-survivor down vs Sepsis_D0_survivor |
| GNPAT | 5.18E-03 | -1.45 | Sepsis_D0_non-survivor down vs Sepsis_D0_survivor |
| RPA3 | 5.90E-03 | -1.45 | Sepsis_D0_non-survivor down vs Sepsis_D0_survivor |
| ZCCHC8 | 2.93E-03 | -1.45 | Sepsis_D0_non-survivor down vs Sepsis_D0_survivor |
| IPO7 | 3.44E-03 | -1.45 | Sepsis_D0_non-survivor down vs Sepsis_D0_survivor |
| STARD13 | 7.60E-06 | -1.45 | Sepsis_D0_non-survivor down vs Sepsis_D0_survivor |
| ATP5S | 2.77E-03 | -1.44 | Sepsis_D0_non-survivor down vs Sepsis_D0_survivor |
| CPEB4 | 2.28E-03 | -1.44 | Sepsis_D0_non-survivor down vs Sepsis_D0_survivor |
| COPB1 | 1.11E-03 | -1.44 | Sepsis_D0_non-survivor down vs Sepsis_D0_survivor |
| SLC35A2 | 3.29E-03 | -1.44 | Sepsis_D0_non-survivor down vs Sepsis_D0_survivor |
| ZDHHC19 | 8.89E-04 | -1.44 | Sepsis_D0_non-survivor down vs Sepsis_D0_survivor |
| ARHGEF6 | 8.58E-04 | -1.44 | Sepsis_D0_non-survivor down vs Sepsis_D0_survivor |
| GTDC1 | 3.67E-03 | -1.44 | Sepsis_D0_non-survivor down vs Sepsis_D0_survivor |
| TIAL1 | 4.34E-04 | -1.44 | Sepsis_D0_non-survivor down vs Sepsis_D0_survivor |
| IGFBP7 | 4.19E-03 | -1.44 | Sepsis_D0_non-survivor down vs Sepsis_D0_survivor |
| YME1L1 | 1.94E-03 | -1.44 | Sepsis_D0_non-survivor down vs Sepsis_D0_survivor |
| MRPS30 | 2.72E-03 | -1.44 | Sepsis_D0_non-survivor down vs Sepsis_D0_survivor |
| PHOSPHO2 | 4.27E-03 | -1.44 | Sepsis_D0_non-survivor down vs Sepsis_D0_survivor |
| HSA9761 | 6.87E-03 | -1.44 | Sepsis_D0_non-survivor down vs Sepsis_D0_survivor |
| HIGD1A | 1.66E-03 | -1.44 | Sepsis_D0_non-survivor down vs Sepsis_D0_survivor |
| GBAP | 2.14E-03 | -1.44 | Sepsis_D0_non-survivor down vs Sepsis_D0_survivor |
| TM2D2 | 4.44E-03 | -1.44 | Sepsis_D0_non-survivor down vs Sepsis_D0_survivor |
| SLC5A6 | 7.59E-03 | -1.44 | Sepsis_D0_non-survivor down vs Sepsis_D0_survivor |
| MRPL19 | 2.88E-03 | -1.44 | Sepsis_D0_non-survivor down vs Sepsis_D0_survivor |
| ANXA1 | 5.46E-03 | -1.44 | Sepsis_D0_non-survivor down vs Sepsis_D0_survivor |
| OMA1 | 5.06E-03 | -1.44 | Sepsis_D0_non-survivor down vs Sepsis_D0_survivor |
| LDHA | 1.27E-03 | -1.44 | Sepsis_D0_non-survivor down vs Sepsis_D0_survivor |
| NSFL1C | 4.07E-03 | -1.44 | Sepsis_D0_non-survivor down vs Sepsis_D0_survivor |
| SMNDC1 | 9.92E-05 | -1.43 | Sepsis_D0_non-survivor down vs Sepsis_D0_survivor |
| SNX5 | 3.35E-05 | -1.43 | Sepsis_D0_non-survivor down vs Sepsis_D0_survivor |
| NDUFA10 | 1.16E-03 | -1.43 | Sepsis_D0_non-survivor down vs Sepsis_D0_survivor |
| CCDC47 | 3.11E-03 | -1.43 | Sepsis_D0_non-survivor down vs Sepsis_D0_survivor |
| CRAMP1L | 3.09E-03 | -1.43 | Sepsis_D0_non-survivor down vs Sepsis_D0_survivor |
| NDUFA9 | 3.81E-03 | -1.43 | Sepsis_D0_non-survivor down vs Sepsis_D0_survivor |
| SETDB2 | 3.78E-03 | -1.43 | Sepsis_D0_non-survivor down vs Sepsis_D0_survivor |
| USP10 | 1.24E-03 | -1.43 | Sepsis_D0_non-survivor down vs Sepsis_D0_survivor |
| ZNF211 | 5.73E-03 | -1.43 | Sepsis_D0_non-survivor down vs Sepsis_D0_survivor |
| SARS2 | 6.53E-04 | -1.43 | Sepsis_D0_non-survivor down vs Sepsis_D0_survivor |
| JMJD1C | 5.65E-03 | -1.43 | Sepsis_D0_non-survivor down vs Sepsis_D0_survivor |
| CBARA1 | 6.62E-03 | -1.43 | Sepsis_D0_non-survivor down vs Sepsis_D0_survivor |
| HSPA14 | 1.07E-04 | -1.43 | Sepsis_D0_non-survivor down vs Sepsis_D0_survivor |
| SEPX1 | 4.42E-03 | -1.43 | Sepsis_D0_non-survivor down vs Sepsis_D0_survivor |
| SLC35B1 | 4.80E-03 | -1.43 | Sepsis_D0_non-survivor down vs Sepsis_D0_survivor |
| TCEA2 | 4.10E-03 | -1.43 | Sepsis_D0_non-survivor down vs Sepsis_D0_survivor |
| CD36 | 5.39E-03 | -1.43 | Sepsis_D0_non-survivor down vs Sepsis_D0_survivor |
| UBE3A | 1.67E-03 | -1.43 | Sepsis_D0_non-survivor down vs Sepsis_D0_survivor |
| MR1 | 3.47E-03 | -1.43 | Sepsis_D0_non-survivor down vs Sepsis_D0_survivor |
| TCF12 | 4.78E-03 | -1.43 | Sepsis_D0_non-survivor down vs Sepsis_D0_survivor |
| TOR1AIP1 | 7.33E-03 | -1.43 | Sepsis_D0_non-survivor down vs Sepsis_D0_survivor |
| STARD3 | 2.42E-03 | -1.43 | Sepsis_D0_non-survivor down vs Sepsis_D0_survivor |
| SERP1 | 2.87E-03 | -1.43 | Sepsis_D0_non-survivor down vs Sepsis_D0_survivor |
| MTMR6 | 2.53E-03 | -1.43 | Sepsis_D0_non-survivor down vs Sepsis_D0_survivor |
| FUT8 | 3.50E-03 | -1.42 | Sepsis_D0_non-survivor down vs Sepsis_D0_survivor |
| PSMA3 | 4.16E-03 | -1.42 | Sepsis_D0_non-survivor down vs Sepsis_D0_survivor |
| ATXN10 | 2.44E-03 | -1.42 | Sepsis_D0_non-survivor down vs Sepsis_D0_survivor |
| TXN | 2.74E-04 | -1.42 | Sepsis_D0_non-survivor down vs Sepsis_D0_survivor |
| MAPK1 | 1.02E-03 | -1.42 | Sepsis_D0_non-survivor down vs Sepsis_D0_survivor |
| LY96 | 6.80E-03 | -1.42 | Sepsis_D0_non-survivor down vs Sepsis_D0_survivor |
| COPA | 5.33E-03 | -1.42 | Sepsis_D0_non-survivor down vs Sepsis_D0_survivor |
| KCTD9 | 5.76E-03 | -1.42 | Sepsis_D0_non-survivor down vs Sepsis_D0_survivor |
| CWF19L1 | 4.35E-03 | -1.42 | Sepsis_D0_non-survivor down vs Sepsis_D0_survivor |
| COPS4 | 7.15E-04 | -1.42 | Sepsis_D0_non-survivor down vs Sepsis_D0_survivor |
| PPP2R5A | 1.37E-04 | -1.42 | Sepsis_D0_non-survivor down vs Sepsis_D0_survivor |
| MIER1 | 7.79E-04 | -1.42 | Sepsis_D0_non-survivor down vs Sepsis_D0_survivor |
| IL28RA | 4.07E-03 | -1.42 | Sepsis_D0_non-survivor down vs Sepsis_D0_survivor |
| UBE1DC1 | 4.65E-03 | -1.42 | Sepsis_D0_non-survivor down vs Sepsis_D0_survivor |
| BAT5 | 6.68E-03 | -1.42 | Sepsis_D0_non-survivor down vs Sepsis_D0_survivor |
| CCNH | 2.69E-03 | -1.42 | Sepsis_D0_non-survivor down vs Sepsis_D0_survivor |
| XPNPEP1 | 1.42E-03 | -1.42 | Sepsis_D0_non-survivor down vs Sepsis_D0_survivor |
| COG1 | 3.20E-03 | -1.42 | Sepsis_D0_non-survivor down vs Sepsis_D0_survivor |
| XPO6 | 7.01E-03 | -1.42 | Sepsis_D0_non-survivor down vs Sepsis_D0_survivor |
| FAM49B | 3.71E-03 | -1.42 | Sepsis_D0_non-survivor down vs Sepsis_D0_survivor |
| FLOT1 | 2.45E-03 | -1.42 | Sepsis_D0_non-survivor down vs Sepsis_D0_survivor |
| RPL4 | 7.11E-03 | -1.42 | Sepsis_D0_non-survivor down vs Sepsis_D0_survivor |
| NARS | 5.03E-03 | -1.41 | Sepsis_D0_non-survivor down vs Sepsis_D0_survivor |
| MIS12 | 3.43E-03 | -1.41 | Sepsis_D0_non-survivor down vs Sepsis_D0_survivor |
| ZNRF1 | 4.98E-03 | -1.41 | Sepsis_D0_non-survivor down vs Sepsis_D0_survivor |
| RAP2B | 1.34E-04 | -1.41 | Sepsis_D0_non-survivor down vs Sepsis_D0_survivor |
| NAT5 | 5.25E-03 | -1.41 | Sepsis_D0_non-survivor down vs Sepsis_D0_survivor |
| RAB6A | 6.22E-03 | -1.41 | Sepsis_D0_non-survivor down vs Sepsis_D0_survivor |
| MDP-1 | 1.52E-03 | -1.41 | Sepsis_D0_non-survivor down vs Sepsis_D0_survivor |
| GALNT1 | 5.40E-03 | -1.41 | Sepsis_D0_non-survivor down vs Sepsis_D0_survivor |
| STXBP3 | 2.15E-03 | -1.41 | Sepsis_D0_non-survivor down vs Sepsis_D0_survivor |
| ARL5A | 1.04E-03 | -1.41 | Sepsis_D0_non-survivor down vs Sepsis_D0_survivor |
| AARSD1 | 1.90E-04 | -1.41 | Sepsis_D0_non-survivor down vs Sepsis_D0_survivor |
| MLLT4 | 5.87E-03 | -1.41 | Sepsis_D0_non-survivor down vs Sepsis_D0_survivor |
| TSPYL1 | 4.08E-03 | -1.41 | Sepsis_D0_non-survivor down vs Sepsis_D0_survivor |
| FAM76B | 1.96E-03 | -1.41 | Sepsis_D0_non-survivor down vs Sepsis_D0_survivor |
| SLC25A16 | 7.90E-04 | -1.41 | Sepsis_D0_non-survivor down vs Sepsis_D0_survivor |
| SLC39A11 | 2.25E-03 | -1.41 | Sepsis_D0_non-survivor down vs Sepsis_D0_survivor |
| DYM | 3.87E-03 | -1.41 | Sepsis_D0_non-survivor down vs Sepsis_D0_survivor |
| CHSY1 | 2.19E-03 | -1.41 | Sepsis_D0_non-survivor down vs Sepsis_D0_survivor |
| METTL6 | 1.05E-03 | -1.41 | Sepsis_D0_non-survivor down vs Sepsis_D0_survivor |
| NIP7 | 4.41E-03 | -1.41 | Sepsis_D0_non-survivor down vs Sepsis_D0_survivor |
| DR1 | 7.35E-03 | -1.41 | Sepsis_D0_non-survivor down vs Sepsis_D0_survivor |
| ATP5F1 | 7.90E-07 | -1.41 | Sepsis_D0_non-survivor down vs Sepsis_D0_survivor |
| MRPL42 | 6.57E-04 | -1.40 | Sepsis_D0_non-survivor down vs Sepsis_D0_survivor |
| BPNT1 | 2.56E-04 | -1.40 | Sepsis_D0_non-survivor down vs Sepsis_D0_survivor |
| DNAJC14 | 2.75E-03 | -1.40 | Sepsis_D0_non-survivor down vs Sepsis_D0_survivor |
| FBXO9 | 3.94E-06 | -1.40 | Sepsis_D0_non-survivor down vs Sepsis_D0_survivor |
| GPR1 | 3.94E-03 | -1.40 | Sepsis_D0_non-survivor down vs Sepsis_D0_survivor |
| PACS2 | 7.22E-03 | -1.40 | Sepsis_D0_non-survivor down vs Sepsis_D0_survivor |
| SUCLG1 | 1.51E-03 | -1.40 | Sepsis_D0_non-survivor down vs Sepsis_D0_survivor |
| PJA2 | 5.68E-03 | -1.40 | Sepsis_D0_non-survivor down vs Sepsis_D0_survivor |
| PPARA | 7.51E-04 | -1.40 | Sepsis_D0_non-survivor down vs Sepsis_D0_survivor |
| ABI2 | 2.01E-03 | -1.40 | Sepsis_D0_non-survivor down vs Sepsis_D0_survivor |
| ATG4B | 2.04E-03 | -1.40 | Sepsis_D0_non-survivor down vs Sepsis_D0_survivor |
| STAU1 | 3.68E-03 | -1.40 | Sepsis_D0_non-survivor down vs Sepsis_D0_survivor |
| PDZD11 | 2.88E-03 | -1.40 | Sepsis_D0_non-survivor down vs Sepsis_D0_survivor |
| SLC35A3 | 3.60E-03 | -1.40 | Sepsis_D0_non-survivor down vs Sepsis_D0_survivor |
| S100A12 | 6.03E-03 | -1.40 | Sepsis_D0_non-survivor down vs Sepsis_D0_survivor |
| MED6 | 7.43E-03 | -1.40 | Sepsis_D0_non-survivor down vs Sepsis_D0_survivor |
| NEK3 | 4.58E-03 | -1.40 | Sepsis_D0_non-survivor down vs Sepsis_D0_survivor |
| LRRC8A | 5.08E-03 | -1.40 | Sepsis_D0_non-survivor down vs Sepsis_D0_survivor |
| PSMD6 | 3.63E-03 | -1.39 | Sepsis_D0_non-survivor down vs Sepsis_D0_survivor |
| TFDP1 | 5.04E-03 | -1.39 | Sepsis_D0_non-survivor down vs Sepsis_D0_survivor |
| USP14 | 2.95E-03 | -1.39 | Sepsis_D0_non-survivor down vs Sepsis_D0_survivor |
| G3BP2 | 1.81E-03 | -1.39 | Sepsis_D0_non-survivor down vs Sepsis_D0_survivor |
| EFTUD1 | 4.35E-03 | -1.39 | Sepsis_D0_non-survivor down vs Sepsis_D0_survivor |
| BST1 | 3.73E-03 | -1.39 | Sepsis_D0_non-survivor down vs Sepsis_D0_survivor |
| HINT1 | 1.61E-03 | -1.39 | Sepsis_D0_non-survivor down vs Sepsis_D0_survivor |
| TXNL1 | 6.12E-03 | -1.39 | Sepsis_D0_non-survivor down vs Sepsis_D0_survivor |
| EED | 6.89E-03 | -1.39 | Sepsis_D0_non-survivor down vs Sepsis_D0_survivor |
| PCGF3 | 5.34E-03 | -1.39 | Sepsis_D0_non-survivor down vs Sepsis_D0_survivor |
| RPL8 | 2.48E-03 | -1.39 | Sepsis_D0_non-survivor down vs Sepsis_D0_survivor |
| UFC1 | 1.28E-03 | -1.39 | Sepsis_D0_non-survivor down vs Sepsis_D0_survivor |
| LONPL | 6.59E-03 | -1.39 | Sepsis_D0_non-survivor down vs Sepsis_D0_survivor |
| NGRN | 8.40E-04 | -1.39 | Sepsis_D0_non-survivor down vs Sepsis_D0_survivor |
| ST6GALNAC2 | 1.13E-03 | -1.39 | Sepsis_D0_non-survivor down vs Sepsis_D0_survivor |
| PAPD1 | 6.96E-03 | -1.39 | Sepsis_D0_non-survivor down vs Sepsis_D0_survivor |
| PDCD5 | 8.18E-04 | -1.39 | Sepsis_D0_non-survivor down vs Sepsis_D0_survivor |
| GTF2I | 4.61E-03 | -1.39 | Sepsis_D0_non-survivor down vs Sepsis_D0_survivor |
| MRPL50 | 8.86E-04 | -1.39 | Sepsis_D0_non-survivor down vs Sepsis_D0_survivor |
| PARK7 | 6.92E-03 | -1.39 | Sepsis_D0_non-survivor down vs Sepsis_D0_survivor |
| ANKRD26 | 6.73E-03 | -1.39 | Sepsis_D0_non-survivor down vs Sepsis_D0_survivor |
| OSTF1 | 3.01E-05 | -1.38 | Sepsis_D0_non-survivor down vs Sepsis_D0_survivor |
| ARHGAP12 | 3.32E-03 | -1.38 | Sepsis_D0_non-survivor down vs Sepsis_D0_survivor |
| SSBP1 | 2.70E-03 | -1.38 | Sepsis_D0_non-survivor down vs Sepsis_D0_survivor |
| AFARP1 | 4.69E-03 | -1.38 | Sepsis_D0_non-survivor down vs Sepsis_D0_survivor |
| NF2 | 3.76E-03 | -1.38 | Sepsis_D0_non-survivor down vs Sepsis_D0_survivor |
| PTBP1 | 5.56E-03 | -1.38 | Sepsis_D0_non-survivor down vs Sepsis_D0_survivor |
| PSMD12 | 7.31E-05 | -1.38 | Sepsis_D0_non-survivor down vs Sepsis_D0_survivor |
| RNF13 | 3.95E-03 | -1.38 | Sepsis_D0_non-survivor down vs Sepsis_D0_survivor |
| MRPS5 | 1.40E-03 | -1.38 | Sepsis_D0_non-survivor down vs Sepsis_D0_survivor |
| RNGTT | 2.06E-03 | -1.38 | Sepsis_D0_non-survivor down vs Sepsis_D0_survivor |
| ARHGEF17 | 2.31E-03 | -1.38 | Sepsis_D0_non-survivor down vs Sepsis_D0_survivor |
| TMEM101 | 4.01E-03 | -1.38 | Sepsis_D0_non-survivor down vs Sepsis_D0_survivor |
| SPPL2A | 2.92E-03 | -1.38 | Sepsis_D0_non-survivor down vs Sepsis_D0_survivor |
| KLHDC1 | 6.49E-03 | -1.38 | Sepsis_D0_non-survivor down vs Sepsis_D0_survivor |
| GALNT2 | 2.03E-03 | -1.38 | Sepsis_D0_non-survivor down vs Sepsis_D0_survivor |
| LAMP2 | 4.04E-03 | -1.38 | Sepsis_D0_non-survivor down vs Sepsis_D0_survivor |
| GLT28D1 | 5.63E-03 | -1.38 | Sepsis_D0_non-survivor down vs Sepsis_D0_survivor |
| ABCB10 | 4.54E-03 | -1.38 | Sepsis_D0_non-survivor down vs Sepsis_D0_survivor |
| PCCB | 2.13E-03 | -1.38 | Sepsis_D0_non-survivor down vs Sepsis_D0_survivor |
| POMP | 1.08E-03 | -1.38 | Sepsis_D0_non-survivor down vs Sepsis_D0_survivor |
| NDUFA8 | 7.50E-03 | -1.37 | Sepsis_D0_non-survivor down vs Sepsis_D0_survivor |
| METTL2B | 5.39E-03 | -1.37 | Sepsis_D0_non-survivor down vs Sepsis_D0_survivor |
| DBNL | 2.53E-03 | -1.37 | Sepsis_D0_non-survivor down vs Sepsis_D0_survivor |
| CYCS | 6.46E-03 | -1.37 | Sepsis_D0_non-survivor down vs Sepsis_D0_survivor |
| PDHB | 6.71E-04 | -1.37 | Sepsis_D0_non-survivor down vs Sepsis_D0_survivor |
| CNOT6 | 4.15E-03 | -1.37 | Sepsis_D0_non-survivor down vs Sepsis_D0_survivor |
| NDUFC2 | 2.98E-03 | -1.37 | Sepsis_D0_non-survivor down vs Sepsis_D0_survivor |
| CCDC25 | 1.33E-03 | -1.37 | Sepsis_D0_non-survivor down vs Sepsis_D0_survivor |
| FH | 3.49E-04 | -1.37 | Sepsis_D0_non-survivor down vs Sepsis_D0_survivor |
| HIST1H2AG | 5.86E-03 | -1.37 | Sepsis_D0_non-survivor down vs Sepsis_D0_survivor |
| MARK1 | 2.93E-03 | -1.37 | Sepsis_D0_non-survivor down vs Sepsis_D0_survivor |
| RUFY3 | 5.18E-04 | -1.37 | Sepsis_D0_non-survivor down vs Sepsis_D0_survivor |
| KATNAL1 | 1.46E-03 | -1.37 | Sepsis_D0_non-survivor down vs Sepsis_D0_survivor |
| CTAGE5 | 1.19E-03 | -1.37 | Sepsis_D0_non-survivor down vs Sepsis_D0_survivor |
| GRPEL1 | 2.64E-03 | -1.36 | Sepsis_D0_non-survivor down vs Sepsis_D0_survivor |
| PPAPDC1A | 4.93E-03 | -1.36 | Sepsis_D0_non-survivor down vs Sepsis_D0_survivor |
| TMEM1 | 6.94E-03 | -1.36 | Sepsis_D0_non-survivor down vs Sepsis_D0_survivor |
| BRPF3 | 6.48E-03 | -1.36 | Sepsis_D0_non-survivor down vs Sepsis_D0_survivor |
| FADS3 | 6.24E-04 | -1.36 | Sepsis_D0_non-survivor down vs Sepsis_D0_survivor |
| RY1 | 7.49E-03 | -1.36 | Sepsis_D0_non-survivor down vs Sepsis_D0_survivor |
| NUDT21 | 2.85E-03 | -1.36 | Sepsis_D0_non-survivor down vs Sepsis_D0_survivor |
| HDDC3 | 3.58E-04 | -1.36 | Sepsis_D0_non-survivor down vs Sepsis_D0_survivor |
| ADD3 | 4.78E-03 | -1.36 | Sepsis_D0_non-survivor down vs Sepsis_D0_survivor |
| MKLN1 | 1.60E-04 | -1.36 | Sepsis_D0_non-survivor down vs Sepsis_D0_survivor |
| WRNIP1 | 2.95E-03 | -1.36 | Sepsis_D0_non-survivor down vs Sepsis_D0_survivor |
| LSM14B | 2.42E-04 | -1.36 | Sepsis_D0_non-survivor down vs Sepsis_D0_survivor |
| BNIP2 | 2.86E-04 | -1.36 | Sepsis_D0_non-survivor down vs Sepsis_D0_survivor |
| PSPC1 | 8.20E-04 | -1.36 | Sepsis_D0_non-survivor down vs Sepsis_D0_survivor |
| ETFDH | 3.71E-03 | -1.35 | Sepsis_D0_non-survivor down vs Sepsis_D0_survivor |
| MAPKAP1 | 2.21E-03 | -1.35 | Sepsis_D0_non-survivor down vs Sepsis_D0_survivor |
| GLIPR1 | 6.02E-03 | -1.35 | Sepsis_D0_non-survivor down vs Sepsis_D0_survivor |
| EIF2A | 2.24E-03 | -1.35 | Sepsis_D0_non-survivor down vs Sepsis_D0_survivor |
| DDX46 | 1.32E-03 | -1.35 | Sepsis_D0_non-survivor down vs Sepsis_D0_survivor |
| SCC-112 | 1.20E-03 | -1.35 | Sepsis_D0_non-survivor down vs Sepsis_D0_survivor |
| ANKRA2 | 2.69E-04 | -1.35 | Sepsis_D0_non-survivor down vs Sepsis_D0_survivor |
| NGLY1 | 5.36E-03 | -1.35 | Sepsis_D0_non-survivor down vs Sepsis_D0_survivor |
| DNAH14 | 1.46E-03 | -1.35 | Sepsis_D0_non-survivor down vs Sepsis_D0_survivor |
| HIP2 | 1.62E-03 | -1.35 | Sepsis_D0_non-survivor down vs Sepsis_D0_survivor |
| ERCC3 | 4.98E-03 | -1.35 | Sepsis_D0_non-survivor down vs Sepsis_D0_survivor |
| CSRP1 | 6.43E-03 | -1.35 | Sepsis_D0_non-survivor down vs Sepsis_D0_survivor |
| NCKAP1L | 1.57E-04 | -1.35 | Sepsis_D0_non-survivor down vs Sepsis_D0_survivor |
| VDAC3 | 8.49E-04 | -1.35 | Sepsis_D0_non-survivor down vs Sepsis_D0_survivor |
| UBL3 | 1.45E-03 | -1.35 | Sepsis_D0_non-survivor down vs Sepsis_D0_survivor |
| NCOR1 | 6.21E-03 | -1.35 | Sepsis_D0_non-survivor down vs Sepsis_D0_survivor |
| COPS8 | 3.30E-04 | -1.35 | Sepsis_D0_non-survivor down vs Sepsis_D0_survivor |
| PITRM1 | 6.58E-03 | -1.35 | Sepsis_D0_non-survivor down vs Sepsis_D0_survivor |
| ARF4 | 2.00E-03 | -1.35 | Sepsis_D0_non-survivor down vs Sepsis_D0_survivor |
| LPIN2 | 2.24E-03 | -1.35 | Sepsis_D0_non-survivor down vs Sepsis_D0_survivor |
| GPKOW | 4.93E-03 | -1.35 | Sepsis_D0_non-survivor down vs Sepsis_D0_survivor |
| SERINC3 | 6.33E-03 | -1.35 | Sepsis_D0_non-survivor down vs Sepsis_D0_survivor |
| RAE1 | 7.08E-06 | -1.35 | Sepsis_D0_non-survivor down vs Sepsis_D0_survivor |
| CCDC111 | 4.10E-04 | -1.35 | Sepsis_D0_non-survivor down vs Sepsis_D0_survivor |
| GMPR2 | 2.03E-03 | -1.34 | Sepsis_D0_non-survivor down vs Sepsis_D0_survivor |
| PDHA1 | 4.50E-03 | -1.34 | Sepsis_D0_non-survivor down vs Sepsis_D0_survivor |
| ATP5J | 1.74E-03 | -1.34 | Sepsis_D0_non-survivor down vs Sepsis_D0_survivor |
| CDK5RAP1 | 3.07E-03 | -1.34 | Sepsis_D0_non-survivor down vs Sepsis_D0_survivor |
| KLHL2 | 6.76E-03 | -1.34 | Sepsis_D0_non-survivor down vs Sepsis_D0_survivor |
| MPDZ | 4.46E-04 | -1.34 | Sepsis_D0_non-survivor down vs Sepsis_D0_survivor |
| UCHL5 | 1.55E-03 | -1.34 | Sepsis_D0_non-survivor down vs Sepsis_D0_survivor |
| ZNF720 | 1.40E-03 | -1.34 | Sepsis_D0_non-survivor down vs Sepsis_D0_survivor |
| AEBP2 | 7.66E-04 | -1.34 | Sepsis_D0_non-survivor down vs Sepsis_D0_survivor |
| CCDC52 | 1.12E-04 | -1.34 | Sepsis_D0_non-survivor down vs Sepsis_D0_survivor |
| PMPCB | 2.49E-03 | -1.34 | Sepsis_D0_non-survivor down vs Sepsis_D0_survivor |
| ATP2C1 | 1.86E-03 | -1.34 | Sepsis_D0_non-survivor down vs Sepsis_D0_survivor |
| PXMP3 | 1.32E-03 | -1.34 | Sepsis_D0_non-survivor down vs Sepsis_D0_survivor |
| COMT | 1.93E-04 | -1.34 | Sepsis_D0_non-survivor down vs Sepsis_D0_survivor |
| PARN | 1.23E-03 | -1.34 | Sepsis_D0_non-survivor down vs Sepsis_D0_survivor |
| PSMC2 | 3.84E-03 | -1.34 | Sepsis_D0_non-survivor down vs Sepsis_D0_survivor |
| ZNF37A | 4.82E-03 | -1.33 | Sepsis_D0_non-survivor down vs Sepsis_D0_survivor |
| CTNNBL1 | 7.10E-05 | -1.33 | Sepsis_D0_non-survivor down vs Sepsis_D0_survivor |
| HADHB | 3.99E-04 | -1.33 | Sepsis_D0_non-survivor down vs Sepsis_D0_survivor |
| TMEM43 | 1.04E-03 | -1.33 | Sepsis_D0_non-survivor down vs Sepsis_D0_survivor |
| PRMT2 | 2.23E-03 | -1.33 | Sepsis_D0_non-survivor down vs Sepsis_D0_survivor |
| ZFAND5 | 5.96E-03 | -1.33 | Sepsis_D0_non-survivor down vs Sepsis_D0_survivor |
| RPS6KA4 | 1.51E-03 | -1.33 | Sepsis_D0_non-survivor down vs Sepsis_D0_survivor |
| COX5B | 9.17E-04 | -1.33 | Sepsis_D0_non-survivor down vs Sepsis_D0_survivor |
| TTC32 | 5.54E-04 | -1.33 | Sepsis_D0_non-survivor down vs Sepsis_D0_survivor |
| DCP2 | 3.41E-03 | -1.33 | Sepsis_D0_non-survivor down vs Sepsis_D0_survivor |
| CPSF6 | 9.63E-04 | -1.33 | Sepsis_D0_non-survivor down vs Sepsis_D0_survivor |
| SKP1A | 8.72E-05 | -1.33 | Sepsis_D0_non-survivor down vs Sepsis_D0_survivor |
| PPM1B | 7.28E-04 | -1.33 | Sepsis_D0_non-survivor down vs Sepsis_D0_survivor |
| PMS2 | 1.22E-03 | -1.33 | Sepsis_D0_non-survivor down vs Sepsis_D0_survivor |
| ECH1 | 7.60E-03 | -1.33 | Sepsis_D0_non-survivor down vs Sepsis_D0_survivor |
| TTC17 | 2.77E-03 | -1.32 | Sepsis_D0_non-survivor down vs Sepsis_D0_survivor |
| SMC6 | 5.45E-03 | -1.32 | Sepsis_D0_non-survivor down vs Sepsis_D0_survivor |
| PIGW | 5.32E-03 | -1.32 | Sepsis_D0_non-survivor down vs Sepsis_D0_survivor |
| CYB5R4 | 1.84E-03 | -1.32 | Sepsis_D0_non-survivor down vs Sepsis_D0_survivor |
| PDCD6IP | 6.26E-03 | -1.32 | Sepsis_D0_non-survivor down vs Sepsis_D0_survivor |
| CDC16 | 5.55E-04 | -1.32 | Sepsis_D0_non-survivor down vs Sepsis_D0_survivor |
| PDHX | 7.23E-03 | -1.32 | Sepsis_D0_non-survivor down vs Sepsis_D0_survivor |
| MRLC2 | 4.53E-03 | -1.32 | Sepsis_D0_non-survivor down vs Sepsis_D0_survivor |
| PRDX1 | 1.90E-03 | -1.32 | Sepsis_D0_non-survivor down vs Sepsis_D0_survivor |
| LUC7L2 | 6.07E-03 | -1.32 | Sepsis_D0_non-survivor down vs Sepsis_D0_survivor |
| GPD1L | 4.17E-04 | -1.32 | Sepsis_D0_non-survivor down vs Sepsis_D0_survivor |
| GAPVD1 | 4.18E-03 | -1.32 | Sepsis_D0_non-survivor down vs Sepsis_D0_survivor |
| PSMA1 | 1.08E-04 | -1.32 | Sepsis_D0_non-survivor down vs Sepsis_D0_survivor |
| NIN | 7.58E-03 | -1.32 | Sepsis_D0_non-survivor down vs Sepsis_D0_survivor |
| ORAOV1 | 2.04E-03 | -1.31 | Sepsis_D0_non-survivor down vs Sepsis_D0_survivor |
| PTEN | 5.09E-03 | -1.31 | Sepsis_D0_non-survivor down vs Sepsis_D0_survivor |
| KIF9 | 4.83E-03 | -1.31 | Sepsis_D0_non-survivor down vs Sepsis_D0_survivor |
| PRR12 | 2.19E-03 | -1.31 | Sepsis_D0_non-survivor down vs Sepsis_D0_survivor |
| PPP1R7 | 5.13E-03 | -1.31 | Sepsis_D0_non-survivor down vs Sepsis_D0_survivor |
| DERL2 | 4.17E-03 | -1.31 | Sepsis_D0_non-survivor down vs Sepsis_D0_survivor |
| RB1CC1 | 1.01E-03 | -1.31 | Sepsis_D0_non-survivor down vs Sepsis_D0_survivor |
| OSBPL2 | 2.62E-03 | -1.31 | Sepsis_D0_non-survivor down vs Sepsis_D0_survivor |
| PRLR | 4.59E-03 | -1.31 | Sepsis_D0_non-survivor down vs Sepsis_D0_survivor |
| GUSB | 4.07E-03 | -1.31 | Sepsis_D0_non-survivor down vs Sepsis_D0_survivor |
| GHITM | 2.01E-03 | -1.31 | Sepsis_D0_non-survivor down vs Sepsis_D0_survivor |
| UBE2F | 4.62E-03 | -1.31 | Sepsis_D0_non-survivor down vs Sepsis_D0_survivor |
| CPNE3 | 3.79E-04 | -1.31 | Sepsis_D0_non-survivor down vs Sepsis_D0_survivor |
| LRRN6A | 6.99E-03 | -1.31 | Sepsis_D0_non-survivor down vs Sepsis_D0_survivor |
| APLP2 | 5.10E-03 | -1.31 | Sepsis_D0_non-survivor down vs Sepsis_D0_survivor |
| DDX5 | 7.01E-03 | -1.31 | Sepsis_D0_non-survivor down vs Sepsis_D0_survivor |
| PPTC7 | 1.60E-03 | -1.31 | Sepsis_D0_non-survivor down vs Sepsis_D0_survivor |
| ZNF364 | 5.72E-03 | -1.31 | Sepsis_D0_non-survivor down vs Sepsis_D0_survivor |
| CDC26 | 3.13E-03 | -1.30 | Sepsis_D0_non-survivor down vs Sepsis_D0_survivor |
| MAPRE1 | 2.35E-03 | -1.30 | Sepsis_D0_non-survivor down vs Sepsis_D0_survivor |
| MAP3K7 | 5.61E-03 | -1.30 | Sepsis_D0_non-survivor down vs Sepsis_D0_survivor |
| CDH26 | 3.55E-03 | -1.30 | Sepsis_D0_non-survivor down vs Sepsis_D0_survivor |
| ITGB3BP | 5.13E-04 | -1.30 | Sepsis_D0_non-survivor down vs Sepsis_D0_survivor |
| TXNL4A | 6.38E-03 | -1.30 | Sepsis_D0_non-survivor down vs Sepsis_D0_survivor |
| ALG6 | 1.30E-03 | -1.30 | Sepsis_D0_non-survivor down vs Sepsis_D0_survivor |
| PRMT5 | 7.19E-03 | -1.30 | Sepsis_D0_non-survivor down vs Sepsis_D0_survivor |
| DNASE1L1 | 6.91E-03 | -1.30 | Sepsis_D0_non-survivor down vs Sepsis_D0_survivor |
| ZFYVE20 | 8.31E-04 | -1.30 | Sepsis_D0_non-survivor down vs Sepsis_D0_survivor |
| EPS8L1 | 5.15E-03 | -1.30 | Sepsis_D0_non-survivor down vs Sepsis_D0_survivor |
| DDHD2 | 5.29E-03 | -1.30 | Sepsis_D0_non-survivor down vs Sepsis_D0_survivor |
| LARS | 4.13E-03 | -1.30 | Sepsis_D0_non-survivor down vs Sepsis_D0_survivor |
| PPIE | 2.19E-03 | -1.30 | Sepsis_D0_non-survivor down vs Sepsis_D0_survivor |
| MRPS22 | 6.07E-03 | -1.30 | Sepsis_D0_non-survivor down vs Sepsis_D0_survivor |
| TSN | 6.72E-03 | -1.30 | Sepsis_D0_non-survivor down vs Sepsis_D0_survivor |
| ATP11A | 7.11E-03 | -1.29 | Sepsis_D0_non-survivor down vs Sepsis_D0_survivor |
| TIPRL | 4.63E-04 | -1.29 | Sepsis_D0_non-survivor down vs Sepsis_D0_survivor |
| ASH2L | 5.81E-03 | -1.29 | Sepsis_D0_non-survivor down vs Sepsis_D0_survivor |
| PIGS | 5.86E-03 | -1.29 | Sepsis_D0_non-survivor down vs Sepsis_D0_survivor |
| PDE1C | 4.82E-03 | -1.29 | Sepsis_D0_non-survivor down vs Sepsis_D0_survivor |
| MRPL47 | 1.54E-03 | -1.29 | Sepsis_D0_non-survivor down vs Sepsis_D0_survivor |
| ZNF542 | 2.39E-03 | -1.29 | Sepsis_D0_non-survivor down vs Sepsis_D0_survivor |
| OCIAD1 | 4.14E-04 | -1.29 | Sepsis_D0_non-survivor down vs Sepsis_D0_survivor |
| POP4 | 7.40E-04 | -1.29 | Sepsis_D0_non-survivor down vs Sepsis_D0_survivor |
| RNF170 | 4.84E-03 | -1.29 | Sepsis_D0_non-survivor down vs Sepsis_D0_survivor |
| MBTPS1 | 1.14E-04 | -1.29 | Sepsis_D0_non-survivor down vs Sepsis_D0_survivor |
| DCTD | 2.93E-03 | -1.29 | Sepsis_D0_non-survivor down vs Sepsis_D0_survivor |
| SEC61B | 3.96E-03 | -1.29 | Sepsis_D0_non-survivor down vs Sepsis_D0_survivor |
| TNFRSF19 | 5.81E-03 | -1.29 | Sepsis_D0_non-survivor down vs Sepsis_D0_survivor |
| ERCC6 | 6.37E-03 | -1.28 | Sepsis_D0_non-survivor down vs Sepsis_D0_survivor |
| PIGY | 1.66E-03 | -1.28 | Sepsis_D0_non-survivor down vs Sepsis_D0_survivor |
| RPS24 | 4.49E-03 | -1.28 | Sepsis_D0_non-survivor down vs Sepsis_D0_survivor |
| CRSP3 | 6.71E-03 | -1.28 | Sepsis_D0_non-survivor down vs Sepsis_D0_survivor |
| FBXO42 | 1.01E-04 | -1.28 | Sepsis_D0_non-survivor down vs Sepsis_D0_survivor |
| COX7A2 | 9.55E-05 | -1.28 | Sepsis_D0_non-survivor down vs Sepsis_D0_survivor |
| PHF11 | 1.09E-03 | -1.28 | Sepsis_D0_non-survivor down vs Sepsis_D0_survivor |
| VDAC2 | 5.68E-03 | -1.28 | Sepsis_D0_non-survivor down vs Sepsis_D0_survivor |
| ARHGAP4 | 7.02E-03 | -1.28 | Sepsis_D0_non-survivor down vs Sepsis_D0_survivor |
| SNRPG | 1.88E-03 | -1.28 | Sepsis_D0_non-survivor down vs Sepsis_D0_survivor |
| FAM20B | 6.45E-04 | -1.28 | Sepsis_D0_non-survivor down vs Sepsis_D0_survivor |
| NOP17 | 1.04E-03 | -1.28 | Sepsis_D0_non-survivor down vs Sepsis_D0_survivor |
| LARP2 | 4.95E-03 | -1.28 | Sepsis_D0_non-survivor down vs Sepsis_D0_survivor |
| SLC25A5 | 5.45E-04 | -1.28 | Sepsis_D0_non-survivor down vs Sepsis_D0_survivor |
| OTOR | 1.66E-03 | -1.28 | Sepsis_D0_non-survivor down vs Sepsis_D0_survivor |
| VAPA | 2.13E-03 | -1.27 | Sepsis_D0_non-survivor down vs Sepsis_D0_survivor |
| RANBP9 | 5.50E-03 | -1.27 | Sepsis_D0_non-survivor down vs Sepsis_D0_survivor |
| ZNF161 | 1.78E-03 | -1.27 | Sepsis_D0_non-survivor down vs Sepsis_D0_survivor |
| MYOHD1 | 4.54E-03 | -1.27 | Sepsis_D0_non-survivor down vs Sepsis_D0_survivor |
| BLOC1S1 | 2.19E-04 | -1.27 | Sepsis_D0_non-survivor down vs Sepsis_D0_survivor |
| XPA | 4.88E-03 | -1.27 | Sepsis_D0_non-survivor down vs Sepsis_D0_survivor |
| USP48 | 6.61E-03 | -1.27 | Sepsis_D0_non-survivor down vs Sepsis_D0_survivor |
| USP39 | 6.32E-03 | -1.27 | Sepsis_D0_non-survivor down vs Sepsis_D0_survivor |
| ARCN1 | 6.23E-03 | -1.27 | Sepsis_D0_non-survivor down vs Sepsis_D0_survivor |
| ROCK1 | 1.49E-03 | -1.27 | Sepsis_D0_non-survivor down vs Sepsis_D0_survivor |
| FUSIP1 | 1.01E-03 | -1.27 | Sepsis_D0_non-survivor down vs Sepsis_D0_survivor |
| CLASP1 | 1.48E-03 | -1.27 | Sepsis_D0_non-survivor down vs Sepsis_D0_survivor |
| FAM98C | 7.38E-03 | -1.27 | Sepsis_D0_non-survivor down vs Sepsis_D0_survivor |
| TAF9 | 1.38E-03 | -1.27 | Sepsis_D0_non-survivor down vs Sepsis_D0_survivor |
| RPS27L | 1.61E-03 | -1.27 | Sepsis_D0_non-survivor down vs Sepsis_D0_survivor |
| COX5A | 6.79E-04 | -1.26 | Sepsis_D0_non-survivor down vs Sepsis_D0_survivor |
| UBE3C | 2.61E-04 | -1.26 | Sepsis_D0_non-survivor down vs Sepsis_D0_survivor |
| GAPDH | 9.56E-04 | -1.26 | Sepsis_D0_non-survivor down vs Sepsis_D0_survivor |
| CLYBL | 2.01E-03 | -1.26 | Sepsis_D0_non-survivor down vs Sepsis_D0_survivor |
| ALG8 | 3.17E-03 | -1.26 | Sepsis_D0_non-survivor down vs Sepsis_D0_survivor |
| RAC1 | 6.34E-03 | -1.26 | Sepsis_D0_non-survivor down vs Sepsis_D0_survivor |
| CDC42 | 2.13E-03 | -1.26 | Sepsis_D0_non-survivor down vs Sepsis_D0_survivor |
| IREB2 | 7.50E-03 | -1.26 | Sepsis_D0_non-survivor down vs Sepsis_D0_survivor |
| PITPNA | 7.15E-03 | -1.26 | Sepsis_D0_non-survivor down vs Sepsis_D0_survivor |
| SLU7 | 4.56E-03 | -1.26 | Sepsis_D0_non-survivor down vs Sepsis_D0_survivor |
| ARPC1A | 1.79E-03 | -1.26 | Sepsis_D0_non-survivor down vs Sepsis_D0_survivor |
| MRPL9 | 1.59E-03 | -1.25 | Sepsis_D0_non-survivor down vs Sepsis_D0_survivor |
| ZC3H8 | 2.87E-03 | -1.25 | Sepsis_D0_non-survivor down vs Sepsis_D0_survivor |
| SNRPB2 | 4.96E-03 | -1.25 | Sepsis_D0_non-survivor down vs Sepsis_D0_survivor |
| MKI67IP | 3.03E-04 | -1.25 | Sepsis_D0_non-survivor down vs Sepsis_D0_survivor |
| CAMTA1 | 5.87E-03 | -1.25 | Sepsis_D0_non-survivor down vs Sepsis_D0_survivor |
| SLC25A3 | 3.16E-03 | -1.25 | Sepsis_D0_non-survivor down vs Sepsis_D0_survivor |
| COMMD1 | 1.35E-04 | -1.25 | Sepsis_D0_non-survivor down vs Sepsis_D0_survivor |
| ZNF451 | 6.66E-03 | -1.25 | Sepsis_D0_non-survivor down vs Sepsis_D0_survivor |
| RPS21 | 3.14E-03 | -1.25 | Sepsis_D0_non-survivor down vs Sepsis_D0_survivor |
| SUB1 | 2.37E-03 | -1.25 | Sepsis_D0_non-survivor down vs Sepsis_D0_survivor |
| MYEOV2 | 6.36E-03 | -1.25 | Sepsis_D0_non-survivor down vs Sepsis_D0_survivor |
| ZNF480 | 1.92E-03 | -1.25 | Sepsis_D0_non-survivor down vs Sepsis_D0_survivor |
| NAP1L4 | 5.97E-03 | -1.25 | Sepsis_D0_non-survivor down vs Sepsis_D0_survivor |
| FRAP1 | 8.84E-04 | -1.25 | Sepsis_D0_non-survivor down vs Sepsis_D0_survivor |
| PPP3CB | 6.72E-03 | -1.25 | Sepsis_D0_non-survivor down vs Sepsis_D0_survivor |
| EDEM2 | 4.21E-03 | -1.24 | Sepsis_D0_non-survivor down vs Sepsis_D0_survivor |
| PHF8 | 6.32E-04 | -1.24 | Sepsis_D0_non-survivor down vs Sepsis_D0_survivor |
| COX7C | 9.42E-04 | -1.24 | Sepsis_D0_non-survivor down vs Sepsis_D0_survivor |
| UBE4A | 3.92E-03 | -1.24 | Sepsis_D0_non-survivor down vs Sepsis_D0_survivor |
| TITF1 | 4.09E-03 | -1.24 | Sepsis_D0_non-survivor down vs Sepsis_D0_survivor |
| RPS15 | 1.92E-03 | -1.24 | Sepsis_D0_non-survivor down vs Sepsis_D0_survivor |
| ENY2 | 6.11E-03 | -1.24 | Sepsis_D0_non-survivor down vs Sepsis_D0_survivor |
| CSDE1 | 8.80E-04 | -1.24 | Sepsis_D0_non-survivor down vs Sepsis_D0_survivor |
| RPL37A | 6.71E-03 | -1.24 | Sepsis_D0_non-survivor down vs Sepsis_D0_survivor |
| DNAJB12 | 6.94E-03 | -1.23 | Sepsis_D0_non-survivor down vs Sepsis_D0_survivor |
| SNX13 | 2.13E-03 | -1.23 | Sepsis_D0_non-survivor down vs Sepsis_D0_survivor |
| BZW1 | 8.91E-04 | -1.23 | Sepsis_D0_non-survivor down vs Sepsis_D0_survivor |
| PHF20L1 | 3.23E-03 | -1.23 | Sepsis_D0_non-survivor down vs Sepsis_D0_survivor |
| ZBTB11 | 5.23E-03 | -1.23 | Sepsis_D0_non-survivor down vs Sepsis_D0_survivor |
| GPBP1 | 5.38E-03 | -1.23 | Sepsis_D0_non-survivor down vs Sepsis_D0_survivor |
| MRPS15 | 5.53E-04 | -1.23 | Sepsis_D0_non-survivor down vs Sepsis_D0_survivor |
| IMPA2 | 2.02E-03 | -1.23 | Sepsis_D0_non-survivor down vs Sepsis_D0_survivor |
| DNAJC8 | 4.92E-03 | -1.23 | Sepsis_D0_non-survivor down vs Sepsis_D0_survivor |
| ATP5B | 4.43E-03 | -1.23 | Sepsis_D0_non-survivor down vs Sepsis_D0_survivor |
| RER1 | 3.13E-04 | -1.23 | Sepsis_D0_non-survivor down vs Sepsis_D0_survivor |
| ING5 | 5.67E-03 | -1.23 | Sepsis_D0_non-survivor down vs Sepsis_D0_survivor |
| MTHFD1 | 7.55E-03 | -1.23 | Sepsis_D0_non-survivor down vs Sepsis_D0_survivor |
| NDUFA7 | 3.62E-03 | -1.23 | Sepsis_D0_non-survivor down vs Sepsis_D0_survivor |
| TAOK3 | 3.58E-03 | -1.22 | Sepsis_D0_non-survivor down vs Sepsis_D0_survivor |
| NDUFB1 | 4.67E-03 | -1.22 | Sepsis_D0_non-survivor down vs Sepsis_D0_survivor |
| WDR39 | 3.16E-03 | -1.22 | Sepsis_D0_non-survivor down vs Sepsis_D0_survivor |
| HIST1H2AB | 5.27E-03 | -1.22 | Sepsis_D0_non-survivor down vs Sepsis_D0_survivor |
| FKBP2 | 4.36E-03 | -1.22 | Sepsis_D0_non-survivor down vs Sepsis_D0_survivor |
| ACTR1A | 7.16E-03 | -1.22 | Sepsis_D0_non-survivor down vs Sepsis_D0_survivor |
| SMYD2 | 2.16E-03 | -1.22 | Sepsis_D0_non-survivor down vs Sepsis_D0_survivor |
| HLA-F | 2.65E-03 | -1.22 | Sepsis_D0_non-survivor down vs Sepsis_D0_survivor |
| RNU3IP2 | 5.50E-03 | -1.22 | Sepsis_D0_non-survivor down vs Sepsis_D0_survivor |
| CCT6A | 5.15E-03 | -1.21 | Sepsis_D0_non-survivor down vs Sepsis_D0_survivor |
| EVC2 | 2.34E-03 | -1.21 | Sepsis_D0_non-survivor down vs Sepsis_D0_survivor |
| EIF4E | 7.02E-03 | -1.21 | Sepsis_D0_non-survivor down vs Sepsis_D0_survivor |
| RPL36A | 3.94E-03 | -1.21 | Sepsis_D0_non-survivor down vs Sepsis_D0_survivor |
| PTPN11 | 5.06E-03 | -1.21 | Sepsis_D0_non-survivor down vs Sepsis_D0_survivor |
| STK24 | 3.96E-03 | -1.21 | Sepsis_D0_non-survivor down vs Sepsis_D0_survivor |
| USP19 | 6.81E-03 | -1.21 | Sepsis_D0_non-survivor down vs Sepsis_D0_survivor |
| NDUFA4 | 3.86E-04 | -1.21 | Sepsis_D0_non-survivor down vs Sepsis_D0_survivor |
| RPL17 | 3.84E-03 | -1.20 | Sepsis_D0_non-survivor down vs Sepsis_D0_survivor |
| NDUFA11 | 3.84E-03 | -1.20 | Sepsis_D0_non-survivor down vs Sepsis_D0_survivor |
| LENG4 | 1.25E-03 | -1.20 | Sepsis_D0_non-survivor down vs Sepsis_D0_survivor |
| UBE2L3 | 5.69E-04 | -1.19 | Sepsis_D0_non-survivor down vs Sepsis_D0_survivor |
| TBX20 | 3.92E-03 | -1.19 | Sepsis_D0_non-survivor down vs Sepsis_D0_survivor |
| XRCC5 | 6.80E-03 | -1.19 | Sepsis_D0_non-survivor down vs Sepsis_D0_survivor |
| OSBPL9 | 5.73E-03 | -1.19 | Sepsis_D0_non-survivor down vs Sepsis_D0_survivor |
| RANGNRF | 6.24E-03 | -1.18 | Sepsis_D0_non-survivor down vs Sepsis_D0_survivor |
| VCP | 2.00E-03 | -1.18 | Sepsis_D0_non-survivor down vs Sepsis_D0_survivor |
| PZP | 5.64E-03 | -1.18 | Sepsis_D0_non-survivor down vs Sepsis_D0_survivor |
| TCEB1 | 4.47E-03 | -1.17 | Sepsis_D0_non-survivor down vs Sepsis_D0_survivor |
| CCL22 | 5.44E-03 | -1.17 | Sepsis_D0_non-survivor down vs Sepsis_D0_survivor |
| GFER | 4.23E-04 | -1.17 | Sepsis_D0_non-survivor down vs Sepsis_D0_survivor |
| MTCP1 | 3.14E-03 | -1.16 | Sepsis_D0_non-survivor down vs Sepsis_D0_survivor |
| PSMC1 | 6.27E-04 | -1.16 | Sepsis_D0_non-survivor down vs Sepsis_D0_survivor |
| LSM12 | 5.81E-03 | -1.16 | Sepsis_D0_non-survivor down vs Sepsis_D0_survivor |
| FBXO18 | 4.55E-03 | -1.16 | Sepsis_D0_non-survivor down vs Sepsis_D0_survivor |
| CRADD | 5.25E-03 | -1.15 | Sepsis_D0_non-survivor down vs Sepsis_D0_survivor |
| ZNF334 | 2.74E-03 | -1.15 | Sepsis_D0_non-survivor down vs Sepsis_D0_survivor |
| CER1 | 1.24E-03 | -1.15 | Sepsis_D0_non-survivor down vs Sepsis_D0_survivor |
| PPP1R2P9 | 5.33E-03 | -1.14 | Sepsis_D0_non-survivor down vs Sepsis_D0_survivor |
| AIM1 | 7.27E-03 | -1.13 | Sepsis_D0_non-survivor down vs Sepsis_D0_survivor |
| BTRC | 4.70E-03 | -1.13 | Sepsis_D0_non-survivor down vs Sepsis_D0_survivor |
| FKBP1A | 1.26E-03 | -1.12 | Sepsis_D0_non-survivor down vs Sepsis_D0_survivor |
| CCDC68 | 2.15E-03 | -1.12 | Sepsis_D0_non-survivor down vs Sepsis_D0_survivor |
| GREM1 | 7.58E-03 | -1.10 | Sepsis_D0_non-survivor down vs Sepsis_D0_survivor |
| PCDH18 | 3.04E-03 | -1.10 | Sepsis_D0_non-survivor down vs Sepsis_D0_survivor |
| FSIP2 | 4.12E-03 | -1.10 | Sepsis_D0_non-survivor down vs Sepsis_D0_survivor |
| FAM70A | 5.62E-03 | -1.10 | Sepsis_D0_non-survivor down vs Sepsis_D0_survivor |
| LAMB4 | 4.32E-04 | -1.09 | Sepsis_D0_non-survivor down vs Sepsis_D0_survivor |
| DSCR9 | 5.52E-03 | -1.08 | Sepsis_D0_non-survivor down vs Sepsis_D0_survivor |
| PAP2D | 6.89E-03 | -1.07 | Sepsis_D0_non-survivor down vs Sepsis_D0_survivor |
| XKRY2 | 6.87E-03 | -1.07 | Sepsis_D0_non-survivor down vs Sepsis_D0_survivor |
| FAM55A | 2.81E-03 | -1.07 | Sepsis_D0_non-survivor down vs Sepsis_D0_survivor |
| TAS2R38 | 1.86E-03 | -1.05 | Sepsis_D0_non-survivor down vs Sepsis_D0_survivor |
| PLSCR2 | 5.18E-03 | 1.11 | Sepsis_D0_non-survivor up vs Sepsis_D0_survivor |
| PSMD5 | 3.48E-03 | 1.16 | Sepsis_D0_non-survivor up vs Sepsis_D0_survivor |
| CCDC12 | 3.89E-03 | 1.18 | Sepsis_D0_non-survivor up vs Sepsis_D0_survivor |
| COL6A1 | 3.53E-03 | 1.18 | Sepsis_D0_non-survivor up vs Sepsis_D0_survivor |
| SP100 | 4.78E-03 | 1.20 | Sepsis_D0_non-survivor up vs Sepsis_D0_survivor |
| OR7E156P | 3.81E-03 | 1.20 | Sepsis_D0_non-survivor up vs Sepsis_D0_survivor |
| VIPR2 | 7.19E-03 | 1.20 | Sepsis_D0_non-survivor up vs Sepsis_D0_survivor |
| DUSP19 | 4.61E-03 | 1.20 | Sepsis_D0_non-survivor up vs Sepsis_D0_survivor |
| MEF2D | 3.17E-03 | 1.20 | Sepsis_D0_non-survivor up vs Sepsis_D0_survivor |
| BRD2 | 6.83E-03 | 1.21 | Sepsis_D0_non-survivor up vs Sepsis_D0_survivor |
| ADD2 | 1.83E-03 | 1.21 | Sepsis_D0_non-survivor up vs Sepsis_D0_survivor |
| RPS20 | 1.55E-03 | 1.21 | Sepsis_D0_non-survivor up vs Sepsis_D0_survivor |
| KLK15 | 3.44E-04 | 1.22 | Sepsis_D0_non-survivor up vs Sepsis_D0_survivor |
| FAM73B | 3.78E-03 | 1.22 | Sepsis_D0_non-survivor up vs Sepsis_D0_survivor |
| ELOVL2 | 4.76E-03 | 1.22 | Sepsis_D0_non-survivor up vs Sepsis_D0_survivor |
| FGF10 | 1.75E-03 | 1.22 | Sepsis_D0_non-survivor up vs Sepsis_D0_survivor |
| RCN3 | 2.64E-03 | 1.22 | Sepsis_D0_non-survivor up vs Sepsis_D0_survivor |
| HHLA3 | 6.16E-04 | 1.22 | Sepsis_D0_non-survivor up vs Sepsis_D0_survivor |
| AGTR1 | 5.53E-03 | 1.22 | Sepsis_D0_non-survivor up vs Sepsis_D0_survivor |
| PLEC1 | 9.22E-04 | 1.23 | Sepsis_D0_non-survivor up vs Sepsis_D0_survivor |
| DLG4 | 1.45E-04 | 1.23 | Sepsis_D0_non-survivor up vs Sepsis_D0_survivor |
| AAAS | 6.95E-03 | 1.23 | Sepsis_D0_non-survivor up vs Sepsis_D0_survivor |
| MFSD7 | 5.51E-03 | 1.23 | Sepsis_D0_non-survivor up vs Sepsis_D0_survivor |
| ARL6IP4 | 2.24E-04 | 1.23 | Sepsis_D0_non-survivor up vs Sepsis_D0_survivor |
| ITFG2 | 6.03E-03 | 1.23 | Sepsis_D0_non-survivor up vs Sepsis_D0_survivor |
| FERD3L | 3.77E-03 | 1.23 | Sepsis_D0_non-survivor up vs Sepsis_D0_survivor |
| RPL13 | 1.66E-03 | 1.23 | Sepsis_D0_non-survivor up vs Sepsis_D0_survivor |
| PCBD1 | 3.56E-03 | 1.23 | Sepsis_D0_non-survivor up vs Sepsis_D0_survivor |
| OR2A20P | 5.74E-03 | 1.23 | Sepsis_D0_non-survivor up vs Sepsis_D0_survivor |
| RNASEH2A | 5.15E-03 | 1.24 | Sepsis_D0_non-survivor up vs Sepsis_D0_survivor |
| RBM15 | 7.36E-03 | 1.24 | Sepsis_D0_non-survivor up vs Sepsis_D0_survivor |
| CEP164 | 3.04E-03 | 1.24 | Sepsis_D0_non-survivor up vs Sepsis_D0_survivor |
| WBSCR18 | 7.61E-03 | 1.24 | Sepsis_D0_non-survivor up vs Sepsis_D0_survivor |
| HIST1H2BA | 4.00E-03 | 1.24 | Sepsis_D0_non-survivor up vs Sepsis_D0_survivor |
| PXMP2 | 6.21E-03 | 1.25 | Sepsis_D0_non-survivor up vs Sepsis_D0_survivor |
| TMEM160 | 6.00E-03 | 1.25 | Sepsis_D0_non-survivor up vs Sepsis_D0_survivor |
| FBS1 | 2.41E-03 | 1.25 | Sepsis_D0_non-survivor up vs Sepsis_D0_survivor |
| SLC5A5 | 6.48E-03 | 1.25 | Sepsis_D0_non-survivor up vs Sepsis_D0_survivor |
| SLC30A2 | 2.76E-03 | 1.25 | Sepsis_D0_non-survivor up vs Sepsis_D0_survivor |
| NCL | 5.45E-03 | 1.25 | Sepsis_D0_non-survivor up vs Sepsis_D0_survivor |
| SOX2 | 6.39E-03 | 1.25 | Sepsis_D0_non-survivor up vs Sepsis_D0_survivor |
| CYP4A11 | 7.01E-04 | 1.25 | Sepsis_D0_non-survivor up vs Sepsis_D0_survivor |
| HSF4 | 6.04E-03 | 1.25 | Sepsis_D0_non-survivor up vs Sepsis_D0_survivor |
| DCTN1 | 3.97E-03 | 1.25 | Sepsis_D0_non-survivor up vs Sepsis_D0_survivor |
| FTSJ1 | 1.42E-03 | 1.26 | Sepsis_D0_non-survivor up vs Sepsis_D0_survivor |
| VSIG1 | 7.47E-03 | 1.26 | Sepsis_D0_non-survivor up vs Sepsis_D0_survivor |
| KRTAP19-1 | 6.24E-03 | 1.26 | Sepsis_D0_non-survivor up vs Sepsis_D0_survivor |
| CTNNB1 | 2.06E-03 | 1.26 | Sepsis_D0_non-survivor up vs Sepsis_D0_survivor |
| SETD5 | 1.08E-03 | 1.26 | Sepsis_D0_non-survivor up vs Sepsis_D0_survivor |
| HGS | 8.02E-04 | 1.26 | Sepsis_D0_non-survivor up vs Sepsis_D0_survivor |
| GPR161 | 1.98E-05 | 1.26 | Sepsis_D0_non-survivor up vs Sepsis_D0_survivor |
| ZNF592 | 9.72E-05 | 1.26 | Sepsis_D0_non-survivor up vs Sepsis_D0_survivor |
| FRAS1 | 2.18E-03 | 1.26 | Sepsis_D0_non-survivor up vs Sepsis_D0_survivor |
| PAX9 | 7.35E-03 | 1.26 | Sepsis_D0_non-survivor up vs Sepsis_D0_survivor |
| GHRHR | 3.97E-03 | 1.27 | Sepsis_D0_non-survivor up vs Sepsis_D0_survivor |
| RPUSD1 | 1.90E-03 | 1.27 | Sepsis_D0_non-survivor up vs Sepsis_D0_survivor |
| VPS37D | 4.93E-04 | 1.27 | Sepsis_D0_non-survivor up vs Sepsis_D0_survivor |
| ADMR | 2.10E-03 | 1.27 | Sepsis_D0_non-survivor up vs Sepsis_D0_survivor |
| PRR4 | 1.02E-03 | 1.27 | Sepsis_D0_non-survivor up vs Sepsis_D0_survivor |
| COL23A1 | 5.14E-04 | 1.27 | Sepsis_D0_non-survivor up vs Sepsis_D0_survivor |
| COL4A2 | 6.16E-03 | 1.27 | Sepsis_D0_non-survivor up vs Sepsis_D0_survivor |
| NT5DC3 | 6.49E-03 | 1.27 | Sepsis_D0_non-survivor up vs Sepsis_D0_survivor |
| PIP | 1.40E-03 | 1.27 | Sepsis_D0_non-survivor up vs Sepsis_D0_survivor |
| IL1F6 | 1.60E-03 | 1.27 | Sepsis_D0_non-survivor up vs Sepsis_D0_survivor |
| BHLHB8 | 4.25E-03 | 1.27 | Sepsis_D0_non-survivor up vs Sepsis_D0_survivor |
| CHRNA5 | 5.38E-03 | 1.27 | Sepsis_D0_non-survivor up vs Sepsis_D0_survivor |
| SSU72 | 3.19E-03 | 1.28 | Sepsis_D0_non-survivor up vs Sepsis_D0_survivor |
| TTLL3 | 5.35E-03 | 1.28 | Sepsis_D0_non-survivor up vs Sepsis_D0_survivor |
| NAGS | 3.27E-03 | 1.28 | Sepsis_D0_non-survivor up vs Sepsis_D0_survivor |
| MST1 | 4.30E-03 | 1.28 | Sepsis_D0_non-survivor up vs Sepsis_D0_survivor |
| ZFAND2B | 3.61E-03 | 1.28 | Sepsis_D0_non-survivor up vs Sepsis_D0_survivor |
| CUGBP1 | 2.73E-03 | 1.29 | Sepsis_D0_non-survivor up vs Sepsis_D0_survivor |
| TBCC | 6.93E-03 | 1.29 | Sepsis_D0_non-survivor up vs Sepsis_D0_survivor |
| LONRF3 | 1.46E-03 | 1.29 | Sepsis_D0_non-survivor up vs Sepsis_D0_survivor |
| HIC2 | 6.99E-03 | 1.29 | Sepsis_D0_non-survivor up vs Sepsis_D0_survivor |
| CYP2A13 | 2.33E-04 | 1.29 | Sepsis_D0_non-survivor up vs Sepsis_D0_survivor |
| ICAM5 | 4.34E-05 | 1.29 | Sepsis_D0_non-survivor up vs Sepsis_D0_survivor |
| RNF183 | 2.04E-03 | 1.29 | Sepsis_D0_non-survivor up vs Sepsis_D0_survivor |
| KCNK16 | 1.82E-03 | 1.29 | Sepsis_D0_non-survivor up vs Sepsis_D0_survivor |
| FLG | 3.21E-03 | 1.29 | Sepsis_D0_non-survivor up vs Sepsis_D0_survivor |
| ARHGEF18 | 5.63E-03 | 1.29 | Sepsis_D0_non-survivor up vs Sepsis_D0_survivor |
| ZNF688 | 1.08E-03 | 1.29 | Sepsis_D0_non-survivor up vs Sepsis_D0_survivor |
| CATSPER2 | 6.40E-03 | 1.30 | Sepsis_D0_non-survivor up vs Sepsis_D0_survivor |
| PROKR1 | 1.94E-03 | 1.30 | Sepsis_D0_non-survivor up vs Sepsis_D0_survivor |
| TRIM29 | 4.03E-03 | 1.30 | Sepsis_D0_non-survivor up vs Sepsis_D0_survivor |
| PORCN | 4.28E-03 | 1.30 | Sepsis_D0_non-survivor up vs Sepsis_D0_survivor |
| KRTAP4-5 | 9.62E-04 | 1.30 | Sepsis_D0_non-survivor up vs Sepsis_D0_survivor |
| SETMAR | 4.55E-03 | 1.30 | Sepsis_D0_non-survivor up vs Sepsis_D0_survivor |
| CHMP6 | 2.35E-03 | 1.31 | Sepsis_D0_non-survivor up vs Sepsis_D0_survivor |
| SLC5A12 | 1.91E-03 | 1.31 | Sepsis_D0_non-survivor up vs Sepsis_D0_survivor |
| SKIP | 3.98E-03 | 1.31 | Sepsis_D0_non-survivor up vs Sepsis_D0_survivor |
| GAL3ST3 | 2.79E-03 | 1.31 | Sepsis_D0_non-survivor up vs Sepsis_D0_survivor |
| PITPNM3 | 2.35E-03 | 1.31 | Sepsis_D0_non-survivor up vs Sepsis_D0_survivor |
| ZNF142 | 8.13E-04 | 1.31 | Sepsis_D0_non-survivor up vs Sepsis_D0_survivor |
| DHX34 | 3.02E-04 | 1.31 | Sepsis_D0_non-survivor up vs Sepsis_D0_survivor |
| MAST1 | 4.13E-03 | 1.31 | Sepsis_D0_non-survivor up vs Sepsis_D0_survivor |
| BTBD5 | 5.57E-04 | 1.31 | Sepsis_D0_non-survivor up vs Sepsis_D0_survivor |
| CA7 | 2.30E-03 | 1.31 | Sepsis_D0_non-survivor up vs Sepsis_D0_survivor |
| CENPB | 9.61E-04 | 1.31 | Sepsis_D0_non-survivor up vs Sepsis_D0_survivor |
| JUND | 5.06E-03 | 1.31 | Sepsis_D0_non-survivor up vs Sepsis_D0_survivor |
| OR2J2 | 2.36E-03 | 1.31 | Sepsis_D0_non-survivor up vs Sepsis_D0_survivor |
| PAPLN | 4.71E-03 | 1.31 | Sepsis_D0_non-survivor up vs Sepsis_D0_survivor |
| SLC6A18 | 3.82E-03 | 1.31 | Sepsis_D0_non-survivor up vs Sepsis_D0_survivor |
| OKL38 | 4.95E-03 | 1.31 | Sepsis_D0_non-survivor up vs Sepsis_D0_survivor |
| KCNG1 | 1.98E-03 | 1.31 | Sepsis_D0_non-survivor up vs Sepsis_D0_survivor |
| SLC2A4 | 5.16E-03 | 1.32 | Sepsis_D0_non-survivor up vs Sepsis_D0_survivor |
| DOHH | 4.16E-03 | 1.32 | Sepsis_D0_non-survivor up vs Sepsis_D0_survivor |
| TNNI3K | 1.46E-03 | 1.32 | Sepsis_D0_non-survivor up vs Sepsis_D0_survivor |
| SPATA20 | 4.61E-03 | 1.32 | Sepsis_D0_non-survivor up vs Sepsis_D0_survivor |
| RABEP2 | 2.12E-03 | 1.32 | Sepsis_D0_non-survivor up vs Sepsis_D0_survivor |
| KCNJ1 | 1.31E-03 | 1.32 | Sepsis_D0_non-survivor up vs Sepsis_D0_survivor |
| GRM7 | 4.99E-03 | 1.32 | Sepsis_D0_non-survivor up vs Sepsis_D0_survivor |
| PYY | 7.52E-04 | 1.32 | Sepsis_D0_non-survivor up vs Sepsis_D0_survivor |
| SCARF2 | 9.81E-04 | 1.32 | Sepsis_D0_non-survivor up vs Sepsis_D0_survivor |
| GPR113 | 1.39E-03 | 1.32 | Sepsis_D0_non-survivor up vs Sepsis_D0_survivor |
| CECR1 | 1.66E-03 | 1.32 | Sepsis_D0_non-survivor up vs Sepsis_D0_survivor |
| LRRC15 | 6.52E-04 | 1.32 | Sepsis_D0_non-survivor up vs Sepsis_D0_survivor |
| NUDT16L1 | 2.77E-03 | 1.32 | Sepsis_D0_non-survivor up vs Sepsis_D0_survivor |
| KRT2B | 6.30E-03 | 1.32 | Sepsis_D0_non-survivor up vs Sepsis_D0_survivor |
| SPN | 3.20E-03 | 1.33 | Sepsis_D0_non-survivor up vs Sepsis_D0_survivor |
| ZNF423 | 2.86E-03 | 1.33 | Sepsis_D0_non-survivor up vs Sepsis_D0_survivor |
| PLCD4 | 3.40E-03 | 1.33 | Sepsis_D0_non-survivor up vs Sepsis_D0_survivor |
| ORMDL3 | 7.60E-04 | 1.33 | Sepsis_D0_non-survivor up vs Sepsis_D0_survivor |
| MEG3 | 7.91E-04 | 1.33 | Sepsis_D0_non-survivor up vs Sepsis_D0_survivor |
| GPR62 | 4.31E-04 | 1.33 | Sepsis_D0_non-survivor up vs Sepsis_D0_survivor |
| MEIS3 | 4.70E-03 | 1.33 | Sepsis_D0_non-survivor up vs Sepsis_D0_survivor |
| KIF1C | 2.04E-03 | 1.33 | Sepsis_D0_non-survivor up vs Sepsis_D0_survivor |
| SH3BP1 | 6.05E-03 | 1.33 | Sepsis_D0_non-survivor up vs Sepsis_D0_survivor |
| PELI2 | 5.95E-04 | 1.33 | Sepsis_D0_non-survivor up vs Sepsis_D0_survivor |
| KCNE1 | 6.61E-03 | 1.33 | Sepsis_D0_non-survivor up vs Sepsis_D0_survivor |
| ATF4 | 3.67E-03 | 1.33 | Sepsis_D0_non-survivor up vs Sepsis_D0_survivor |
| ZNF446 | 7.07E-04 | 1.33 | Sepsis_D0_non-survivor up vs Sepsis_D0_survivor |
| NPAS2 | 6.67E-03 | 1.33 | Sepsis_D0_non-survivor up vs Sepsis_D0_survivor |
| BRF1 | 8.12E-04 | 1.33 | Sepsis_D0_non-survivor up vs Sepsis_D0_survivor |
| ZNF135 | 2.20E-03 | 1.34 | Sepsis_D0_non-survivor up vs Sepsis_D0_survivor |
| IGF1 | 6.96E-03 | 1.34 | Sepsis_D0_non-survivor up vs Sepsis_D0_survivor |
| TCF2 | 6.33E-03 | 1.34 | Sepsis_D0_non-survivor up vs Sepsis_D0_survivor |
| YPEL1 | 6.64E-03 | 1.34 | Sepsis_D0_non-survivor up vs Sepsis_D0_survivor |
| LRRC41 | 1.26E-03 | 1.34 | Sepsis_D0_non-survivor up vs Sepsis_D0_survivor |
| DCAKD | 7.59E-03 | 1.34 | Sepsis_D0_non-survivor up vs Sepsis_D0_survivor |
| EXOC2 | 2.50E-03 | 1.34 | Sepsis_D0_non-survivor up vs Sepsis_D0_survivor |
| SCARA3 | 9.05E-04 | 1.34 | Sepsis_D0_non-survivor up vs Sepsis_D0_survivor |
| SLIT1 | 4.69E-03 | 1.34 | Sepsis_D0_non-survivor up vs Sepsis_D0_survivor |
| SLC6A8 | 5.13E-04 | 1.34 | Sepsis_D0_non-survivor up vs Sepsis_D0_survivor |
| GOLGA | 5.95E-03 | 1.34 | Sepsis_D0_non-survivor up vs Sepsis_D0_survivor |
| CSF1 | 6.39E-03 | 1.34 | Sepsis_D0_non-survivor up vs Sepsis_D0_survivor |
| ITIH3 | 5.90E-03 | 1.35 | Sepsis_D0_non-survivor up vs Sepsis_D0_survivor |
| FLYWCH1 | 9.03E-04 | 1.35 | Sepsis_D0_non-survivor up vs Sepsis_D0_survivor |
| SCRIB | 5.42E-03 | 1.35 | Sepsis_D0_non-survivor up vs Sepsis_D0_survivor |
| ICOSLG | 1.91E-03 | 1.35 | Sepsis_D0_non-survivor up vs Sepsis_D0_survivor |
| ODZ1 | 1.41E-03 | 1.35 | Sepsis_D0_non-survivor up vs Sepsis_D0_survivor |
| C1QTNF1 | 8.65E-04 | 1.35 | Sepsis_D0_non-survivor up vs Sepsis_D0_survivor |
| RNF26 | 7.81E-04 | 1.35 | Sepsis_D0_non-survivor up vs Sepsis_D0_survivor |
| HSF5 | 1.78E-03 | 1.35 | Sepsis_D0_non-survivor up vs Sepsis_D0_survivor |
| TUBB4 | 2.46E-03 | 1.35 | Sepsis_D0_non-survivor up vs Sepsis_D0_survivor |
| HIF3A | 5.21E-03 | 1.35 | Sepsis_D0_non-survivor up vs Sepsis_D0_survivor |
| LMAN1L | 9.38E-04 | 1.35 | Sepsis_D0_non-survivor up vs Sepsis_D0_survivor |
| TSPYL2 | 7.48E-03 | 1.35 | Sepsis_D0_non-survivor up vs Sepsis_D0_survivor |
| BMP10 | 2.53E-03 | 1.35 | Sepsis_D0_non-survivor up vs Sepsis_D0_survivor |
| DNAJC6 | 5.22E-03 | 1.35 | Sepsis_D0_non-survivor up vs Sepsis_D0_survivor |
| EMILIN1 | 6.52E-03 | 1.35 | Sepsis_D0_non-survivor up vs Sepsis_D0_survivor |
| FOLR2 | 5.40E-03 | 1.35 | Sepsis_D0_non-survivor up vs Sepsis_D0_survivor |
| PCDHB8 | 3.93E-03 | 1.35 | Sepsis_D0_non-survivor up vs Sepsis_D0_survivor |
| FCAMR | 1.61E-03 | 1.35 | Sepsis_D0_non-survivor up vs Sepsis_D0_survivor |
| HTR3A | 7.71E-03 | 1.35 | Sepsis_D0_non-survivor up vs Sepsis_D0_survivor |
| ARID3A | 5.87E-03 | 1.35 | Sepsis_D0_non-survivor up vs Sepsis_D0_survivor |
| ALS2CL | 4.38E-04 | 1.35 | Sepsis_D0_non-survivor up vs Sepsis_D0_survivor |
| MYL7 | 3.18E-04 | 1.36 | Sepsis_D0_non-survivor up vs Sepsis_D0_survivor |
| SIRT6 | 4.29E-03 | 1.36 | Sepsis_D0_non-survivor up vs Sepsis_D0_survivor |
| ABCA8 | 5.23E-03 | 1.36 | Sepsis_D0_non-survivor up vs Sepsis_D0_survivor |
| PEX10 | 1.39E-03 | 1.36 | Sepsis_D0_non-survivor up vs Sepsis_D0_survivor |
| CCL17 | 2.23E-03 | 1.36 | Sepsis_D0_non-survivor up vs Sepsis_D0_survivor |
| ZNF679 | 6.64E-04 | 1.36 | Sepsis_D0_non-survivor up vs Sepsis_D0_survivor |
| PPAN | 3.36E-03 | 1.36 | Sepsis_D0_non-survivor up vs Sepsis_D0_survivor |
| TAS2R43 | 4.51E-03 | 1.36 | Sepsis_D0_non-survivor up vs Sepsis_D0_survivor |
| ANKRD2 | 2.72E-03 | 1.36 | Sepsis_D0_non-survivor up vs Sepsis_D0_survivor |
| MCCD1 | 4.94E-03 | 1.36 | Sepsis_D0_non-survivor up vs Sepsis_D0_survivor |
| TAOK2 | 7.55E-03 | 1.36 | Sepsis_D0_non-survivor up vs Sepsis_D0_survivor |
| OR7E47P | 5.50E-04 | 1.36 | Sepsis_D0_non-survivor up vs Sepsis_D0_survivor |
| ALX3 | 3.74E-03 | 1.36 | Sepsis_D0_non-survivor up vs Sepsis_D0_survivor |
| GIPC3 | 6.73E-03 | 1.36 | Sepsis_D0_non-survivor up vs Sepsis_D0_survivor |
| PCOLCE | 5.30E-03 | 1.37 | Sepsis_D0_non-survivor up vs Sepsis_D0_survivor |
| GPR3 | 1.65E-03 | 1.37 | Sepsis_D0_non-survivor up vs Sepsis_D0_survivor |
| SHB | 2.92E-03 | 1.37 | Sepsis_D0_non-survivor up vs Sepsis_D0_survivor |
| RAPSN | 7.08E-03 | 1.37 | Sepsis_D0_non-survivor up vs Sepsis_D0_survivor |
| PRIMA1 | 6.42E-03 | 1.37 | Sepsis_D0_non-survivor up vs Sepsis_D0_survivor |
| FRAG1 | 3.84E-03 | 1.37 | Sepsis_D0_non-survivor up vs Sepsis_D0_survivor |
| OR51E2 | 6.20E-04 | 1.37 | Sepsis_D0_non-survivor up vs Sepsis_D0_survivor |
| PRO2964 | 2.14E-03 | 1.37 | Sepsis_D0_non-survivor up vs Sepsis_D0_survivor |
| CHKA | 1.54E-03 | 1.37 | Sepsis_D0_non-survivor up vs Sepsis_D0_survivor |
| RTEL1 | 4.96E-03 | 1.37 | Sepsis_D0_non-survivor up vs Sepsis_D0_survivor |
| FKSG83 | 3.36E-03 | 1.37 | Sepsis_D0_non-survivor up vs Sepsis_D0_survivor |
| PNMA5 | 2.92E-03 | 1.37 | Sepsis_D0_non-survivor up vs Sepsis_D0_survivor |
| NUP188 | 6.88E-03 | 1.37 | Sepsis_D0_non-survivor up vs Sepsis_D0_survivor |
| TNNT2 | 6.11E-03 | 1.37 | Sepsis_D0_non-survivor up vs Sepsis_D0_survivor |
| AVPR2 | 7.01E-04 | 1.37 | Sepsis_D0_non-survivor up vs Sepsis_D0_survivor |
| LRIG1 | 7.12E-03 | 1.37 | Sepsis_D0_non-survivor up vs Sepsis_D0_survivor |
| CHST13 | 1.54E-03 | 1.37 | Sepsis_D0_non-survivor up vs Sepsis_D0_survivor |
| SLC6A13 | 8.27E-04 | 1.37 | Sepsis_D0_non-survivor up vs Sepsis_D0_survivor |
| VTN | 7.46E-03 | 1.38 | Sepsis_D0_non-survivor up vs Sepsis_D0_survivor |
| ACVR2B | 4.74E-03 | 1.38 | Sepsis_D0_non-survivor up vs Sepsis_D0_survivor |
| FBLIM1 | 2.11E-03 | 1.38 | Sepsis_D0_non-survivor up vs Sepsis_D0_survivor |
| ZNF500 | 2.76E-03 | 1.38 | Sepsis_D0_non-survivor up vs Sepsis_D0_survivor |
| CIRBP | 9.19E-04 | 1.38 | Sepsis_D0_non-survivor up vs Sepsis_D0_survivor |
| OR6N1 | 1.86E-03 | 1.38 | Sepsis_D0_non-survivor up vs Sepsis_D0_survivor |
| GALR2 | 6.29E-03 | 1.38 | Sepsis_D0_non-survivor up vs Sepsis_D0_survivor |
| MRPL38 | 4.52E-04 | 1.38 | Sepsis_D0_non-survivor up vs Sepsis_D0_survivor |
| OR10H3 | 5.63E-03 | 1.38 | Sepsis_D0_non-survivor up vs Sepsis_D0_survivor |
| TUBD1 | 3.05E-03 | 1.38 | Sepsis_D0_non-survivor up vs Sepsis_D0_survivor |
| HMCN2 | 9.51E-04 | 1.38 | Sepsis_D0_non-survivor up vs Sepsis_D0_survivor |
| SMOC2 | 4.45E-03 | 1.38 | Sepsis_D0_non-survivor up vs Sepsis_D0_survivor |
| DOCK3 | 6.37E-05 | 1.38 | Sepsis_D0_non-survivor up vs Sepsis_D0_survivor |
| AGRIN | 1.90E-04 | 1.38 | Sepsis_D0_non-survivor up vs Sepsis_D0_survivor |
| HRK | 3.37E-03 | 1.38 | Sepsis_D0_non-survivor up vs Sepsis_D0_survivor |
| NES | 5.10E-03 | 1.38 | Sepsis_D0_non-survivor up vs Sepsis_D0_survivor |
| FTH1 | 2.94E-03 | 1.39 | Sepsis_D0_non-survivor up vs Sepsis_D0_survivor |
| PRO1580 | 1.46E-05 | 1.39 | Sepsis_D0_non-survivor up vs Sepsis_D0_survivor |
| TOR2A | 5.03E-05 | 1.39 | Sepsis_D0_non-survivor up vs Sepsis_D0_survivor |
| ZNF221 | 6.29E-03 | 1.39 | Sepsis_D0_non-survivor up vs Sepsis_D0_survivor |
| CSDC2 | 2.18E-03 | 1.39 | Sepsis_D0_non-survivor up vs Sepsis_D0_survivor |
| HLA-C | 3.53E-03 | 1.39 | Sepsis_D0_non-survivor up vs Sepsis_D0_survivor |
| PCDHB18 | 9.37E-04 | 1.39 | Sepsis_D0_non-survivor up vs Sepsis_D0_survivor |
| VCX2 | 1.67E-03 | 1.39 | Sepsis_D0_non-survivor up vs Sepsis_D0_survivor |
| FRZB | 7.43E-04 | 1.39 | Sepsis_D0_non-survivor up vs Sepsis_D0_survivor |
| HOXB5 | 1.47E-03 | 1.39 | Sepsis_D0_non-survivor up vs Sepsis_D0_survivor |
| KCNK15 | 1.96E-03 | 1.39 | Sepsis_D0_non-survivor up vs Sepsis_D0_survivor |
| RPS28 | 4.01E-03 | 1.39 | Sepsis_D0_non-survivor up vs Sepsis_D0_survivor |
| CPA6 | 5.86E-03 | 1.39 | Sepsis_D0_non-survivor up vs Sepsis_D0_survivor |
| CYP11A1 | 3.64E-03 | 1.39 | Sepsis_D0_non-survivor up vs Sepsis_D0_survivor |
| TPTE | 5.47E-04 | 1.39 | Sepsis_D0_non-survivor up vs Sepsis_D0_survivor |
| PKMYT1 | 2.70E-03 | 1.39 | Sepsis_D0_non-survivor up vs Sepsis_D0_survivor |
| TEAD3 | 5.11E-03 | 1.40 | Sepsis_D0_non-survivor up vs Sepsis_D0_survivor |
| APLN | 2.93E-04 | 1.40 | Sepsis_D0_non-survivor up vs Sepsis_D0_survivor |
| RPL11 | 2.21E-04 | 1.40 | Sepsis_D0_non-survivor up vs Sepsis_D0_survivor |
| PRIC285 | 3.74E-04 | 1.40 | Sepsis_D0_non-survivor up vs Sepsis_D0_survivor |
| IQGAP3 | 4.92E-03 | 1.40 | Sepsis_D0_non-survivor up vs Sepsis_D0_survivor |
| MANBAL | 3.30E-03 | 1.40 | Sepsis_D0_non-survivor up vs Sepsis_D0_survivor |
| IGLV6-57 | 3.83E-03 | 1.40 | Sepsis_D0_non-survivor up vs Sepsis_D0_survivor |
| TMEM151 | 6.02E-03 | 1.40 | Sepsis_D0_non-survivor up vs Sepsis_D0_survivor |
| IGHA1 | 5.66E-03 | 1.40 | Sepsis_D0_non-survivor up vs Sepsis_D0_survivor |
| CCDC88 | 1.47E-03 | 1.40 | Sepsis_D0_non-survivor up vs Sepsis_D0_survivor |
| SYNGR3 | 5.60E-03 | 1.40 | Sepsis_D0_non-survivor up vs Sepsis_D0_survivor |
| NAB2 | 6.63E-03 | 1.40 | Sepsis_D0_non-survivor up vs Sepsis_D0_survivor |
| UNC5A | 2.15E-03 | 1.40 | Sepsis_D0_non-survivor up vs Sepsis_D0_survivor |
| EXDL1 | 5.40E-03 | 1.40 | Sepsis_D0_non-survivor up vs Sepsis_D0_survivor |
| ELN | 1.36E-03 | 1.40 | Sepsis_D0_non-survivor up vs Sepsis_D0_survivor |
| AHSG | 1.65E-03 | 1.40 | Sepsis_D0_non-survivor up vs Sepsis_D0_survivor |
| RPS6KA2 | 1.41E-03 | 1.40 | Sepsis_D0_non-survivor up vs Sepsis_D0_survivor |
| BHLHB5 | 1.73E-04 | 1.40 | Sepsis_D0_non-survivor up vs Sepsis_D0_survivor |
| TMED6 | 4.04E-03 | 1.40 | Sepsis_D0_non-survivor up vs Sepsis_D0_survivor |
| NRN1 | 6.87E-04 | 1.40 | Sepsis_D0_non-survivor up vs Sepsis_D0_survivor |
| CORO7 | 2.75E-03 | 1.40 | Sepsis_D0_non-survivor up vs Sepsis_D0_survivor |
| KCNC3 | 6.33E-03 | 1.40 | Sepsis_D0_non-survivor up vs Sepsis_D0_survivor |
| SYT2 | 2.90E-03 | 1.40 | Sepsis_D0_non-survivor up vs Sepsis_D0_survivor |
| HDAC11 | 5.41E-03 | 1.40 | Sepsis_D0_non-survivor up vs Sepsis_D0_survivor |
| PRR3 | 1.61E-04 | 1.41 | Sepsis_D0_non-survivor up vs Sepsis_D0_survivor |
| SCGB1A1 | 3.82E-03 | 1.41 | Sepsis_D0_non-survivor up vs Sepsis_D0_survivor |
| SPATA1 | 4.47E-03 | 1.41 | Sepsis_D0_non-survivor up vs Sepsis_D0_survivor |
| EPB41L2 | 1.32E-04 | 1.41 | Sepsis_D0_non-survivor up vs Sepsis_D0_survivor |
| MOS | 8.84E-04 | 1.41 | Sepsis_D0_non-survivor up vs Sepsis_D0_survivor |
| FUT5 | 3.41E-03 | 1.41 | Sepsis_D0_non-survivor up vs Sepsis_D0_survivor |
| WFDC3 | 3.64E-03 | 1.41 | Sepsis_D0_non-survivor up vs Sepsis_D0_survivor |
| LHFPL4 | 6.30E-03 | 1.41 | Sepsis_D0_non-survivor up vs Sepsis_D0_survivor |
| AQP5 | 1.62E-03 | 1.41 | Sepsis_D0_non-survivor up vs Sepsis_D0_survivor |
| NRIP3 | 3.61E-03 | 1.41 | Sepsis_D0_non-survivor up vs Sepsis_D0_survivor |
| HCN4 | 6.23E-03 | 1.41 | Sepsis_D0_non-survivor up vs Sepsis_D0_survivor |
| CIB2 | 4.16E-03 | 1.41 | Sepsis_D0_non-survivor up vs Sepsis_D0_survivor |
| KCNQ5 | 4.07E-03 | 1.41 | Sepsis_D0_non-survivor up vs Sepsis_D0_survivor |
| TAS2R39 | 7.50E-03 | 1.41 | Sepsis_D0_non-survivor up vs Sepsis_D0_survivor |
| OR10G8 | 4.45E-03 | 1.41 | Sepsis_D0_non-survivor up vs Sepsis_D0_survivor |
| VIM | 1.29E-03 | 1.41 | Sepsis_D0_non-survivor up vs Sepsis_D0_survivor |
| TRIM42 | 7.43E-03 | 1.41 | Sepsis_D0_non-survivor up vs Sepsis_D0_survivor |
| NOPE | 7.42E-04 | 1.41 | Sepsis_D0_non-survivor up vs Sepsis_D0_survivor |
| HTR1B | 1.93E-03 | 1.42 | Sepsis_D0_non-survivor up vs Sepsis_D0_survivor |
| EML4 | 6.29E-03 | 1.42 | Sepsis_D0_non-survivor up vs Sepsis_D0_survivor |
| GFRA3 | 6.70E-03 | 1.42 | Sepsis_D0_non-survivor up vs Sepsis_D0_survivor |
| GPR156 | 5.02E-04 | 1.42 | Sepsis_D0_non-survivor up vs Sepsis_D0_survivor |
| OR12D3 | 7.61E-03 | 1.42 | Sepsis_D0_non-survivor up vs Sepsis_D0_survivor |
| APC2 | 1.96E-04 | 1.42 | Sepsis_D0_non-survivor up vs Sepsis_D0_survivor |
| SUPT5H | 3.37E-03 | 1.42 | Sepsis_D0_non-survivor up vs Sepsis_D0_survivor |
| ZC3H10 | 5.86E-03 | 1.42 | Sepsis_D0_non-survivor up vs Sepsis_D0_survivor |
| CDR2L | 2.93E-03 | 1.42 | Sepsis_D0_non-survivor up vs Sepsis_D0_survivor |
| ARFGEF1 | 4.85E-03 | 1.42 | Sepsis_D0_non-survivor up vs Sepsis_D0_survivor |
| CLCNKA | 2.23E-04 | 1.42 | Sepsis_D0_non-survivor up vs Sepsis_D0_survivor |
| SYTL5 | 9.67E-04 | 1.42 | Sepsis_D0_non-survivor up vs Sepsis_D0_survivor |
| UTRN | 4.60E-04 | 1.43 | Sepsis_D0_non-survivor up vs Sepsis_D0_survivor |
| TRPM8 | 5.32E-03 | 1.43 | Sepsis_D0_non-survivor up vs Sepsis_D0_survivor |
| DDX11 | 5.47E-03 | 1.43 | Sepsis_D0_non-survivor up vs Sepsis_D0_survivor |
| KLC2 | 6.49E-03 | 1.43 | Sepsis_D0_non-survivor up vs Sepsis_D0_survivor |
| NDFIP2 | 2.71E-03 | 1.43 | Sepsis_D0_non-survivor up vs Sepsis_D0_survivor |
| XTP7 | 4.83E-03 | 1.43 | Sepsis_D0_non-survivor up vs Sepsis_D0_survivor |
| KIF19 | 5.45E-03 | 1.43 | Sepsis_D0_non-survivor up vs Sepsis_D0_survivor |
| S100A2 | 1.04E-03 | 1.43 | Sepsis_D0_non-survivor up vs Sepsis_D0_survivor |
| SAMD11 | 6.02E-03 | 1.43 | Sepsis_D0_non-survivor up vs Sepsis_D0_survivor |
| EMX1 | 1.12E-03 | 1.43 | Sepsis_D0_non-survivor up vs Sepsis_D0_survivor |
| PLCD3 | 1.03E-04 | 1.43 | Sepsis_D0_non-survivor up vs Sepsis_D0_survivor |
| CSH1 | 4.69E-03 | 1.43 | Sepsis_D0_non-survivor up vs Sepsis_D0_survivor |
| IQSEC2 | 4.87E-03 | 1.43 | Sepsis_D0_non-survivor up vs Sepsis_D0_survivor |
| RSHL1 | 6.26E-05 | 1.44 | Sepsis_D0_non-survivor up vs Sepsis_D0_survivor |
| PNPLA7 | 4.85E-03 | 1.44 | Sepsis_D0_non-survivor up vs Sepsis_D0_survivor |
| CCIN | 2.41E-03 | 1.44 | Sepsis_D0_non-survivor up vs Sepsis_D0_survivor |
| TCOF1 | 1.09E-03 | 1.44 | Sepsis_D0_non-survivor up vs Sepsis_D0_survivor |
| ZNF169 | 3.30E-03 | 1.44 | Sepsis_D0_non-survivor up vs Sepsis_D0_survivor |
| WNT10A | 4.59E-03 | 1.44 | Sepsis_D0_non-survivor up vs Sepsis_D0_survivor |
| HRH2 | 1.95E-03 | 1.44 | Sepsis_D0_non-survivor up vs Sepsis_D0_survivor |
| LUZP4 | 3.58E-03 | 1.44 | Sepsis_D0_non-survivor up vs Sepsis_D0_survivor |
| TSSK4 | 1.33E-03 | 1.44 | Sepsis_D0_non-survivor up vs Sepsis_D0_survivor |
| NOG | 1.06E-03 | 1.44 | Sepsis_D0_non-survivor up vs Sepsis_D0_survivor |
| MOSPD3 | 3.16E-03 | 1.44 | Sepsis_D0_non-survivor up vs Sepsis_D0_survivor |
| OR4C15 | 5.44E-03 | 1.44 | Sepsis_D0_non-survivor up vs Sepsis_D0_survivor |
| GM632 | 2.79E-04 | 1.44 | Sepsis_D0_non-survivor up vs Sepsis_D0_survivor |
| REG1B | 5.21E-04 | 1.44 | Sepsis_D0_non-survivor up vs Sepsis_D0_survivor |
| TIP39 | 8.94E-04 | 1.44 | Sepsis_D0_non-survivor up vs Sepsis_D0_survivor |
| ERCC2 | 1.12E-04 | 1.44 | Sepsis_D0_non-survivor up vs Sepsis_D0_survivor |
| GALNTL1 | 7.51E-03 | 1.44 | Sepsis_D0_non-survivor up vs Sepsis_D0_survivor |
| VN1R1 | 4.82E-03 | 1.44 | Sepsis_D0_non-survivor up vs Sepsis_D0_survivor |
| PARS2 | 2.74E-05 | 1.44 | Sepsis_D0_non-survivor up vs Sepsis_D0_survivor |
| ZNRF4 | 2.03E-03 | 1.44 | Sepsis_D0_non-survivor up vs Sepsis_D0_survivor |
| DEDD2 | 3.37E-05 | 1.44 | Sepsis_D0_non-survivor up vs Sepsis_D0_survivor |
| ELAVL3 | 7.18E-03 | 1.44 | Sepsis_D0_non-survivor up vs Sepsis_D0_survivor |
| CRYBB3 | 1.20E-04 | 1.44 | Sepsis_D0_non-survivor up vs Sepsis_D0_survivor |
| ZNF496 | 2.55E-03 | 1.44 | Sepsis_D0_non-survivor up vs Sepsis_D0_survivor |
| POLD2 | 9.95E-04 | 1.44 | Sepsis_D0_non-survivor up vs Sepsis_D0_survivor |
| C3 | 5.76E-03 | 1.45 | Sepsis_D0_non-survivor up vs Sepsis_D0_survivor |
| SSX4 | 3.02E-03 | 1.45 | Sepsis_D0_non-survivor up vs Sepsis_D0_survivor |
| CIB3 | 4.07E-03 | 1.45 | Sepsis_D0_non-survivor up vs Sepsis_D0_survivor |
| GCS1 | 1.03E-03 | 1.45 | Sepsis_D0_non-survivor up vs Sepsis_D0_survivor |
| LRCH3 | 8.11E-04 | 1.45 | Sepsis_D0_non-survivor up vs Sepsis_D0_survivor |
| ENPEP | 5.63E-04 | 1.45 | Sepsis_D0_non-survivor up vs Sepsis_D0_survivor |
| FUZ | 2.76E-04 | 1.45 | Sepsis_D0_non-survivor up vs Sepsis_D0_survivor |
| RAP1GAP | 1.60E-03 | 1.45 | Sepsis_D0_non-survivor up vs Sepsis_D0_survivor |
| ISYNA1 | 7.45E-03 | 1.45 | Sepsis_D0_non-survivor up vs Sepsis_D0_survivor |
| SYT5 | 6.10E-03 | 1.45 | Sepsis_D0_non-survivor up vs Sepsis_D0_survivor |
| LMX1A | 3.10E-03 | 1.45 | Sepsis_D0_non-survivor up vs Sepsis_D0_survivor |
| OR11A1 | 6.42E-04 | 1.45 | Sepsis_D0_non-survivor up vs Sepsis_D0_survivor |
| TTC9B | 8.17E-04 | 1.45 | Sepsis_D0_non-survivor up vs Sepsis_D0_survivor |
| DRD1IP | 2.45E-03 | 1.45 | Sepsis_D0_non-survivor up vs Sepsis_D0_survivor |
| PLG | 6.61E-03 | 1.45 | Sepsis_D0_non-survivor up vs Sepsis_D0_survivor |
| NLC1-C | 7.20E-04 | 1.45 | Sepsis_D0_non-survivor up vs Sepsis_D0_survivor |
| SMAD6 | 4.90E-04 | 1.45 | Sepsis_D0_non-survivor up vs Sepsis_D0_survivor |
| FAIM2 | 1.06E-03 | 1.45 | Sepsis_D0_non-survivor up vs Sepsis_D0_survivor |
| SFXN5 | 5.39E-03 | 1.45 | Sepsis_D0_non-survivor up vs Sepsis_D0_survivor |
| CLCA4 | 1.62E-03 | 1.45 | Sepsis_D0_non-survivor up vs Sepsis_D0_survivor |
| SNCB | 2.17E-03 | 1.45 | Sepsis_D0_non-survivor up vs Sepsis_D0_survivor |
| TAS2R44 | 3.07E-03 | 1.45 | Sepsis_D0_non-survivor up vs Sepsis_D0_survivor |
| UGT2B17 | 3.93E-03 | 1.46 | Sepsis_D0_non-survivor up vs Sepsis_D0_survivor |
| PROK1 | 4.17E-03 | 1.46 | Sepsis_D0_non-survivor up vs Sepsis_D0_survivor |
| MAPK15 | 3.62E-04 | 1.46 | Sepsis_D0_non-survivor up vs Sepsis_D0_survivor |
| GRHL3 | 2.29E-03 | 1.46 | Sepsis_D0_non-survivor up vs Sepsis_D0_survivor |
| CABP4 | 3.41E-03 | 1.46 | Sepsis_D0_non-survivor up vs Sepsis_D0_survivor |
| DUX4 | 5.34E-03 | 1.46 | Sepsis_D0_non-survivor up vs Sepsis_D0_survivor |
| LYPD4 | 3.76E-04 | 1.46 | Sepsis_D0_non-survivor up vs Sepsis_D0_survivor |
| KLF14 | 3.58E-05 | 1.46 | Sepsis_D0_non-survivor up vs Sepsis_D0_survivor |
| IGSF3 | 1.96E-03 | 1.46 | Sepsis_D0_non-survivor up vs Sepsis_D0_survivor |
| NKD1 | 3.78E-03 | 1.46 | Sepsis_D0_non-survivor up vs Sepsis_D0_survivor |
| ADRA1D | 5.14E-03 | 1.46 | Sepsis_D0_non-survivor up vs Sepsis_D0_survivor |
| ST8SIA2 | 2.64E-03 | 1.47 | Sepsis_D0_non-survivor up vs Sepsis_D0_survivor |
| RGMA | 2.83E-03 | 1.47 | Sepsis_D0_non-survivor up vs Sepsis_D0_survivor |
| ST6GALNAC4 | 7.55E-04 | 1.47 | Sepsis_D0_non-survivor up vs Sepsis_D0_survivor |
| OR51D1 | 2.46E-03 | 1.47 | Sepsis_D0_non-survivor up vs Sepsis_D0_survivor |
| CD3E | 1.10E-03 | 1.47 | Sepsis_D0_non-survivor up vs Sepsis_D0_survivor |
| SLC22A6 | 6.79E-03 | 1.47 | Sepsis_D0_non-survivor up vs Sepsis_D0_survivor |
| UMODL1 | 6.00E-04 | 1.47 | Sepsis_D0_non-survivor up vs Sepsis_D0_survivor |
| NDRG4 | 6.39E-03 | 1.47 | Sepsis_D0_non-survivor up vs Sepsis_D0_survivor |
| IL21 | 1.97E-03 | 1.47 | Sepsis_D0_non-survivor up vs Sepsis_D0_survivor |
| KCNN2 | 6.78E-03 | 1.47 | Sepsis_D0_non-survivor up vs Sepsis_D0_survivor |
| B3GNT6 | 1.87E-03 | 1.47 | Sepsis_D0_non-survivor up vs Sepsis_D0_survivor |
| BTN2A3 | 7.51E-03 | 1.47 | Sepsis_D0_non-survivor up vs Sepsis_D0_survivor |
| KCNH4 | 2.15E-03 | 1.47 | Sepsis_D0_non-survivor up vs Sepsis_D0_survivor |
| PCDHGA12 | 6.39E-03 | 1.47 | Sepsis_D0_non-survivor up vs Sepsis_D0_survivor |
| M74509 | 4.27E-04 | 1.47 | Sepsis_D0_non-survivor up vs Sepsis_D0_survivor |
| TLX2 | 4.94E-03 | 1.47 | Sepsis_D0_non-survivor up vs Sepsis_D0_survivor |
| CACNB3 | 1.70E-04 | 1.47 | Sepsis_D0_non-survivor up vs Sepsis_D0_survivor |
| SERPINA9 | 4.42E-03 | 1.47 | Sepsis_D0_non-survivor up vs Sepsis_D0_survivor |
| AES | 6.41E-03 | 1.47 | Sepsis_D0_non-survivor up vs Sepsis_D0_survivor |
| MYOG | 3.34E-04 | 1.47 | Sepsis_D0_non-survivor up vs Sepsis_D0_survivor |
| MRGPRE | 1.86E-03 | 1.47 | Sepsis_D0_non-survivor up vs Sepsis_D0_survivor |
| SFTPA1 | 4.40E-03 | 1.47 | Sepsis_D0_non-survivor up vs Sepsis_D0_survivor |
| E2F6 | 7.28E-03 | 1.47 | Sepsis_D0_non-survivor up vs Sepsis_D0_survivor |
| GFRA2 | 3.16E-04 | 1.48 | Sepsis_D0_non-survivor up vs Sepsis_D0_survivor |
| RBM19 | 1.32E-03 | 1.48 | Sepsis_D0_non-survivor up vs Sepsis_D0_survivor |
| PRRT1 | 1.47E-03 | 1.48 | Sepsis_D0_non-survivor up vs Sepsis_D0_survivor |
| LSP1 | 6.13E-03 | 1.48 | Sepsis_D0_non-survivor up vs Sepsis_D0_survivor |
| ARMC9 | 1.81E-04 | 1.48 | Sepsis_D0_non-survivor up vs Sepsis_D0_survivor |
| CPLX2 | 3.96E-04 | 1.48 | Sepsis_D0_non-survivor up vs Sepsis_D0_survivor |
| IL1F7 | 2.85E-03 | 1.48 | Sepsis_D0_non-survivor up vs Sepsis_D0_survivor |
| RTDR1 | 5.28E-03 | 1.48 | Sepsis_D0_non-survivor up vs Sepsis_D0_survivor |
| TNNT3 | 3.51E-04 | 1.48 | Sepsis_D0_non-survivor up vs Sepsis_D0_survivor |
| MAPK11 | 6.93E-03 | 1.48 | Sepsis_D0_non-survivor up vs Sepsis_D0_survivor |
| MAP3K10 | 7.12E-04 | 1.48 | Sepsis_D0_non-survivor up vs Sepsis_D0_survivor |
| KLK9 | 2.58E-03 | 1.48 | Sepsis_D0_non-survivor up vs Sepsis_D0_survivor |
| PPP1R3F | 2.35E-04 | 1.48 | Sepsis_D0_non-survivor up vs Sepsis_D0_survivor |
| PRR7 | 3.21E-03 | 1.48 | Sepsis_D0_non-survivor up vs Sepsis_D0_survivor |
| LBX2 | 7.29E-04 | 1.48 | Sepsis_D0_non-survivor up vs Sepsis_D0_survivor |
| SDCBP2 | 1.39E-03 | 1.48 | Sepsis_D0_non-survivor up vs Sepsis_D0_survivor |
| AP1S1 | 4.04E-03 | 1.48 | Sepsis_D0_non-survivor up vs Sepsis_D0_survivor |
| S100A16 | 6.63E-03 | 1.48 | Sepsis_D0_non-survivor up vs Sepsis_D0_survivor |
| MYBL2 | 5.97E-04 | 1.48 | Sepsis_D0_non-survivor up vs Sepsis_D0_survivor |
| PAX7 | 1.71E-03 | 1.48 | Sepsis_D0_non-survivor up vs Sepsis_D0_survivor |
| OR2H2 | 7.22E-03 | 1.48 | Sepsis_D0_non-survivor up vs Sepsis_D0_survivor |
| OBSCN | 5.61E-03 | 1.48 | Sepsis_D0_non-survivor up vs Sepsis_D0_survivor |
| CTRC | 5.02E-03 | 1.48 | Sepsis_D0_non-survivor up vs Sepsis_D0_survivor |
| NXF2 | 1.80E-03 | 1.48 | Sepsis_D0_non-survivor up vs Sepsis_D0_survivor |
| F8 | 6.11E-04 | 1.48 | Sepsis_D0_non-survivor up vs Sepsis_D0_survivor |
| SLC28A1 | 1.67E-03 | 1.48 | Sepsis_D0_non-survivor up vs Sepsis_D0_survivor |
| ANKRD6 | 1.55E-03 | 1.48 | Sepsis_D0_non-survivor up vs Sepsis_D0_survivor |
| KCNC2 | 8.67E-05 | 1.49 | Sepsis_D0_non-survivor up vs Sepsis_D0_survivor |
| TETRAN | 7.56E-03 | 1.49 | Sepsis_D0_non-survivor up vs Sepsis_D0_survivor |
| SLC17A7 | 2.64E-03 | 1.49 | Sepsis_D0_non-survivor up vs Sepsis_D0_survivor |
| SSTR2 | 5.77E-03 | 1.49 | Sepsis_D0_non-survivor up vs Sepsis_D0_survivor |
| MAP2K7 | 1.62E-03 | 1.49 | Sepsis_D0_non-survivor up vs Sepsis_D0_survivor |
| ABHD4 | 7.29E-04 | 1.49 | Sepsis_D0_non-survivor up vs Sepsis_D0_survivor |
| IRX4 | 4.85E-04 | 1.49 | Sepsis_D0_non-survivor up vs Sepsis_D0_survivor |
| SSX3 | 2.65E-04 | 1.49 | Sepsis_D0_non-survivor up vs Sepsis_D0_survivor |
| UBE2G2 | 4.46E-03 | 1.49 | Sepsis_D0_non-survivor up vs Sepsis_D0_survivor |
| TBC1D20 | 2.94E-06 | 1.49 | Sepsis_D0_non-survivor up vs Sepsis_D0_survivor |
| GALNT9 | 1.68E-03 | 1.49 | Sepsis_D0_non-survivor up vs Sepsis_D0_survivor |
| MXRA8 | 3.43E-03 | 1.49 | Sepsis_D0_non-survivor up vs Sepsis_D0_survivor |
| CCDC71 | 7.17E-04 | 1.49 | Sepsis_D0_non-survivor up vs Sepsis_D0_survivor |
| CRYBB2 | 1.97E-03 | 1.49 | Sepsis_D0_non-survivor up vs Sepsis_D0_survivor |
| LRRC19 | 1.16E-03 | 1.49 | Sepsis_D0_non-survivor up vs Sepsis_D0_survivor |
| CNIH2 | 1.07E-03 | 1.49 | Sepsis_D0_non-survivor up vs Sepsis_D0_survivor |
| MAMDC4 | 1.67E-03 | 1.49 | Sepsis_D0_non-survivor up vs Sepsis_D0_survivor |
| MATN4 | 7.62E-03 | 1.49 | Sepsis_D0_non-survivor up vs Sepsis_D0_survivor |
| PON3 | 3.30E-03 | 1.49 | Sepsis_D0_non-survivor up vs Sepsis_D0_survivor |
| GPR6 | 6.89E-03 | 1.49 | Sepsis_D0_non-survivor up vs Sepsis_D0_survivor |
| CCL24 | 2.49E-03 | 1.49 | Sepsis_D0_non-survivor up vs Sepsis_D0_survivor |
| VGF | 2.52E-03 | 1.49 | Sepsis_D0_non-survivor up vs Sepsis_D0_survivor |
| PKLR | 3.39E-03 | 1.50 | Sepsis_D0_non-survivor up vs Sepsis_D0_survivor |
| PCYT2 | 2.25E-04 | 1.50 | Sepsis_D0_non-survivor up vs Sepsis_D0_survivor |
| RELB | 3.29E-04 | 1.50 | Sepsis_D0_non-survivor up vs Sepsis_D0_survivor |
| FCHSD1 | 1.93E-03 | 1.50 | Sepsis_D0_non-survivor up vs Sepsis_D0_survivor |
| Z21967 | 3.46E-03 | 1.50 | Sepsis_D0_non-survivor up vs Sepsis_D0_survivor |
| NKX2-3 | 3.97E-03 | 1.50 | Sepsis_D0_non-survivor up vs Sepsis_D0_survivor |
| SMARCC2 | 5.88E-04 | 1.50 | Sepsis_D0_non-survivor up vs Sepsis_D0_survivor |
| TUSC5 | 6.90E-03 | 1.50 | Sepsis_D0_non-survivor up vs Sepsis_D0_survivor |
| OXER1 | 1.19E-03 | 1.50 | Sepsis_D0_non-survivor up vs Sepsis_D0_survivor |
| NEIL1 | 2.22E-03 | 1.50 | Sepsis_D0_non-survivor up vs Sepsis_D0_survivor |
| TNFRSF9 | 1.87E-03 | 1.50 | Sepsis_D0_non-survivor up vs Sepsis_D0_survivor |
| TFAP2D | 7.15E-03 | 1.50 | Sepsis_D0_non-survivor up vs Sepsis_D0_survivor |
| UPK3A | 7.60E-04 | 1.50 | Sepsis_D0_non-survivor up vs Sepsis_D0_survivor |
| GJA4 | 4.46E-03 | 1.50 | Sepsis_D0_non-survivor up vs Sepsis_D0_survivor |
| SLC37A4 | 2.57E-03 | 1.50 | Sepsis_D0_non-survivor up vs Sepsis_D0_survivor |
| RLTPR | 6.42E-03 | 1.50 | Sepsis_D0_non-survivor up vs Sepsis_D0_survivor |
| KLB | 7.64E-03 | 1.50 | Sepsis_D0_non-survivor up vs Sepsis_D0_survivor |
| MMP15 | 2.80E-03 | 1.50 | Sepsis_D0_non-survivor up vs Sepsis_D0_survivor |
| CDH12 | 2.36E-03 | 1.50 | Sepsis_D0_non-survivor up vs Sepsis_D0_survivor |
| OR5H1 | 1.87E-03 | 1.50 | Sepsis_D0_non-survivor up vs Sepsis_D0_survivor |
| IXL | 1.30E-04 | 1.50 | Sepsis_D0_non-survivor up vs Sepsis_D0_survivor |
| OR1F2 | 3.27E-04 | 1.50 | Sepsis_D0_non-survivor up vs Sepsis_D0_survivor |
| UGT2B28 | 1.06E-03 | 1.51 | Sepsis_D0_non-survivor up vs Sepsis_D0_survivor |
| ALDH1B1 | 1.29E-04 | 1.51 | Sepsis_D0_non-survivor up vs Sepsis_D0_survivor |
| TM7SF2 | 6.36E-06 | 1.51 | Sepsis_D0_non-survivor up vs Sepsis_D0_survivor |
| FOXE1 | 5.20E-04 | 1.51 | Sepsis_D0_non-survivor up vs Sepsis_D0_survivor |
| hCAP-H2 | 4.43E-03 | 1.51 | Sepsis_D0_non-survivor up vs Sepsis_D0_survivor |
| DEFB4 | 2.70E-03 | 1.51 | Sepsis_D0_non-survivor up vs Sepsis_D0_survivor |
| DRD2 | 1.47E-04 | 1.51 | Sepsis_D0_non-survivor up vs Sepsis_D0_survivor |
| NOC4L | 2.70E-05 | 1.51 | Sepsis_D0_non-survivor up vs Sepsis_D0_survivor |
| BCAN | 5.42E-03 | 1.51 | Sepsis_D0_non-survivor up vs Sepsis_D0_survivor |
| SCGN | 2.06E-03 | 1.51 | Sepsis_D0_non-survivor up vs Sepsis_D0_survivor |
| NELF | 1.39E-03 | 1.51 | Sepsis_D0_non-survivor up vs Sepsis_D0_survivor |
| HOXB13 | 5.96E-03 | 1.51 | Sepsis_D0_non-survivor up vs Sepsis_D0_survivor |
| TMEM30B | 5.37E-04 | 1.51 | Sepsis_D0_non-survivor up vs Sepsis_D0_survivor |
| COL9A1 | 6.76E-04 | 1.51 | Sepsis_D0_non-survivor up vs Sepsis_D0_survivor |
| TRPM3 | 1.18E-03 | 1.51 | Sepsis_D0_non-survivor up vs Sepsis_D0_survivor |
| MLLT1 | 1.59E-04 | 1.51 | Sepsis_D0_non-survivor up vs Sepsis_D0_survivor |
| P2RY4 | 6.89E-03 | 1.51 | Sepsis_D0_non-survivor up vs Sepsis_D0_survivor |
| TTC28 | 7.52E-03 | 1.51 | Sepsis_D0_non-survivor up vs Sepsis_D0_survivor |
| GUCA1A | 2.86E-05 | 1.52 | Sepsis_D0_non-survivor up vs Sepsis_D0_survivor |
| SLC2A8 | 5.71E-03 | 1.52 | Sepsis_D0_non-survivor up vs Sepsis_D0_survivor |
| INHBC | 1.69E-03 | 1.52 | Sepsis_D0_non-survivor up vs Sepsis_D0_survivor |
| MMRN2 | 3.38E-04 | 1.52 | Sepsis_D0_non-survivor up vs Sepsis_D0_survivor |
| PTF1A | 9.83E-05 | 1.52 | Sepsis_D0_non-survivor up vs Sepsis_D0_survivor |
| MON1B | 9.60E-05 | 1.52 | Sepsis_D0_non-survivor up vs Sepsis_D0_survivor |
| AQP2 | 2.46E-03 | 1.52 | Sepsis_D0_non-survivor up vs Sepsis_D0_survivor |
| PLXNA3 | 4.74E-06 | 1.52 | Sepsis_D0_non-survivor up vs Sepsis_D0_survivor |
| ZNF645 | 1.44E-03 | 1.52 | Sepsis_D0_non-survivor up vs Sepsis_D0_survivor |
| CHST10 | 1.31E-03 | 1.52 | Sepsis_D0_non-survivor up vs Sepsis_D0_survivor |
| CCL3L3 | 3.61E-03 | 1.52 | Sepsis_D0_non-survivor up vs Sepsis_D0_survivor |
| RFNG | 9.34E-04 | 1.52 | Sepsis_D0_non-survivor up vs Sepsis_D0_survivor |
| INS | 1.72E-03 | 1.52 | Sepsis_D0_non-survivor up vs Sepsis_D0_survivor |
| CHST8 | 5.61E-04 | 1.52 | Sepsis_D0_non-survivor up vs Sepsis_D0_survivor |
| SEMA3B | 2.12E-03 | 1.52 | Sepsis_D0_non-survivor up vs Sepsis_D0_survivor |
| LCE3B | 1.22E-03 | 1.52 | Sepsis_D0_non-survivor up vs Sepsis_D0_survivor |
| STC2 | 2.53E-03 | 1.52 | Sepsis_D0_non-survivor up vs Sepsis_D0_survivor |
| LSR | 2.55E-03 | 1.52 | Sepsis_D0_non-survivor up vs Sepsis_D0_survivor |
| LILRA1 | 7.95E-06 | 1.52 | Sepsis_D0_non-survivor up vs Sepsis_D0_survivor |
| CD7 | 7.00E-04 | 1.52 | Sepsis_D0_non-survivor up vs Sepsis_D0_survivor |
| ELA2B | 1.42E-03 | 1.53 | Sepsis_D0_non-survivor up vs Sepsis_D0_survivor |
| ALG12 | 5.08E-03 | 1.53 | Sepsis_D0_non-survivor up vs Sepsis_D0_survivor |
| CSPG4LYP1 | 3.23E-03 | 1.53 | Sepsis_D0_non-survivor up vs Sepsis_D0_survivor |
| FAM71A | 2.02E-04 | 1.53 | Sepsis_D0_non-survivor up vs Sepsis_D0_survivor |
| ATXN7L2 | 1.15E-03 | 1.53 | Sepsis_D0_non-survivor up vs Sepsis_D0_survivor |
| ALPI | 3.74E-05 | 1.53 | Sepsis_D0_non-survivor up vs Sepsis_D0_survivor |
| SPDEF | 2.32E-03 | 1.53 | Sepsis_D0_non-survivor up vs Sepsis_D0_survivor |
| TIAM1 | 2.55E-03 | 1.53 | Sepsis_D0_non-survivor up vs Sepsis_D0_survivor |
| MT3 | 3.18E-03 | 1.53 | Sepsis_D0_non-survivor up vs Sepsis_D0_survivor |
| TRIM35 | 1.48E-03 | 1.53 | Sepsis_D0_non-survivor up vs Sepsis_D0_survivor |
| PAK6 | 2.05E-03 | 1.53 | Sepsis_D0_non-survivor up vs Sepsis_D0_survivor |
| NFKBIL2 | 2.03E-04 | 1.53 | Sepsis_D0_non-survivor up vs Sepsis_D0_survivor |
| GPHA2 | 1.86E-03 | 1.53 | Sepsis_D0_non-survivor up vs Sepsis_D0_survivor |
| PLEKHB1 | 7.93E-04 | 1.53 | Sepsis_D0_non-survivor up vs Sepsis_D0_survivor |
| PLA2G4F | 2.65E-03 | 1.53 | Sepsis_D0_non-survivor up vs Sepsis_D0_survivor |
| RHO | 2.37E-04 | 1.53 | Sepsis_D0_non-survivor up vs Sepsis_D0_survivor |
| HS3ST3B1 | 1.56E-03 | 1.53 | Sepsis_D0_non-survivor up vs Sepsis_D0_survivor |
| NAT9 | 1.04E-03 | 1.53 | Sepsis_D0_non-survivor up vs Sepsis_D0_survivor |
| MORN1 | 3.40E-03 | 1.53 | Sepsis_D0_non-survivor up vs Sepsis_D0_survivor |
| MSI1 | 5.81E-03 | 1.53 | Sepsis_D0_non-survivor up vs Sepsis_D0_survivor |
| FGF3 | 2.73E-03 | 1.53 | Sepsis_D0_non-survivor up vs Sepsis_D0_survivor |
| ADAM7 | 6.96E-03 | 1.53 | Sepsis_D0_non-survivor up vs Sepsis_D0_survivor |
| AGC1 | 1.18E-03 | 1.53 | Sepsis_D0_non-survivor up vs Sepsis_D0_survivor |
| CAST1 | 5.02E-03 | 1.54 | Sepsis_D0_non-survivor up vs Sepsis_D0_survivor |
| GSTA4 | 1.34E-03 | 1.54 | Sepsis_D0_non-survivor up vs Sepsis_D0_survivor |
| TGM7 | 7.61E-04 | 1.54 | Sepsis_D0_non-survivor up vs Sepsis_D0_survivor |
| KCNJ12 | 3.32E-03 | 1.54 | Sepsis_D0_non-survivor up vs Sepsis_D0_survivor |
| PRAF2 | 2.09E-04 | 1.54 | Sepsis_D0_non-survivor up vs Sepsis_D0_survivor |
| PELP1 | 4.22E-03 | 1.54 | Sepsis_D0_non-survivor up vs Sepsis_D0_survivor |
| HIST1H2AA | 4.06E-03 | 1.54 | Sepsis_D0_non-survivor up vs Sepsis_D0_survivor |
| PPP1R9B | 5.14E-03 | 1.54 | Sepsis_D0_non-survivor up vs Sepsis_D0_survivor |
| CCL25 | 1.59E-04 | 1.54 | Sepsis_D0_non-survivor up vs Sepsis_D0_survivor |
| S100A7 | 5.07E-03 | 1.54 | Sepsis_D0_non-survivor up vs Sepsis_D0_survivor |
| EPB41L5 | 5.76E-03 | 1.55 | Sepsis_D0_non-survivor up vs Sepsis_D0_survivor |
| CHDH | 1.08E-03 | 1.55 | Sepsis_D0_non-survivor up vs Sepsis_D0_survivor |
| NOTCH3 | 2.53E-03 | 1.55 | Sepsis_D0_non-survivor up vs Sepsis_D0_survivor |
| TNAP | 4.22E-03 | 1.55 | Sepsis_D0_non-survivor up vs Sepsis_D0_survivor |
| RBMS3 | 3.85E-03 | 1.55 | Sepsis_D0_non-survivor up vs Sepsis_D0_survivor |
| BCL6B | 5.14E-04 | 1.55 | Sepsis_D0_non-survivor up vs Sepsis_D0_survivor |
| ADAM33 | 5.23E-04 | 1.55 | Sepsis_D0_non-survivor up vs Sepsis_D0_survivor |
| SLCO6A1 | 2.27E-04 | 1.55 | Sepsis_D0_non-survivor up vs Sepsis_D0_survivor |
| HAGHL | 3.43E-04 | 1.55 | Sepsis_D0_non-survivor up vs Sepsis_D0_survivor |
| RGS4 | 4.29E-03 | 1.55 | Sepsis_D0_non-survivor up vs Sepsis_D0_survivor |
| TBC1D10B | 6.09E-05 | 1.55 | Sepsis_D0_non-survivor up vs Sepsis_D0_survivor |
| RNF157 | 8.36E-04 | 1.55 | Sepsis_D0_non-survivor up vs Sepsis_D0_survivor |
| NR2E1 | 7.09E-03 | 1.55 | Sepsis_D0_non-survivor up vs Sepsis_D0_survivor |
| PES1 | 1.78E-03 | 1.55 | Sepsis_D0_non-survivor up vs Sepsis_D0_survivor |
| CRHR1 | 4.72E-03 | 1.55 | Sepsis_D0_non-survivor up vs Sepsis_D0_survivor |
| ZFYVE9 | 6.03E-03 | 1.55 | Sepsis_D0_non-survivor up vs Sepsis_D0_survivor |
| BTBD14B | 3.03E-04 | 1.55 | Sepsis_D0_non-survivor up vs Sepsis_D0_survivor |
| NRSN2 | 1.05E-04 | 1.55 | Sepsis_D0_non-survivor up vs Sepsis_D0_survivor |
| PRDM4 | 2.23E-04 | 1.55 | Sepsis_D0_non-survivor up vs Sepsis_D0_survivor |
| TRIM74 | 6.80E-03 | 1.55 | Sepsis_D0_non-survivor up vs Sepsis_D0_survivor |
| IFNA5 | 6.96E-03 | 1.56 | Sepsis_D0_non-survivor up vs Sepsis_D0_survivor |
| ZNF342 | 2.78E-04 | 1.56 | Sepsis_D0_non-survivor up vs Sepsis_D0_survivor |
| KRTAP9-8 | 3.75E-03 | 1.56 | Sepsis_D0_non-survivor up vs Sepsis_D0_survivor |
| MCM2 | 1.71E-03 | 1.56 | Sepsis_D0_non-survivor up vs Sepsis_D0_survivor |
| ZNF533 | 9.22E-04 | 1.56 | Sepsis_D0_non-survivor up vs Sepsis_D0_survivor |
| CNN2 | 7.05E-04 | 1.56 | Sepsis_D0_non-survivor up vs Sepsis_D0_survivor |
| SYN1 | 1.36E-03 | 1.56 | Sepsis_D0_non-survivor up vs Sepsis_D0_survivor |
| MYCN | 1.21E-03 | 1.56 | Sepsis_D0_non-survivor up vs Sepsis_D0_survivor |
| RGS11 | 1.01E-03 | 1.56 | Sepsis_D0_non-survivor up vs Sepsis_D0_survivor |
| SPPL2B | 3.66E-03 | 1.56 | Sepsis_D0_non-survivor up vs Sepsis_D0_survivor |
| MYBBP1A | 3.63E-04 | 1.56 | Sepsis_D0_non-survivor up vs Sepsis_D0_survivor |
| RXFP4 | 4.61E-03 | 1.56 | Sepsis_D0_non-survivor up vs Sepsis_D0_survivor |
| DEFA1 | 2.32E-03 | 1.56 | Sepsis_D0_non-survivor up vs Sepsis_D0_survivor |
| OTUD7A | 1.53E-03 | 1.56 | Sepsis_D0_non-survivor up vs Sepsis_D0_survivor |
| GAMT | 1.28E-03 | 1.56 | Sepsis_D0_non-survivor up vs Sepsis_D0_survivor |
| KHSRP | 6.32E-03 | 1.56 | Sepsis_D0_non-survivor up vs Sepsis_D0_survivor |
| CTRL | 2.84E-03 | 1.56 | Sepsis_D0_non-survivor up vs Sepsis_D0_survivor |
| SSPN | 1.19E-03 | 1.56 | Sepsis_D0_non-survivor up vs Sepsis_D0_survivor |
| VAMP2 | 1.53E-03 | 1.57 | Sepsis_D0_non-survivor up vs Sepsis_D0_survivor |
| FAM113A | 7.34E-05 | 1.57 | Sepsis_D0_non-survivor up vs Sepsis_D0_survivor |
| APOE | 9.89E-07 | 1.57 | Sepsis_D0_non-survivor up vs Sepsis_D0_survivor |
| KIF12 | 3.16E-03 | 1.57 | Sepsis_D0_non-survivor up vs Sepsis_D0_survivor |
| ZBTB22 | 8.56E-05 | 1.57 | Sepsis_D0_non-survivor up vs Sepsis_D0_survivor |
| OTOS | 7.43E-03 | 1.57 | Sepsis_D0_non-survivor up vs Sepsis_D0_survivor |
| MAP1S | 5.31E-03 | 1.57 | Sepsis_D0_non-survivor up vs Sepsis_D0_survivor |
| EDG5 | 2.26E-04 | 1.57 | Sepsis_D0_non-survivor up vs Sepsis_D0_survivor |
| CPAMD8 | 2.83E-04 | 1.57 | Sepsis_D0_non-survivor up vs Sepsis_D0_survivor |
| HDAC4 | 3.76E-05 | 1.57 | Sepsis_D0_non-survivor up vs Sepsis_D0_survivor |
| IL23R | 3.11E-03 | 1.57 | Sepsis_D0_non-survivor up vs Sepsis_D0_survivor |
| CYP2W1 | 3.33E-03 | 1.57 | Sepsis_D0_non-survivor up vs Sepsis_D0_survivor |
| OAZ1 | 1.71E-03 | 1.57 | Sepsis_D0_non-survivor up vs Sepsis_D0_survivor |
| OR10C1 | 2.79E-03 | 1.57 | Sepsis_D0_non-survivor up vs Sepsis_D0_survivor |
| CLTCL1 | 3.46E-03 | 1.57 | Sepsis_D0_non-survivor up vs Sepsis_D0_survivor |
| GRID2 | 1.19E-03 | 1.57 | Sepsis_D0_non-survivor up vs Sepsis_D0_survivor |
| RPH3AL | 4.18E-03 | 1.57 | Sepsis_D0_non-survivor up vs Sepsis_D0_survivor |
| COL27A1 | 8.86E-05 | 1.57 | Sepsis_D0_non-survivor up vs Sepsis_D0_survivor |
| ODF4 | 2.60E-03 | 1.57 | Sepsis_D0_non-survivor up vs Sepsis_D0_survivor |
| CHRNA4 | 6.61E-04 | 1.57 | Sepsis_D0_non-survivor up vs Sepsis_D0_survivor |
| TANC2 | 9.47E-04 | 1.57 | Sepsis_D0_non-survivor up vs Sepsis_D0_survivor |
| S100A3 | 7.72E-05 | 1.57 | Sepsis_D0_non-survivor up vs Sepsis_D0_survivor |
| CEACAM19 | 3.11E-04 | 1.57 | Sepsis_D0_non-survivor up vs Sepsis_D0_survivor |
| COX4I2 | 3.28E-03 | 1.57 | Sepsis_D0_non-survivor up vs Sepsis_D0_survivor |
| GPR61 | 7.52E-03 | 1.58 | Sepsis_D0_non-survivor up vs Sepsis_D0_survivor |
| POLM | 5.84E-04 | 1.58 | Sepsis_D0_non-survivor up vs Sepsis_D0_survivor |
| RHBDL1 | 7.37E-03 | 1.58 | Sepsis_D0_non-survivor up vs Sepsis_D0_survivor |
| KY | 1.67E-03 | 1.58 | Sepsis_D0_non-survivor up vs Sepsis_D0_survivor |
| RTN3 | 8.63E-05 | 1.58 | Sepsis_D0_non-survivor up vs Sepsis_D0_survivor |
| COMP | 2.73E-05 | 1.58 | Sepsis_D0_non-survivor up vs Sepsis_D0_survivor |
| NTNG1 | 4.39E-03 | 1.58 | Sepsis_D0_non-survivor up vs Sepsis_D0_survivor |
| OR10H2 | 2.62E-03 | 1.58 | Sepsis_D0_non-survivor up vs Sepsis_D0_survivor |
| IL1B | 9.96E-04 | 1.58 | Sepsis_D0_non-survivor up vs Sepsis_D0_survivor |
| UBD | 3.04E-03 | 1.58 | Sepsis_D0_non-survivor up vs Sepsis_D0_survivor |
| FAM120C | 4.32E-04 | 1.58 | Sepsis_D0_non-survivor up vs Sepsis_D0_survivor |
| TMEM28 | 2.82E-04 | 1.58 | Sepsis_D0_non-survivor up vs Sepsis_D0_survivor |
| EMILIN3 | 2.45E-03 | 1.58 | Sepsis_D0_non-survivor up vs Sepsis_D0_survivor |
| GPR144 | 9.09E-05 | 1.58 | Sepsis_D0_non-survivor up vs Sepsis_D0_survivor |
| GNL1 | 1.15E-03 | 1.58 | Sepsis_D0_non-survivor up vs Sepsis_D0_survivor |
| HSPBP1 | 4.10E-04 | 1.58 | Sepsis_D0_non-survivor up vs Sepsis_D0_survivor |
| IL28A | 2.48E-05 | 1.58 | Sepsis_D0_non-survivor up vs Sepsis_D0_survivor |
| CALCB | 1.06E-03 | 1.58 | Sepsis_D0_non-survivor up vs Sepsis_D0_survivor |
| SLC22A18AS | 7.04E-05 | 1.58 | Sepsis_D0_non-survivor up vs Sepsis_D0_survivor |
| KCNJ3 | 7.66E-04 | 1.58 | Sepsis_D0_non-survivor up vs Sepsis_D0_survivor |
| ZNF703 | 1.20E-05 | 1.59 | Sepsis_D0_non-survivor up vs Sepsis_D0_survivor |
| ITLN2 | 3.14E-03 | 1.59 | Sepsis_D0_non-survivor up vs Sepsis_D0_survivor |
| CEP72 | 1.13E-03 | 1.59 | Sepsis_D0_non-survivor up vs Sepsis_D0_survivor |
| HKR2 | 1.26E-03 | 1.59 | Sepsis_D0_non-survivor up vs Sepsis_D0_survivor |
| OGFR | 1.62E-03 | 1.59 | Sepsis_D0_non-survivor up vs Sepsis_D0_survivor |
| MAPK8IP1 | 4.18E-04 | 1.59 | Sepsis_D0_non-survivor up vs Sepsis_D0_survivor |
| ODF1 | 7.35E-03 | 1.59 | Sepsis_D0_non-survivor up vs Sepsis_D0_survivor |
| SPRR3 | 1.94E-03 | 1.59 | Sepsis_D0_non-survivor up vs Sepsis_D0_survivor |
| CYP46A1 | 3.95E-03 | 1.59 | Sepsis_D0_non-survivor up vs Sepsis_D0_survivor |
| PLOD3 | 3.72E-05 | 1.59 | Sepsis_D0_non-survivor up vs Sepsis_D0_survivor |
| TP53TG3 | 5.37E-03 | 1.59 | Sepsis_D0_non-survivor up vs Sepsis_D0_survivor |
| R3HDM2 | 2.32E-04 | 1.59 | Sepsis_D0_non-survivor up vs Sepsis_D0_survivor |
| FOXI1 | 3.64E-03 | 1.59 | Sepsis_D0_non-survivor up vs Sepsis_D0_survivor |
| ZNF574 | 8.89E-04 | 1.59 | Sepsis_D0_non-survivor up vs Sepsis_D0_survivor |
| ADRB3 | 1.08E-04 | 1.59 | Sepsis_D0_non-survivor up vs Sepsis_D0_survivor |
| P2RY11 | 5.41E-03 | 1.59 | Sepsis_D0_non-survivor up vs Sepsis_D0_survivor |
| Y10152 | 2.22E-03 | 1.60 | Sepsis_D0_non-survivor up vs Sepsis_D0_survivor |
| SHARPIN | 1.11E-04 | 1.60 | Sepsis_D0_non-survivor up vs Sepsis_D0_survivor |
| SRM | 3.67E-03 | 1.60 | Sepsis_D0_non-survivor up vs Sepsis_D0_survivor |
| LRFN2 | 1.27E-03 | 1.60 | Sepsis_D0_non-survivor up vs Sepsis_D0_survivor |
| DPT | 1.34E-04 | 1.60 | Sepsis_D0_non-survivor up vs Sepsis_D0_survivor |
| MEF2B | 2.45E-04 | 1.60 | Sepsis_D0_non-survivor up vs Sepsis_D0_survivor |
| ALPP | 7.27E-04 | 1.60 | Sepsis_D0_non-survivor up vs Sepsis_D0_survivor |
| LTBP3 | 1.49E-03 | 1.60 | Sepsis_D0_non-survivor up vs Sepsis_D0_survivor |
| RAVER1 | 4.44E-04 | 1.60 | Sepsis_D0_non-survivor up vs Sepsis_D0_survivor |
| BSPRY | 5.25E-03 | 1.60 | Sepsis_D0_non-survivor up vs Sepsis_D0_survivor |
| POLR3A | 1.97E-03 | 1.60 | Sepsis_D0_non-survivor up vs Sepsis_D0_survivor |
| KRT14 | 2.64E-04 | 1.60 | Sepsis_D0_non-survivor up vs Sepsis_D0_survivor |
| FOXQ1 | 1.68E-03 | 1.60 | Sepsis_D0_non-survivor up vs Sepsis_D0_survivor |
| CASKIN2 | 1.17E-03 | 1.60 | Sepsis_D0_non-survivor up vs Sepsis_D0_survivor |
| CACNA2D1 | 5.26E-03 | 1.60 | Sepsis_D0_non-survivor up vs Sepsis_D0_survivor |
| TMIGD2 | 1.26E-03 | 1.61 | Sepsis_D0_non-survivor up vs Sepsis_D0_survivor |
| MAFA | 1.04E-03 | 1.61 | Sepsis_D0_non-survivor up vs Sepsis_D0_survivor |
| MADCAM1 | 2.94E-04 | 1.61 | Sepsis_D0_non-survivor up vs Sepsis_D0_survivor |
| EN2 | 2.56E-03 | 1.61 | Sepsis_D0_non-survivor up vs Sepsis_D0_survivor |
| TMSL3 | 1.59E-04 | 1.61 | Sepsis_D0_non-survivor up vs Sepsis_D0_survivor |
| NDOR1 | 1.15E-03 | 1.61 | Sepsis_D0_non-survivor up vs Sepsis_D0_survivor |
| RKHD1 | 5.39E-03 | 1.61 | Sepsis_D0_non-survivor up vs Sepsis_D0_survivor |
| EML2 | 1.61E-04 | 1.61 | Sepsis_D0_non-survivor up vs Sepsis_D0_survivor |
| SPRY4 | 2.80E-03 | 1.61 | Sepsis_D0_non-survivor up vs Sepsis_D0_survivor |
| ENTPD2 | 3.38E-03 | 1.61 | Sepsis_D0_non-survivor up vs Sepsis_D0_survivor |
| ALPPL2 | 1.44E-03 | 1.61 | Sepsis_D0_non-survivor up vs Sepsis_D0_survivor |
| YIF1B | 6.77E-05 | 1.61 | Sepsis_D0_non-survivor up vs Sepsis_D0_survivor |
| UNQ9438 | 5.50E-05 | 1.61 | Sepsis_D0_non-survivor up vs Sepsis_D0_survivor |
| ADAMTSL4 | 1.34E-03 | 1.61 | Sepsis_D0_non-survivor up vs Sepsis_D0_survivor |
| LMOD1 | 8.56E-04 | 1.62 | Sepsis_D0_non-survivor up vs Sepsis_D0_survivor |
| DMWD | 1.12E-03 | 1.62 | Sepsis_D0_non-survivor up vs Sepsis_D0_survivor |
| OR1F1 | 3.52E-04 | 1.62 | Sepsis_D0_non-survivor up vs Sepsis_D0_survivor |
| CDX1 | 3.33E-03 | 1.62 | Sepsis_D0_non-survivor up vs Sepsis_D0_survivor |
| TBC1D17 | 2.17E-04 | 1.62 | Sepsis_D0_non-survivor up vs Sepsis_D0_survivor |
| LGR4 | 3.19E-03 | 1.62 | Sepsis_D0_non-survivor up vs Sepsis_D0_survivor |
| ZNF506 | 1.39E-03 | 1.62 | Sepsis_D0_non-survivor up vs Sepsis_D0_survivor |
| ADCY2 | 5.33E-03 | 1.62 | Sepsis_D0_non-survivor up vs Sepsis_D0_survivor |
| CPA5 | 7.59E-04 | 1.62 | Sepsis_D0_non-survivor up vs Sepsis_D0_survivor |
| SMC1B | 1.85E-03 | 1.62 | Sepsis_D0_non-survivor up vs Sepsis_D0_survivor |
| CDC42BPB | 1.56E-03 | 1.62 | Sepsis_D0_non-survivor up vs Sepsis_D0_survivor |
| FOXN1 | 2.28E-03 | 1.62 | Sepsis_D0_non-survivor up vs Sepsis_D0_survivor |
| WFDC10B | 2.24E-04 | 1.62 | Sepsis_D0_non-survivor up vs Sepsis_D0_survivor |
| CPN2 | 1.64E-04 | 1.62 | Sepsis_D0_non-survivor up vs Sepsis_D0_survivor |
| LENG8 | 1.75E-04 | 1.62 | Sepsis_D0_non-survivor up vs Sepsis_D0_survivor |
| SNCG | 1.15E-03 | 1.62 | Sepsis_D0_non-survivor up vs Sepsis_D0_survivor |
| TMEM143 | 1.88E-03 | 1.62 | Sepsis_D0_non-survivor up vs Sepsis_D0_survivor |
| KRT31 | 4.65E-03 | 1.62 | Sepsis_D0_non-survivor up vs Sepsis_D0_survivor |
| P2RY6 | 9.97E-04 | 1.62 | Sepsis_D0_non-survivor up vs Sepsis_D0_survivor |
| PRB3 | 5.18E-04 | 1.62 | Sepsis_D0_non-survivor up vs Sepsis_D0_survivor |
| DGKQ | 2.30E-03 | 1.62 | Sepsis_D0_non-survivor up vs Sepsis_D0_survivor |
| TMEM63B | 2.42E-03 | 1.62 | Sepsis_D0_non-survivor up vs Sepsis_D0_survivor |
| SNTA1 | 1.38E-03 | 1.63 | Sepsis_D0_non-survivor up vs Sepsis_D0_survivor |
| CTXN1 | 9.62E-04 | 1.63 | Sepsis_D0_non-survivor up vs Sepsis_D0_survivor |
| PCDHB9 | 3.66E-04 | 1.63 | Sepsis_D0_non-survivor up vs Sepsis_D0_survivor |
| CLCN1 | 2.07E-04 | 1.63 | Sepsis_D0_non-survivor up vs Sepsis_D0_survivor |
| PSORS1C2 | 7.50E-04 | 1.63 | Sepsis_D0_non-survivor up vs Sepsis_D0_survivor |
| MAG | 8.70E-04 | 1.63 | Sepsis_D0_non-survivor up vs Sepsis_D0_survivor |
| DPEP3 | 3.18E-03 | 1.63 | Sepsis_D0_non-survivor up vs Sepsis_D0_survivor |
| FBXL7 | 1.06E-04 | 1.63 | Sepsis_D0_non-survivor up vs Sepsis_D0_survivor |
| CACNB2 | 5.07E-03 | 1.63 | Sepsis_D0_non-survivor up vs Sepsis_D0_survivor |
| OR10A2 | 7.29E-03 | 1.63 | Sepsis_D0_non-survivor up vs Sepsis_D0_survivor |
| ARL4D | 1.29E-03 | 1.63 | Sepsis_D0_non-survivor up vs Sepsis_D0_survivor |
| AFF3 | 3.32E-03 | 1.63 | Sepsis_D0_non-survivor up vs Sepsis_D0_survivor |
| CRYAA | 1.33E-03 | 1.63 | Sepsis_D0_non-survivor up vs Sepsis_D0_survivor |
| PAX5 | 1.32E-03 | 1.63 | Sepsis_D0_non-survivor up vs Sepsis_D0_survivor |
| UBTD1 | 3.13E-03 | 1.63 | Sepsis_D0_non-survivor up vs Sepsis_D0_survivor |
| APOL1 | 2.67E-03 | 1.63 | Sepsis_D0_non-survivor up vs Sepsis_D0_survivor |
| MFRP | 6.32E-04 | 1.63 | Sepsis_D0_non-survivor up vs Sepsis_D0_survivor |
| CD3EAP | 2.16E-03 | 1.63 | Sepsis_D0_non-survivor up vs Sepsis_D0_survivor |
| TMEM38A | 1.43E-03 | 1.63 | Sepsis_D0_non-survivor up vs Sepsis_D0_survivor |
| GDDR | 8.04E-04 | 1.64 | Sepsis_D0_non-survivor up vs Sepsis_D0_survivor |
| APOA4 | 1.64E-04 | 1.64 | Sepsis_D0_non-survivor up vs Sepsis_D0_survivor |
| PDLIM4 | 1.25E-03 | 1.64 | Sepsis_D0_non-survivor up vs Sepsis_D0_survivor |
| DLX3 | 4.00E-04 | 1.64 | Sepsis_D0_non-survivor up vs Sepsis_D0_survivor |
| LYNX1 | 1.73E-04 | 1.64 | Sepsis_D0_non-survivor up vs Sepsis_D0_survivor |
| CLIPR-59 | 6.21E-04 | 1.64 | Sepsis_D0_non-survivor up vs Sepsis_D0_survivor |
| SPRR1A | 1.20E-03 | 1.64 | Sepsis_D0_non-survivor up vs Sepsis_D0_survivor |
| DYRK1B | 7.40E-04 | 1.64 | Sepsis_D0_non-survivor up vs Sepsis_D0_survivor |
| LCAT | 3.70E-03 | 1.64 | Sepsis_D0_non-survivor up vs Sepsis_D0_survivor |
| ADAMTSL5 | 9.64E-04 | 1.64 | Sepsis_D0_non-survivor up vs Sepsis_D0_survivor |
| PAK4 | 5.56E-05 | 1.64 | Sepsis_D0_non-survivor up vs Sepsis_D0_survivor |
| ZG16 | 6.42E-03 | 1.64 | Sepsis_D0_non-survivor up vs Sepsis_D0_survivor |
| JMJD5 | 1.58E-03 | 1.64 | Sepsis_D0_non-survivor up vs Sepsis_D0_survivor |
| RASL12 | 1.26E-03 | 1.64 | Sepsis_D0_non-survivor up vs Sepsis_D0_survivor |
| SFTPA2 | 1.74E-04 | 1.64 | Sepsis_D0_non-survivor up vs Sepsis_D0_survivor |
| RASGRF2 | 5.90E-04 | 1.64 | Sepsis_D0_non-survivor up vs Sepsis_D0_survivor |
| MT1JP | 3.14E-03 | 1.64 | Sepsis_D0_non-survivor up vs Sepsis_D0_survivor |
| RIMBP2 | 2.05E-04 | 1.64 | Sepsis_D0_non-survivor up vs Sepsis_D0_survivor |
| TMEM130 | 1.65E-04 | 1.65 | Sepsis_D0_non-survivor up vs Sepsis_D0_survivor |
| NECAP1 | 3.17E-04 | 1.65 | Sepsis_D0_non-survivor up vs Sepsis_D0_survivor |
| CORO2B | 1.77E-03 | 1.65 | Sepsis_D0_non-survivor up vs Sepsis_D0_survivor |
| RBM35B | 2.56E-03 | 1.65 | Sepsis_D0_non-survivor up vs Sepsis_D0_survivor |
| P2RXL1 | 1.13E-03 | 1.65 | Sepsis_D0_non-survivor up vs Sepsis_D0_survivor |
| CP | 5.65E-03 | 1.65 | Sepsis_D0_non-survivor up vs Sepsis_D0_survivor |
| MUC5AC | 3.26E-05 | 1.65 | Sepsis_D0_non-survivor up vs Sepsis_D0_survivor |
| VCY | 1.15E-05 | 1.65 | Sepsis_D0_non-survivor up vs Sepsis_D0_survivor |
| HRG | 3.98E-04 | 1.65 | Sepsis_D0_non-survivor up vs Sepsis_D0_survivor |
| MYOZ2 | 4.04E-03 | 1.66 | Sepsis_D0_non-survivor up vs Sepsis_D0_survivor |
| UMOD | 8.67E-04 | 1.66 | Sepsis_D0_non-survivor up vs Sepsis_D0_survivor |
| RHBDL3 | 1.70E-03 | 1.66 | Sepsis_D0_non-survivor up vs Sepsis_D0_survivor |
| KRTAP10-10 | 3.08E-03 | 1.66 | Sepsis_D0_non-survivor up vs Sepsis_D0_survivor |
| HSD17B6 | 4.69E-03 | 1.66 | Sepsis_D0_non-survivor up vs Sepsis_D0_survivor |
| ILDR1 | 4.78E-04 | 1.66 | Sepsis_D0_non-survivor up vs Sepsis_D0_survivor |
| THEM5 | 4.50E-03 | 1.66 | Sepsis_D0_non-survivor up vs Sepsis_D0_survivor |
| P53AIP1 | 2.71E-04 | 1.66 | Sepsis_D0_non-survivor up vs Sepsis_D0_survivor |
| LCE3D | 1.29E-03 | 1.66 | Sepsis_D0_non-survivor up vs Sepsis_D0_survivor |
| TCF15 | 5.57E-04 | 1.66 | Sepsis_D0_non-survivor up vs Sepsis_D0_survivor |
| NOXO1 | 5.33E-03 | 1.66 | Sepsis_D0_non-survivor up vs Sepsis_D0_survivor |
| FKBP4 | 7.68E-04 | 1.66 | Sepsis_D0_non-survivor up vs Sepsis_D0_survivor |
| OR7E91P | 1.22E-03 | 1.66 | Sepsis_D0_non-survivor up vs Sepsis_D0_survivor |
| CAV3 | 5.40E-03 | 1.67 | Sepsis_D0_non-survivor up vs Sepsis_D0_survivor |
| PPP2R1A | 5.92E-04 | 1.67 | Sepsis_D0_non-survivor up vs Sepsis_D0_survivor |
| SARDH | 3.48E-03 | 1.67 | Sepsis_D0_non-survivor up vs Sepsis_D0_survivor |
| MIER2 | 6.63E-04 | 1.67 | Sepsis_D0_non-survivor up vs Sepsis_D0_survivor |
| CLDN19 | 7.50E-03 | 1.67 | Sepsis_D0_non-survivor up vs Sepsis_D0_survivor |
| PALM2-AKAP2 | 4.72E-05 | 1.67 | Sepsis_D0_non-survivor up vs Sepsis_D0_survivor |
| SLC5A2 | 5.42E-04 | 1.67 | Sepsis_D0_non-survivor up vs Sepsis_D0_survivor |
| GABRG3 | 1.60E-05 | 1.67 | Sepsis_D0_non-survivor up vs Sepsis_D0_survivor |
| ARL17P1 | 7.42E-03 | 1.67 | Sepsis_D0_non-survivor up vs Sepsis_D0_survivor |
| ARHGEF15 | 9.12E-04 | 1.67 | Sepsis_D0_non-survivor up vs Sepsis_D0_survivor |
| GNRH2 | 6.83E-05 | 1.67 | Sepsis_D0_non-survivor up vs Sepsis_D0_survivor |
| TACR2 | 1.55E-03 | 1.67 | Sepsis_D0_non-survivor up vs Sepsis_D0_survivor |
| KRTAP4-10 | 2.39E-03 | 1.67 | Sepsis_D0_non-survivor up vs Sepsis_D0_survivor |
| IGSF1 | 7.21E-03 | 1.67 | Sepsis_D0_non-survivor up vs Sepsis_D0_survivor |
| TNK2 | 3.41E-05 | 1.67 | Sepsis_D0_non-survivor up vs Sepsis_D0_survivor |
| TNFSF15 | 9.86E-04 | 1.68 | Sepsis_D0_non-survivor up vs Sepsis_D0_survivor |
| OR1D2 | 4.27E-03 | 1.68 | Sepsis_D0_non-survivor up vs Sepsis_D0_survivor |
| SLC34A3 | 1.06E-03 | 1.68 | Sepsis_D0_non-survivor up vs Sepsis_D0_survivor |
| MYOT | 1.91E-03 | 1.68 | Sepsis_D0_non-survivor up vs Sepsis_D0_survivor |
| S75896 | 1.12E-04 | 1.68 | Sepsis_D0_non-survivor up vs Sepsis_D0_survivor |
| OR2H1 | 2.10E-03 | 1.68 | Sepsis_D0_non-survivor up vs Sepsis_D0_survivor |
| HSD11B1L | 1.95E-05 | 1.68 | Sepsis_D0_non-survivor up vs Sepsis_D0_survivor |
| MID1 | 4.13E-04 | 1.68 | Sepsis_D0_non-survivor up vs Sepsis_D0_survivor |
| DNAJC5B | 2.01E-04 | 1.68 | Sepsis_D0_non-survivor up vs Sepsis_D0_survivor |
| EXPH5 | 2.10E-05 | 1.68 | Sepsis_D0_non-survivor up vs Sepsis_D0_survivor |
| CASP14 | 3.21E-04 | 1.68 | Sepsis_D0_non-survivor up vs Sepsis_D0_survivor |
| CLDN9 | 1.76E-03 | 1.68 | Sepsis_D0_non-survivor up vs Sepsis_D0_survivor |
| PRM1 | 1.74E-03 | 1.68 | Sepsis_D0_non-survivor up vs Sepsis_D0_survivor |
| BMP8A | 5.99E-03 | 1.68 | Sepsis_D0_non-survivor up vs Sepsis_D0_survivor |
| OR6A2 | 4.68E-03 | 1.68 | Sepsis_D0_non-survivor up vs Sepsis_D0_survivor |
| FBXL8 | 1.46E-03 | 1.68 | Sepsis_D0_non-survivor up vs Sepsis_D0_survivor |
| HSD17B1 | 8.36E-04 | 1.68 | Sepsis_D0_non-survivor up vs Sepsis_D0_survivor |
| KRT19 | 3.97E-03 | 1.69 | Sepsis_D0_non-survivor up vs Sepsis_D0_survivor |
| GAGE3 | 7.30E-03 | 1.69 | Sepsis_D0_non-survivor up vs Sepsis_D0_survivor |
| TNFRSF11B | 2.06E-03 | 1.69 | Sepsis_D0_non-survivor up vs Sepsis_D0_survivor |
| KCNG2 | 4.87E-03 | 1.69 | Sepsis_D0_non-survivor up vs Sepsis_D0_survivor |
| PYCR1 | 7.90E-04 | 1.69 | Sepsis_D0_non-survivor up vs Sepsis_D0_survivor |
| PRSS22 | 2.38E-03 | 1.69 | Sepsis_D0_non-survivor up vs Sepsis_D0_survivor |
| CYP11B1 | 5.52E-05 | 1.69 | Sepsis_D0_non-survivor up vs Sepsis_D0_survivor |
| COL6A3 | 1.27E-04 | 1.69 | Sepsis_D0_non-survivor up vs Sepsis_D0_survivor |
| TMEM58 | 3.15E-05 | 1.69 | Sepsis_D0_non-survivor up vs Sepsis_D0_survivor |
| CGB2 | 2.53E-03 | 1.69 | Sepsis_D0_non-survivor up vs Sepsis_D0_survivor |
| IL1RL2 | 5.30E-05 | 1.70 | Sepsis_D0_non-survivor up vs Sepsis_D0_survivor |
| LRRC46 | 4.46E-05 | 1.70 | Sepsis_D0_non-survivor up vs Sepsis_D0_survivor |
| NTF3 | 1.23E-03 | 1.70 | Sepsis_D0_non-survivor up vs Sepsis_D0_survivor |
| CDH24 | 6.13E-03 | 1.70 | Sepsis_D0_non-survivor up vs Sepsis_D0_survivor |
| FAM5B | 4.57E-03 | 1.70 | Sepsis_D0_non-survivor up vs Sepsis_D0_survivor |
| MMP28 | 1.49E-03 | 1.70 | Sepsis_D0_non-survivor up vs Sepsis_D0_survivor |
| RPLP1 | 2.06E-03 | 1.70 | Sepsis_D0_non-survivor up vs Sepsis_D0_survivor |
| EEFSEC | 1.35E-03 | 1.70 | Sepsis_D0_non-survivor up vs Sepsis_D0_survivor |
| RAB17 | 1.60E-03 | 1.70 | Sepsis_D0_non-survivor up vs Sepsis_D0_survivor |
| SOX17 | 8.06E-04 | 1.70 | Sepsis_D0_non-survivor up vs Sepsis_D0_survivor |
| FGD1 | 4.62E-06 | 1.71 | Sepsis_D0_non-survivor up vs Sepsis_D0_survivor |
| GNG13 | 3.88E-03 | 1.71 | Sepsis_D0_non-survivor up vs Sepsis_D0_survivor |
| ABCA2 | 1.49E-03 | 1.71 | Sepsis_D0_non-survivor up vs Sepsis_D0_survivor |
| GCNT4 | 3.75E-04 | 1.71 | Sepsis_D0_non-survivor up vs Sepsis_D0_survivor |
| APLP1 | 2.91E-03 | 1.71 | Sepsis_D0_non-survivor up vs Sepsis_D0_survivor |
| PFKFB3 | 1.68E-03 | 1.71 | Sepsis_D0_non-survivor up vs Sepsis_D0_survivor |
| TRIP10 | 6.36E-04 | 1.71 | Sepsis_D0_non-survivor up vs Sepsis_D0_survivor |
| BAI1 | 9.59E-05 | 1.71 | Sepsis_D0_non-survivor up vs Sepsis_D0_survivor |
| PRR5 | 1.00E-04 | 1.71 | Sepsis_D0_non-survivor up vs Sepsis_D0_survivor |
| SIM1 | 1.16E-03 | 1.71 | Sepsis_D0_non-survivor up vs Sepsis_D0_survivor |
| ATAD3C | 3.32E-04 | 1.71 | Sepsis_D0_non-survivor up vs Sepsis_D0_survivor |
| RTBDN | 8.40E-04 | 1.71 | Sepsis_D0_non-survivor up vs Sepsis_D0_survivor |
| CDC42EP1 | 5.47E-04 | 1.71 | Sepsis_D0_non-survivor up vs Sepsis_D0_survivor |
| ARMC5 | 2.76E-03 | 1.71 | Sepsis_D0_non-survivor up vs Sepsis_D0_survivor |
| KCNV2 | 4.40E-04 | 1.71 | Sepsis_D0_non-survivor up vs Sepsis_D0_survivor |
| HCRT | 1.24E-03 | 1.71 | Sepsis_D0_non-survivor up vs Sepsis_D0_survivor |
| COL20A1 | 2.37E-04 | 1.71 | Sepsis_D0_non-survivor up vs Sepsis_D0_survivor |
| HES4 | 3.89E-04 | 1.71 | Sepsis_D0_non-survivor up vs Sepsis_D0_survivor |
| LRP3 | 1.77E-03 | 1.72 | Sepsis_D0_non-survivor up vs Sepsis_D0_survivor |
| CREG2 | 1.82E-05 | 1.72 | Sepsis_D0_non-survivor up vs Sepsis_D0_survivor |
| VPREB1 | 7.42E-04 | 1.72 | Sepsis_D0_non-survivor up vs Sepsis_D0_survivor |
| VSIG2 | 3.30E-06 | 1.72 | Sepsis_D0_non-survivor up vs Sepsis_D0_survivor |
| RASD2 | 6.41E-04 | 1.72 | Sepsis_D0_non-survivor up vs Sepsis_D0_survivor |
| GALR3 | 1.88E-03 | 1.72 | Sepsis_D0_non-survivor up vs Sepsis_D0_survivor |
| NRG2 | 8.48E-06 | 1.72 | Sepsis_D0_non-survivor up vs Sepsis_D0_survivor |
| PANX2 | 4.30E-04 | 1.72 | Sepsis_D0_non-survivor up vs Sepsis_D0_survivor |
| STOX2 | 3.27E-03 | 1.72 | Sepsis_D0_non-survivor up vs Sepsis_D0_survivor |
| OXT | 3.70E-04 | 1.72 | Sepsis_D0_non-survivor up vs Sepsis_D0_survivor |
| CENTG3 | 2.20E-05 | 1.73 | Sepsis_D0_non-survivor up vs Sepsis_D0_survivor |
| P2RX1 | 1.56E-03 | 1.73 | Sepsis_D0_non-survivor up vs Sepsis_D0_survivor |
| PGM5 | 9.60E-04 | 1.73 | Sepsis_D0_non-survivor up vs Sepsis_D0_survivor |
| KRTAP2-4 | 1.30E-04 | 1.73 | Sepsis_D0_non-survivor up vs Sepsis_D0_survivor |
| SLAMF9 | 2.47E-04 | 1.73 | Sepsis_D0_non-survivor up vs Sepsis_D0_survivor |
| PSPN | 9.61E-05 | 1.73 | Sepsis_D0_non-survivor up vs Sepsis_D0_survivor |
| RGNEF | 1.55E-04 | 1.73 | Sepsis_D0_non-survivor up vs Sepsis_D0_survivor |
| ROR1 | 1.68E-03 | 1.73 | Sepsis_D0_non-survivor up vs Sepsis_D0_survivor |
| H1FNT | 5.37E-05 | 1.73 | Sepsis_D0_non-survivor up vs Sepsis_D0_survivor |
| GJB7 | 2.17E-03 | 1.73 | Sepsis_D0_non-survivor up vs Sepsis_D0_survivor |
| DARC | 2.83E-05 | 1.73 | Sepsis_D0_non-survivor up vs Sepsis_D0_survivor |
| BCL2L10 | 4.90E-03 | 1.73 | Sepsis_D0_non-survivor up vs Sepsis_D0_survivor |
| TCF23 | 2.08E-03 | 1.73 | Sepsis_D0_non-survivor up vs Sepsis_D0_survivor |
| NYX | 1.31E-05 | 1.73 | Sepsis_D0_non-survivor up vs Sepsis_D0_survivor |
| LRP11 | 4.30E-03 | 1.73 | Sepsis_D0_non-survivor up vs Sepsis_D0_survivor |
| CCDC114 | 1.48E-04 | 1.73 | Sepsis_D0_non-survivor up vs Sepsis_D0_survivor |
| MASP2 | 9.94E-05 | 1.74 | Sepsis_D0_non-survivor up vs Sepsis_D0_survivor |
| HPX-2 | 1.05E-03 | 1.74 | Sepsis_D0_non-survivor up vs Sepsis_D0_survivor |
| ZDHHC8 | 1.62E-03 | 1.74 | Sepsis_D0_non-survivor up vs Sepsis_D0_survivor |
| DUB3 | 3.99E-04 | 1.74 | Sepsis_D0_non-survivor up vs Sepsis_D0_survivor |
| TTLL5 | 4.31E-04 | 1.74 | Sepsis_D0_non-survivor up vs Sepsis_D0_survivor |
| FOXE3 | 9.80E-04 | 1.74 | Sepsis_D0_non-survivor up vs Sepsis_D0_survivor |
| COX6A2 | 3.02E-04 | 1.74 | Sepsis_D0_non-survivor up vs Sepsis_D0_survivor |
| GNMT | 2.63E-03 | 1.74 | Sepsis_D0_non-survivor up vs Sepsis_D0_survivor |
| PLA2G6 | 2.99E-03 | 1.74 | Sepsis_D0_non-survivor up vs Sepsis_D0_survivor |
| BHLHB4 | 3.66E-04 | 1.74 | Sepsis_D0_non-survivor up vs Sepsis_D0_survivor |
| LRSAM1 | 7.81E-04 | 1.74 | Sepsis_D0_non-survivor up vs Sepsis_D0_survivor |
| KHDRBS3 | 1.68E-03 | 1.75 | Sepsis_D0_non-survivor up vs Sepsis_D0_survivor |
| FIBCD1 | 4.24E-04 | 1.75 | Sepsis_D0_non-survivor up vs Sepsis_D0_survivor |
| LCE2C | 3.34E-03 | 1.75 | Sepsis_D0_non-survivor up vs Sepsis_D0_survivor |
| CHST4 | 2.17E-03 | 1.75 | Sepsis_D0_non-survivor up vs Sepsis_D0_survivor |
| NTN2L | 5.40E-04 | 1.75 | Sepsis_D0_non-survivor up vs Sepsis_D0_survivor |
| PLA2G2F | 3.36E-03 | 1.75 | Sepsis_D0_non-survivor up vs Sepsis_D0_survivor |
| MRGPRF | 1.23E-04 | 1.75 | Sepsis_D0_non-survivor up vs Sepsis_D0_survivor |
| HLA-B | 1.47E-03 | 1.75 | Sepsis_D0_non-survivor up vs Sepsis_D0_survivor |
| CFHR4 | 1.50E-03 | 1.75 | Sepsis_D0_non-survivor up vs Sepsis_D0_survivor |
| EFNA2 | 4.68E-04 | 1.75 | Sepsis_D0_non-survivor up vs Sepsis_D0_survivor |
| SPINK4 | 1.42E-03 | 1.76 | Sepsis_D0_non-survivor up vs Sepsis_D0_survivor |
| SDK2 | 1.07E-04 | 1.76 | Sepsis_D0_non-survivor up vs Sepsis_D0_survivor |
| HEPN1 | 5.17E-03 | 1.76 | Sepsis_D0_non-survivor up vs Sepsis_D0_survivor |
| GRIN1 | 4.52E-04 | 1.76 | Sepsis_D0_non-survivor up vs Sepsis_D0_survivor |
| TMEM95 | 1.75E-03 | 1.76 | Sepsis_D0_non-survivor up vs Sepsis_D0_survivor |
| MYPN | 2.05E-04 | 1.76 | Sepsis_D0_non-survivor up vs Sepsis_D0_survivor |
| GAL3ST2 | 7.17E-04 | 1.76 | Sepsis_D0_non-survivor up vs Sepsis_D0_survivor |
| LPHN1 | 2.95E-04 | 1.76 | Sepsis_D0_non-survivor up vs Sepsis_D0_survivor |
| GFRA4 | 4.81E-04 | 1.76 | Sepsis_D0_non-survivor up vs Sepsis_D0_survivor |
| FAM47C | 2.22E-03 | 1.76 | Sepsis_D0_non-survivor up vs Sepsis_D0_survivor |
| AMAC1L2 | 4.19E-03 | 1.76 | Sepsis_D0_non-survivor up vs Sepsis_D0_survivor |
| PCDHB13 | 2.49E-04 | 1.76 | Sepsis_D0_non-survivor up vs Sepsis_D0_survivor |
| CHMP4C | 6.03E-04 | 1.76 | Sepsis_D0_non-survivor up vs Sepsis_D0_survivor |
| UGT2B11 | 4.62E-04 | 1.76 | Sepsis_D0_non-survivor up vs Sepsis_D0_survivor |
| PROP1 | 8.75E-04 | 1.77 | Sepsis_D0_non-survivor up vs Sepsis_D0_survivor |
| L12234 | 4.61E-04 | 1.77 | Sepsis_D0_non-survivor up vs Sepsis_D0_survivor |
| TTTY13 | 1.60E-03 | 1.77 | Sepsis_D0_non-survivor up vs Sepsis_D0_survivor |
| GYPE | 2.47E-03 | 1.77 | Sepsis_D0_non-survivor up vs Sepsis_D0_survivor |
| CHRM3 | 3.06E-03 | 1.77 | Sepsis_D0_non-survivor up vs Sepsis_D0_survivor |
| GPR153 | 7.49E-04 | 1.77 | Sepsis_D0_non-survivor up vs Sepsis_D0_survivor |
| TRIM46 | 8.29E-04 | 1.77 | Sepsis_D0_non-survivor up vs Sepsis_D0_survivor |
| CCDC113 | 1.33E-04 | 1.77 | Sepsis_D0_non-survivor up vs Sepsis_D0_survivor |
| S100A8 | 6.72E-03 | 1.77 | Sepsis_D0_non-survivor up vs Sepsis_D0_survivor |
| KLK14 | 1.48E-03 | 1.77 | Sepsis_D0_non-survivor up vs Sepsis_D0_survivor |
| LTB4R2 | 2.27E-03 | 1.77 | Sepsis_D0_non-survivor up vs Sepsis_D0_survivor |
| PI16 | 7.44E-03 | 1.77 | Sepsis_D0_non-survivor up vs Sepsis_D0_survivor |
| WNK2 | 6.24E-03 | 1.77 | Sepsis_D0_non-survivor up vs Sepsis_D0_survivor |
| FBXL19 | 3.56E-05 | 1.78 | Sepsis_D0_non-survivor up vs Sepsis_D0_survivor |
| SPINK7 | 1.40E-03 | 1.78 | Sepsis_D0_non-survivor up vs Sepsis_D0_survivor |
| OR4D2 | 1.29E-03 | 1.78 | Sepsis_D0_non-survivor up vs Sepsis_D0_survivor |
| ADAMTS14 | 5.39E-03 | 1.78 | Sepsis_D0_non-survivor up vs Sepsis_D0_survivor |
| UNQ473 | 2.16E-05 | 1.78 | Sepsis_D0_non-survivor up vs Sepsis_D0_survivor |
| IL1RAPL1 | 2.28E-05 | 1.78 | Sepsis_D0_non-survivor up vs Sepsis_D0_survivor |
| TTBK1 | 2.58E-05 | 1.78 | Sepsis_D0_non-survivor up vs Sepsis_D0_survivor |
| FGF18 | 1.47E-03 | 1.78 | Sepsis_D0_non-survivor up vs Sepsis_D0_survivor |
| STIM2 | 6.63E-03 | 1.78 | Sepsis_D0_non-survivor up vs Sepsis_D0_survivor |
| TRPV1 | 1.14E-04 | 1.78 | Sepsis_D0_non-survivor up vs Sepsis_D0_survivor |
| BAI3 | 4.85E-03 | 1.78 | Sepsis_D0_non-survivor up vs Sepsis_D0_survivor |
| SLC16A4 | 1.07E-03 | 1.78 | Sepsis_D0_non-survivor up vs Sepsis_D0_survivor |
| ZNF575 | 2.81E-03 | 1.78 | Sepsis_D0_non-survivor up vs Sepsis_D0_survivor |
| RTN4RL2 | 2.71E-05 | 1.78 | Sepsis_D0_non-survivor up vs Sepsis_D0_survivor |
| LRRN5 | 1.17E-04 | 1.78 | Sepsis_D0_non-survivor up vs Sepsis_D0_survivor |
| ENPP7 | 2.47E-04 | 1.78 | Sepsis_D0_non-survivor up vs Sepsis_D0_survivor |
| CACNG4 | 2.32E-05 | 1.78 | Sepsis_D0_non-survivor up vs Sepsis_D0_survivor |
| TNXB | 1.13E-03 | 1.79 | Sepsis_D0_non-survivor up vs Sepsis_D0_survivor |
| KCNAB3 | 3.90E-03 | 1.79 | Sepsis_D0_non-survivor up vs Sepsis_D0_survivor |
| OXCT2 | 2.37E-03 | 1.79 | Sepsis_D0_non-survivor up vs Sepsis_D0_survivor |
| OR5F1 | 5.74E-06 | 1.79 | Sepsis_D0_non-survivor up vs Sepsis_D0_survivor |
| MUC6 | 3.14E-04 | 1.79 | Sepsis_D0_non-survivor up vs Sepsis_D0_survivor |
| ACPT | 3.76E-04 | 1.79 | Sepsis_D0_non-survivor up vs Sepsis_D0_survivor |
| GEMIN5 | 1.22E-03 | 1.79 | Sepsis_D0_non-survivor up vs Sepsis_D0_survivor |
| CYB561D1 | 1.13E-03 | 1.79 | Sepsis_D0_non-survivor up vs Sepsis_D0_survivor |
| ANKRD53 | 9.07E-04 | 1.79 | Sepsis_D0_non-survivor up vs Sepsis_D0_survivor |
| TMUB1 | 2.55E-04 | 1.79 | Sepsis_D0_non-survivor up vs Sepsis_D0_survivor |
| MYBPC2 | 1.85E-03 | 1.79 | Sepsis_D0_non-survivor up vs Sepsis_D0_survivor |
| PCDHGA2 | 2.44E-04 | 1.79 | Sepsis_D0_non-survivor up vs Sepsis_D0_survivor |
| NKX3-1 | 5.11E-04 | 1.79 | Sepsis_D0_non-survivor up vs Sepsis_D0_survivor |
| SLC7A4 | 2.30E-04 | 1.79 | Sepsis_D0_non-survivor up vs Sepsis_D0_survivor |
| LRP5 | 4.24E-04 | 1.80 | Sepsis_D0_non-survivor up vs Sepsis_D0_survivor |
| FRMD4A | 7.98E-04 | 1.80 | Sepsis_D0_non-survivor up vs Sepsis_D0_survivor |
| MYH14 | 1.57E-03 | 1.80 | Sepsis_D0_non-survivor up vs Sepsis_D0_survivor |
| SLC6A19 | 1.31E-03 | 1.80 | Sepsis_D0_non-survivor up vs Sepsis_D0_survivor |
| GABRA3 | 1.71E-03 | 1.80 | Sepsis_D0_non-survivor up vs Sepsis_D0_survivor |
| TRPM1 | 1.67E-03 | 1.80 | Sepsis_D0_non-survivor up vs Sepsis_D0_survivor |
| KCNK7 | 2.85E-03 | 1.80 | Sepsis_D0_non-survivor up vs Sepsis_D0_survivor |
| FKHL18 | 2.28E-04 | 1.80 | Sepsis_D0_non-survivor up vs Sepsis_D0_survivor |
| SDS | 1.20E-04 | 1.80 | Sepsis_D0_non-survivor up vs Sepsis_D0_survivor |
| TERT | 2.17E-03 | 1.80 | Sepsis_D0_non-survivor up vs Sepsis_D0_survivor |
| GCGR | 1.27E-03 | 1.80 | Sepsis_D0_non-survivor up vs Sepsis_D0_survivor |
| KRT8 | 3.66E-04 | 1.80 | Sepsis_D0_non-survivor up vs Sepsis_D0_survivor |
| CDH22 | 4.53E-04 | 1.80 | Sepsis_D0_non-survivor up vs Sepsis_D0_survivor |
| SAA4 | 9.10E-04 | 1.81 | Sepsis_D0_non-survivor up vs Sepsis_D0_survivor |
| CCL15 | 4.60E-03 | 1.81 | Sepsis_D0_non-survivor up vs Sepsis_D0_survivor |
| THY1 | 1.25E-03 | 1.81 | Sepsis_D0_non-survivor up vs Sepsis_D0_survivor |
| PKN3 | 1.41E-04 | 1.81 | Sepsis_D0_non-survivor up vs Sepsis_D0_survivor |
| RPL19 | 8.57E-04 | 1.81 | Sepsis_D0_non-survivor up vs Sepsis_D0_survivor |
| APOL5 | 6.28E-04 | 1.81 | Sepsis_D0_non-survivor up vs Sepsis_D0_survivor |
| DRD5 | 4.00E-03 | 1.81 | Sepsis_D0_non-survivor up vs Sepsis_D0_survivor |
| SMCR5 | 1.04E-04 | 1.81 | Sepsis_D0_non-survivor up vs Sepsis_D0_survivor |
| TTTY5 | 3.63E-03 | 1.81 | Sepsis_D0_non-survivor up vs Sepsis_D0_survivor |
| CPNE7 | 1.49E-03 | 1.81 | Sepsis_D0_non-survivor up vs Sepsis_D0_survivor |
| ENPP1 | 8.57E-06 | 1.81 | Sepsis_D0_non-survivor up vs Sepsis_D0_survivor |
| TSNARE1 | 1.29E-04 | 1.81 | Sepsis_D0_non-survivor up vs Sepsis_D0_survivor |
| KRT15 | 8.67E-04 | 1.81 | Sepsis_D0_non-survivor up vs Sepsis_D0_survivor |
| HSPB9 | 1.57E-03 | 1.82 | Sepsis_D0_non-survivor up vs Sepsis_D0_survivor |
| MICAL3 | 2.48E-03 | 1.82 | Sepsis_D0_non-survivor up vs Sepsis_D0_survivor |
| FAM70B | 1.07E-04 | 1.82 | Sepsis_D0_non-survivor up vs Sepsis_D0_survivor |
| EIF1 | 9.10E-05 | 1.82 | Sepsis_D0_non-survivor up vs Sepsis_D0_survivor |
| KAAG1 | 1.20E-03 | 1.82 | Sepsis_D0_non-survivor up vs Sepsis_D0_survivor |
| FAM46B | 6.98E-03 | 1.82 | Sepsis_D0_non-survivor up vs Sepsis_D0_survivor |
| CDK5R2 | 7.61E-05 | 1.82 | Sepsis_D0_non-survivor up vs Sepsis_D0_survivor |
| CREB3L3 | 1.32E-04 | 1.82 | Sepsis_D0_non-survivor up vs Sepsis_D0_survivor |
| SPSB4 | 1.61E-03 | 1.82 | Sepsis_D0_non-survivor up vs Sepsis_D0_survivor |
| ADCYAP1R1 | 8.30E-04 | 1.82 | Sepsis_D0_non-survivor up vs Sepsis_D0_survivor |
| CNNM1 | 1.52E-03 | 1.83 | Sepsis_D0_non-survivor up vs Sepsis_D0_survivor |
| SPACA3 | 1.12E-03 | 1.83 | Sepsis_D0_non-survivor up vs Sepsis_D0_survivor |
| COTL1 | 4.67E-03 | 1.83 | Sepsis_D0_non-survivor up vs Sepsis_D0_survivor |
| CCDC120 | 1.52E-04 | 1.83 | Sepsis_D0_non-survivor up vs Sepsis_D0_survivor |
| FLT4 | 9.03E-05 | 1.83 | Sepsis_D0_non-survivor up vs Sepsis_D0_survivor |
| RASGEF1C | 2.08E-04 | 1.83 | Sepsis_D0_non-survivor up vs Sepsis_D0_survivor |
| POLR2A | 2.03E-04 | 1.83 | Sepsis_D0_non-survivor up vs Sepsis_D0_survivor |
| CNKSR1 | 5.51E-04 | 1.83 | Sepsis_D0_non-survivor up vs Sepsis_D0_survivor |
| BCAM | 5.72E-04 | 1.83 | Sepsis_D0_non-survivor up vs Sepsis_D0_survivor |
| UGT2B10 | 1.09E-03 | 1.83 | Sepsis_D0_non-survivor up vs Sepsis_D0_survivor |
| PRX | 1.24E-03 | 1.84 | Sepsis_D0_non-survivor up vs Sepsis_D0_survivor |
| MMP17 | 4.26E-04 | 1.84 | Sepsis_D0_non-survivor up vs Sepsis_D0_survivor |
| LIMS2 | 1.02E-03 | 1.85 | Sepsis_D0_non-survivor up vs Sepsis_D0_survivor |
| CACNA1B | 5.33E-04 | 1.85 | Sepsis_D0_non-survivor up vs Sepsis_D0_survivor |
| ABLIM2 | 2.94E-03 | 1.85 | Sepsis_D0_non-survivor up vs Sepsis_D0_survivor |
| CCBP2 | 1.84E-03 | 1.85 | Sepsis_D0_non-survivor up vs Sepsis_D0_survivor |
| UTF1 | 1.07E-03 | 1.85 | Sepsis_D0_non-survivor up vs Sepsis_D0_survivor |
| COL4A3 | 6.07E-03 | 1.85 | Sepsis_D0_non-survivor up vs Sepsis_D0_survivor |
| LY6D | 6.68E-04 | 1.85 | Sepsis_D0_non-survivor up vs Sepsis_D0_survivor |
| FAM83C | 5.93E-03 | 1.85 | Sepsis_D0_non-survivor up vs Sepsis_D0_survivor |
| TM4SF5 | 7.13E-03 | 1.85 | Sepsis_D0_non-survivor up vs Sepsis_D0_survivor |
| PTMS | 9.90E-05 | 1.86 | Sepsis_D0_non-survivor up vs Sepsis_D0_survivor |
| PROCA1 | 7.38E-05 | 1.86 | Sepsis_D0_non-survivor up vs Sepsis_D0_survivor |
| PDE4C | 7.15E-03 | 1.86 | Sepsis_D0_non-survivor up vs Sepsis_D0_survivor |
| HR | 3.53E-04 | 1.86 | Sepsis_D0_non-survivor up vs Sepsis_D0_survivor |
| TNNI1 | 2.80E-04 | 1.87 | Sepsis_D0_non-survivor up vs Sepsis_D0_survivor |
| NLF2 | 2.17E-03 | 1.87 | Sepsis_D0_non-survivor up vs Sepsis_D0_survivor |
| PPL | 1.56E-04 | 1.87 | Sepsis_D0_non-survivor up vs Sepsis_D0_survivor |
| SOX18 | 2.52E-04 | 1.87 | Sepsis_D0_non-survivor up vs Sepsis_D0_survivor |
| NPPC | 3.52E-03 | 1.87 | Sepsis_D0_non-survivor up vs Sepsis_D0_survivor |
| PCSK1N | 2.87E-04 | 1.87 | Sepsis_D0_non-survivor up vs Sepsis_D0_survivor |
| ZKSCAN1 | 4.90E-03 | 1.87 | Sepsis_D0_non-survivor up vs Sepsis_D0_survivor |
| SMA4 | 5.01E-03 | 1.87 | Sepsis_D0_non-survivor up vs Sepsis_D0_survivor |
| TWIST1 | 1.52E-04 | 1.87 | Sepsis_D0_non-survivor up vs Sepsis_D0_survivor |
| KRT81 | 3.05E-04 | 1.87 | Sepsis_D0_non-survivor up vs Sepsis_D0_survivor |
| CAMK2A | 2.03E-03 | 1.88 | Sepsis_D0_non-survivor up vs Sepsis_D0_survivor |
| LIME1 | 8.50E-05 | 1.88 | Sepsis_D0_non-survivor up vs Sepsis_D0_survivor |
| NFKBIA | 4.58E-04 | 1.88 | Sepsis_D0_non-survivor up vs Sepsis_D0_survivor |
| SLC44A5 | 5.41E-03 | 1.88 | Sepsis_D0_non-survivor up vs Sepsis_D0_survivor |
| SERPINB3 | 1.48E-03 | 1.88 | Sepsis_D0_non-survivor up vs Sepsis_D0_survivor |
| RAXL1 | 7.92E-04 | 1.88 | Sepsis_D0_non-survivor up vs Sepsis_D0_survivor |
| ALPL | 1.50E-03 | 1.88 | Sepsis_D0_non-survivor up vs Sepsis_D0_survivor |
| LONRF2 | 9.60E-04 | 1.88 | Sepsis_D0_non-survivor up vs Sepsis_D0_survivor |
| SH3RF2 | 3.10E-03 | 1.88 | Sepsis_D0_non-survivor up vs Sepsis_D0_survivor |
| GPR157 | 3.15E-03 | 1.89 | Sepsis_D0_non-survivor up vs Sepsis_D0_survivor |
| TCEB3C | 6.47E-03 | 1.89 | Sepsis_D0_non-survivor up vs Sepsis_D0_survivor |
| BHMT2 | 9.29E-04 | 1.89 | Sepsis_D0_non-survivor up vs Sepsis_D0_survivor |
| A2BP1 | 6.60E-04 | 1.89 | Sepsis_D0_non-survivor up vs Sepsis_D0_survivor |
| CCDC36 | 3.20E-03 | 1.89 | Sepsis_D0_non-survivor up vs Sepsis_D0_survivor |
| CRYBA4 | 2.15E-03 | 1.89 | Sepsis_D0_non-survivor up vs Sepsis_D0_survivor |
| SNF1LK | 5.05E-04 | 1.90 | Sepsis_D0_non-survivor up vs Sepsis_D0_survivor |
| ADAMTS13 | 1.07E-03 | 1.90 | Sepsis_D0_non-survivor up vs Sepsis_D0_survivor |
| MUCDHL | 2.50E-03 | 1.90 | Sepsis_D0_non-survivor up vs Sepsis_D0_survivor |
| CYP3A4 | 2.33E-03 | 1.90 | Sepsis_D0_non-survivor up vs Sepsis_D0_survivor |
| IRGC | 3.71E-03 | 1.90 | Sepsis_D0_non-survivor up vs Sepsis_D0_survivor |
| TMEM132A | 1.35E-03 | 1.90 | Sepsis_D0_non-survivor up vs Sepsis_D0_survivor |
| ORC6L | 2.82E-03 | 1.90 | Sepsis_D0_non-survivor up vs Sepsis_D0_survivor |
| OCM | 4.57E-05 | 1.90 | Sepsis_D0_non-survivor up vs Sepsis_D0_survivor |
| ARPM1 | 3.30E-03 | 1.90 | Sepsis_D0_non-survivor up vs Sepsis_D0_survivor |
| CTF1 | 3.59E-03 | 1.90 | Sepsis_D0_non-survivor up vs Sepsis_D0_survivor |
| UCN3 | 2.61E-04 | 1.90 | Sepsis_D0_non-survivor up vs Sepsis_D0_survivor |
| BGN | 2.07E-03 | 1.90 | Sepsis_D0_non-survivor up vs Sepsis_D0_survivor |
| CHRD | 1.52E-04 | 1.91 | Sepsis_D0_non-survivor up vs Sepsis_D0_survivor |
| GPR172B | 3.25E-03 | 1.91 | Sepsis_D0_non-survivor up vs Sepsis_D0_survivor |
| IGSF22 | 6.70E-03 | 1.91 | Sepsis_D0_non-survivor up vs Sepsis_D0_survivor |
| GPR17 | 1.23E-03 | 1.91 | Sepsis_D0_non-survivor up vs Sepsis_D0_survivor |
| ZBTB20 | 4.88E-04 | 1.91 | Sepsis_D0_non-survivor up vs Sepsis_D0_survivor |
| PDE7B | 4.26E-03 | 1.91 | Sepsis_D0_non-survivor up vs Sepsis_D0_survivor |
| SULT2B1 | 2.59E-03 | 1.91 | Sepsis_D0_non-survivor up vs Sepsis_D0_survivor |
| XCR1 | 3.60E-03 | 1.91 | Sepsis_D0_non-survivor up vs Sepsis_D0_survivor |
| MLL3 | 6.32E-04 | 1.91 | Sepsis_D0_non-survivor up vs Sepsis_D0_survivor |
| PLAC4 | 3.14E-05 | 1.91 | Sepsis_D0_non-survivor up vs Sepsis_D0_survivor |
| FOXC2 | 5.28E-03 | 1.92 | Sepsis_D0_non-survivor up vs Sepsis_D0_survivor |
| DUSP26 | 4.02E-03 | 1.92 | Sepsis_D0_non-survivor up vs Sepsis_D0_survivor |
| SSTR4 | 3.22E-03 | 1.92 | Sepsis_D0_non-survivor up vs Sepsis_D0_survivor |
| WNT5B | 4.40E-03 | 1.92 | Sepsis_D0_non-survivor up vs Sepsis_D0_survivor |
| CTRB2 | 2.81E-03 | 1.92 | Sepsis_D0_non-survivor up vs Sepsis_D0_survivor |
| CCL4 | 6.21E-03 | 1.92 | Sepsis_D0_non-survivor up vs Sepsis_D0_survivor |
| BBS5 | 2.21E-03 | 1.93 | Sepsis_D0_non-survivor up vs Sepsis_D0_survivor |
| BTNL2 | 5.18E-04 | 1.93 | Sepsis_D0_non-survivor up vs Sepsis_D0_survivor |
| DEPDC2 | 2.58E-03 | 1.93 | Sepsis_D0_non-survivor up vs Sepsis_D0_survivor |
| MMEL1 | 1.49E-03 | 1.93 | Sepsis_D0_non-survivor up vs Sepsis_D0_survivor |
| UNQ6411 | 3.52E-05 | 1.93 | Sepsis_D0_non-survivor up vs Sepsis_D0_survivor |
| PIP5K1C | 1.25E-03 | 1.93 | Sepsis_D0_non-survivor up vs Sepsis_D0_survivor |
| MESP1 | 7.67E-04 | 1.93 | Sepsis_D0_non-survivor up vs Sepsis_D0_survivor |
| TMEM125 | 9.95E-04 | 1.93 | Sepsis_D0_non-survivor up vs Sepsis_D0_survivor |
| ACR | 9.56E-06 | 1.93 | Sepsis_D0_non-survivor up vs Sepsis_D0_survivor |
| SLC16A8 | 2.78E-03 | 1.94 | Sepsis_D0_non-survivor up vs Sepsis_D0_survivor |
| MUC2 | 1.56E-04 | 1.94 | Sepsis_D0_non-survivor up vs Sepsis_D0_survivor |
| ADAMTS8 | 1.61E-04 | 1.94 | Sepsis_D0_non-survivor up vs Sepsis_D0_survivor |
| CYP1A2 | 2.75E-03 | 1.94 | Sepsis_D0_non-survivor up vs Sepsis_D0_survivor |
| NUP62CL | 3.87E-03 | 1.95 | Sepsis_D0_non-survivor up vs Sepsis_D0_survivor |
| NKX6-2 | 3.56E-03 | 1.95 | Sepsis_D0_non-survivor up vs Sepsis_D0_survivor |
| SOX8 | 2.67E-04 | 1.95 | Sepsis_D0_non-survivor up vs Sepsis_D0_survivor |
| NGFB | 7.83E-04 | 1.95 | Sepsis_D0_non-survivor up vs Sepsis_D0_survivor |
| TH | 5.53E-05 | 1.96 | Sepsis_D0_non-survivor up vs Sepsis_D0_survivor |
| CDCA7L | 9.97E-04 | 1.96 | Sepsis_D0_non-survivor up vs Sepsis_D0_survivor |
| LECT1 | 1.53E-03 | 1.96 | Sepsis_D0_non-survivor up vs Sepsis_D0_survivor |
| ITGB4 | 8.39E-04 | 1.96 | Sepsis_D0_non-survivor up vs Sepsis_D0_survivor |
| CHRNA6 | 1.92E-06 | 1.96 | Sepsis_D0_non-survivor up vs Sepsis_D0_survivor |
| KRT3 | 2.51E-04 | 1.97 | Sepsis_D0_non-survivor up vs Sepsis_D0_survivor |
| NPBWR1 | 3.83E-04 | 1.97 | Sepsis_D0_non-survivor up vs Sepsis_D0_survivor |
| GPR149 | 3.76E-03 | 1.97 | Sepsis_D0_non-survivor up vs Sepsis_D0_survivor |
| PRO2900 | 5.75E-03 | 1.97 | Sepsis_D0_non-survivor up vs Sepsis_D0_survivor |
| KLHL10 | 7.57E-03 | 1.97 | Sepsis_D0_non-survivor up vs Sepsis_D0_survivor |
| ZNF206 | 9.22E-04 | 1.97 | Sepsis_D0_non-survivor up vs Sepsis_D0_survivor |
| CSAG1 | 6.61E-04 | 1.97 | Sepsis_D0_non-survivor up vs Sepsis_D0_survivor |
| CHRNA7 | 3.79E-05 | 1.97 | Sepsis_D0_non-survivor up vs Sepsis_D0_survivor |
| NHLH2 | 2.33E-05 | 1.97 | Sepsis_D0_non-survivor up vs Sepsis_D0_survivor |
| AD7C-NTP | 5.41E-04 | 1.97 | Sepsis_D0_non-survivor up vs Sepsis_D0_survivor |
| TMEM153 | 6.82E-04 | 1.97 | Sepsis_D0_non-survivor up vs Sepsis_D0_survivor |
| SRPX | 8.24E-04 | 1.98 | Sepsis_D0_non-survivor up vs Sepsis_D0_survivor |
| GYLTL1B | 2.87E-05 | 1.98 | Sepsis_D0_non-survivor up vs Sepsis_D0_survivor |
| NTRK2 | 5.73E-03 | 1.98 | Sepsis_D0_non-survivor up vs Sepsis_D0_survivor |
| KCNK4 | 2.50E-03 | 1.98 | Sepsis_D0_non-survivor up vs Sepsis_D0_survivor |
| S80864 | 2.17E-03 | 1.98 | Sepsis_D0_non-survivor up vs Sepsis_D0_survivor |
| FBXO17 | 2.63E-04 | 1.98 | Sepsis_D0_non-survivor up vs Sepsis_D0_survivor |
| EYA4 | 1.92E-03 | 1.98 | Sepsis_D0_non-survivor up vs Sepsis_D0_survivor |
| PTCHD1 | 1.35E-03 | 1.99 | Sepsis_D0_non-survivor up vs Sepsis_D0_survivor |
| TMEM142A | 6.13E-03 | 2.00 | Sepsis_D0_non-survivor up vs Sepsis_D0_survivor |
| GALNTL2 | 6.81E-04 | 2.00 | Sepsis_D0_non-survivor up vs Sepsis_D0_survivor |
| KIF26A | 2.34E-03 | 2.00 | Sepsis_D0_non-survivor up vs Sepsis_D0_survivor |
| ALPK1 | 1.41E-03 | 2.00 | Sepsis_D0_non-survivor up vs Sepsis_D0_survivor |
| GJB4 | 1.23E-03 | 2.01 | Sepsis_D0_non-survivor up vs Sepsis_D0_survivor |
| TNFAIP8L3 | 3.55E-04 | 2.01 | Sepsis_D0_non-survivor up vs Sepsis_D0_survivor |
| LCE3E | 4.89E-04 | 2.02 | Sepsis_D0_non-survivor up vs Sepsis_D0_survivor |
| PLXNB3 | 1.30E-06 | 2.02 | Sepsis_D0_non-survivor up vs Sepsis_D0_survivor |
| VCX | 4.84E-03 | 2.02 | Sepsis_D0_non-survivor up vs Sepsis_D0_survivor |
| TMEM16A | 8.58E-04 | 2.02 | Sepsis_D0_non-survivor up vs Sepsis_D0_survivor |
| POLR1C | 1.15E-04 | 2.03 | Sepsis_D0_non-survivor up vs Sepsis_D0_survivor |
| RPIB9 | 1.30E-03 | 2.03 | Sepsis_D0_non-survivor up vs Sepsis_D0_survivor |
| TMEFF2 | 1.71E-03 | 2.03 | Sepsis_D0_non-survivor up vs Sepsis_D0_survivor |
| ABP1 | 7.25E-04 | 2.03 | Sepsis_D0_non-survivor up vs Sepsis_D0_survivor |
| RHCG | 2.36E-04 | 2.03 | Sepsis_D0_non-survivor up vs Sepsis_D0_survivor |
| HTR1A | 3.33E-03 | 2.03 | Sepsis_D0_non-survivor up vs Sepsis_D0_survivor |
| IGFBP6 | 9.81E-04 | 2.03 | Sepsis_D0_non-survivor up vs Sepsis_D0_survivor |
| WT1 | 6.03E-03 | 2.04 | Sepsis_D0_non-survivor up vs Sepsis_D0_survivor |
| GRIK3 | 6.03E-04 | 2.04 | Sepsis_D0_non-survivor up vs Sepsis_D0_survivor |
| DNAJB5 | 5.73E-03 | 2.04 | Sepsis_D0_non-survivor up vs Sepsis_D0_survivor |
| CCDC108 | 9.86E-04 | 2.04 | Sepsis_D0_non-survivor up vs Sepsis_D0_survivor |
| MPN2 | 6.54E-03 | 2.05 | Sepsis_D0_non-survivor up vs Sepsis_D0_survivor |
| SYNPO | 8.88E-05 | 2.05 | Sepsis_D0_non-survivor up vs Sepsis_D0_survivor |
| TDRD10 | 1.17E-04 | 2.06 | Sepsis_D0_non-survivor up vs Sepsis_D0_survivor |
| HOXD3 | 3.53E-05 | 2.06 | Sepsis_D0_non-survivor up vs Sepsis_D0_survivor |
| GABRB1 | 2.69E-03 | 2.06 | Sepsis_D0_non-survivor up vs Sepsis_D0_survivor |
| RFPL1 | 5.92E-03 | 2.06 | Sepsis_D0_non-survivor up vs Sepsis_D0_survivor |
| DMBX1 | 4.68E-03 | 2.07 | Sepsis_D0_non-survivor up vs Sepsis_D0_survivor |
| FAM92A3 | 1.12E-04 | 2.07 | Sepsis_D0_non-survivor up vs Sepsis_D0_survivor |
| MYOD1 | 1.89E-04 | 2.08 | Sepsis_D0_non-survivor up vs Sepsis_D0_survivor |
| TBC1D21 | 2.27E-04 | 2.08 | Sepsis_D0_non-survivor up vs Sepsis_D0_survivor |
| FAM59A | 3.20E-04 | 2.09 | Sepsis_D0_non-survivor up vs Sepsis_D0_survivor |
| SPTBN2 | 2.29E-04 | 2.10 | Sepsis_D0_non-survivor up vs Sepsis_D0_survivor |
| PARD6G | 3.53E-04 | 2.10 | Sepsis_D0_non-survivor up vs Sepsis_D0_survivor |
| WIT1 | 1.41E-05 | 2.10 | Sepsis_D0_non-survivor up vs Sepsis_D0_survivor |
| MAGED4 | 7.20E-04 | 2.10 | Sepsis_D0_non-survivor up vs Sepsis_D0_survivor |
| VSX1 | 2.05E-05 | 2.10 | Sepsis_D0_non-survivor up vs Sepsis_D0_survivor |
| ADRA2A | 2.07E-03 | 2.10 | Sepsis_D0_non-survivor up vs Sepsis_D0_survivor |
| LENG9 | 2.01E-05 | 2.10 | Sepsis_D0_non-survivor up vs Sepsis_D0_survivor |
| GNAZ | 3.41E-04 | 2.10 | Sepsis_D0_non-survivor up vs Sepsis_D0_survivor |
| PTCHD2 | 5.65E-04 | 2.11 | Sepsis_D0_non-survivor up vs Sepsis_D0_survivor |
| BAIAP2L1 | 7.53E-03 | 2.11 | Sepsis_D0_non-survivor up vs Sepsis_D0_survivor |
| PPYR1 | 4.69E-04 | 2.12 | Sepsis_D0_non-survivor up vs Sepsis_D0_survivor |
| NLF1 | 3.66E-03 | 2.12 | Sepsis_D0_non-survivor up vs Sepsis_D0_survivor |
| UPK3B | 4.54E-04 | 2.12 | Sepsis_D0_non-survivor up vs Sepsis_D0_survivor |
| TNFSF9 | 6.80E-04 | 2.12 | Sepsis_D0_non-survivor up vs Sepsis_D0_survivor |
| TSPAN10 | 1.62E-04 | 2.12 | Sepsis_D0_non-survivor up vs Sepsis_D0_survivor |
| HOXA2 | 2.33E-04 | 2.12 | Sepsis_D0_non-survivor up vs Sepsis_D0_survivor |
| DUX2 | 8.44E-04 | 2.12 | Sepsis_D0_non-survivor up vs Sepsis_D0_survivor |
| KRTAP13-2 | 2.10E-03 | 2.13 | Sepsis_D0_non-survivor up vs Sepsis_D0_survivor |
| SNX26 | 1.91E-03 | 2.13 | Sepsis_D0_non-survivor up vs Sepsis_D0_survivor |
| HOXC9 | 3.70E-04 | 2.14 | Sepsis_D0_non-survivor up vs Sepsis_D0_survivor |
| TMEM16C | 3.42E-06 | 2.14 | Sepsis_D0_non-survivor up vs Sepsis_D0_survivor |
| FOXB1 | 8.10E-05 | 2.14 | Sepsis_D0_non-survivor up vs Sepsis_D0_survivor |
| GPR20 | 6.90E-04 | 2.14 | Sepsis_D0_non-survivor up vs Sepsis_D0_survivor |
| REEP6 | 2.73E-04 | 2.15 | Sepsis_D0_non-survivor up vs Sepsis_D0_survivor |
| ZFHX4 | 3.02E-04 | 2.15 | Sepsis_D0_non-survivor up vs Sepsis_D0_survivor |
| MOG | 1.78E-07 | 2.15 | Sepsis_D0_non-survivor up vs Sepsis_D0_survivor |
| PRB4 | 1.88E-04 | 2.15 | Sepsis_D0_non-survivor up vs Sepsis_D0_survivor |
| PRR11 | 2.30E-03 | 2.15 | Sepsis_D0_non-survivor up vs Sepsis_D0_survivor |
| REXO1L1 | 3.11E-03 | 2.17 | Sepsis_D0_non-survivor up vs Sepsis_D0_survivor |
| CRYGC | 2.85E-04 | 2.17 | Sepsis_D0_non-survivor up vs Sepsis_D0_survivor |
| CSRP2 | 1.49E-03 | 2.17 | Sepsis_D0_non-survivor up vs Sepsis_D0_survivor |
| EPHA2 | 6.79E-05 | 2.17 | Sepsis_D0_non-survivor up vs Sepsis_D0_survivor |
| LGALS7 | 5.63E-04 | 2.17 | Sepsis_D0_non-survivor up vs Sepsis_D0_survivor |
| SH2B2 | 2.02E-05 | 2.18 | Sepsis_D0_non-survivor up vs Sepsis_D0_survivor |
| PGLYRP2 | 1.36E-05 | 2.18 | Sepsis_D0_non-survivor up vs Sepsis_D0_survivor |
| GCM2 | 5.28E-03 | 2.18 | Sepsis_D0_non-survivor up vs Sepsis_D0_survivor |
| IQCF2 | 2.00E-03 | 2.18 | Sepsis_D0_non-survivor up vs Sepsis_D0_survivor |
| PIK3C2A | 3.80E-03 | 2.18 | Sepsis_D0_non-survivor up vs Sepsis_D0_survivor |
| ADRA2C | 1.22E-03 | 2.19 | Sepsis_D0_non-survivor up vs Sepsis_D0_survivor |
| GLIS2 | 8.08E-05 | 2.19 | Sepsis_D0_non-survivor up vs Sepsis_D0_survivor |
| CCL21 | 1.59E-05 | 2.20 | Sepsis_D0_non-survivor up vs Sepsis_D0_survivor |
| LHFPL2 | 5.67E-03 | 2.21 | Sepsis_D0_non-survivor up vs Sepsis_D0_survivor |
| MPPED1 | 1.19E-03 | 2.21 | Sepsis_D0_non-survivor up vs Sepsis_D0_survivor |
| SPRR2B | 3.23E-04 | 2.21 | Sepsis_D0_non-survivor up vs Sepsis_D0_survivor |
| SRCAP | 2.58E-04 | 2.21 | Sepsis_D0_non-survivor up vs Sepsis_D0_survivor |
| ATN1 | 9.94E-05 | 2.21 | Sepsis_D0_non-survivor up vs Sepsis_D0_survivor |
| CLCF1 | 6.33E-03 | 2.22 | Sepsis_D0_non-survivor up vs Sepsis_D0_survivor |
| TCTE3 | 9.38E-05 | 2.23 | Sepsis_D0_non-survivor up vs Sepsis_D0_survivor |
| CHGA | 8.27E-05 | 2.24 | Sepsis_D0_non-survivor up vs Sepsis_D0_survivor |
| GRIN3B | 1.52E-04 | 2.24 | Sepsis_D0_non-survivor up vs Sepsis_D0_survivor |
| FOXA3 | 1.12E-03 | 2.25 | Sepsis_D0_non-survivor up vs Sepsis_D0_survivor |
| SCNN1D | 1.16E-03 | 2.25 | Sepsis_D0_non-survivor up vs Sepsis_D0_survivor |
| GC | 6.73E-03 | 2.25 | Sepsis_D0_non-survivor up vs Sepsis_D0_survivor |
| NRIP2 | 5.38E-05 | 2.26 | Sepsis_D0_non-survivor up vs Sepsis_D0_survivor |
| GSG1 | 6.93E-04 | 2.26 | Sepsis_D0_non-survivor up vs Sepsis_D0_survivor |
| BCAR1 | 8.27E-04 | 2.27 | Sepsis_D0_non-survivor up vs Sepsis_D0_survivor |
| TNC | 3.64E-04 | 2.27 | Sepsis_D0_non-survivor up vs Sepsis_D0_survivor |
| C1QL1 | 8.70E-04 | 2.27 | Sepsis_D0_non-survivor up vs Sepsis_D0_survivor |
| CDH18 | 3.21E-03 | 2.28 | Sepsis_D0_non-survivor up vs Sepsis_D0_survivor |
| CCDC38 | 4.06E-03 | 2.28 | Sepsis_D0_non-survivor up vs Sepsis_D0_survivor |
| PLEKHH2 | 8.54E-04 | 2.29 | Sepsis_D0_non-survivor up vs Sepsis_D0_survivor |
| FGFRL1 | 4.74E-04 | 2.29 | Sepsis_D0_non-survivor up vs Sepsis_D0_survivor |
| LRRC8E | 9.95E-04 | 2.29 | Sepsis_D0_non-survivor up vs Sepsis_D0_survivor |
| HYDIN | 9.35E-05 | 2.30 | Sepsis_D0_non-survivor up vs Sepsis_D0_survivor |
| GPM6A | 4.36E-04 | 2.30 | Sepsis_D0_non-survivor up vs Sepsis_D0_survivor |
| ACCN5 | 2.29E-03 | 2.32 | Sepsis_D0_non-survivor up vs Sepsis_D0_survivor |
| UNC13B | 1.71E-04 | 2.32 | Sepsis_D0_non-survivor up vs Sepsis_D0_survivor |
| PCDHA9 | 3.77E-05 | 2.33 | Sepsis_D0_non-survivor up vs Sepsis_D0_survivor |
| PRM3 | 1.31E-03 | 2.33 | Sepsis_D0_non-survivor up vs Sepsis_D0_survivor |
| LHX9 | 1.48E-03 | 2.34 | Sepsis_D0_non-survivor up vs Sepsis_D0_survivor |
| NPAS4 | 3.06E-05 | 2.34 | Sepsis_D0_non-survivor up vs Sepsis_D0_survivor |
| KISS1R | 3.83E-04 | 2.36 | Sepsis_D0_non-survivor up vs Sepsis_D0_survivor |
| S72604 | 1.12E-03 | 2.36 | Sepsis_D0_non-survivor up vs Sepsis_D0_survivor |
| MUC4 | 9.49E-05 | 2.37 | Sepsis_D0_non-survivor up vs Sepsis_D0_survivor |
| RNASE1 | 5.33E-03 | 2.37 | Sepsis_D0_non-survivor up vs Sepsis_D0_survivor |
| OR4X2 | 1.08E-04 | 2.38 | Sepsis_D0_non-survivor up vs Sepsis_D0_survivor |
| MBL1P1 | 1.16E-04 | 2.39 | Sepsis_D0_non-survivor up vs Sepsis_D0_survivor |
| MMP21 | 6.91E-04 | 2.40 | Sepsis_D0_non-survivor up vs Sepsis_D0_survivor |
| PLVAP | 3.56E-04 | 2.41 | Sepsis_D0_non-survivor up vs Sepsis_D0_survivor |
| NBLA00301 | 2.81E-03 | 2.41 | Sepsis_D0_non-survivor up vs Sepsis_D0_survivor |
| NEUROG1 | 3.04E-05 | 2.42 | Sepsis_D0_non-survivor up vs Sepsis_D0_survivor |
| GGT6 | 2.15E-03 | 2.44 | Sepsis_D0_non-survivor up vs Sepsis_D0_survivor |
| HOXC6 | 1.45E-04 | 2.44 | Sepsis_D0_non-survivor up vs Sepsis_D0_survivor |
| IL26 | 2.62E-03 | 2.45 | Sepsis_D0_non-survivor up vs Sepsis_D0_survivor |
| CYLC2 | 4.36E-03 | 2.46 | Sepsis_D0_non-survivor up vs Sepsis_D0_survivor |
| GUCA2A | 6.93E-05 | 2.48 | Sepsis_D0_non-survivor up vs Sepsis_D0_survivor |
| CDX2 | 3.58E-03 | 2.50 | Sepsis_D0_non-survivor up vs Sepsis_D0_survivor |
| KRT18 | 5.89E-03 | 2.50 | Sepsis_D0_non-survivor up vs Sepsis_D0_survivor |
| NIPA1 | 6.50E-03 | 2.51 | Sepsis_D0_non-survivor up vs Sepsis_D0_survivor |
| SHH | 1.55E-03 | 2.51 | Sepsis_D0_non-survivor up vs Sepsis_D0_survivor |
| SPRR2C | 3.89E-04 | 2.52 | Sepsis_D0_non-survivor up vs Sepsis_D0_survivor |
| NPM2 | 1.05E-03 | 2.54 | Sepsis_D0_non-survivor up vs Sepsis_D0_survivor |
| D4S234E | 5.49E-03 | 2.55 | Sepsis_D0_non-survivor up vs Sepsis_D0_survivor |
| FMO3 | 1.65E-03 | 2.55 | Sepsis_D0_non-survivor up vs Sepsis_D0_survivor |
| HDAC10 | 1.06E-03 | 2.55 | Sepsis_D0_non-survivor up vs Sepsis_D0_survivor |
| FAM90A1 | 1.20E-03 | 2.56 | Sepsis_D0_non-survivor up vs Sepsis_D0_survivor |
| S100A14 | 4.88E-04 | 2.56 | Sepsis_D0_non-survivor up vs Sepsis_D0_survivor |
| ABCB9 | 2.12E-04 | 2.56 | Sepsis_D0_non-survivor up vs Sepsis_D0_survivor |
| WBSCR23 | 4.35E-03 | 2.57 | Sepsis_D0_non-survivor up vs Sepsis_D0_survivor |
| L40520 | 4.53E-03 | 2.57 | Sepsis_D0_non-survivor up vs Sepsis_D0_survivor |
| MAGEC1 | 3.80E-04 | 2.57 | Sepsis_D0_non-survivor up vs Sepsis_D0_survivor |
| FAM92B | 8.26E-06 | 2.58 | Sepsis_D0_non-survivor up vs Sepsis_D0_survivor |
| TRIM63 | 5.44E-05 | 2.58 | Sepsis_D0_non-survivor up vs Sepsis_D0_survivor |
| CORO6 | 9.66E-05 | 2.59 | Sepsis_D0_non-survivor up vs Sepsis_D0_survivor |
| SHANK1 | 5.16E-03 | 2.59 | Sepsis_D0_non-survivor up vs Sepsis_D0_survivor |
| ADAMTS3 | 2.94E-05 | 2.59 | Sepsis_D0_non-survivor up vs Sepsis_D0_survivor |
| ITGBL1 | 1.38E-03 | 2.60 | Sepsis_D0_non-survivor up vs Sepsis_D0_survivor |
| HAPLN1 | 5.03E-04 | 2.64 | Sepsis_D0_non-survivor up vs Sepsis_D0_survivor |
| ARSE | 6.85E-03 | 2.64 | Sepsis_D0_non-survivor up vs Sepsis_D0_survivor |
| INE1 | 1.64E-03 | 2.65 | Sepsis_D0_non-survivor up vs Sepsis_D0_survivor |
| SLC26A4 | 2.08E-03 | 2.65 | Sepsis_D0_non-survivor up vs Sepsis_D0_survivor |
| C7 | 1.80E-03 | 2.65 | Sepsis_D0_non-survivor up vs Sepsis_D0_survivor |
| PAX4 | 8.34E-04 | 2.66 | Sepsis_D0_non-survivor up vs Sepsis_D0_survivor |
| LIPE | 8.96E-05 | 2.67 | Sepsis_D0_non-survivor up vs Sepsis_D0_survivor |
| RBP2 | 6.59E-05 | 2.68 | Sepsis_D0_non-survivor up vs Sepsis_D0_survivor |
| NOL4 | 6.19E-03 | 2.68 | Sepsis_D0_non-survivor up vs Sepsis_D0_survivor |
| GAGE7 | 8.57E-06 | 2.68 | Sepsis_D0_non-survivor up vs Sepsis_D0_survivor |
| PFN4 | 4.05E-03 | 2.68 | Sepsis_D0_non-survivor up vs Sepsis_D0_survivor |
| IGKV1-5 | 8.47E-04 | 2.69 | Sepsis_D0_non-survivor up vs Sepsis_D0_survivor |
| SAA3P | 3.15E-03 | 2.70 | Sepsis_D0_non-survivor up vs Sepsis_D0_survivor |
| ZNF114 | 1.16E-04 | 2.72 | Sepsis_D0_non-survivor up vs Sepsis_D0_survivor |
| JSRP1 | 1.62E-04 | 2.76 | Sepsis_D0_non-survivor up vs Sepsis_D0_survivor |
| LIFR | 1.29E-04 | 2.77 | Sepsis_D0_non-survivor up vs Sepsis_D0_survivor |
| KCNQ1DN | 1.36E-03 | 2.79 | Sepsis_D0_non-survivor up vs Sepsis_D0_survivor |
| PRO0478 | 1.75E-03 | 2.82 | Sepsis_D0_non-survivor up vs Sepsis_D0_survivor |
| TUSC3 | 1.06E-04 | 2.83 | Sepsis_D0_non-survivor up vs Sepsis_D0_survivor |
| CXADR | 5.58E-03 | 2.84 | Sepsis_D0_non-survivor up vs Sepsis_D0_survivor |
| MMP14 | 2.46E-04 | 2.84 | Sepsis_D0_non-survivor up vs Sepsis_D0_survivor |
| ERVWE1 | 4.02E-04 | 2.89 | Sepsis_D0_non-survivor up vs Sepsis_D0_survivor |
| RALGDS | 5.10E-03 | 2.89 | Sepsis_D0_non-survivor up vs Sepsis_D0_survivor |
| RNF186 | 6.11E-05 | 2.91 | Sepsis_D0_non-survivor up vs Sepsis_D0_survivor |
| TDRD6 | 4.28E-03 | 2.91 | Sepsis_D0_non-survivor up vs Sepsis_D0_survivor |
| POTE14 | 7.63E-03 | 2.94 | Sepsis_D0_non-survivor up vs Sepsis_D0_survivor |
| SORCS3 | 1.19E-03 | 2.95 | Sepsis_D0_non-survivor up vs Sepsis_D0_survivor |
| F7 | 6.99E-05 | 2.95 | Sepsis_D0_non-survivor up vs Sepsis_D0_survivor |
| TCF1 | 9.05E-05 | 2.98 | Sepsis_D0_non-survivor up vs Sepsis_D0_survivor |
| SLIT2 | 1.05E-03 | 3.00 | Sepsis_D0_non-survivor up vs Sepsis_D0_survivor |
| PXT1 | 4.98E-05 | 3.00 | Sepsis_D0_non-survivor up vs Sepsis_D0_survivor |
| ARTN | 6.53E-03 | 3.01 | Sepsis_D0_non-survivor up vs Sepsis_D0_survivor |
| CGB1 | 4.09E-03 | 3.03 | Sepsis_D0_non-survivor up vs Sepsis_D0_survivor |
| FGA | 2.51E-03 | 3.04 | Sepsis_D0_non-survivor up vs Sepsis_D0_survivor |
| RNF151 | 8.59E-04 | 3.05 | Sepsis_D0_non-survivor up vs Sepsis_D0_survivor |
| LEPREL2 | 1.25E-03 | 3.08 | Sepsis_D0_non-survivor up vs Sepsis_D0_survivor |
| PRAC | 1.36E-03 | 3.12 | Sepsis_D0_non-survivor up vs Sepsis_D0_survivor |
| RDS | 3.21E-05 | 3.13 | Sepsis_D0_non-survivor up vs Sepsis_D0_survivor |
| K03192 | 7.40E-04 | 3.13 | Sepsis_D0_non-survivor up vs Sepsis_D0_survivor |
| GJA8 | 1.48E-04 | 3.24 | Sepsis_D0_non-survivor up vs Sepsis_D0_survivor |
| RGPD2 | 2.09E-03 | 3.27 | Sepsis_D0_non-survivor up vs Sepsis_D0_survivor |
| GLYATL1 | 2.77E-03 | 3.29 | Sepsis_D0_non-survivor up vs Sepsis_D0_survivor |
| OR4N4 | 7.01E-04 | 3.54 | Sepsis_D0_non-survivor up vs Sepsis_D0_survivor |
| IGSF10 | 1.17E-04 | 3.61 | Sepsis_D0_non-survivor up vs Sepsis_D0_survivor |
| PTPRB | 7.90E-05 | 3.73 | Sepsis_D0_non-survivor up vs Sepsis_D0_survivor |
| CHRDL1 | 6.79E-03 | 3.75 | Sepsis_D0_non-survivor up vs Sepsis_D0_survivor |
| CBLN1 | 4.27E-03 | 3.82 | Sepsis_D0_non-survivor up vs Sepsis_D0_survivor |
| CAPN14 | 1.91E-03 | 4.03 | Sepsis_D0_non-survivor up vs Sepsis_D0_survivor |
| S81524 | 1.14E-03 | 4.19 | Sepsis_D0_non-survivor up vs Sepsis_D0_survivor |
| GPR120 | 1.02E-03 | 4.20 | Sepsis_D0_non-survivor up vs Sepsis_D0_survivor |
| OPCML | 2.70E-04 | 5.33 | Sepsis_D0_non-survivor up vs Sepsis_D0_survivor |
| CLDN8 | 1.34E-03 | 5.51 | Sepsis_D0_non-survivor up vs Sepsis_D0_survivor |
| BMP4 | 2.51E-03 | 5.64 | Sepsis_D0_non-survivor up vs Sepsis_D0_survivor |
| PNLIPRP2 | 1.78E-04 | 6.44 | Sepsis_D0_non-survivor up vs Sepsis_D0_survivor |
| CRYBA1 | 1.27E-05 | 6.48 | Sepsis_D0_non-survivor up vs Sepsis_D0_survivor |
| KCNT1 | 5.56E-04 | 6.71 | Sepsis_D0_non-survivor up vs Sepsis_D0_survivor |
| CRLF1 | 3.85E-04 | 7.66 | Sepsis_D0_non-survivor up vs Sepsis_D0_survivor |
| FAM118A | 6.28E-04 | 8.94 | Sepsis_D0_non-survivor up vs Sepsis_D0_survivor |
| RPAP1 | 4.56E-03 | 9.70 | Sepsis_D0_non-survivor up vs Sepsis_D0_survivor |
| PCDHGB2 | 2.37E-03 | 10.28 | Sepsis_D0_non-survivor up vs Sepsis_D0_survivor |
| WDR76 | 1.48E-05 | 11.52 | Sepsis_D0_non-survivor up vs Sepsis_D0_survivor |
| GPR88 | 3.21E-03 | 12.73 | Sepsis_D0_non-survivor up vs Sepsis_D0_survivor |
| GABRE | 1.60E-03 | 12.98 | Sepsis_D0_non-survivor up vs Sepsis_D0_survivor |
| ATP1A2 | 6.26E-06 | 14.74 | Sepsis_D0_non-survivor up vs Sepsis_D0_survivor |
| PMCHL1 | 2.32E-03 | 17.11 | Sepsis_D0_non-survivor up vs Sepsis_D0_survivor |
| XKRX | 3.01E-03 | 46.70 | Sepsis_D0_non-survivor up vs Sepsis_D0_survivor |
