# Supplementary material for: Patterns of Gene Expression in Peripheral Blood Mononuclear Cells and Outcomes from Patients with Sepsis Secondary to Community Acquired Pneumonia
Source: PLoS One. 2014 Mar 25;9(3):e91886. doi: 10.1371/journal.pone.0091886 (PMC3965402; doi:10.1371/journal.pone.0091886)
Supplement: Table S3 — Differential gene expression between survivors at D0 and survivors at D7. Only genes presenting at least a 1.7 fold change are reported. (DOCX) [file pone.0091886.s003.docx]

**Table S3: Differential gene expression between survivors at D0 and survivors at D7. Only genes presenting at least a 1.7 fold change are reported.**

| Gene  Symbol | FoldChange  D0_survivor vs. D7_survivor | FoldChange Description |
| --- | --- | --- |
| CKMT2 | -32.70 | D0_survivor down vs D7_survivor |
| MAGI1 | -7.55 | D0_survivor down vs D7_survivor |
| SPRR4 | -6.00 | D0_survivor down vs D7_survivor |
| RWDD2 | -4.67 | D0_survivor down vs D7_survivor |
| CRYBA1 | -4.54 | D0_survivor down vs D7_survivor |
| GSTM3 | -4.42 | D0_survivor down vs D7_survivor |
| GPR37 | -4.26 | D0_survivor down vs D7_survivor |
| ARCN1 | -4.23 | D0_survivor down vs D7_survivor |
| PLCE1 | -3.75 | D0_survivor down vs D7_survivor |
| SYMPK | -3.44 | D0_survivor down vs D7_survivor |
| PERP | -3.43 | D0_survivor down vs D7_survivor |
| PLXNA2 | -3.22 | D0_survivor down vs D7_survivor |
| OTUD4 | -3.07 | D0_survivor down vs D7_survivor |
| RGPD2 | -2.94 | D0_survivor down vs D7_survivor |
| CYP4F11 | -2.94 | D0_survivor down vs D7_survivor |
| ULBP1 | -2.94 | D0_survivor down vs D7_survivor |
| TULP4 | -2.92 | D0_survivor down vs D7_survivor |
| SLFN13 | -2.92 | D0_survivor down vs D7_survivor |
| PAX3 | -2.92 | D0_survivor down vs D7_survivor |
| KLRC2 | -2.90 | D0_survivor down vs D7_survivor |
| CCL7 | -2.76 | D0_survivor down vs D7_survivor |
| SGCD | -2.75 | D0_survivor down vs D7_survivor |
| SPTA1 | -2.74 | D0_survivor down vs D7_survivor |
| PMCHL1 | -2.73 | D0_survivor down vs D7_survivor |
| PPP1R14C | -2.72 | D0_survivor down vs D7_survivor |
| CMTM8 | -2.70 | D0_survivor down vs D7_survivor |
| MLLT6 | -2.70 | D0_survivor down vs D7_survivor |
| MATN3 | -2.65 | D0_survivor down vs D7_survivor |
| BNC1 | -2.64 | D0_survivor down vs D7_survivor |
| EPHA5 | -2.60 | D0_survivor down vs D7_survivor |
| POFUT1 | -2.52 | D0_survivor down vs D7_survivor |
| PPARGC1A | -2.46 | D0_survivor down vs D7_survivor |
| CDH26 | -2.45 | D0_survivor down vs D7_survivor |
| SCARNA17 | -2.44 | D0_survivor down vs D7_survivor |
| ZRANB3 | -2.42 | D0_survivor down vs D7_survivor |
| ADAMTS3 | -2.40 | D0_survivor down vs D7_survivor |
| SLC6A8 | -2.40 | D0_survivor down vs D7_survivor |
| KIAA1345 | -2.36 | D0_survivor down vs D7_survivor |
| EXOSC6 | -2.35 | D0_survivor down vs D7_survivor |
| KRT1 | -2.34 | D0_survivor down vs D7_survivor |
| LOXL4 | -2.34 | D0_survivor down vs D7_survivor |
| ACACB | -2.32 | D0_survivor down vs D7_survivor |
| NT5DC3 | -2.31 | D0_survivor down vs D7_survivor |
| FAM84A | -2.30 | D0_survivor down vs D7_survivor |
| TTLL7 | -2.30 | D0_survivor down vs D7_survivor |
| SCRG1 | -2.29 | D0_survivor down vs D7_survivor |
| ARVCF | -2.27 | D0_survivor down vs D7_survivor |
| ALDH1A1 | -2.26 | D0_survivor down vs D7_survivor |
| SPRYD5 | -2.26 | D0_survivor down vs D7_survivor |
| ZNF566 | -2.24 | D0_survivor down vs D7_survivor |
| PITPNM3 | -2.23 | D0_survivor down vs D7_survivor |
| LTF | -2.22 | D0_survivor down vs D7_survivor |
| CYP26A1 | -2.22 | D0_survivor down vs D7_survivor |
| DPT | -2.21 | D0_survivor down vs D7_survivor |
| GK2 | -2.20 | D0_survivor down vs D7_survivor |
| NRXN1 | -2.20 | D0_survivor down vs D7_survivor |
| IZUMO1 | -2.18 | D0_survivor down vs D7_survivor |
| H19 | -2.18 | D0_survivor down vs D7_survivor |
| MMP3 | -2.17 | D0_survivor down vs D7_survivor |
| CFB | -2.16 | D0_survivor down vs D7_survivor |
| NFIX | -2.16 | D0_survivor down vs D7_survivor |
| IGJ | -2.15 | D0_survivor down vs D7_survivor |
| ATRN | -2.15 | D0_survivor down vs D7_survivor |
| GNAO1 | -2.14 | D0_survivor down vs D7_survivor |
| HBD | -2.13 | D0_survivor down vs D7_survivor |
| ANK1 | -2.13 | D0_survivor down vs D7_survivor |
| ADCY2 | -2.12 | D0_survivor down vs D7_survivor |
| PTPN21 | -2.11 | D0_survivor down vs D7_survivor |
| SH2D1B | -2.11 | D0_survivor down vs D7_survivor |
| APOL6 | -2.09 | D0_survivor down vs D7_survivor |
| DDX53 | -2.09 | D0_survivor down vs D7_survivor |
| CNTNAP2 | -2.09 | D0_survivor down vs D7_survivor |
| ELA3B | -2.09 | D0_survivor down vs D7_survivor |
| ZNF138 | -2.07 | D0_survivor down vs D7_survivor |
| DEPDC1 | -2.06 | D0_survivor down vs D7_survivor |
| ABCA13 | -2.05 | D0_survivor down vs D7_survivor |
| TSPAN5 | -2.05 | D0_survivor down vs D7_survivor |
| ZNF507 | -2.05 | D0_survivor down vs D7_survivor |
| ANK1 | -2.04 | D0_survivor down vs D7_survivor |
| TTTY4C | -2.03 | D0_survivor down vs D7_survivor |
| PLIN | -2.02 | D0_survivor down vs D7_survivor |
| GYPA | -2.02 | D0_survivor down vs D7_survivor |
| ERAF | -2.01 | D0_survivor down vs D7_survivor |
| CASP8 | -2.00 | D0_survivor down vs D7_survivor |
| TDRD6 | -2.00 | D0_survivor down vs D7_survivor |
| ITGAD | -2.00 | D0_survivor down vs D7_survivor |
| RALY | -2.00 | D0_survivor down vs D7_survivor |
| MXRA8 | -2.00 | D0_survivor down vs D7_survivor |
| DPP4 | -2.00 | D0_survivor down vs D7_survivor |
| CLN5 | -2.00 | D0_survivor down vs D7_survivor |
| CHI3L1 | -2.00 | D0_survivor down vs D7_survivor |
| MMP1 | -1.99 | D0_survivor down vs D7_survivor |
| OLIG1 | -1.99 | D0_survivor down vs D7_survivor |
| SPDYA | -1.99 | D0_survivor down vs D7_survivor |
| PRMT8 | -1.98 | D0_survivor down vs D7_survivor |
| DACH2 | -1.98 | D0_survivor down vs D7_survivor |
| CD28 | -1.98 | D0_survivor down vs D7_survivor |
| MGC16384 | -1.98 | D0_survivor down vs D7_survivor |
| GATA3 | -1.98 | D0_survivor down vs D7_survivor |
| CAMP | -1.98 | D0_survivor down vs D7_survivor |
| ARG1 | -1.97 | D0_survivor down vs D7_survivor |
| SLC26A4 | -1.96 | D0_survivor down vs D7_survivor |
| SEMA7A | -1.96 | D0_survivor down vs D7_survivor |
| LEF1 | -1.95 | D0_survivor down vs D7_survivor |
| ACSL6 | -1.94 | D0_survivor down vs D7_survivor |
| LONRF1 | -1.93 | D0_survivor down vs D7_survivor |
| FGF9 | -1.93 | D0_survivor down vs D7_survivor |
| EPHA4 | -1.93 | D0_survivor down vs D7_survivor |
| TBX21 | -1.93 | D0_survivor down vs D7_survivor |
| STXBP6 | -1.93 | D0_survivor down vs D7_survivor |
| AZGP1 | -1.91 | D0_survivor down vs D7_survivor |
| RASGRF1 | -1.91 | D0_survivor down vs D7_survivor |
| LGI2 | -1.91 | D0_survivor down vs D7_survivor |
| WNT8A | -1.91 | D0_survivor down vs D7_survivor |
| ITIH5 | -1.90 | D0_survivor down vs D7_survivor |
| MUC13 | -1.90 | D0_survivor down vs D7_survivor |
| PF4V1 | -1.90 | D0_survivor down vs D7_survivor |
| INSRR | -1.89 | D0_survivor down vs D7_survivor |
| TSPAN5 | -1.89 | D0_survivor down vs D7_survivor |
| CASP12 | -1.88 | D0_survivor down vs D7_survivor |
| CYP4F3 | -1.88 | D0_survivor down vs D7_survivor |
| TRIM59 | -1.88 | D0_survivor down vs D7_survivor |
| KLHL24 | -1.88 | D0_survivor down vs D7_survivor |
| G31710 | -1.88 | D0_survivor down vs D7_survivor |
| CCDC65 | -1.87 | D0_survivor down vs D7_survivor |
| UNQ6488 | -1.87 | D0_survivor down vs D7_survivor |
| PRO2949 | -1.87 | D0_survivor down vs D7_survivor |
| SHPRH | -1.87 | D0_survivor down vs D7_survivor |
| BNC2 | -1.86 | D0_survivor down vs D7_survivor |
| PGC | -1.86 | D0_survivor down vs D7_survivor |
| FOXP2 | -1.86 | D0_survivor down vs D7_survivor |
| RSHL3 | -1.85 | D0_survivor down vs D7_survivor |
| NT5C1B | -1.84 | D0_survivor down vs D7_survivor |
| SLC25A10 | -1.84 | D0_survivor down vs D7_survivor |
| DLEC1 | -1.84 | D0_survivor down vs D7_survivor |
| TRIM32 | -1.84 | D0_survivor down vs D7_survivor |
| SLC22A3 | -1.84 | D0_survivor down vs D7_survivor |
| CCL2 | -1.83 | D0_survivor down vs D7_survivor |
| SYTL2 | -1.83 | D0_survivor down vs D7_survivor |
| POPDC3 | -1.83 | D0_survivor down vs D7_survivor |
| GREM2 | -1.82 | D0_survivor down vs D7_survivor |
| HSF2BP | -1.82 | D0_survivor down vs D7_survivor |
| PP8961 | -1.82 | D0_survivor down vs D7_survivor |
| DHDDS | -1.81 | D0_survivor down vs D7_survivor |
| CBX2 | -1.81 | D0_survivor down vs D7_survivor |
| SLC4A4 | -1.81 | D0_survivor down vs D7_survivor |
| HIG2 | -1.81 | D0_survivor down vs D7_survivor |
| MRPS11 | -1.81 | D0_survivor down vs D7_survivor |
| ACP1 | -1.80 | D0_survivor down vs D7_survivor |
| CRISP2 | -1.80 | D0_survivor down vs D7_survivor |
| NCKIPSD | -1.80 | D0_survivor down vs D7_survivor |
| GIMAP5 | -1.80 | D0_survivor down vs D7_survivor |
| PRKAB2 | -1.80 | D0_survivor down vs D7_survivor |
| INSAF | -1.80 | D0_survivor down vs D7_survivor |
| TRIM65 | -1.79 | D0_survivor down vs D7_survivor |
| TMC6 | -1.79 | D0_survivor down vs D7_survivor |
| TIE1 | -1.79 | D0_survivor down vs D7_survivor |
| EPB42 | -1.79 | D0_survivor down vs D7_survivor |
| KIAA0773 | -1.79 | D0_survivor down vs D7_survivor |
| CNTNAP3 | -1.78 | D0_survivor down vs D7_survivor |
| FECH | -1.78 | D0_survivor down vs D7_survivor |
| CAPS2 | -1.78 | D0_survivor down vs D7_survivor |
| NPAT | -1.78 | D0_survivor down vs D7_survivor |
| EDG1 | -1.78 | D0_survivor down vs D7_survivor |
| PSCA | -1.78 | D0_survivor down vs D7_survivor |
| GAD2 | -1.78 | D0_survivor down vs D7_survivor |
| LAX1 | -1.78 | D0_survivor down vs D7_survivor |
| HRASLS | -1.77 | D0_survivor down vs D7_survivor |
| ZNF14 | -1.77 | D0_survivor down vs D7_survivor |
| SPSB1 | -1.77 | D0_survivor down vs D7_survivor |
| ITK | -1.76 | D0_survivor down vs D7_survivor |
| RSNL2 | -1.76 | D0_survivor down vs D7_survivor |
| ADAMTS1 | -1.76 | D0_survivor down vs D7_survivor |
| RDS | -1.76 | D0_survivor down vs D7_survivor |
| TNS1 | -1.75 | D0_survivor down vs D7_survivor |
| XG | -1.75 | D0_survivor down vs D7_survivor |
| NARG2 | -1.75 | D0_survivor down vs D7_survivor |
| CDO1 | -1.75 | D0_survivor down vs D7_survivor |
| CLIC3 | -1.75 | D0_survivor down vs D7_survivor |
| ZC3H12D | -1.74 | D0_survivor down vs D7_survivor |
| MYST4 | -1.74 | D0_survivor down vs D7_survivor |
| STAT4 | -1.74 | D0_survivor down vs D7_survivor |
| SLC6A10P | -1.74 | D0_survivor down vs D7_survivor |
| CHIT1 | -1.74 | D0_survivor down vs D7_survivor |
| RTTN | -1.74 | D0_survivor down vs D7_survivor |
| TBX15 | -1.73 | D0_survivor down vs D7_survivor |
| GYPA | -1.73 | D0_survivor down vs D7_survivor |
| TEX14 | -1.73 | D0_survivor down vs D7_survivor |
| MOCS3 | -1.73 | D0_survivor down vs D7_survivor |
| TTTY1 | -1.73 | D0_survivor down vs D7_survivor |
| ARHGAP10 | -1.73 | D0_survivor down vs D7_survivor |
| MGC16037 | -1.73 | D0_survivor down vs D7_survivor |
| DCP_1_11 | -1.72 | D0_survivor down vs D7_survivor |
| KSP37 | -1.72 | D0_survivor down vs D7_survivor |
| EPB41L4A | -1.72 | D0_survivor down vs D7_survivor |
| GP5 | -1.72 | D0_survivor down vs D7_survivor |
| BPGM | -1.72 | D0_survivor down vs D7_survivor |
| PMCH | -1.72 | D0_survivor down vs D7_survivor |
| ALS2CR7 | -1.71 | D0_survivor down vs D7_survivor |
| FAM84B | -1.70 | D0_survivor down vs D7_survivor |
| PLN | -1.70 | D0_survivor down vs D7_survivor |
| MYL4 | -1.70 | D0_survivor down vs D7_survivor |
| KLHDC6 | -1.70 | D0_survivor down vs D7_survivor |
| GUCY2C | -1.70 | D0_survivor down vs D7_survivor |
| EDNRB | 1.70 | D0_survivor up vs D7_survivor |
| LRRTM2 | 1.70 | D0_survivor up vs D7_survivor |
| SERPINC1 | 1.70 | D0_survivor up vs D7_survivor |
| ASPM | 1.70 | D0_survivor up vs D7_survivor |
| GALC | 1.70 | D0_survivor up vs D7_survivor |
| GGH | 1.71 | D0_survivor up vs D7_survivor |
| MS4A8B | 1.71 | D0_survivor up vs D7_survivor |
| U01925 | 1.71 | D0_survivor up vs D7_survivor |
| TNRC6A | 1.71 | D0_survivor up vs D7_survivor |
| TNRC4 | 1.71 | D0_survivor up vs D7_survivor |
| USP13 | 1.71 | D0_survivor up vs D7_survivor |
| CSMD2 | 1.72 | D0_survivor up vs D7_survivor |
| SEMA6D | 1.72 | D0_survivor up vs D7_survivor |
| ANKRD22 | 1.73 | D0_survivor up vs D7_survivor |
| HIP1 | 1.73 | D0_survivor up vs D7_survivor |
| MPO | 1.73 | D0_survivor up vs D7_survivor |
| ST8SIA1 | 1.73 | D0_survivor up vs D7_survivor |
| DMD | 1.73 | D0_survivor up vs D7_survivor |
| HTRA3 | 1.73 | D0_survivor up vs D7_survivor |
| IFI27 | 1.74 | D0_survivor up vs D7_survivor |
| SSX6 | 1.74 | D0_survivor up vs D7_survivor |
| TTC8 | 1.74 | D0_survivor up vs D7_survivor |
| BIRC5 | 1.75 | D0_survivor up vs D7_survivor |
| KBTBD6 | 1.75 | D0_survivor up vs D7_survivor |
| USP49 | 1.75 | D0_survivor up vs D7_survivor |
| CRISP1 | 1.75 | D0_survivor up vs D7_survivor |
| TEX13B | 1.75 | D0_survivor up vs D7_survivor |
| PLCZ1 | 1.75 | D0_survivor up vs D7_survivor |
| VSIG4 | 1.75 | D0_survivor up vs D7_survivor |
| NHLRC2 | 1.76 | D0_survivor up vs D7_survivor |
| MARCO | 1.76 | D0_survivor up vs D7_survivor |
| UBE2C | 1.76 | D0_survivor up vs D7_survivor |
| LUZP4 | 1.76 | D0_survivor up vs D7_survivor |
| PHKA1 | 1.76 | D0_survivor up vs D7_survivor |
| SF3B4 | 1.76 | D0_survivor up vs D7_survivor |
| CDCA2 | 1.77 | D0_survivor up vs D7_survivor |
| PHLDB2 | 1.77 | D0_survivor up vs D7_survivor |
| MGC50722 | 1.77 | D0_survivor up vs D7_survivor |
| HIP1 | 1.77 | D0_survivor up vs D7_survivor |
| ODF2L | 1.78 | D0_survivor up vs D7_survivor |
| FGF12 | 1.78 | D0_survivor up vs D7_survivor |
| GSG2 | 1.79 | D0_survivor up vs D7_survivor |
| HMG20A | 1.79 | D0_survivor up vs D7_survivor |
| SUV420H1 | 1.79 | D0_survivor up vs D7_survivor |
| PDPN | 1.79 | D0_survivor up vs D7_survivor |
| CCNB2 | 1.79 | D0_survivor up vs D7_survivor |
| CNTN2 | 1.79 | D0_survivor up vs D7_survivor |
| METTL7B | 1.80 | D0_survivor up vs D7_survivor |
| BPIL3 | 1.80 | D0_survivor up vs D7_survivor |
| NT5DC3 | 1.80 | D0_survivor up vs D7_survivor |
| MT | 1.80 | D0_survivor up vs D7_survivor |
| CYP1A2 | 1.80 | D0_survivor up vs D7_survivor |
| GINS1 | 1.81 | D0_survivor up vs D7_survivor |
| CDH5 | 1.81 | D0_survivor up vs D7_survivor |
| ANXA13 | 1.81 | D0_survivor up vs D7_survivor |
| PPAPDC1B | 1.81 | D0_survivor up vs D7_survivor |
| MGC40574 | 1.81 | D0_survivor up vs D7_survivor |
| BMP3 | 1.82 | D0_survivor up vs D7_survivor |
| MARS2 | 1.83 | D0_survivor up vs D7_survivor |
| HPR | 1.83 | D0_survivor up vs D7_survivor |
| IFI27 | 1.84 | D0_survivor up vs D7_survivor |
| T52140 | 1.84 | D0_survivor up vs D7_survivor |
| GCKR | 1.85 | D0_survivor up vs D7_survivor |
| CRYGA | 1.85 | D0_survivor up vs D7_survivor |
| MTHFD2L | 1.86 | D0_survivor up vs D7_survivor |
| SCN10A | 1.87 | D0_survivor up vs D7_survivor |
| ACSL6 | 1.88 | D0_survivor up vs D7_survivor |
| GPSM2 | 1.89 | D0_survivor up vs D7_survivor |
| SEMA3E | 1.89 | D0_survivor up vs D7_survivor |
| MTNR1B | 1.89 | D0_survivor up vs D7_survivor |
| VAV2 | 1.89 | D0_survivor up vs D7_survivor |
| TSSK2 | 1.90 | D0_survivor up vs D7_survivor |
| DCTN1 | 1.90 | D0_survivor up vs D7_survivor |
| RNF43 | 1.90 | D0_survivor up vs D7_survivor |
| SLC22A9 | 1.90 | D0_survivor up vs D7_survivor |
| NT5C1B | 1.90 | D0_survivor up vs D7_survivor |
| IGSF4D | 1.91 | D0_survivor up vs D7_survivor |
| PKIB | 1.91 | D0_survivor up vs D7_survivor |
| GTF2I | 1.91 | D0_survivor up vs D7_survivor |
| CNGB3 | 1.91 | D0_survivor up vs D7_survivor |
| CR1 | 1.91 | D0_survivor up vs D7_survivor |
| IL2RA | 1.92 | D0_survivor up vs D7_survivor |
| CETP | 1.92 | D0_survivor up vs D7_survivor |
| ANLN | 1.93 | D0_survivor up vs D7_survivor |
| PADI1 | 1.93 | D0_survivor up vs D7_survivor |
| ZNF512 | 1.93 | D0_survivor up vs D7_survivor |
| IL28B | 1.93 | D0_survivor up vs D7_survivor |
| SLC5A6 | 1.94 | D0_survivor up vs D7_survivor |
| RTP1 | 1.94 | D0_survivor up vs D7_survivor |
| PPARG | 1.94 | D0_survivor up vs D7_survivor |
| CPZ | 1.95 | D0_survivor up vs D7_survivor |
| NALP5 | 1.95 | D0_survivor up vs D7_survivor |
| GIT1 | 1.95 | D0_survivor up vs D7_survivor |
| TDGF1 | 1.96 | D0_survivor up vs D7_survivor |
| LSM14B | 1.97 | D0_survivor up vs D7_survivor |
| GDAP1 | 1.97 | D0_survivor up vs D7_survivor |
| TCF7L2 | 1.97 | D0_survivor up vs D7_survivor |
| SERPINB12 | 1.98 | D0_survivor up vs D7_survivor |
| ELA1 | 1.98 | D0_survivor up vs D7_survivor |
| HCG9 | 1.99 | D0_survivor up vs D7_survivor |
| DTX1 | 1.99 | D0_survivor up vs D7_survivor |
| FAM64A | 1.99 | D0_survivor up vs D7_survivor |
| IL17RB | 2.00 | D0_survivor up vs D7_survivor |
| POU4F3 | 2.00 | D0_survivor up vs D7_survivor |
| FNDC3A | 2.05 | D0_survivor up vs D7_survivor |
| ARTS-1 | 2.07 | D0_survivor up vs D7_survivor |
| SLC4A11 | 2.07 | D0_survivor up vs D7_survivor |
| IBSP | 2.07 | D0_survivor up vs D7_survivor |
| CIT | 2.08 | D0_survivor up vs D7_survivor |
| YAP1 | 2.10 | D0_survivor up vs D7_survivor |
| CDKN3 | 2.11 | D0_survivor up vs D7_survivor |
| IFNA10 | 2.11 | D0_survivor up vs D7_survivor |
| NOM1 | 2.13 | D0_survivor up vs D7_survivor |
| TMEM2 | 2.13 | D0_survivor up vs D7_survivor |
| ADAMTS4 | 2.15 | D0_survivor up vs D7_survivor |
| FAM20A | 2.17 | D0_survivor up vs D7_survivor |
| HP | 2.18 | D0_survivor up vs D7_survivor |
| SFRS12 | 2.19 | D0_survivor up vs D7_survivor |
| FAM20A | 2.19 | D0_survivor up vs D7_survivor |
| DEFB106A | 2.19 | D0_survivor up vs D7_survivor |
| LGALS13 | 2.20 | D0_survivor up vs D7_survivor |
| MGC4268 | 2.26 | D0_survivor up vs D7_survivor |
| MAGEC2 | 2.28 | D0_survivor up vs D7_survivor |
| CCR9 | 2.28 | D0_survivor up vs D7_survivor |
| PRKAG3 | 2.29 | D0_survivor up vs D7_survivor |
| PER3 | 2.30 | D0_survivor up vs D7_survivor |
| CEP55 | 2.30 | D0_survivor up vs D7_survivor |
| ABRA | 2.30 | D0_survivor up vs D7_survivor |
| ADAM20 | 2.32 | D0_survivor up vs D7_survivor |
| DIAPH3 | 2.38 | D0_survivor up vs D7_survivor |
| CALML5 | 2.38 | D0_survivor up vs D7_survivor |
| FSCN1 | 2.39 | D0_survivor up vs D7_survivor |
| RASSF8 | 2.43 | D0_survivor up vs D7_survivor |
| TMEM12 | 2.43 | D0_survivor up vs D7_survivor |
| SENP6 | 2.47 | D0_survivor up vs D7_survivor |
| CRSP6 | 2.49 | D0_survivor up vs D7_survivor |
| QRSL1 | 2.49 | D0_survivor up vs D7_survivor |
| LRAT | 2.50 | D0_survivor up vs D7_survivor |
| TOP2A | 2.50 | D0_survivor up vs D7_survivor |
| FGF7 | 2.51 | D0_survivor up vs D7_survivor |
| TBC1D16 | 2.53 | D0_survivor up vs D7_survivor |
| NF1 | 2.54 | D0_survivor up vs D7_survivor |
| USH2A | 2.55 | D0_survivor up vs D7_survivor |
| CENPA | 2.57 | D0_survivor up vs D7_survivor |
| GYS2 | 2.60 | D0_survivor up vs D7_survivor |
| L27175 | 2.63 | D0_survivor up vs D7_survivor |
| ICA1L | 2.65 | D0_survivor up vs D7_survivor |
| TJP1 | 2.68 | D0_survivor up vs D7_survivor |
| MFAP3 | 2.70 | D0_survivor up vs D7_survivor |
| RETN | 2.70 | D0_survivor up vs D7_survivor |
| FILIP1 | 2.71 | D0_survivor up vs D7_survivor |
| FAM84A | 2.72 | D0_survivor up vs D7_survivor |
| MAGEB4 | 2.72 | D0_survivor up vs D7_survivor |
| GINS4 | 2.75 | D0_survivor up vs D7_survivor |
| WDR76 | 2.75 | D0_survivor up vs D7_survivor |
| NIPA1 | 2.77 | D0_survivor up vs D7_survivor |
| POLK | 2.83 | D0_survivor up vs D7_survivor |
| NPR3 | 2.84 | D0_survivor up vs D7_survivor |
| NAALAD2 | 2.87 | D0_survivor up vs D7_survivor |
| SEMG2 | 2.94 | D0_survivor up vs D7_survivor |
| ADAMTS2 | 2.95 | D0_survivor up vs D7_survivor |
| FHL5 | 3.13 | D0_survivor up vs D7_survivor |
| KCNMB2 | 3.15 | D0_survivor up vs D7_survivor |
| RSPO3 | 3.19 | D0_survivor up vs D7_survivor |
| KRTAP1-1 | 3.32 | D0_survivor up vs D7_survivor |
| CAPN14 | 3.44 | D0_survivor up vs D7_survivor |
| RFC1 | 3.47 | D0_survivor up vs D7_survivor |
| ELL3 | 3.51 | D0_survivor up vs D7_survivor |
| ZNF682 | 3.53 | D0_survivor up vs D7_survivor |
| COMP | 3.66 | D0_survivor up vs D7_survivor |
| PCOLCE2 | 3.85 | D0_survivor up vs D7_survivor |
| RP11-564C4.1 | 4.05 | D0_survivor up vs D7_survivor |
| NALP13 | 5.36 | D0_survivor up vs D7_survivor |
| UGT1A8 | 5.71 | D0_survivor up vs D7_survivor |
| RAPGEF4 | 6.05 | D0_survivor up vs D7_survivor |
| MBNL3 | 12.04 | D0_survivor up vs D7_survivor |
| GABRB3 | 15.13 | D0_survivor up vs D7_survivor |
