# Supplementary material for: Patterns of Gene Expression in Peripheral Blood Mononuclear Cells and Outcomes from Patients with Sepsis Secondary to Community Acquired Pneumonia
Source: PLoS One. 2014 Mar 25;9(3):e91886. doi: 10.1371/journal.pone.0091886 (PMC3965402; doi:10.1371/journal.pone.0091886)
Supplement: Table S4 — Differential gene expression between non-survivors at D0 and non-survivors at D7. Only genes presenting at least a 1.7 fold change are reported. (DOCX) [file pone.0091886.s004.docx]

**Table S4: Differential gene expression between non-survivors at D0 and non-survivors at D7. Only genes presenting at least a 1.7 fold change are reported.**

| Gene Symbol | FoldChange D0_non-survivor vs. D7_non-survivor) | FoldChange Description |
| --- | --- | --- |
| OLFM4 | -92.08 | D0_non-survivor down vs D7_non-survivor |
| CXADR | -9.15 | D0_non-survivor down vs D7_non-survivor |
| ARG1 | -8.44 | D0_non-survivor down vs D7_non-survivor |
| ZNF702 | -6.85 | D0_non-survivor down vs D7_non-survivor |
| WBP2NL | -5.61 | D0_non-survivor down vs D7_non-survivor |
| GPR116 | -5.16 | D0_non-survivor down vs D7_non-survivor |
| FLJ23577 | -4.17 | D0_non-survivor down vs D7_non-survivor |
| TDGF3 | -4.17 | D0_non-survivor down vs D7_non-survivor |
| LTF | -4.00 | D0_non-survivor down vs D7_non-survivor |
| PCSK9 | -3.79 | D0_non-survivor down vs D7_non-survivor |
| FAM62C | -3.68 | D0_non-survivor down vs D7_non-survivor |
| CYP4F3 | -3.52 | D0_non-survivor down vs D7_non-survivor |
| CLSTN2 | -3.52 | D0_non-survivor down vs D7_non-survivor |
| IQCF1 | -3.51 | D0_non-survivor down vs D7_non-survivor |
| IL1R2 | -3.20 | D0_non-survivor down vs D7_non-survivor |
| CNTNAP4 | -3.03 | D0_non-survivor down vs D7_non-survivor |
| PRO1483 | -2.97 | D0_non-survivor down vs D7_non-survivor |
| CALN1 | -2.97 | D0_non-survivor down vs D7_non-survivor |
| FLT4 | -2.89 | D0_non-survivor down vs D7_non-survivor |
| ALAS2 | -2.85 | D0_non-survivor down vs D7_non-survivor |
| SELENBP1 | -2.83 | D0_non-survivor down vs D7_non-survivor |
| X77737 | -2.79 | D0_non-survivor down vs D7_non-survivor |
| NEIL3 | -2.74 | D0_non-survivor down vs D7_non-survivor |
| EDA | -2.73 | D0_non-survivor down vs D7_non-survivor |
| HBD | -2.71 | D0_non-survivor down vs D7_non-survivor |
| PCDH21 | -2.56 | D0_non-survivor down vs D7_non-survivor |
| LRRC27 | -2.56 | D0_non-survivor down vs D7_non-survivor |
| SYNPO2 | -2.50 | D0_non-survivor down vs D7_non-survivor |
| NUCKS1 | -2.50 | D0_non-survivor down vs D7_non-survivor |
| ALAS2 | -2.47 | D0_non-survivor down vs D7_non-survivor |
| ALB | -2.46 | D0_non-survivor down vs D7_non-survivor |
| ETNK2 | -2.43 | D0_non-survivor down vs D7_non-survivor |
| CEACAM8 | -2.39 | D0_non-survivor down vs D7_non-survivor |
| TNS1 | -2.36 | D0_non-survivor down vs D7_non-survivor |
| SIX4 | -2.34 | D0_non-survivor down vs D7_non-survivor |
| CH25H | -2.30 | D0_non-survivor down vs D7_non-survivor |
| DUSP13 | -2.26 | D0_non-survivor down vs D7_non-survivor |
| UGT3A1 | -2.25 | D0_non-survivor down vs D7_non-survivor |
| PARP14 | -2.24 | D0_non-survivor down vs D7_non-survivor |
| CCL15 | -2.23 | D0_non-survivor down vs D7_non-survivor |
| HOXA9 | -2.23 | D0_non-survivor down vs D7_non-survivor |
| DEFA3 | -2.22 | D0_non-survivor down vs D7_non-survivor |
| OLAH | -2.21 | D0_non-survivor down vs D7_non-survivor |
| KIAA1804 | -2.20 | D0_non-survivor down vs D7_non-survivor |
| DQX1 | -2.18 | D0_non-survivor down vs D7_non-survivor |
| INADL | -2.18 | D0_non-survivor down vs D7_non-survivor |
| KLHL14 | -2.18 | D0_non-survivor down vs D7_non-survivor |
| HBD | -2.17 | D0_non-survivor down vs D7_non-survivor |
| CDH13 | -2.16 | D0_non-survivor down vs D7_non-survivor |
| SAP30 | -2.11 | D0_non-survivor down vs D7_non-survivor |
| TMEM33 | -2.11 | D0_non-survivor down vs D7_non-survivor |
| ADORA3 | -2.10 | D0_non-survivor down vs D7_non-survivor |
| CHI3L1 | -2.09 | D0_non-survivor down vs D7_non-survivor |
| CA1 | -2.08 | D0_non-survivor down vs D7_non-survivor |
| KCNE3 | -2.07 | D0_non-survivor down vs D7_non-survivor |
| UEVLD | -2.05 | D0_non-survivor down vs D7_non-survivor |
| TPST1 | -2.02 | D0_non-survivor down vs D7_non-survivor |
| ARMC9 | -2.02 | D0_non-survivor down vs D7_non-survivor |
| LRRC7 | -2.02 | D0_non-survivor down vs D7_non-survivor |
| MAOA | -2.01 | D0_non-survivor down vs D7_non-survivor |
| EPB42 | -1.97 | D0_non-survivor down vs D7_non-survivor |
| KLHDC4 | -1.95 | D0_non-survivor down vs D7_non-survivor |
| FLT3 | -1.94 | D0_non-survivor down vs D7_non-survivor |
| TMEM119 | -1.90 | D0_non-survivor down vs D7_non-survivor |
| LCN2 | -1.90 | D0_non-survivor down vs D7_non-survivor |
| CAMP | -1.90 | D0_non-survivor down vs D7_non-survivor |
| IGHG1 | -1.88 | D0_non-survivor down vs D7_non-survivor |
| KIAA0831 | -1.87 | D0_non-survivor down vs D7_non-survivor |
| ALDH1A1 | -1.86 | D0_non-survivor down vs D7_non-survivor |
| KRT1 | -1.85 | D0_non-survivor down vs D7_non-survivor |
| IRX3 | -1.84 | D0_non-survivor down vs D7_non-survivor |
| FAM78B | -1.84 | D0_non-survivor down vs D7_non-survivor |
| IL18RAP | -1.83 | D0_non-survivor down vs D7_non-survivor |
| ZBTB16 | -1.82 | D0_non-survivor down vs D7_non-survivor |
| TMEM45A | -1.81 | D0_non-survivor down vs D7_non-survivor |
| GRB10 | -1.81 | D0_non-survivor down vs D7_non-survivor |
| HBM | -1.81 | D0_non-survivor down vs D7_non-survivor |
| CD177 | -1.80 | D0_non-survivor down vs D7_non-survivor |
| HBA2 | -1.80 | D0_non-survivor down vs D7_non-survivor |
| ABCA13 | -1.79 | D0_non-survivor down vs D7_non-survivor |
| PFKFB2 | -1.79 | D0_non-survivor down vs D7_non-survivor |
| ECSM2 | -1.78 | D0_non-survivor down vs D7_non-survivor |
| FN1 | -1.78 | D0_non-survivor down vs D7_non-survivor |
| SDR-O | -1.78 | D0_non-survivor down vs D7_non-survivor |
| D83692 | -1.77 | D0_non-survivor down vs D7_non-survivor |
| IL18R1 | -1.76 | D0_non-survivor down vs D7_non-survivor |
| MDM4 | -1.76 | D0_non-survivor down vs D7_non-survivor |
| ERAF | -1.75 | D0_non-survivor down vs D7_non-survivor |
| IGHG1 | -1.74 | D0_non-survivor down vs D7_non-survivor |
| TPD52L3 | -1.74 | D0_non-survivor down vs D7_non-survivor |
| CEACAM1 | -1.74 | D0_non-survivor down vs D7_non-survivor |
| PAPOLB | -1.74 | D0_non-survivor down vs D7_non-survivor |
| OMG | -1.74 | D0_non-survivor down vs D7_non-survivor |
| AQP1 | -1.73 | D0_non-survivor down vs D7_non-survivor |
| HLF | -1.73 | D0_non-survivor down vs D7_non-survivor |
| DNAH5 | -1.72 | D0_non-survivor down vs D7_non-survivor |
| ECHDC3 | -1.72 | D0_non-survivor down vs D7_non-survivor |
| CNTNAP4 | -1.72 | D0_non-survivor down vs D7_non-survivor |
| KCNIP4 | -1.71 | D0_non-survivor down vs D7_non-survivor |
| IGJ | -1.71 | D0_non-survivor down vs D7_non-survivor |
| HSD17B13 | -1.71 | D0_non-survivor down vs D7_non-survivor |
| DKFZp547C195 | -1.71 | D0_non-survivor down vs D7_non-survivor |
| ZNF155 | -1.71 | D0_non-survivor down vs D7_non-survivor |
| RRAD | 1.71 | D0_non-survivor up vs D7_non-survivor |
| PMCHL1 | 1.71 | D0_non-survivor up vs D7_non-survivor |
| UBE2E1 | 1.71 | D0_non-survivor up vs D7_non-survivor |
| ABCA1 | 1.71 | D0_non-survivor up vs D7_non-survivor |
| PLAGL2 | 1.71 | D0_non-survivor up vs D7_non-survivor |
| PTX3 | 1.71 | D0_non-survivor up vs D7_non-survivor |
| PDGFRA | 1.71 | D0_non-survivor up vs D7_non-survivor |
| STXBP2 | 1.71 | D0_non-survivor up vs D7_non-survivor |
| KIAA1383 | 1.71 | D0_non-survivor up vs D7_non-survivor |
| RHOU | 1.71 | D0_non-survivor up vs D7_non-survivor |
| PHACTR1 | 1.71 | D0_non-survivor up vs D7_non-survivor |
| PTGER4 | 1.72 | D0_non-survivor up vs D7_non-survivor |
| HLA-DQA2 | 1.72 | D0_non-survivor up vs D7_non-survivor |
| P4HA3 | 1.72 | D0_non-survivor up vs D7_non-survivor |
| OLR1 | 1.72 | D0_non-survivor up vs D7_non-survivor |
| CCDC27 | 1.72 | D0_non-survivor up vs D7_non-survivor |
| OPCML | 1.73 | D0_non-survivor up vs D7_non-survivor |
| PIP5K2B | 1.73 | D0_non-survivor up vs D7_non-survivor |
| ATF3 | 1.73 | D0_non-survivor up vs D7_non-survivor |
| RBMY1B | 1.73 | D0_non-survivor up vs D7_non-survivor |
| PTGER4 | 1.74 | D0_non-survivor up vs D7_non-survivor |
| PKD2 | 1.74 | D0_non-survivor up vs D7_non-survivor |
| WDR76 | 1.74 | D0_non-survivor up vs D7_non-survivor |
| USP3 | 1.74 | D0_non-survivor up vs D7_non-survivor |
| B4GALT2 | 1.74 | D0_non-survivor up vs D7_non-survivor |
| MGC12916 | 1.75 | D0_non-survivor up vs D7_non-survivor |
| SLC2A6 | 1.75 | D0_non-survivor up vs D7_non-survivor |
| GBP1 | 1.76 | D0_non-survivor up vs D7_non-survivor |
| RHOU | 1.76 | D0_non-survivor up vs D7_non-survivor |
| SLC5A12 | 1.76 | D0_non-survivor up vs D7_non-survivor |
| USP22 | 1.77 | D0_non-survivor up vs D7_non-survivor |
| IFNB1 | 1.77 | D0_non-survivor up vs D7_non-survivor |
| PLP1 | 1.78 | D0_non-survivor up vs D7_non-survivor |
| TREM1 | 1.78 | D0_non-survivor up vs D7_non-survivor |
| PDE5A | 1.78 | D0_non-survivor up vs D7_non-survivor |
| INSIG1 | 1.79 | D0_non-survivor up vs D7_non-survivor |
| X98266 | 1.79 | D0_non-survivor up vs D7_non-survivor |
| LONRF1 | 1.79 | D0_non-survivor up vs D7_non-survivor |
| TNF | 1.79 | D0_non-survivor up vs D7_non-survivor |
| LHFPL2 | 1.80 | D0_non-survivor up vs D7_non-survivor |
| SLC16A6 | 1.80 | D0_non-survivor up vs D7_non-survivor |
| HERV-FRD | 1.80 | D0_non-survivor up vs D7_non-survivor |
| FER1L3 | 1.81 | D0_non-survivor up vs D7_non-survivor |
| CCR1 | 1.81 | D0_non-survivor up vs D7_non-survivor |
| CPEB2 | 1.81 | D0_non-survivor up vs D7_non-survivor |
| LONRF1 | 1.81 | D0_non-survivor up vs D7_non-survivor |
| FAM20A | 1.81 | D0_non-survivor up vs D7_non-survivor |
| WNT5A | 1.81 | D0_non-survivor up vs D7_non-survivor |
| CCL23 | 1.82 | D0_non-survivor up vs D7_non-survivor |
| SSTR2 | 1.82 | D0_non-survivor up vs D7_non-survivor |
| KLF5 | 1.82 | D0_non-survivor up vs D7_non-survivor |
| RABGEF1 | 1.83 | D0_non-survivor up vs D7_non-survivor |
| N4BP1 | 1.83 | D0_non-survivor up vs D7_non-survivor |
| B4GALT5 | 1.83 | D0_non-survivor up vs D7_non-survivor |
| HES1 | 1.83 | D0_non-survivor up vs D7_non-survivor |
| ZMYND17 | 1.84 | D0_non-survivor up vs D7_non-survivor |
| VPS13C | 1.84 | D0_non-survivor up vs D7_non-survivor |
| NFKB1 | 1.84 | D0_non-survivor up vs D7_non-survivor |
| U94902 | 1.84 | D0_non-survivor up vs D7_non-survivor |
| GPR88 | 1.85 | D0_non-survivor up vs D7_non-survivor |
| SNAI1 | 1.85 | D0_non-survivor up vs D7_non-survivor |
| ATP6V0A4 | 1.85 | D0_non-survivor up vs D7_non-survivor |
| TTLL7 | 1.85 | D0_non-survivor up vs D7_non-survivor |
| OLFM1 | 1.85 | D0_non-survivor up vs D7_non-survivor |
| RGC32 | 1.85 | D0_non-survivor up vs D7_non-survivor |
| U92981 | 1.85 | D0_non-survivor up vs D7_non-survivor |
| ZNF701 | 1.86 | D0_non-survivor up vs D7_non-survivor |
| EMR1 | 1.86 | D0_non-survivor up vs D7_non-survivor |
| RP11-130N24.1 | 1.86 | D0_non-survivor up vs D7_non-survivor |
| VPS13B | 1.86 | D0_non-survivor up vs D7_non-survivor |
| CD74 | 1.87 | D0_non-survivor up vs D7_non-survivor |
| NOTCH2NL | 1.87 | D0_non-survivor up vs D7_non-survivor |
| FGA | 1.88 | D0_non-survivor up vs D7_non-survivor |
| SLC35D3 | 1.89 | D0_non-survivor up vs D7_non-survivor |
| ADORA2A | 1.89 | D0_non-survivor up vs D7_non-survivor |
| ADAM6 | 1.89 | D0_non-survivor up vs D7_non-survivor |
| ITGA1 | 1.89 | D0_non-survivor up vs D7_non-survivor |
| EGR3 | 1.90 | D0_non-survivor up vs D7_non-survivor |
| BACH1 | 1.90 | D0_non-survivor up vs D7_non-survivor |
| SNFT | 1.90 | D0_non-survivor up vs D7_non-survivor |
| GDDR | 1.91 | D0_non-survivor up vs D7_non-survivor |
| MARCO | 1.91 | D0_non-survivor up vs D7_non-survivor |
| PHACTR1 | 1.91 | D0_non-survivor up vs D7_non-survivor |
| B4GALT5 | 1.91 | D0_non-survivor up vs D7_non-survivor |
| KCNA3 | 1.91 | D0_non-survivor up vs D7_non-survivor |
| RP6-213H19.2 | 1.92 | D0_non-survivor up vs D7_non-survivor |
| SMPDL3A | 1.92 | D0_non-survivor up vs D7_non-survivor |
| PELI1 | 1.92 | D0_non-survivor up vs D7_non-survivor |
| TMEM16F | 1.93 | D0_non-survivor up vs D7_non-survivor |
| SCML1 | 1.93 | D0_non-survivor up vs D7_non-survivor |
| DUOX1 | 1.93 | D0_non-survivor up vs D7_non-survivor |
| ARHGDIA | 1.94 | D0_non-survivor up vs D7_non-survivor |
| MSC | 1.94 | D0_non-survivor up vs D7_non-survivor |
| SAMSN1 | 1.95 | D0_non-survivor up vs D7_non-survivor |
| OAS3 | 1.95 | D0_non-survivor up vs D7_non-survivor |
| PTPN11 | 1.96 | D0_non-survivor up vs D7_non-survivor |
| ARL5B | 1.96 | D0_non-survivor up vs D7_non-survivor |
| FST | 1.97 | D0_non-survivor up vs D7_non-survivor |
| ATP6V1G3 | 1.97 | D0_non-survivor up vs D7_non-survivor |
| ANKRD22 | 1.97 | D0_non-survivor up vs D7_non-survivor |
| B3GALT5 | 1.97 | D0_non-survivor up vs D7_non-survivor |
| TRAF1 | 1.97 | D0_non-survivor up vs D7_non-survivor |
| CCL2 | 1.97 | D0_non-survivor up vs D7_non-survivor |
| YWHAE | 1.98 | D0_non-survivor up vs D7_non-survivor |
| LIMK2 | 1.99 | D0_non-survivor up vs D7_non-survivor |
| RASAL2 | 1.99 | D0_non-survivor up vs D7_non-survivor |
| NTRK2 | 1.99 | D0_non-survivor up vs D7_non-survivor |
| RAPGEF2 | 2.01 | D0_non-survivor up vs D7_non-survivor |
| CXCL2 | 2.01 | D0_non-survivor up vs D7_non-survivor |
| CSF3 | 2.01 | D0_non-survivor up vs D7_non-survivor |
| HIF3A | 2.02 | D0_non-survivor up vs D7_non-survivor |
| SMPDL3A | 2.05 | D0_non-survivor up vs D7_non-survivor |
| CXCL3 | 2.05 | D0_non-survivor up vs D7_non-survivor |
| IL23A | 2.06 | D0_non-survivor up vs D7_non-survivor |
| MAPK6 | 2.06 | D0_non-survivor up vs D7_non-survivor |
| TP53INP2 | 2.06 | D0_non-survivor up vs D7_non-survivor |
| CLEC5A | 2.07 | D0_non-survivor up vs D7_non-survivor |
| GJB2 | 2.08 | D0_non-survivor up vs D7_non-survivor |
| IBRDC3 | 2.09 | D0_non-survivor up vs D7_non-survivor |
| GBP1 | 2.10 | D0_non-survivor up vs D7_non-survivor |
| IFIT1 | 2.10 | D0_non-survivor up vs D7_non-survivor |
| ZNF697 | 2.11 | D0_non-survivor up vs D7_non-survivor |
| HNMT | 2.12 | D0_non-survivor up vs D7_non-survivor |
| DNAJB4 | 2.13 | D0_non-survivor up vs D7_non-survivor |
| ARL5B | 2.13 | D0_non-survivor up vs D7_non-survivor |
| RRAD | 2.13 | D0_non-survivor up vs D7_non-survivor |
| GJB2 | 2.13 | D0_non-survivor up vs D7_non-survivor |
| DMXL2 | 2.14 | D0_non-survivor up vs D7_non-survivor |
| RAPGEF2 | 2.14 | D0_non-survivor up vs D7_non-survivor |
| IFI44L | 2.16 | D0_non-survivor up vs D7_non-survivor |
| OASL | 2.17 | D0_non-survivor up vs D7_non-survivor |
| GPT2 | 2.17 | D0_non-survivor up vs D7_non-survivor |
| KL | 2.20 | D0_non-survivor up vs D7_non-survivor |
| RASGEF1B | 2.20 | D0_non-survivor up vs D7_non-survivor |
| KCNJ2 | 2.26 | D0_non-survivor up vs D7_non-survivor |
| SDC2 | 2.26 | D0_non-survivor up vs D7_non-survivor |
| IFIT3 | 2.27 | D0_non-survivor up vs D7_non-survivor |
| FLT1 | 2.27 | D0_non-survivor up vs D7_non-survivor |
| PLD1 | 2.28 | D0_non-survivor up vs D7_non-survivor |
| FAM20A | 2.28 | D0_non-survivor up vs D7_non-survivor |
| GPR84 | 2.28 | D0_non-survivor up vs D7_non-survivor |
| IL1RN | 2.34 | D0_non-survivor up vs D7_non-survivor |
| SIGLEC1 | 2.37 | D0_non-survivor up vs D7_non-survivor |
| CFB | 2.38 | D0_non-survivor up vs D7_non-survivor |
| DOCK4 | 2.39 | D0_non-survivor up vs D7_non-survivor |
| SAA1 | 2.42 | D0_non-survivor up vs D7_non-survivor |
| LAMB3 | 2.46 | D0_non-survivor up vs D7_non-survivor |
| CCRL2 | 2.46 | D0_non-survivor up vs D7_non-survivor |
| SLC39A8 | 2.47 | D0_non-survivor up vs D7_non-survivor |
| NUPL1 | 2.48 | D0_non-survivor up vs D7_non-survivor |
| ABCA1 | 2.53 | D0_non-survivor up vs D7_non-survivor |
| CCL23 | 2.54 | D0_non-survivor up vs D7_non-survivor |
| SERPINB9 | 2.54 | D0_non-survivor up vs D7_non-survivor |
| TFAP4 | 2.57 | D0_non-survivor up vs D7_non-survivor |
| CASC5 | 2.57 | D0_non-survivor up vs D7_non-survivor |
| CDH19 | 2.58 | D0_non-survivor up vs D7_non-survivor |
| RIN2 | 2.63 | D0_non-survivor up vs D7_non-survivor |
| CBLN2 | 2.63 | D0_non-survivor up vs D7_non-survivor |
| ATP2B1 | 2.68 | D0_non-survivor up vs D7_non-survivor |
| TNFAIP6 | 2.68 | D0_non-survivor up vs D7_non-survivor |
| RIN2 | 2.71 | D0_non-survivor up vs D7_non-survivor |
| ACVR2A | 2.72 | D0_non-survivor up vs D7_non-survivor |
| REN | 2.75 | D0_non-survivor up vs D7_non-survivor |
| CAMKV | 2.78 | D0_non-survivor up vs D7_non-survivor |
| ZNF517 | 2.91 | D0_non-survivor up vs D7_non-survivor |
| CCRL2 | 2.93 | D0_non-survivor up vs D7_non-survivor |
| KIAA1731 | 2.99 | D0_non-survivor up vs D7_non-survivor |
| H12329 | 2.99 | D0_non-survivor up vs D7_non-survivor |
| TMEM22 | 3.01 | D0_non-survivor up vs D7_non-survivor |
| TPD52L3 | 3.01 | D0_non-survivor up vs D7_non-survivor |
| IFIT2 | 3.10 | D0_non-survivor up vs D7_non-survivor |
| TNIP3 | 3.14 | D0_non-survivor up vs D7_non-survivor |
| ZNF17 | 3.45 | D0_non-survivor up vs D7_non-survivor |
| HES1 | 3.65 | D0_non-survivor up vs D7_non-survivor |
| IL6 | 3.88 | D0_non-survivor up vs D7_non-survivor |
| NEK1 | 3.89 | D0_non-survivor up vs D7_non-survivor |
| CXCL10 | 3.91 | D0_non-survivor up vs D7_non-survivor |
| RASAL2 | 4.09 | D0_non-survivor up vs D7_non-survivor |
| STEAP4 | 4.38 | D0_non-survivor up vs D7_non-survivor |
| IFIT2 | 4.55 | D0_non-survivor up vs D7_non-survivor |
| KIAA0492 | 5.11 | D0_non-survivor up vs D7_non-survivor |
| IFI27 | 9.05 | D0_non-survivor up vs D7_non-survivor |
| IFI27 | 9.98 | D0_non-survivor up vs D7_non-survivor |
| IL1A | 10.15 | D0_non-survivor up vs D7_non-survivor |
| CCL7 | 15.10 | D0_non-survivor up vs D7_non-survivor |
| SIGLEC1 | 97.01 | D0_non-survivor up vs D7_non-survivor |
