# Supplementary material for: Patterns of Gene Expression in Peripheral Blood Mononuclear Cells and Outcomes from Patients with Sepsis Secondary to Community Acquired Pneumonia
Source: PLoS One. 2014 Mar 25;9(3):e91886. doi: 10.1371/journal.pone.0091886 (PMC3965402; doi:10.1371/journal.pone.0091886)
Supplement: Table S5 — Differential gene expression between survivors at D7 and non-survivors at D7. Only genes presenting at least a 1.7 fold change and p value <0.05 are reported. (DOCX) [file pone.0091886.s005.docx]

**Table S5: Differential gene expression between survivors at D7 and non-survivors at D7. Only genes presenting at least a 1.7 fold change and p value < 0.05 are reported.**

| Gene  Symbol | p-value | FoldChange  Survivor_D7 vs. Nonsurvivor_D7 | FoldChange Description |
| --- | --- | --- | --- |
| MBNL3 | 4.90E-04 | -55.33 | Survivor down vs Non-survivor |
| POU3F1 | 4.82E-04 | -50.85 | Survivor down vs Non-survivor |
| NF1 | 1.41E-02 | -31.32 | Survivor down vs Non-survivor |
| NIPA1 | 4.52E-04 | -27.69 | Survivor down vs Non-survivor |
| BBS5 | 4.48E-04 | -23.44 | Survivor down vs Non-survivor |
| XKRX | 1.41E-02 | -16.29 | Survivor down vs Non-survivor |
| QRSL1 | 7.64E-04 | -16.26 | Survivor down vs Non-survivor |
| SENP6 | 2.32E-03 | -16.12 | Survivor down vs Non-survivor |
| AIPL1 | 8.18E-04 | -15.21 | Survivor down vs Non-survivor |
| CKMT2 | 5.28E-04 | -14.27 | Survivor down vs Non-survivor |
| CLDN8 | 7.92E-04 | -13.77 | Survivor down vs Non-survivor |
| TGFB3 | 1.51E-04 | -12.90 | Survivor down vs Non-survivor |
| AFF4 | 3.67E-04 | -12.31 | Survivor down vs Non-survivor |
| PCDHB5 | 4.00E-04 | -11.86 | Survivor down vs Non-survivor |
| WDR76 | 1.55E-02 | -11.57 | Survivor down vs Non-survivor |
| KLK13 | 3.92E-04 | -11.26 | Survivor down vs Non-survivor |
| POPDC2 | 5.94E-04 | -11.24 | Survivor down vs Non-survivor |
| NRG2 | 2.75E-03 | -10.35 | Survivor down vs Non-survivor |
| NALP13 | 3.02E-04 | -10.34 | Survivor down vs Non-survivor |
| IL2RA | 2.00E-03 | -10.21 | Survivor down vs Non-survivor |
| TRIM9 | 8.23E-05 | -9.31 | Survivor down vs Non-survivor |
| TM4SF18 | 4.10E-04 | -8.88 | Survivor down vs Non-survivor |
| RAPGEF4 | 1.67E-02 | -8.47 | Survivor down vs Non-survivor |
| PHLDB2 | 7.03E-03 | -8.36 | Survivor down vs Non-survivor |
| PNMA2 | 2.96E-04 | -7.97 | Survivor down vs Non-survivor |
| DACT1 | 1.61E-02 | -7.64 | Survivor down vs Non-survivor |
| PCDHGB2 | 1.66E-02 | -7.37 | Survivor down vs Non-survivor |
| WWTR1 | 3.70E-04 | -6.53 | Survivor down vs Non-survivor |
| STIM2 | 2.44E-04 | -6.39 | Survivor down vs Non-survivor |
| KLRC2 | 3.02E-02 | -6.38 | Survivor down vs Non-survivor |
| CRLF1 | 2.08E-03 | -6.32 | Survivor down vs Non-survivor |
| SCN3B | 2.18E-03 | -5.94 | Survivor down vs Non-survivor |
| BAIAP2 | 4.39E-04 | -5.90 | Survivor down vs Non-survivor |
| ATP1A2 | 1.09E-03 | -5.73 | Survivor down vs Non-survivor |
| CCDC66 | 1.22E-02 | -5.68 | Survivor down vs Non-survivor |
| GPR88 | 2.47E-02 | -5.66 | Survivor down vs Non-survivor |
| ZPBP2 | 3.58E-04 | -5.56 | Survivor down vs Non-survivor |
| SLC6A9 | 1.20E-03 | -5.53 | Survivor down vs Non-survivor |
| BMP4 | 4.54E-02 | -5.49 | Survivor down vs Non-survivor |
| PPEF2 | 1.16E-03 | -5.40 | Survivor down vs Non-survivor |
| PMCHL1 | 6.36E-03 | -5.35 | Survivor down vs Non-survivor |
| ADAMTS4 | 4.73E-03 | -5.33 | Survivor down vs Non-survivor |
| CLCN3 | 8.54E-03 | -5.32 | Survivor down vs Non-survivor |
| NAALAD2 | 1.05E-03 | -5.09 | Survivor down vs Non-survivor |
| GABRE | 1.09E-02 | -5.01 | Survivor down vs Non-survivor |
| SH2D1B | 1.13E-02 | -5.00 | Survivor down vs Non-survivor |
| CTNND1 | 1.36E-03 | -4.99 | Survivor down vs Non-survivor |
| TCEA3 | 2.79E-02 | -4.89 | Survivor down vs Non-survivor |
| MUCDHL | 2.96E-05 | -4.74 | Survivor down vs Non-survivor |
| RGPD2 | 4.92E-04 | -4.71 | Survivor down vs Non-survivor |
| AK5 | 1.38E-02 | -4.58 | Survivor down vs Non-survivor |
| CAPN12 | 1.63E-04 | -4.50 | Survivor down vs Non-survivor |
| TPD52L3 | 1.30E-02 | -4.45 | Survivor down vs Non-survivor |
| TSLP | 3.09E-04 | -4.43 | Survivor down vs Non-survivor |
| FLG | 3.26E-04 | -4.43 | Survivor down vs Non-survivor |
| FMO3 | 4.96E-03 | -4.42 | Survivor down vs Non-survivor |
| FILIP1 | 8.66E-03 | -4.40 | Survivor down vs Non-survivor |
| RNF157 | 1.18E-04 | -4.25 | Survivor down vs Non-survivor |
| RHOT1 | 2.49E-04 | -4.25 | Survivor down vs Non-survivor |
| GCC2 | 6.19E-04 | -4.23 | Survivor down vs Non-survivor |
| PLEKHH2 | 2.35E-03 | -4.20 | Survivor down vs Non-survivor |
| LGALS13 | 2.99E-02 | -4.06 | Survivor down vs Non-survivor |
| ODF3L1 | 2.95E-04 | -4.05 | Survivor down vs Non-survivor |
| SPRR4 | 1.85E-02 | -4.04 | Survivor down vs Non-survivor |
| LIFR | 1.02E-02 | -3.99 | Survivor down vs Non-survivor |
| FANK1 | 2.72E-04 | -3.99 | Survivor down vs Non-survivor |
| HYDIN | 1.96E-06 | -3.97 | Survivor down vs Non-survivor |
| SMG6 | 1.07E-03 | -3.78 | Survivor down vs Non-survivor |
| LZTS1 | 6.56E-03 | -3.64 | Survivor down vs Non-survivor |
| PF4V1 | 3.59E-02 | -3.62 | Survivor down vs Non-survivor |
| FGF7 | 3.18E-02 | -3.60 | Survivor down vs Non-survivor |
| LRRC43 | 1.75E-03 | -3.59 | Survivor down vs Non-survivor |
| PLEKHK1 | 1.89E-04 | -3.56 | Survivor down vs Non-survivor |
| PER3 | 1.25E-02 | -3.53 | Survivor down vs Non-survivor |
| TCF7 | 2.05E-03 | -3.50 | Survivor down vs Non-survivor |
| RREB1 | 1.16E-02 | -3.42 | Survivor down vs Non-survivor |
| KCNT1 | 9.29E-03 | -3.41 | Survivor down vs Non-survivor |
| PNPLA4 | 1.49E-02 | -3.39 | Survivor down vs Non-survivor |
| TSPYL6 | 4.31E-03 | -3.36 | Survivor down vs Non-survivor |
| HOXD10 | 2.54E-04 | -3.35 | Survivor down vs Non-survivor |
| MAL | 3.50E-03 | -3.29 | Survivor down vs Non-survivor |
| NRSN1 | 1.72E-02 | -3.28 | Survivor down vs Non-survivor |
| AXIN2 | 9.75E-03 | -3.27 | Survivor down vs Non-survivor |
| RGNEF | 1.03E-04 | -3.26 | Survivor down vs Non-survivor |
| GBX2 | 2.60E-03 | -3.26 | Survivor down vs Non-survivor |
| LRRN3 | 1.06E-04 | -3.25 | Survivor down vs Non-survivor |
| ANKS6 | 1.20E-04 | -3.23 | Survivor down vs Non-survivor |
| GPR44 | 4.87E-03 | -3.18 | Survivor down vs Non-survivor |
| EBF3 | 4.21E-03 | -3.16 | Survivor down vs Non-survivor |
| BRWD1 | 5.45E-03 | -3.14 | Survivor down vs Non-survivor |
| CDH5 | 7.16E-03 | -3.12 | Survivor down vs Non-survivor |
| ANKRD55 | 5.97E-03 | -3.10 | Survivor down vs Non-survivor |
| TMEPAI | 4.76E-02 | -3.09 | Survivor down vs Non-survivor |
| OSGEPL1 | 4.12E-03 | -3.08 | Survivor down vs Non-survivor |
| RASD1 | 5.35E-03 | -3.07 | Survivor down vs Non-survivor |
| NPAS1 | 1.35E-03 | -3.07 | Survivor down vs Non-survivor |
| LEF1 | 4.48E-03 | -3.06 | Survivor down vs Non-survivor |
| MDGA1 | 4.81E-02 | -3.05 | Survivor down vs Non-survivor |
| TMEM16A | 2.95E-03 | -3.04 | Survivor down vs Non-survivor |
| CENTB5 | 4.86E-04 | -3.03 | Survivor down vs Non-survivor |
| NEFL | 3.97E-03 | -3.02 | Survivor down vs Non-survivor |
| RAB15 | 5.64E-03 | -3.01 | Survivor down vs Non-survivor |
| SPAG8 | 2.65E-03 | -2.97 | Survivor down vs Non-survivor |
| CAPN14 | 2.24E-02 | -2.96 | Survivor down vs Non-survivor |
| CDCA2 | 3.41E-03 | -2.95 | Survivor down vs Non-survivor |
| ZNF613 | 1.96E-02 | -2.92 | Survivor down vs Non-survivor |
| CXADR | 3.71E-02 | -2.91 | Survivor down vs Non-survivor |
| AMDD | 5.32E-03 | -2.88 | Survivor down vs Non-survivor |
| FGA | 4.16E-03 | -2.85 | Survivor down vs Non-survivor |
| FAM90A1 | 1.74E-03 | -2.85 | Survivor down vs Non-survivor |
| GPSM2 | 3.36E-02 | -2.84 | Survivor down vs Non-survivor |
| BBS5 | 1.99E-02 | -2.81 | Survivor down vs Non-survivor |
| OR4N4 | 1.34E-03 | -2.81 | Survivor down vs Non-survivor |
| NAT11 | 3.22E-04 | -2.79 | Survivor down vs Non-survivor |
| NTRK2 | 5.63E-03 | -2.77 | Survivor down vs Non-survivor |
| IL17RB | 1.03E-03 | -2.75 | Survivor down vs Non-survivor |
| MAP2 | 3.39E-04 | -2.73 | Survivor down vs Non-survivor |
| FAM113B | 1.06E-03 | -2.73 | Survivor down vs Non-survivor |
| CCND2 | 2.49E-03 | -2.71 | Survivor down vs Non-survivor |
| MTNR1B | 1.86E-05 | -2.71 | Survivor down vs Non-survivor |
| CCR7 | 9.15E-03 | -2.70 | Survivor down vs Non-survivor |
| EFHA2 | 1.81E-02 | -2.69 | Survivor down vs Non-survivor |
| CENPA | 2.76E-03 | -2.69 | Survivor down vs Non-survivor |
| SYNJ2 | 1.86E-02 | -2.68 | Survivor down vs Non-survivor |
| CDH4 | 6.41E-03 | -2.67 | Survivor down vs Non-survivor |
| MTAP | 4.59E-03 | -2.67 | Survivor down vs Non-survivor |
| UGT2B10 | 2.48E-02 | -2.65 | Survivor down vs Non-survivor |
| TPTE | 7.77E-03 | -2.63 | Survivor down vs Non-survivor |
| DTX1 | 8.05E-03 | -2.63 | Survivor down vs Non-survivor |
| TTC28 | 2.06E-02 | -2.63 | Survivor down vs Non-survivor |
| RASSF8 | 8.42E-03 | -2.63 | Survivor down vs Non-survivor |
| BARHL2 | 5.86E-03 | -2.63 | Survivor down vs Non-survivor |
| CBLN1 | 1.63E-02 | -2.62 | Survivor down vs Non-survivor |
| NELL2 | 1.72E-02 | -2.62 | Survivor down vs Non-survivor |
| NCR1 | 5.27E-04 | -2.62 | Survivor down vs Non-survivor |
| FAM19A1 | 2.92E-03 | -2.57 | Survivor down vs Non-survivor |
| AGBL4 | 2.36E-04 | -2.56 | Survivor down vs Non-survivor |
| PA2G4 | 1.10E-02 | -2.55 | Survivor down vs Non-survivor |
| KIR2DS4 | 3.42E-02 | -2.55 | Survivor down vs Non-survivor |
| NCOR1 | 1.44E-02 | -2.55 | Survivor down vs Non-survivor |
| FAM90A1 | 2.17E-03 | -2.55 | Survivor down vs Non-survivor |
| NOX4 | 1.03E-02 | -2.54 | Survivor down vs Non-survivor |
| NR0B2 | 6.14E-03 | -2.53 | Survivor down vs Non-survivor |
| LRRN3 | 2.79E-04 | -2.53 | Survivor down vs Non-survivor |
| EDA2R | 1.04E-03 | -2.53 | Survivor down vs Non-survivor |
| UGT8 | 3.72E-08 | -2.52 | Survivor down vs Non-survivor |
| NXPH2 | 1.79E-03 | -2.52 | Survivor down vs Non-survivor |
| CST6 | 2.54E-07 | -2.49 | Survivor down vs Non-survivor |
| FA2H | 1.45E-02 | -2.48 | Survivor down vs Non-survivor |
| LTBP3 | 5.49E-03 | -2.48 | Survivor down vs Non-survivor |
| DNAI1 | 2.82E-04 | -2.47 | Survivor down vs Non-survivor |
| CTLA4 | 3.24E-04 | -2.46 | Survivor down vs Non-survivor |
| LOXHD1 | 3.54E-02 | -2.45 | Survivor down vs Non-survivor |
| EXOSC6 | 7.76E-04 | -2.45 | Survivor down vs Non-survivor |
| FHIT | 7.58E-03 | -2.45 | Survivor down vs Non-survivor |
| SNAI2 | 1.09E-04 | -2.44 | Survivor down vs Non-survivor |
| TBX5 | 4.98E-03 | -2.43 | Survivor down vs Non-survivor |
| WASL | 3.39E-04 | -2.43 | Survivor down vs Non-survivor |
| KCNK17 | 6.59E-03 | -2.43 | Survivor down vs Non-survivor |
| PTPRB | 1.29E-02 | -2.43 | Survivor down vs Non-survivor |
| PRG4 | 1.09E-03 | -2.41 | Survivor down vs Non-survivor |
| PKHD1 | 1.85E-02 | -2.41 | Survivor down vs Non-survivor |
| TOP2A | 2.93E-02 | -2.40 | Survivor down vs Non-survivor |
| USP9Y | 1.74E-02 | -2.39 | Survivor down vs Non-survivor |
| AKR1C3 | 4.54E-02 | -2.39 | Survivor down vs Non-survivor |
| PRM2 | 1.80E-02 | -2.39 | Survivor down vs Non-survivor |
| AP3M2 | 3.75E-02 | -2.38 | Survivor down vs Non-survivor |
| FCGBP | 2.31E-02 | -2.37 | Survivor down vs Non-survivor |
| ANKRD25 | 1.09E-03 | -2.37 | Survivor down vs Non-survivor |
| UBOX5 | 9.02E-04 | -2.36 | Survivor down vs Non-survivor |
| ANK3 | 1.08E-02 | -2.36 | Survivor down vs Non-survivor |
| PACSIN1 | 2.76E-02 | -2.34 | Survivor down vs Non-survivor |
| AEBP1 | 2.47E-02 | -2.34 | Survivor down vs Non-survivor |
| DNAJC14 | 1.55E-03 | -2.32 | Survivor down vs Non-survivor |
| NTN1 | 8.94E-03 | -2.31 | Survivor down vs Non-survivor |
| ICOSLG | 1.81E-02 | -2.31 | Survivor down vs Non-survivor |
| AGMAT | 2.94E-03 | -2.31 | Survivor down vs Non-survivor |
| TMEM135 | 1.51E-04 | -2.31 | Survivor down vs Non-survivor |
| UTY | 2.81E-02 | -2.30 | Survivor down vs Non-survivor |
| GOLGA8E | 7.78E-03 | -2.30 | Survivor down vs Non-survivor |
| RAB3A | 6.89E-05 | -2.29 | Survivor down vs Non-survivor |
| RBMY1E | 7.45E-03 | -2.28 | Survivor down vs Non-survivor |
| ARTS-1 | 8.25E-04 | -2.27 | Survivor down vs Non-survivor |
| RUTBC2 | 1.24E-02 | -2.27 | Survivor down vs Non-survivor |
| MYO1B | 1.13E-03 | -2.27 | Survivor down vs Non-survivor |
| IL2RA | 1.49E-02 | -2.26 | Survivor down vs Non-survivor |
| TNRC4 | 4.42E-02 | -2.24 | Survivor down vs Non-survivor |
| CSRP3 | 1.56E-02 | -2.24 | Survivor down vs Non-survivor |
| FAM92B | 1.26E-02 | -2.23 | Survivor down vs Non-survivor |
| RBM9 | 5.19E-03 | -2.22 | Survivor down vs Non-survivor |
| TMEM81 | 4.76E-03 | -2.21 | Survivor down vs Non-survivor |
| SPANXA1 | 8.79E-05 | -2.21 | Survivor down vs Non-survivor |
| IGKV1-5 | 9.17E-03 | -2.20 | Survivor down vs Non-survivor |
| TXK | 2.05E-02 | -2.20 | Survivor down vs Non-survivor |
| BCL11B | 2.03E-03 | -2.20 | Survivor down vs Non-survivor |
| CDH19 | 2.44E-02 | -2.19 | Survivor down vs Non-survivor |
| HDC | 3.63E-02 | -2.19 | Survivor down vs Non-survivor |
| TTTY12 | 6.16E-04 | -2.19 | Survivor down vs Non-survivor |
| WISP3 | 7.26E-05 | -2.19 | Survivor down vs Non-survivor |
| VWF | 1.15E-02 | -2.19 | Survivor down vs Non-survivor |
| ZIC5 | 2.11E-02 | -2.19 | Survivor down vs Non-survivor |
| HCG9 | 8.05E-04 | -2.19 | Survivor down vs Non-survivor |
| TMEFF2 | 7.33E-03 | -2.18 | Survivor down vs Non-survivor |
| SMPD3 | 2.71E-02 | -2.18 | Survivor down vs Non-survivor |
| IGHG1 | 3.71E-03 | -2.18 | Survivor down vs Non-survivor |
| FNDC4 | 7.47E-05 | -2.17 | Survivor down vs Non-survivor |
| NCAM1 | 9.28E-03 | -2.17 | Survivor down vs Non-survivor |
| HYDIN | 1.04E-02 | -2.17 | Survivor down vs Non-survivor |
| OPCML | 3.80E-03 | -2.17 | Survivor down vs Non-survivor |
| H2AFJ | 8.44E-03 | -2.16 | Survivor down vs Non-survivor |
| PRF1 | 4.43E-02 | -2.16 | Survivor down vs Non-survivor |
| TMEM25 | 2.45E-02 | -2.16 | Survivor down vs Non-survivor |
| SORCS3 | 1.80E-02 | -2.15 | Survivor down vs Non-survivor |
| JUB | 4.37E-03 | -2.14 | Survivor down vs Non-survivor |
| CRSP6 | 2.64E-02 | -2.14 | Survivor down vs Non-survivor |
| ATM | 1.46E-02 | -2.13 | Survivor down vs Non-survivor |
| EPHX2 | 1.14E-02 | -2.12 | Survivor down vs Non-survivor |
| SLC2A2 | 1.22E-03 | -2.12 | Survivor down vs Non-survivor |
| HYAL4 | 1.14E-02 | -2.12 | Survivor down vs Non-survivor |
| AMID | 1.01E-02 | -2.12 | Survivor down vs Non-survivor |
| KIR2DS2 | 8.18E-03 | -2.11 | Survivor down vs Non-survivor |
| RPL23AP13 | 9.07E-03 | -2.10 | Survivor down vs Non-survivor |
| FRK | 1.15E-03 | -2.10 | Survivor down vs Non-survivor |
| HTR6 | 1.07E-03 | -2.10 | Survivor down vs Non-survivor |
| ARHGEF10 | 4.59E-03 | -2.09 | Survivor down vs Non-survivor |
| ODF2L | 4.41E-03 | -2.09 | Survivor down vs Non-survivor |
| CAV1 | 3.92E-03 | -2.09 | Survivor down vs Non-survivor |
| FGF5 | 2.57E-02 | -2.08 | Survivor down vs Non-survivor |
| FOXP2 | 2.71E-02 | -2.08 | Survivor down vs Non-survivor |
| DOCK9 | 9.32E-03 | -2.08 | Survivor down vs Non-survivor |
| KIF9 | 2.69E-02 | -2.08 | Survivor down vs Non-survivor |
| EIF5A2 | 1.36E-03 | -2.07 | Survivor down vs Non-survivor |
| DPP4 | 2.83E-02 | -2.07 | Survivor down vs Non-survivor |
| SSPO | 7.56E-03 | -2.07 | Survivor down vs Non-survivor |
| TMOD4 | 1.39E-03 | -2.07 | Survivor down vs Non-survivor |
| LGALS8 | 4.14E-02 | -2.07 | Survivor down vs Non-survivor |
| DAZL | 3.77E-02 | -2.06 | Survivor down vs Non-survivor |
| LAMC2 | 3.78E-02 | -2.06 | Survivor down vs Non-survivor |
| FCRL5 | 3.09E-02 | -2.06 | Survivor down vs Non-survivor |
| FGF7 | 8.43E-03 | -2.06 | Survivor down vs Non-survivor |
| PASK | 3.77E-02 | -2.06 | Survivor down vs Non-survivor |
| IGHG1 | 3.29E-02 | -2.06 | Survivor down vs Non-survivor |
| NALP7 | 1.34E-02 | -2.05 | Survivor down vs Non-survivor |
| OAZ3 | 7.44E-03 | -2.05 | Survivor down vs Non-survivor |
| MPZL1 | 2.14E-02 | -2.05 | Survivor down vs Non-survivor |
| PYHIN1 | 1.22E-03 | -2.05 | Survivor down vs Non-survivor |
| ITK | 4.36E-02 | -2.04 | Survivor down vs Non-survivor |
| SCARNA17 | 1.99E-04 | -2.04 | Survivor down vs Non-survivor |
| GSG2 | 8.95E-04 | -2.04 | Survivor down vs Non-survivor |
| PLEKHG4 | 3.87E-02 | -2.04 | Survivor down vs Non-survivor |
| KIR2DL2 | 6.12E-03 | -2.03 | Survivor down vs Non-survivor |
| PLGLB2 | 3.87E-02 | -2.03 | Survivor down vs Non-survivor |
| VAMP1 | 2.59E-03 | -2.03 | Survivor down vs Non-survivor |
| FGFR1 | 2.69E-02 | -2.03 | Survivor down vs Non-survivor |
| DCTN1 | 4.91E-04 | -2.03 | Survivor down vs Non-survivor |
| LEPREL2 | 6.63E-03 | -2.03 | Survivor down vs Non-survivor |
| AUTS2 | 4.67E-02 | -2.03 | Survivor down vs Non-survivor |
| NDFIP2 | 6.19E-03 | -2.02 | Survivor down vs Non-survivor |
| CEP68 | 2.04E-02 | -2.02 | Survivor down vs Non-survivor |
| TEKT2 | 8.96E-05 | -2.01 | Survivor down vs Non-survivor |
| UBASH3A | 2.70E-02 | -2.01 | Survivor down vs Non-survivor |
| PARP3 | 2.41E-03 | -2.01 | Survivor down vs Non-survivor |
| KLHDC4 | 2.78E-02 | -2.00 | Survivor down vs Non-survivor |
| WDR65 | 1.60E-04 | -1.99 | Survivor down vs Non-survivor |
| SCAP1 | 3.00E-02 | -1.99 | Survivor down vs Non-survivor |
| AMOT | 1.99E-03 | -1.99 | Survivor down vs Non-survivor |
| NR3C2 | 3.50E-02 | -1.98 | Survivor down vs Non-survivor |
| THRSP | 2.09E-02 | -1.98 | Survivor down vs Non-survivor |
| GLCCI1 | 4.34E-02 | -1.98 | Survivor down vs Non-survivor |
| PDE7A | 3.87E-02 | -1.98 | Survivor down vs Non-survivor |
| CKS1B | 2.40E-02 | -1.98 | Survivor down vs Non-survivor |
| SHH | 1.04E-03 | -1.98 | Survivor down vs Non-survivor |
| ZBTB10 | 1.96E-02 | -1.98 | Survivor down vs Non-survivor |
| BCL2 | 3.63E-02 | -1.98 | Survivor down vs Non-survivor |
| BPIL2 | 4.91E-03 | -1.98 | Survivor down vs Non-survivor |
| COL25A1 | 9.63E-04 | -1.98 | Survivor down vs Non-survivor |
| DMRT3 | 1.09E-02 | -1.97 | Survivor down vs Non-survivor |
| KIR2DL5A | 7.32E-03 | -1.97 | Survivor down vs Non-survivor |
| SLC10A4 | 9.85E-03 | -1.97 | Survivor down vs Non-survivor |
| GRID1 | 1.62E-03 | -1.96 | Survivor down vs Non-survivor |
| NCR3 | 1.42E-02 | -1.96 | Survivor down vs Non-survivor |
| SNTG2 | 3.30E-03 | -1.96 | Survivor down vs Non-survivor |
| PPP1R9A | 2.81E-02 | -1.96 | Survivor down vs Non-survivor |
| KRT18 | 4.84E-02 | -1.96 | Survivor down vs Non-survivor |
| RGPD5 | 5.17E-04 | -1.96 | Survivor down vs Non-survivor |
| SULT4A1 | 2.03E-03 | -1.95 | Survivor down vs Non-survivor |
| KIF5C | 4.64E-02 | -1.95 | Survivor down vs Non-survivor |
| UGT2B28 | 5.84E-03 | -1.95 | Survivor down vs Non-survivor |
| ACOT4 | 1.63E-02 | -1.94 | Survivor down vs Non-survivor |
| CEP68 | 6.22E-03 | -1.94 | Survivor down vs Non-survivor |
| PPAT | 4.03E-02 | -1.94 | Survivor down vs Non-survivor |
| ASPM | 2.43E-02 | -1.94 | Survivor down vs Non-survivor |
| IGSF4 | 6.93E-03 | -1.94 | Survivor down vs Non-survivor |
| TNFRSF11A | 4.31E-03 | -1.94 | Survivor down vs Non-survivor |
| MDH1B | 2.28E-03 | -1.94 | Survivor down vs Non-survivor |
| SERPINB12 | 7.20E-04 | -1.93 | Survivor down vs Non-survivor |
| PCDHA9 | 3.00E-02 | -1.93 | Survivor down vs Non-survivor |
| SAA3P | 5.02E-02 | -1.93 | Survivor down vs Non-survivor |
| ENPP6 | 8.74E-04 | -1.93 | Survivor down vs Non-survivor |
| CCR3 | 4.11E-02 | -1.93 | Survivor down vs Non-survivor |
| KIR3DL2 | 3.17E-02 | -1.92 | Survivor down vs Non-survivor |
| GNAS | 1.98E-03 | -1.92 | Survivor down vs Non-survivor |
| COL4A6 | 1.04E-02 | -1.92 | Survivor down vs Non-survivor |
| RAI2 | 4.46E-03 | -1.91 | Survivor down vs Non-survivor |
| SMYD3 | 2.13E-02 | -1.91 | Survivor down vs Non-survivor |
| SRCAP | 3.02E-03 | -1.91 | Survivor down vs Non-survivor |
| TBC1D19 | 1.02E-02 | -1.91 | Survivor down vs Non-survivor |
| SNTB1 | 7.41E-03 | -1.91 | Survivor down vs Non-survivor |
| REST | 1.31E-02 | -1.91 | Survivor down vs Non-survivor |
| FATE1 | 2.37E-03 | -1.91 | Survivor down vs Non-survivor |
| SH3PXD2A | 2.37E-03 | -1.91 | Survivor down vs Non-survivor |
| UBE2C | 1.20E-02 | -1.90 | Survivor down vs Non-survivor |
| RAET1E | 1.92E-03 | -1.90 | Survivor down vs Non-survivor |
| ACTL7A | 1.11E-03 | -1.90 | Survivor down vs Non-survivor |
| TRPM3 | 4.25E-02 | -1.90 | Survivor down vs Non-survivor |
| ERVWE1 | 3.98E-02 | -1.90 | Survivor down vs Non-survivor |
| FGD5 | 2.91E-02 | -1.90 | Survivor down vs Non-survivor |
| PLXNB3 | 2.05E-03 | -1.89 | Survivor down vs Non-survivor |
| AP1GBP1 | 9.43E-03 | -1.89 | Survivor down vs Non-survivor |
| TRIB2 | 4.49E-02 | -1.89 | Survivor down vs Non-survivor |
| GOLGA | 9.67E-04 | -1.89 | Survivor down vs Non-survivor |
| SLFNL1 | 4.89E-02 | -1.88 | Survivor down vs Non-survivor |
| DMRTC1 | 2.09E-02 | -1.88 | Survivor down vs Non-survivor |
| OTUD3 | 2.23E-02 | -1.88 | Survivor down vs Non-survivor |
| BBS7 | 4.49E-02 | -1.88 | Survivor down vs Non-survivor |
| BNC2 | 3.08E-02 | -1.88 | Survivor down vs Non-survivor |
| HDHD3 | 4.13E-03 | -1.87 | Survivor down vs Non-survivor |
| ANGPTL4 | 4.95E-03 | -1.87 | Survivor down vs Non-survivor |
| NPCDR1 | 2.12E-03 | -1.87 | Survivor down vs Non-survivor |
| ORAOV1 | 9.17E-03 | -1.87 | Survivor down vs Non-survivor |
| LMCD1 | 9.43E-04 | -1.87 | Survivor down vs Non-survivor |
| TULP3 | 1.59E-03 | -1.87 | Survivor down vs Non-survivor |
| EVL | 2.86E-02 | -1.87 | Survivor down vs Non-survivor |
| TRPC1 | 3.79E-03 | -1.87 | Survivor down vs Non-survivor |
| GPRASP1 | 4.82E-02 | -1.87 | Survivor down vs Non-survivor |
| CCL11 | 1.50E-02 | -1.87 | Survivor down vs Non-survivor |
| GOLGA8A | 1.90E-02 | -1.87 | Survivor down vs Non-survivor |
| ITGBL1 | 3.70E-02 | -1.86 | Survivor down vs Non-survivor |
| SUNC1 | 1.44E-02 | -1.86 | Survivor down vs Non-survivor |
| ARL4C | 4.69E-02 | -1.86 | Survivor down vs Non-survivor |
| OCIAD2 | 2.92E-03 | -1.86 | Survivor down vs Non-survivor |
| EID3 | 4.23E-02 | -1.86 | Survivor down vs Non-survivor |
| SLC6A20 | 6.13E-03 | -1.85 | Survivor down vs Non-survivor |
| SH2D3A | 3.31E-04 | -1.85 | Survivor down vs Non-survivor |
| SATB1 | 6.20E-04 | -1.85 | Survivor down vs Non-survivor |
| RBMS3 | 1.05E-02 | -1.85 | Survivor down vs Non-survivor |
| FAM83C | 8.81E-03 | -1.85 | Survivor down vs Non-survivor |
| BCAS1 | 7.61E-03 | -1.85 | Survivor down vs Non-survivor |
| NME5 | 1.22E-02 | -1.85 | Survivor down vs Non-survivor |
| GRAP | 1.00E-03 | -1.85 | Survivor down vs Non-survivor |
| GCAT | 4.19E-02 | -1.84 | Survivor down vs Non-survivor |
| PTGIS | 1.48E-02 | -1.84 | Survivor down vs Non-survivor |
| RYR3 | 5.77E-04 | -1.84 | Survivor down vs Non-survivor |
| ARHGEF5 | 7.34E-03 | -1.84 | Survivor down vs Non-survivor |
| PLD3 | 2.00E-03 | -1.84 | Survivor down vs Non-survivor |
| GATS | 2.81E-04 | -1.84 | Survivor down vs Non-survivor |
| ASPHD1 | 1.34E-02 | -1.84 | Survivor down vs Non-survivor |
| TEP1 | 2.01E-02 | -1.84 | Survivor down vs Non-survivor |
| SLC26A1 | 3.86E-02 | -1.84 | Survivor down vs Non-survivor |
| PRO2900 | 2.14E-02 | -1.84 | Survivor down vs Non-survivor |
| CACNG2 | 3.02E-03 | -1.84 | Survivor down vs Non-survivor |
| NAT11 | 6.06E-03 | -1.83 | Survivor down vs Non-survivor |
| OTOA | 1.49E-02 | -1.83 | Survivor down vs Non-survivor |
| BCAR3 | 8.63E-06 | -1.83 | Survivor down vs Non-survivor |
| OR3A2 | 3.24E-02 | -1.83 | Survivor down vs Non-survivor |
| TMC8 | 7.55E-03 | -1.83 | Survivor down vs Non-survivor |
| UHG | 9.12E-03 | -1.83 | Survivor down vs Non-survivor |
| STMN3 | 2.93E-03 | -1.83 | Survivor down vs Non-survivor |
| ULBP2 | 3.66E-02 | -1.83 | Survivor down vs Non-survivor |
| C1QL2 | 4.21E-02 | -1.83 | Survivor down vs Non-survivor |
| THEM4 | 3.47E-02 | -1.83 | Survivor down vs Non-survivor |
| ZNF248 | 4.70E-02 | -1.82 | Survivor down vs Non-survivor |
| WT1 | 1.71E-02 | -1.82 | Survivor down vs Non-survivor |
| MTHFD1L | 9.86E-04 | -1.82 | Survivor down vs Non-survivor |
| CIT | 3.48E-02 | -1.82 | Survivor down vs Non-survivor |
| MORC4 | 2.81E-03 | -1.82 | Survivor down vs Non-survivor |
| GOLGA8E | 5.86E-03 | -1.82 | Survivor down vs Non-survivor |
| CNTNAP2 | 4.89E-02 | -1.82 | Survivor down vs Non-survivor |
| ZNF449 | 3.85E-02 | -1.81 | Survivor down vs Non-survivor |
| SPOCK2 | 3.73E-02 | -1.81 | Survivor down vs Non-survivor |
| GOLGA8B | 6.67E-03 | -1.81 | Survivor down vs Non-survivor |
| SHANK1 | 2.89E-02 | -1.81 | Survivor down vs Non-survivor |
| RPUSD2 | 2.55E-03 | -1.80 | Survivor down vs Non-survivor |
| TMEM30B | 1.45E-03 | -1.80 | Survivor down vs Non-survivor |
| POU5F1 | 1.58E-02 | -1.80 | Survivor down vs Non-survivor |
| GCET2 | 2.01E-02 | -1.80 | Survivor down vs Non-survivor |
| OTEX | 1.30E-02 | -1.80 | Survivor down vs Non-survivor |
| AQP7 | 2.96E-02 | -1.80 | Survivor down vs Non-survivor |
| SMYD4 | 1.93E-03 | -1.80 | Survivor down vs Non-survivor |
| SFI1 | 3.78E-03 | -1.80 | Survivor down vs Non-survivor |
| GC | 4.96E-02 | -1.80 | Survivor down vs Non-survivor |
| KRTAP9-4 | 3.45E-03 | -1.80 | Survivor down vs Non-survivor |
| PVRL3 | 4.77E-04 | -1.80 | Survivor down vs Non-survivor |
| RNF126 | 1.86E-02 | -1.80 | Survivor down vs Non-survivor |
| DZIP1L | 3.67E-04 | -1.80 | Survivor down vs Non-survivor |
| HCG18 | 2.04E-02 | -1.79 | Survivor down vs Non-survivor |
| KY | 2.94E-03 | -1.79 | Survivor down vs Non-survivor |
| ZNF680 | 1.61E-02 | -1.79 | Survivor down vs Non-survivor |
| CYFIP2 | 1.49E-03 | -1.79 | Survivor down vs Non-survivor |
| CAD | 1.45E-03 | -1.78 | Survivor down vs Non-survivor |
| TNFSF15 | 1.03E-02 | -1.78 | Survivor down vs Non-survivor |
| SNORD22 | 1.17E-02 | -1.78 | Survivor down vs Non-survivor |
| RGS4 | 1.84E-04 | -1.78 | Survivor down vs Non-survivor |
| CXXC4 | 3.12E-02 | -1.78 | Survivor down vs Non-survivor |
| EVC | 1.96E-04 | -1.78 | Survivor down vs Non-survivor |
| ITGB1BP2 | 4.18E-02 | -1.78 | Survivor down vs Non-survivor |
| EDA | 1.67E-03 | -1.78 | Survivor down vs Non-survivor |
| PLCH2 | 4.19E-02 | -1.77 | Survivor down vs Non-survivor |
| EVI1 | 4.80E-04 | -1.77 | Survivor down vs Non-survivor |
| VIT | 1.02E-03 | -1.77 | Survivor down vs Non-survivor |
| NANP | 1.32E-02 | -1.77 | Survivor down vs Non-survivor |
| RGS4 | 3.97E-02 | -1.77 | Survivor down vs Non-survivor |
| SPTBN1 | 3.09E-02 | -1.77 | Survivor down vs Non-survivor |
| ZNF101 | 2.46E-03 | -1.77 | Survivor down vs Non-survivor |
| ZNF342 | 2.93E-03 | -1.77 | Survivor down vs Non-survivor |
| FAM100B | 4.16E-04 | -1.76 | Survivor down vs Non-survivor |
| CNGA4 | 3.43E-02 | -1.76 | Survivor down vs Non-survivor |
| GOLGA2LY1 | 2.34E-02 | -1.76 | Survivor down vs Non-survivor |
| SPIN3 | 8.74E-03 | -1.76 | Survivor down vs Non-survivor |
| FAM71B | 4.81E-04 | -1.76 | Survivor down vs Non-survivor |
| ZNF468 | 1.83E-02 | -1.76 | Survivor down vs Non-survivor |
| BEX2 | 2.38E-02 | -1.76 | Survivor down vs Non-survivor |
| TNNI3K | 4.38E-03 | -1.76 | Survivor down vs Non-survivor |
| MS4A8B | 5.83E-03 | -1.76 | Survivor down vs Non-survivor |
| NFKBIL2 | 3.37E-02 | -1.76 | Survivor down vs Non-survivor |
| PHKA1 | 2.16E-02 | -1.75 | Survivor down vs Non-survivor |
| ALS2CR11 | 1.19E-02 | -1.75 | Survivor down vs Non-survivor |
| KRT2 | 1.93E-02 | -1.75 | Survivor down vs Non-survivor |
| VSX1 | 2.08E-02 | -1.75 | Survivor down vs Non-survivor |
| G36631 | 2.27E-04 | -1.75 | Survivor down vs Non-survivor |
| TSPYL1 | 5.27E-04 | -1.75 | Survivor down vs Non-survivor |
| HS6ST2 | 1.75E-02 | -1.75 | Survivor down vs Non-survivor |
| SNPH | 2.05E-03 | -1.75 | Survivor down vs Non-survivor |
| ST3GAL4 | 2.30E-02 | -1.75 | Survivor down vs Non-survivor |
| FZD3 | 5.16E-03 | -1.75 | Survivor down vs Non-survivor |
| BAIAP2L2 | 4.35E-02 | -1.74 | Survivor down vs Non-survivor |
| KCNJ1 | 9.19E-04 | -1.74 | Survivor down vs Non-survivor |
| FBXL16 | 3.39E-02 | -1.74 | Survivor down vs Non-survivor |
| GSG1 | 3.15E-02 | -1.74 | Survivor down vs Non-survivor |
| HIST3H2BB | 7.92E-04 | -1.74 | Survivor down vs Non-survivor |
| PLEKHB1 | 2.08E-02 | -1.74 | Survivor down vs Non-survivor |
| ELSPBP1 | 7.38E-03 | -1.74 | Survivor down vs Non-survivor |
| NET1 | 2.15E-02 | -1.73 | Survivor down vs Non-survivor |
| EN1 | 6.40E-03 | -1.73 | Survivor down vs Non-survivor |
| POLR3E | 4.52E-02 | -1.73 | Survivor down vs Non-survivor |
| CKS1B | 5.84E-03 | -1.73 | Survivor down vs Non-survivor |
| GPR150 | 3.19E-03 | -1.73 | Survivor down vs Non-survivor |
| PLEKHA1 | 3.58E-02 | -1.73 | Survivor down vs Non-survivor |
| MLLT6 | 1.33E-02 | -1.73 | Survivor down vs Non-survivor |
| CCDC36 | 3.11E-02 | -1.73 | Survivor down vs Non-survivor |
| AP3B2 | 4.27E-02 | -1.73 | Survivor down vs Non-survivor |
| CYP26B1 | 4.69E-02 | -1.72 | Survivor down vs Non-survivor |
| TNFRSF7 | 7.84E-03 | -1.72 | Survivor down vs Non-survivor |
| ZNF708 | 3.49E-04 | -1.72 | Survivor down vs Non-survivor |
| SRGAP2 | 1.20E-02 | -1.72 | Survivor down vs Non-survivor |
| CHD7 | 3.03E-02 | -1.72 | Survivor down vs Non-survivor |
| CEP72 | 6.44E-05 | -1.72 | Survivor down vs Non-survivor |
| ABLIM1 | 7.48E-03 | -1.72 | Survivor down vs Non-survivor |
| ASNS | 1.57E-02 | -1.72 | Survivor down vs Non-survivor |
| RIMS2 | 2.11E-02 | -1.72 | Survivor down vs Non-survivor |
| CCDC34 | 1.29E-02 | -1.71 | Survivor down vs Non-survivor |
| TBX4 | 3.51E-02 | -1.71 | Survivor down vs Non-survivor |
| HSPA4L | 6.13E-04 | -1.71 | Survivor down vs Non-survivor |
| SCUBE1 | 4.66E-03 | -1.71 | Survivor down vs Non-survivor |
| NUBP2 | 1.33E-02 | -1.71 | Survivor down vs Non-survivor |
| ABCB9 | 3.27E-02 | -1.71 | Survivor down vs Non-survivor |
| CCNB2 | 7.32E-03 | -1.71 | Survivor down vs Non-survivor |
| EPHA3 | 7.30E-03 | -1.71 | Survivor down vs Non-survivor |
| FLT3LG | 4.84E-03 | -1.70 | Survivor down vs Non-survivor |
| XPO6 | 2.34E-02 | -1.70 | Survivor down vs Non-survivor |
| VGLL2 | 2.56E-02 | -1.70 | Survivor down vs Non-survivor |
| SMYD1 | 1.43E-02 | -1.70 | Survivor down vs Non-survivor |
| STMN1 | 4.56E-02 | -1.70 | Survivor down vs Non-survivor |
| CYLC2 | 4.56E-02 | -1.70 | Survivor down vs Non-survivor |
| TTC12 | 2.23E-02 | -1.70 | Survivor down vs Non-survivor |
| SRPX | 4.85E-03 | -1.70 | Survivor down vs Non-survivor |
| DSCR1L2 | 2.46E-02 | -1.70 | Survivor down vs Non-survivor |
| HIST1H1B | 2.03E-02 | -1.70 | Survivor down vs Non-survivor |
| LAP3 | 3.31E-02 | 1.70 | Survivor up vs Non-survivor |
| HOXA1 | 3.17E-02 | 1.70 | Survivor up vs Non-survivor |
| CD86 | 6.08E-04 | 1.70 | Survivor up vs Non-survivor |
| USP3 | 1.77E-02 | 1.71 | Survivor up vs Non-survivor |
| PNPLA8 | 3.70E-02 | 1.71 | Survivor up vs Non-survivor |
| HPSE | 2.00E-02 | 1.71 | Survivor up vs Non-survivor |
| RAB1A | 1.09E-02 | 1.71 | Survivor up vs Non-survivor |
| PHF5A | 9.09E-03 | 1.71 | Survivor up vs Non-survivor |
| HLA-DRB1 | 6.31E-03 | 1.71 | Survivor up vs Non-survivor |
| CCL8 | 1.32E-02 | 1.71 | Survivor up vs Non-survivor |
| TLR1 | 2.97E-02 | 1.71 | Survivor up vs Non-survivor |
| CR590180 | 1.22E-02 | 1.71 | Survivor up vs Non-survivor |
| SEZ6L2 | 3.46E-02 | 1.72 | Survivor up vs Non-survivor |
| PACSIN2 | 2.63E-03 | 1.72 | Survivor up vs Non-survivor |
| TMSB4Y | 2.90E-02 | 1.72 | Survivor up vs Non-survivor |
| HLA-DRB3 | 1.89E-02 | 1.72 | Survivor up vs Non-survivor |
| ZNF644 | 3.84E-02 | 1.72 | Survivor up vs Non-survivor |
| NPNT | 2.55E-02 | 1.72 | Survivor up vs Non-survivor |
| FADD | 2.42E-03 | 1.72 | Survivor up vs Non-survivor |
| HLA-DMA | 8.08E-03 | 1.72 | Survivor up vs Non-survivor |
| KIF3C | 2.06E-02 | 1.72 | Survivor up vs Non-survivor |
| TLR4 | 5.49E-04 | 1.73 | Survivor up vs Non-survivor |
| AGPAT2 | 2.35E-02 | 1.73 | Survivor up vs Non-survivor |
| PHCA | 4.18E-03 | 1.73 | Survivor up vs Non-survivor |
| AFF4 | 3.26E-02 | 1.73 | Survivor up vs Non-survivor |
| TOM1L1 | 1.84E-02 | 1.73 | Survivor up vs Non-survivor |
| IFI44 | 4.31E-02 | 1.74 | Survivor up vs Non-survivor |
| TSC22D2 | 2.41E-02 | 1.74 | Survivor up vs Non-survivor |
| ST3GAL6 | 3.79E-03 | 1.74 | Survivor up vs Non-survivor |
| TCF7L1 | 2.11E-03 | 1.74 | Survivor up vs Non-survivor |
| NBN | 4.03E-02 | 1.74 | Survivor up vs Non-survivor |
| NDUFA5 | 8.87E-03 | 1.74 | Survivor up vs Non-survivor |
| SIAH2 | 2.10E-02 | 1.75 | Survivor up vs Non-survivor |
| LRRK2 | 1.25E-02 | 1.75 | Survivor up vs Non-survivor |
| ITSN1 | 8.59E-03 | 1.75 | Survivor up vs Non-survivor |
| TAS2R50 | 8.88E-03 | 1.76 | Survivor up vs Non-survivor |
| FOXO3A | 9.30E-03 | 1.76 | Survivor up vs Non-survivor |
| ACSS2 | 4.45E-02 | 1.76 | Survivor up vs Non-survivor |
| WNT5A | 5.69E-03 | 1.76 | Survivor up vs Non-survivor |
| CTSS | 6.29E-03 | 1.76 | Survivor up vs Non-survivor |
| MICALCL | 1.69E-02 | 1.76 | Survivor up vs Non-survivor |
| CLC | 1.86E-02 | 1.77 | Survivor up vs Non-survivor |
| ADCK1 | 3.87E-02 | 1.77 | Survivor up vs Non-survivor |
| ZNF383 | 2.98E-02 | 1.77 | Survivor up vs Non-survivor |
| PPM2C | 1.87E-02 | 1.77 | Survivor up vs Non-survivor |
| BTG2 | 8.48E-03 | 1.77 | Survivor up vs Non-survivor |
| BET1 | 3.11E-02 | 1.77 | Survivor up vs Non-survivor |
| CA2 | 4.09E-02 | 1.77 | Survivor up vs Non-survivor |
| PPGB | 2.27E-02 | 1.77 | Survivor up vs Non-survivor |
| MYOC | 4.54E-02 | 1.77 | Survivor up vs Non-survivor |
| PRKAG1 | 3.25E-03 | 1.77 | Survivor up vs Non-survivor |
| PLEKHA2 | 2.27E-02 | 1.78 | Survivor up vs Non-survivor |
| KYNU | 1.59E-02 | 1.78 | Survivor up vs Non-survivor |
| GORASP1 | 1.79E-02 | 1.78 | Survivor up vs Non-survivor |
| TEX13A | 3.39E-02 | 1.78 | Survivor up vs Non-survivor |
| CLEC2L | 2.40E-02 | 1.78 | Survivor up vs Non-survivor |
| OLIG1 | 5.08E-02 | 1.79 | Survivor up vs Non-survivor |
| LEPROT | 2.62E-02 | 1.79 | Survivor up vs Non-survivor |
| RAB6IP2 | 1.84E-02 | 1.79 | Survivor up vs Non-survivor |
| TRA2A | 4.22E-03 | 1.79 | Survivor up vs Non-survivor |
| GDNF | 3.98E-02 | 1.80 | Survivor up vs Non-survivor |
| S100A11 | 1.05E-04 | 1.80 | Survivor up vs Non-survivor |
| FOXRED1 | 4.20E-02 | 1.80 | Survivor up vs Non-survivor |
| LDHD | 8.89E-03 | 1.80 | Survivor up vs Non-survivor |
| GSR | 3.11E-02 | 1.80 | Survivor up vs Non-survivor |
| PRG1 | 3.69E-02 | 1.80 | Survivor up vs Non-survivor |
| CLEC4A | 1.27E-02 | 1.80 | Survivor up vs Non-survivor |
| SPATA6 | 8.13E-03 | 1.80 | Survivor up vs Non-survivor |
| UBE2H | 1.39E-02 | 1.82 | Survivor up vs Non-survivor |
| VPS41 | 6.35E-03 | 1.82 | Survivor up vs Non-survivor |
| GLI2 | 8.18E-03 | 1.82 | Survivor up vs Non-survivor |
| RPGRIP1 | 7.93E-03 | 1.83 | Survivor up vs Non-survivor |
| BATF2 | 1.75E-02 | 1.83 | Survivor up vs Non-survivor |
| TBC1D23 | 2.70E-02 | 1.83 | Survivor up vs Non-survivor |
| PIK3CB | 4.13E-03 | 1.83 | Survivor up vs Non-survivor |
| TXNDC9 | 2.57E-03 | 1.84 | Survivor up vs Non-survivor |
| CDKN1A | 4.34E-02 | 1.84 | Survivor up vs Non-survivor |
| STARD8 | 3.53E-02 | 1.84 | Survivor up vs Non-survivor |
| GLIPR1L1 | 1.86E-02 | 1.85 | Survivor up vs Non-survivor |
| TEF | 4.36E-02 | 1.85 | Survivor up vs Non-survivor |
| RIOK3 | 2.82E-03 | 1.85 | Survivor up vs Non-survivor |
| HLA-DRA | 9.63E-03 | 1.85 | Survivor up vs Non-survivor |
| ANKRD22 | 2.76E-02 | 1.87 | Survivor up vs Non-survivor |
| NCOA4 | 4.77E-04 | 1.87 | Survivor up vs Non-survivor |
| ABCC4 | 3.39E-02 | 1.87 | Survivor up vs Non-survivor |
| NR1H2 | 5.78E-03 | 1.87 | Survivor up vs Non-survivor |
| SENP8 | 3.00E-02 | 1.88 | Survivor up vs Non-survivor |
| TAGAP | 4.54E-03 | 1.89 | Survivor up vs Non-survivor |
| HLA-DQA1 | 2.84E-02 | 1.89 | Survivor up vs Non-survivor |
| REN | 2.56E-02 | 1.90 | Survivor up vs Non-survivor |
| HLA-DMA | 3.18E-02 | 1.91 | Survivor up vs Non-survivor |
| ABCC3 | 3.20E-02 | 1.91 | Survivor up vs Non-survivor |
| LEPR | 6.71E-03 | 1.92 | Survivor up vs Non-survivor |
| PRTFDC1 | 3.56E-02 | 1.92 | Survivor up vs Non-survivor |
| FPRL1 | 1.80E-02 | 1.93 | Survivor up vs Non-survivor |
| RHD | 3.65E-02 | 1.93 | Survivor up vs Non-survivor |
| YWHAE | 3.21E-02 | 1.94 | Survivor up vs Non-survivor |
| BMP2K | 3.02E-02 | 1.94 | Survivor up vs Non-survivor |
| ADPRH | 6.50E-03 | 1.94 | Survivor up vs Non-survivor |
| MPP7 | 1.92E-03 | 1.94 | Survivor up vs Non-survivor |
| TMEM16F | 9.80E-03 | 1.94 | Survivor up vs Non-survivor |
| ANGPT1 | 1.12E-02 | 1.94 | Survivor up vs Non-survivor |
| LILRA3 | 4.64E-02 | 1.95 | Survivor up vs Non-survivor |
| FST | 3.76E-02 | 1.95 | Survivor up vs Non-survivor |
| MBOAT2 | 8.99E-03 | 1.96 | Survivor up vs Non-survivor |
| STXBP2 | 6.30E-03 | 1.96 | Survivor up vs Non-survivor |
| FAM44A | 2.51E-02 | 1.96 | Survivor up vs Non-survivor |
| TEX14 | 5.10E-02 | 1.96 | Survivor up vs Non-survivor |
| CEACAM3 | 4.47E-02 | 1.97 | Survivor up vs Non-survivor |
| BPI | 8.43E-03 | 1.97 | Survivor up vs Non-survivor |
| DEFA4 | 4.03E-02 | 1.98 | Survivor up vs Non-survivor |
| IRX5 | 3.23E-03 | 1.99 | Survivor up vs Non-survivor |
| TMEM107 | 2.44E-02 | 2.00 | Survivor up vs Non-survivor |
| CPEB2 | 2.17E-02 | 2.00 | Survivor up vs Non-survivor |
| EMR1 | 3.11E-02 | 2.01 | Survivor up vs Non-survivor |
| FAM92A1 | 6.79E-03 | 2.01 | Survivor up vs Non-survivor |
| EFCAB3 | 3.47E-03 | 2.01 | Survivor up vs Non-survivor |
| GM2A | 2.72E-03 | 2.01 | Survivor up vs Non-survivor |
| CLEC12A | 1.11E-03 | 2.03 | Survivor up vs Non-survivor |
| PARVB | 2.79E-02 | 2.03 | Survivor up vs Non-survivor |
| RNF150 | 1.10E-02 | 2.03 | Survivor up vs Non-survivor |
| NUPL1 | 4.32E-02 | 2.03 | Survivor up vs Non-survivor |
| PDE6H | 1.74E-02 | 2.03 | Survivor up vs Non-survivor |
| CD74 | 5.95E-03 | 2.04 | Survivor up vs Non-survivor |
| GATA1 | 3.13E-02 | 2.04 | Survivor up vs Non-survivor |
| SHOX | 9.57E-03 | 2.04 | Survivor up vs Non-survivor |
| P4HA3 | 1.91E-02 | 2.05 | Survivor up vs Non-survivor |
| NAB1 | 2.00E-02 | 2.05 | Survivor up vs Non-survivor |
| PLXNC1 | 2.30E-02 | 2.05 | Survivor up vs Non-survivor |
| SOCS6 | 1.01E-02 | 2.05 | Survivor up vs Non-survivor |
| TMEM22 | 1.25E-02 | 2.05 | Survivor up vs Non-survivor |
| SMOX | 4.62E-02 | 2.05 | Survivor up vs Non-survivor |
| IGSF2 | 1.65E-02 | 2.06 | Survivor up vs Non-survivor |
| SHPRH | 6.38E-03 | 2.06 | Survivor up vs Non-survivor |
| NR4A3 | 4.45E-02 | 2.06 | Survivor up vs Non-survivor |
| PDCD10 | 7.52E-04 | 2.07 | Survivor up vs Non-survivor |
| TBC1D12 | 1.97E-02 | 2.09 | Survivor up vs Non-survivor |
| EGR1 | 4.27E-02 | 2.11 | Survivor up vs Non-survivor |
| SLC6A10P | 3.24E-02 | 2.11 | Survivor up vs Non-survivor |
| TMTC1 | 4.02E-02 | 2.11 | Survivor up vs Non-survivor |
| APOBEC3A | 3.00E-02 | 2.11 | Survivor up vs Non-survivor |
| SOCS6 | 2.24E-02 | 2.13 | Survivor up vs Non-survivor |
| GABRA2 | 6.30E-03 | 2.13 | Survivor up vs Non-survivor |
| GK | 3.39E-03 | 2.14 | Survivor up vs Non-survivor |
| SNAPC1 | 1.98E-02 | 2.15 | Survivor up vs Non-survivor |
| ITGA1 | 1.00E-02 | 2.19 | Survivor up vs Non-survivor |
| CLEC5A | 2.06E-02 | 2.23 | Survivor up vs Non-survivor |
| PEA15 | 8.32E-03 | 2.24 | Survivor up vs Non-survivor |
| CLEC7A | 3.27E-02 | 2.25 | Survivor up vs Non-survivor |
| TCN1 | 5.00E-02 | 2.26 | Survivor up vs Non-survivor |
| TREM1 | 1.64E-02 | 2.27 | Survivor up vs Non-survivor |
| GPR177 | 2.07E-02 | 2.31 | Survivor up vs Non-survivor |
| BPI | 1.25E-02 | 2.34 | Survivor up vs Non-survivor |
| OAS3 | 2.72E-02 | 2.35 | Survivor up vs Non-survivor |
| HSD17B12 | 3.02E-02 | 2.37 | Survivor up vs Non-survivor |
| EPB49 | 3.17E-02 | 2.45 | Survivor up vs Non-survivor |
| OLFM1 | 3.78E-02 | 2.51 | Survivor up vs Non-survivor |
| ITLN1 | 2.98E-03 | 2.51 | Survivor up vs Non-survivor |
| TAGLN2 | 1.56E-02 | 2.51 | Survivor up vs Non-survivor |
| SLC25A37 | 5.00E-02 | 2.52 | Survivor up vs Non-survivor |
| ZNF701 | 4.57E-03 | 2.54 | Survivor up vs Non-survivor |
| LCN2 | 9.05E-03 | 2.55 | Survivor up vs Non-survivor |
| NEK1 | 2.42E-02 | 2.58 | Survivor up vs Non-survivor |
| DMXL2 | 6.37E-03 | 2.62 | Survivor up vs Non-survivor |
| RASAL2 | 5.40E-03 | 2.62 | Survivor up vs Non-survivor |
| CAPS2 | 1.97E-02 | 2.64 | Survivor up vs Non-survivor |
| LTF | 5.02E-02 | 2.66 | Survivor up vs Non-survivor |
| PLD1 | 4.47E-02 | 2.79 | Survivor up vs Non-survivor |
| RASAL2 | 2.39E-02 | 2.82 | Survivor up vs Non-survivor |
| PDE5A | 2.99E-02 | 2.98 | Survivor up vs Non-survivor |
| IFIT1 | 2.63E-02 | 3.35 | Survivor up vs Non-survivor |
| HBD | 4.27E-02 | 4.24 | Survivor up vs Non-survivor |
| RHCE | 2.32E-02 | 4.97 | Survivor up vs Non-survivor |
| IFI27 | 3.14E-03 | 4.98 | Survivor up vs Non-survivor |
| IFI27 | 3.13E-03 | 5.39 | Survivor up vs Non-survivor |
| CAMP | 2.03E-02 | 5.55 | Survivor up vs Non-survivor |
| SIGLEC1 | 2.80E-03 | 65.25 | Survivor up vs Non-survivor |
